# Novel genetic associations for blood pressure identified via gene-alcohol interaction in up to 570K individuals across multiple ancestries

**DOI:** 10.1371/journal.pone.0198166

**Published:** 2018-06-18

**Authors:** Mary F. Feitosa, Aldi T. Kraja, Daniel I. Chasman, Yun J. Sung, Thomas W. Winkler, Ioanna Ntalla, Xiuqing Guo, Nora Franceschini, Ching-Yu Cheng, Xueling Sim, Dina Vojinovic, Jonathan Marten, Solomon K. Musani, Changwei Li, Amy R. Bentley, Michael R. Brown, Karen Schwander, Melissa A. Richard, Raymond Noordam, Hugues Aschard, Traci M. Bartz, Lawrence F. Bielak, Rajkumar Dorajoo, Virginia Fisher, Fernando P. Hartwig, Andrea R. V. R. Horimoto, Kurt K. Lohman, Alisa K. Manning, Tuomo Rankinen, Albert V. Smith, Salman M. Tajuddin, Mary K. Wojczynski, Maris Alver, Mathilde Boissel, Qiuyin Cai, Archie Campbell, Jin Fang Chai, Xu Chen, Jasmin Divers, Chuan Gao, Anuj Goel, Yanick Hagemeijer, Sarah E. Harris, Meian He, Fang-Chi Hsu, Anne U. Jackson, Mika Kähönen, Anuradhani Kasturiratne, Pirjo Komulainen, Brigitte Kühnel, Federica Laguzzi, Jian'an Luan, Nana Matoba, Ilja M. Nolte, Sandosh Padmanabhan, Muhammad Riaz, Rico Rueedi, Antonietta Robino, M. Abdullah Said, Robert A. Scott, Tamar Sofer, Alena Stančáková, Fumihiko Takeuchi, Bamidele O. Tayo, Peter J. van der Most, Tibor V. Varga, Veronique Vitart, Yajuan Wang, Erin B. Ware, Helen R. Warren, Stefan Weiss, Wanqing Wen, Lisa R. Yanek, Weihua Zhang, Jing Hua Zhao, Saima Afaq, Najaf Amin, Marzyeh Amini, Dan E. Arking, Tin Aung, Eric Boerwinkle, Ingrid Borecki, Ulrich Broeckel, Morris Brown, Marco Brumat, Gregory L. Burke, Mickaël Canouil, Aravinda Chakravarti, Sabanayagam Charumathi, Yii-Der Ida Chen, John M. Connell, Adolfo Correa, Lisa de las Fuentes, Renée de Mutsert, H. Janaka de Silva, Xuan Deng, Jingzhong Ding, Qing Duan, Charles B. Eaton, Georg Ehret, Ruben N. Eppinga, Evangelos Evangelou, Jessica D. Faul, Stephan B. Felix, Nita G. Forouhi, Terrence Forrester, Oscar H. Franco, Yechiel Friedlander, Ilaria Gandin, He Gao, Mohsen Ghanbari, Bruna Gigante, C. Charles Gu, Dongfeng Gu, Saskia P. Hagenaars, Göran Hallmans, Tamara B. Harris, Jiang He, Sami Heikkinen, Chew-Kiat Heng, Makoto Hirata, Barbara V. Howard, M. Arfan Ikram, InterAct Consortium, Ulrich John, Tomohiro Katsuya, Chiea Chuen Khor, Tuomas O. Kilpeläinen, Woon-Puay Koh, José E. Krieger, Stephen B. Kritchevsky, Michiaki Kubo, Johanna Kuusisto, Timo A. Lakka, Carl D. Langefeld, Claudia Langenberg, Lenore J. Launer, Benjamin Lehne, Cora E. Lewis, Yize Li, Shiow Lin, Jianjun Liu, Jingmin Liu, Marie Loh, Tin Louie, Reedik Mägi, Colin A. McKenzie, Thomas Meitinger, Andres Metspalu, Yuri Milaneschi, Lili Milani, Karen L. Mohlke, Yukihide Momozawa, Mike A. Nalls, Christopher P. Nelson, Nona Sotoodehnia, Jill M. Norris, Jeff R. O'Connell, Nicholette D. Palmer, Thomas Perls, Nancy L. Pedersen, Annette Peters, Patricia A. Peyser, Neil Poulter, Leslie J. Raffel, Olli T. Raitakari, Kathryn Roll, Lynda M. Rose, Frits R. Rosendaal, Jerome I. Rotter, Carsten O. Schmidt, Pamela J. Schreiner, Nicole Schupf, William R. Scott, Peter S. Sever, Yuan Shi, Stephen Sidney, Mario Sims, Colleen M. Sitlani, Jennifer A. Smith, Harold Snieder, John M. Starr, Konstantin Strauch, Heather M. Stringham, Nicholas Y. Q. Tan, Hua Tang, Kent D. Taylor, Yik Ying Teo, Yih Chung Tham, Stephen T. Turner, André G. Uitterlinden, Peter Vollenweider, Melanie Waldenberger, Lihua Wang, Ya Xing Wang, Wen Bin Wei, Christine Williams, Jie Yao, Caizheng Yu, Jian-Min Yuan, Wei Zhao, Alan B. Zonderman, Diane M. Becker, Michael Boehnke, Donald W. Bowden, John C. Chambers, Ian J. Deary, Tõnu Esko, Martin Farrall, Paul W. Franks, Barry I. Freedman, Philippe Froguel, Paolo Gasparini, Christian Gieger, Jost Bruno Jonas, Yoichiro Kamatani, Norihiro Kato, Jaspal S. Kooner, Zoltán Kutalik, Markku Laakso, Cathy C. Laurie, Karin Leander, Terho Lehtimäki, Lifelines Cohort Study, Patrik K. E. Magnusson, Albertine J. Oldehinkel, Brenda W. J. H. Penninx, Ozren Polasek, David J. Porteous, Rainer Rauramaa, Nilesh J. Samani, James Scott, Xiao-Ou Shu, Pim van der Harst, Lynne E. Wagenknecht, Nicholas J. Wareham, Hugh Watkins, David R. Weir, Ananda R. Wickremasinghe, Tangchun Wu, Wei Zheng, Claude Bouchard, Kaare Christensen, Michele K. Evans, Vilmundur Gudnason, Bernardo L. Horta, Sharon L. R. Kardia, Yongmei Liu, Alexandre C. Pereira, Bruce M. Psaty, Paul M. Ridker, Rob M. van Dam, W. James Gauderman, Xiaofeng Zhu, Dennis O. Mook-Kanamori, Myriam Fornage, Charles N. Rotimi, L. Adrienne Cupples, Tanika N. Kelly, Ervin R. Fox, Caroline Hayward, Cornelia M. van Duijn, E Shyong Tai, Tien Yin Wong, Charles Kooperberg, Walter Palmas, Kenneth Rice, Alanna C. Morrison, Paul Elliott, Mark J. Caulfield, Patricia B. Munroe, Dabeeru C. Rao, Michael A. Province, Daniel Levy

**Affiliations:** 1 Division of Statistical Genomics, Department of Genetics, Washington University School of Medicine, St. Louis, Missouri, United States of America; 2 Preventive Medicine, Brigham and Women's Hospital, Boston, Massachusetts, United States of America; 3 Harvard Medical School, Boston, Massachusetts, United States of America; 4 Division of Biostatistics, Washington University School of Medicine, St. Louis, Missouri, United States of America; 5 Department of Genetic Epidemiology, University of Regensburg, Regensburg, Germany; 6 Clinical Pharmacology, William Harvey Research Institute, Barts and The London School of Medicine and Dentistry, Queen Mary University of London, London, United Kingdom; 7 Genomic Outcomes, Pediatrics, Institute for Translational Genomics and Population Sciences, LABioMed at Harbor-UCLA Medical Center, Torrance, California, United States of America; 8 Epidemiology, University of North Carolina Gilling School of Global Public Health, Chapel Hill, North Carolina, United States of America; 9 Singapore Eye Research Institute, Singapore National Eye Centre, Singapore, Singapore; 10 Ophthalmology & Visual Sciences Academic Clinical Program (Eye ACP), Duke-NUS Medical School, Singapore, Singapore; 11 Department of Ophthalmology, Yong Loo Lin School of Medicine, National University of Singapore, Singapore, Singapore; 12 Saw Swee Hock School of Public Health, National University Health System and National University of Singapore, Singapore, Singapore; 13 Department of Epidemiology, Erasmus University Medical Center, Rotterdam, The Netherlands; 14 Medical Research Council Human Genetics Unit, Institute of Genetics and Molecular Medicine, University of Edinburgh, Edinburgh, United Kingdom; 15 Jackson Heart Study, Department of Medicine, University of Mississippi Medical Center, Jackson, Mississippi, United States of America; 16 Epidemiology and Biostatistics, University of Georgia at Athens College of Public Health, Athens, Georgia, United States of America; 17 Center for Research on Genomics and Global Health, National Human Genome Research Institute, National Institutes of Health, Bethesda, Maryland, United States of America; 18 Human Genetics Center, Department of Epidemiology, Human Genetics, and Environmental Sciences, The University of Texas Health Science Center at Houston, Houston, Texas, United States of America; 19 Brown Foundation Institute of Molecular Medicine, The University of Texas Health Science Center at Houston, Houston, Texas, United States of America; 20 Internal Medicine, Gerontology and Geriatrics, Leiden University Medical Center, Leiden, The Netherlands; 21 Department of Epidemiology, Harvard School of Public Health, Boston, Massachusetts, United States of America; 22 Centre de Bioinformatique, Biostatistique et Biologie Intégrative (C3BI), Institut Pasteur, Paris, France; 23 Cardiovascular Health Research Unit, Biostatistics and Medicine, University of Washington, Seattle, Washington, United States of America; 24 Department of Epidemiology, School of Public Health, University of Michigan, Ann Arbor, Michigan, United States of America; 25 Genome Institute of Singapore, Agency for Science Technology and Research, Singapore, Singapore; 26 Biostatistics, Boston University School of Public Health, Boston, Massachusetts, United States of America; 27 Postgraduate Programme in Epidemiology, Federal University of Pelotas, Pelotas, RS, Brazil; 28 Medical Research Council Integrative Epidemiology Unit, University of Bristol, Bristol, United Kingdom; 29 Laboratory of Genetics and Molecular Cardiology, Heart Institute (InCor), University of São Paulo Medical School, São Paulo, SP, Brazil; 30 Biostatistical Sciences, Public Health Sciences, Wake Forest School of Medicine, Winston-Salem, North Carolina, United States of America; 31 Clinical and Translational Epidemiology Unit, Massachusetts General Hospital, Boston, Massachusetts, United States of America; 32 Department of Medicine, Harvard Medical School, Boston, Massachusetts, United States of America; 33 Human Genomics Laboratory, Pennington Biomedical Research Center, Baton Rouge, Louisiana, United States of America; 34 Icelandic Heart Association, Kopavogur, Iceland; 35 Faculty of Medicine, University of Iceland, Reykjavik, Iceland; 36 Health Disparities Research Section, Laboratory of Epidemiology and Population Sciences, National Institute on Aging, National Institutes of Health, Baltimore, Maryland, United States of America; 37 Estonian Genome Center, University of Tartu, Tartu, Estonia; 38 CNRS UMR 8199, European Genomic Institute for Diabetes (EGID), Institut Pasteur de Lille, University of Lille, Lille, France; 39 Division of Epidemiology, Department of Medicine, Vanderbilt University School of Medicine, Nashville, Tennessee, United States of America; 40 Centre for Genomic & Experimental Medicine, Institute of Genetics & Molecular Medicine, University of Edinburgh, Edinburgh, United Kingdom; 41 Department of Medical Epidemiology and Biostatistics, Karolinska Institutet, Stockholm, Stockholm, Sweden; 42 Molecular Genetics and Genomics Program, Wake Forest School of Medicine, Winston-Salem, North Carolina, United States of America; 43 Division of Cardiovascular Medicine, Radcliffe Department of Medicine, University of Oxford, Oxford, Oxfordshire, United Kingdom; 44 Wellcome Centre for Human Genetics, University of Oxford, Oxford, Oxfordshire, United Kingdom; 45 Department of Cardiology, University of Groningen, University Medical Center Groningen, Groningen, The Netherlands; 46 Centre for Cognitive Ageing and Cognitive Epidemiology, The University of Edinburgh, Edinburgh, United Kingdom; 47 Medical Genetics Section, Centre for Genomic and Experimental Medicine and MRC Institute of Genetics and Molecular Medicine, The University of Edinburgh, Edinburgh, United Kingdom; 48 Department of Occupational and Environmental Health, State Key Laboratory of Environmental Health for Incubating, Tongji Medical College, Huazhong University of Science and Technology, Wuhan, China; 49 Department of Biostatistics and Center for Statistical Genetics, University of Michigan, Ann Arbor, Michigan, United States of America; 50 Department of Clinical Physiology, Tampere University Hospital, Tampere, Finland; 51 University of Tampere, Tampere, Finland; 52 Department of Public Health, Faculty of Medicine, University of Kelaniya, Ragama, Sri Lanka; 53 Foundation for Research in Health Exercise and Nutrition, Kuopio Research Institute of Exercise Medicine, Kuopio, Finland; 54 Research Unit of Molecular Epidemiology, Helmholtz Zentrum München, German Research Center for Environmental Health, Neuherberg, Germany; 55 Institute of Epidemiology II, Helmholtz Zentrum München, German Research Center for Environmental Health, Neuherberg, Germany; 56 Unit of Cardiovascular Epidemiology, Institute of Environmental Medicine, Karolinska Institutet, Stockholm, Sweden; 57 MRC Epidemiology Unit, University of Cambridge, Cambridge, United Kingdom; 58 Laboratory for Statistical Analysis, Center for Integrative Medical Sciences, RIKEN, Yokohama, Japan; 59 Department of Epidemiology, University of Groningen, University Medical Center Groningen, Groningen, The Netherlands; 60 Institute of Cardiovascular and Medical Sciences, University of Glasgow, Glasgow, United Kingdom; 61 Department of Cardiovascular Sciences, University of Leicester, Leicester, United Kingdom; 62 NIHR Leicester Biomedical Research Centre, Glenfield Hospital, Leicester, United Kingdom; 63 Department of Computational Biology, University of Lausanne, Lausanne, Switzerland; 64 Swiss Instititute of Bioinformatics, Lausanne, Switzerland; 65 Institute for Maternal and Child Health—IRCCS "Burlo Garofolo", Trieste, Italy; 66 Division of Sleep and Circadian Disorders, Brigham and Women's Hospital, Boston, MA, United States of America; 67 Institute of Clinical Medicine, Internal Medicine, University of Eastern Finland, Kuopio, Finland; 68 Department of Gene Diagnostics and Therapeutics, Research Institute, National Center for Global Health and Medicine, Tokyo, Japan; 69 Department of Public Health Sciences, Loyola University Chicago, Maywood, Illinois, United States of America; 70 Department of Clinical Sciences, Genetic and Molecular Epidemiology Unit, Lund University Diabetes Centre, Skåne University Hospital, Malmö, Sweden; 71 Department of Epidemiology and Biostatistics, Case Western Reserve University, Cleveland, Ohio, United States of America; 72 Survey Research Center, Institute for Social Research, University of Michigan, Ann Arbor, Michigan, United States of America; 73 NIHR Barts Cardiovascular Biomedical Research Unit, Queen Mary University of London, London, London, United Kingdom; 74 Interfaculty Institute for Genetics and Functional genomics, University Medicine Ernst Moritz Arndt University Greifsald, Greifswald, Germany; 75 DZHK (German Center for Cardiovascular Research), partner site Greifswald, Greifswald, Germany; 76 Division of General Internal Medicine, Department of Medicine, Johns Hopkins University School of Medicine, Baltimore, Maryland, United States of America; 77 Department of Epidemiology and Biostatistics, Imperial College London, London, United Kingdom; 78 Department of Cardiology, Ealing Hospital, Middlesex, United Kingdom; 79 McKusick-Nathans Institute of Genetic Medicine, Johns Hopkins University School of Medicine, Baltimore, Maryland, United States of America; 80 Department of Epidemiology, Human Genetics, and Environmental Sciences, The University of Texas School of Public Health, Houston, Texas, United States of America; 81 Human Genome Sequencing Center, Baylor College of Medicine, Houston, Texas, United States of America; 82 Section of Genomic Pediatrics, Department of Pediatrics, Medicine and Physiology, Medical College of Wisconsin, Milwaukee, Wisconsin, United States of America; 83 Department of Medical Sciences, University of Trieste, Trieste, Italy; 84 Public Health Sciences, Wake Forest School of Medicine, Winston-Salem, North Carolina, United States of America; 85 Ninewells Hospital & Medical School, University of Dundee, Dundee, Scotland, United Kingdom; 86 Cardiovascular Division, Department of Medicine, Washington University, St. Louis, Missouri, United States of America; 87 Clinical Epidemiology, Leiden University Medical Center, Leiden, The Netherlands; 88 Department of Medicine, Faculty of Medicine, University of Kelaniya, Ragama, Sri Lanka; 89 Center on Diabetes, Obesity, and Metabolism, Gerontology and Geriatric Medicine, Wake Forest University Health Sciences, Winston-Salem, North Carolina, United States of America; 90 Department of Genetics, University of North Carolina, Chapel Hill, North Carolina, United States of America; 91 Department of Family Medicine and Epidemiology, Alpert Medical School of Brown University, Providence, Rhode Island, United States of America; 92 Cardiology, Geneva University Hospital, Geneva, Switzerland; 93 Department of Hygiene and Epidemiology, University of Ioannina Medical School, Ioannina, Greece; 94 Department of Internal Medicine B, University Medicine Greifswald, Greifswald, Germany; 95 The Caribbean Institute for Health Research (CAIHR), University of the West Indies, Mona, Jamaica; 96 Braun School of Public Health, Hebrew University-Hadassah Medical Center, Jerusalem, Israel; 97 Department of Genetics, School of Medicine, Mashhad University of Medical Sciences, Mashhad, Iran; 98 Department of Epidemiology, State Key Laboratory of Cardiovascular Disease, Fuwai Hospital, National Center of Cardiovascular Diseases, Chinese Academy of Medical Sciences and Peking Union Medical College, Beijing, China; 99 Psychology, The University of Edinburgh, Edinburgh, United Kingdom; 100 Department of Public Health and Clinical Medicine, Nutritional Research, Umeå University, Umeå, Västerbotten, Sweden; 101 Laboratory of Epidemiology and Population Sciences, National Institute on Aging, National Institutes of Health, Bethesda, Maryland, United States of America; 102 Epidemiology, Tulane University School of Public Health and Tropical Medicine, New Orleans, Louisiana, United States of America; 103 Medicine, Tulane University School of Medicine, New Orleans, Louisiana, United States of America; 104 Institute of Biomedicine, School of Medicine, University of Eastern Finland, Kuopio Campus, Finland; 105 Department of Paediatrics, Yong Loo Lin School of Medicine, National University of Singapore, Singapore; 106 Khoo Teck Puat–National University Children's Medical Institute, National University Health System, Singapore; 107 Laboratory of Genome Technology, Human Genome Center, Institute of Medical Science, The University of Tokyo, Minato-ku, Japan; 108 MedStar Health Research Institute, Hyattsville, Maryland, United States of America; 109 Center for Clinical and Translational Sciences and Department of Medicine, Georgetown-Howard Universities, Washington, DC, United States of America; 110 Department of Radiology and Nuclear Medicine, Erasmus University Medical Center, Rotterdam, The Netherlands; 111 Department of Neurology, Erasmus University Medical Center, Rotterdam, The Netherlands; 112 Institute of Social Medicine and Prevention, University Medicine Greifswald, Greifswald, Germany; 113 Department of Clinical Gene Therapy, Osaka University Graduate School of Medicine, Suita, Japan; 114 Department of Geriatric Medicine and Nephrology, Osaka University Graduate School of Medicine, Suita, Japan; 115 Department of Biochemistry, National University of Singapore, Singapore, Singapore; 116 Novo Nordisk Foundation Center for Basic Metabolic Research, Section of Metabolic Genetics, Faculty of Health and Medical Sciences, University of Copenhagen, Copenhagen, Denmark; 117 Department of Environmental Medicine and Public Health, The Icahn School of Medicine at Mount Sinai, New York, New York, United States of America; 118 Duke-NUS Medical School, Singapore, Singapore; 119 Sticht Center for Healthy Aging and Alzheimer's Prevention, Department of Internal Medicine, Wake Forest School of Medicine, Winston-Salem, North Carolina, United States of America; 120 Center for Integrative Medical Sciences, RIKEN, Yokohama, Japan; 121 Department of Clinical Physiology and Nuclear Medicine, Kuopio University Hospital, Kuopio, Finland; 122 Department of Medicine, University of Alabama at Birmingham, Birmingham, AL, United States of America; 123 WHI CCC, Fred Hutchinson Cancer Research Center, Seattle, Washington, United States of America; 124 Translational Laboratory in Genetic Medicine, Agency for Science, Technology and Research, Singapore; 125 Department of Biostatistics, University of Washington, Seattle, Washington, United States of America; 126 Institute of Human Genetics, Helmholtz Zentrum München, German Research Center for Environmental Health, Neuherberg, Germany; 127 Institute of Human Genetics, Technische Universität München, Munich, Germany; 128 Department of Psychiatry, Amsterdam Neuroscience and Amsterdam Public Health Research Institute, VU University Medical Center, Amsterdam, The Netherlands; 129 Laboratory for Genotyping Development, Center for Integrative Medical Sciences, RIKEN, Yokohama, Japan; 130 Data Tecnica International, Glen Echo, Maryland, United States of America; 131 Laboratory of Neurogenetics, National Institute on Aging, Bethesda, Maryland, United States of America; 132 Cardiovascular Health Research Unit, Division of Cardiology, University of Washington, Seattle, Washington, United States of America; 133 Department of Epidemiology, Colorado School of Public Health, Aurora, Colorado, United States of America; 134 Division of Endocrinology, Diabetes, and Nutrition, University of Maryland School of Medicine, Baltimore, Maryland, United States of America; 135 Program for Personalized and Genomic Medicine, University of Maryland School of Medicine, Baltimore, Maryland, United States of America; 136 Biochemistry, Wake Forest School of Medicine, Winston-Salem, North Carolina, United States of America; 137 Geriatrics Section, Boston University Medical Center, Boston, Massachusetts, United States of America; 138 DZHK (German Centre for Cardiovascular Research), partner site Munich Heart Alliance, Neuherberg, Germany; 139 School of Public Health, Imperial College London, London, London, United Kingdom; 140 Division of Genetic and Genomic Medicine, Department of Pediatrics, University of California, Irvine, California, United States of America; 141 Department of Clinical Physiology and Nuclear Medicine, Turku University Hospital, Turku, Finland; 142 Research Centre of Applied and Preventive Cardiovascular Medicine, University of Turku, Turku, Finland; 143 Institute for Community Medicine, University Medicine Greifswald, Greifswald, Germany; 144 Epidemiology & Community Health, School of Public Health, University of Minnesota, Minneapolis, Minnesota, United States of America; 145 Taub Institute for Research on Alzheimer’s Disease and the Aging Brain, Columbia University Medical Center, New York, New York, United States of America; 146 National Heart and Lung Institute, Imperial College London, London, United Kingdom; 147 Division of Research, Kaiser Permanente of Northern California, Oakland, California, United States of America; 148 Cardiovascular Health Research Unit, Medicine, University of Washington, Seattle, Washington, United States of America; 149 Alzheimer Scotland Dementia Research Centre, The University of Edinburgh, Edinburgh, United Kingdom; 150 Institute of Genetic Epidemiology, Helmholtz Zentrum München, German Research Center for Environmental Health, Neuherberg, Germany; 151 Chair of Genetic Epidemiology, IBE, Faculty of Medicine, LMU, Munich, Germany; 152 Department of Genetics, Stanford University, Stanford, California, United States of America; 153 Life Sciences Institute, National University of Singapore, Singapore, Singapore; 154 NUS Graduate School for Integrative Science and Engineering, National University of Singapore, Singapore, Singapore; 155 Department of Statistics and Applied Probability, National University of Singapore, Singapore, Singapore; 156 Division of Nephrology and Hypertension, Mayo Clinic, Rochester, Minnesota, United States of America; 157 Department of Internal Medicine, Erasmus University Medical Center, Rotterdam, The Netherlands; 158 Service of Internal Medicine, Department of Internal Medicine, University Hospital, Lausanne, Switzerland; 159 Beijing Institute of Ophthalmology, Beijing Ophthalmology and Visual Science Key Lab, Beijing Tongren Eye Center, Capital Medical University, Beijing, China; 160 Beijing Tongren Eye Center, Beijing Tongren Hospital, Capital Medical University, Beijing, China; 161 Department of Epidemiology, Graduate School of Public Health, University of Pittsburgh, Pittsburgh, Pennsylvania, United States of America; 162 Division of Cancer Control and Population Sciences, UPMC Hillman Cancer, University of Pittsburgh, Pittsburgh, Pennsylvania, United States of America; 163 Behavioral Epidemiology Section, Laboratory of Epidemiology and Population Sciences, National Institute on Aging, National Institutes of Health, Baltimore, Maryland, United States of America; 164 Lee Kong Chian School of Medicine, Nanyang Technological University, Singapore, Singapore; 165 Imperial College Healthcare NHS Trust, London, United Kingdom; 166 MRC-PHE Centre for Environment and Health, Department of Epidemiology & Biostatistics, School of Public Health, Imperial College London, London, United Kingdom; 167 Broad Institute of the Massachusetts Institute of Technology and Harvard University, Boston, Massachusetts, United States of America; 168 Harvard T. H. Chan School of Public Health, Department of Nutrition, Harvard University, Boston, Massachusetts, United States of America; 169 Nephrology, Internal Medicine, Wake Forest School of Medicine, Winston-Salem, North Carolina, United States of America; 170 Department of Genomics of Common Disease, Imperial College London, London, United Kingdom; 171 German Center for Diabetes Research (DZD e.V.), Neuherberg, Germany; 172 Department of Ophthalmology, Medical Faculty Mannheim, University Heidelberg, Mannheim, Germany, Germany; 173 Institute of Social and Preventive Medicine, Lausanne University Hospital, Lausanne, Switzerland; 174 Department of Clinical Chemistry, Fimlab Laboratories, Tampere, Finland; 175 Department of Clinical Chemistry, Finnish Cardiovascular Research Center—Tampere, Faculty of Medicine and Life Sciences, University of Tampere, Tampere, Finland; 176 Lifelines Cohort, Groningen, The Netherlands; 177 Department of Psychiatry, University of Groningen, University Medical Center Groningen, Groningen, The Netherlands; 178 Department of Public Health, Department of Medicine, University of Split, Split, Croatia; 179 Psychiatric Hospital "Sveti Ivan", Zagreb, Croatia; 180 Gen-info Ltd, Zagreb, Croatia; 181 Department of Genetics, University of Groningen, University Medical Center Groningen, Groningen, The Netherlands; 182 The Danish Aging Research Center, Institute of Public Health, University of Southern Denmark, Odense, Denmark; 183 Public Health Sciences, Epidemiology and Prevention, Wake Forest University Health Sciences, Winston-Salem, North Carolina, United States of America; 184 Cardiovascular Health Research Unit, Epidemiology, Medicine and Health Services, University of Washington, Seattle, Washington, United States of America; 185 Kaiser Permanente Washington, Health Research Institute, Seattle, Washington, United States of America; 186 Department of Medicine, Yong Loo Lin School of Medicine, National University of Singapore, Singapore, Singapore; 187 Biostatistics, Preventive Medicine, University of Southern California, Los Angeles, California, United States of America; 188 Public Health and Primary Care, Leiden University Medical Center, Leiden, The Netherlands; 189 The Framingham Heart Study, Framingham, Massachusetts, United States of America; 190 Cardiology, Medicine, University of Mississippi Medical Center, Jackson, Mississippi, United States of America; 191 Fred Hutchinson Cancer Research Center, University of Washington School of Public Health, Seattle, Washington, United States of America; 192 Medicine, Columbia University Medical Center, New York, New York, United States of America; 193 The Population Sciences Branch, National Heart, Lung, and Blood Institute, National Institutes of Health, Bethesda, Maryland, United States of America; Stellenbosch University Faculty of Medicine and Health Sciences, SOUTH AFRICA

## Abstract

Heavy alcohol consumption is an established risk factor for hypertension; the mechanism by which alcohol consumption impact blood pressure (BP) regulation remains unknown. We hypothesized that a genome-wide association study accounting for gene-alcohol consumption interaction for BP might identify additional BP loci and contribute to the understanding of alcohol-related BP regulation. We conducted a large two-stage investigation incorporating joint testing of main genetic effects and single nucleotide variant (SNV)-alcohol consumption interactions. In Stage 1, genome-wide discovery meta-analyses in ≈131K individuals across several ancestry groups yielded 3,514 SNVs (245 loci) with suggestive evidence of association (*P* < 1.0 x 10^−5^). In Stage 2, these SNVs were tested for independent external replication in ≈440K individuals across multiple ancestries. We identified and replicated (at Bonferroni correction threshold) five novel BP loci (380 SNVs in 21 genes) and 49 previously reported BP loci (2,159 SNVs in 109 genes) in European ancestry, and in multi-ancestry meta-analyses (*P* < 5.0 x 10^−8^). For African ancestry samples, we detected 18 potentially novel BP loci (*P* < 5.0 x 10^−8^) in Stage 1 that warrant further replication. Additionally, correlated meta-analysis identified eight novel BP loci (11 genes). Several genes in these loci (*e*.*g*., *PINX1*, *GATA4*, *BLK*, *FTO* and *GABBR2*) have been previously reported to be associated with alcohol consumption. These findings provide insights into the role of alcohol consumption in the genetic architecture of hypertension.

## Introduction

Hypertension is a major risk factor for cardiovascular disease (CVD)[1], which in 2015 alone was estimated to cause about 10.7 million deaths worldwide[2]. The prevalence of hypertension in the US is ~46% for those of African ancestry compared to ~33% for European ancestry and ~30% for Hispanic ancestry[3] based on previous blood pressure (BP) guidelines (The Seventh Report of the Joint National Committee on Prevention)[4]. Recently, based on the 2017 American College of Cardiology/ American Heart Association high BP guideline, the overall prevalence of hypertension among US adults is estimated at 45.6%[5]. Blood pressure levels are influenced by alcohol consumption independently of adiposity, sodium intake, smoking and socio-economic status[6]. Alcohol shows a dose-dependent effect on systolic BP (SBP) after adjusting for environmental confounders[7].

Genome-wide association studies (GWAS) have identified more than 400 single nucleotide variants (SNVs) for BP[8–14] and about 30 SNVs for alcohol consumption[15–17]. However, few studies have explored SNV-alcohol interactions in relation to BP[18, 19], in part due to the large sample sizes required to obtain adequate power[18, 20]. SNVs, which effect differ by level of alcohol consumption, can harbor modest marginal effects and might therefore be missed by standard marginal effects association screening. As previously demonstrated, a joint test of main genetic effect and gene-environmental interaction can have higher power[21] to identify such variants.

Within the CHARGE Gene-Lifestyle Interactions Working Group[22, 23], we studied a total of 571,652 adults across multiple ancestries to identify variants associated with SBP, diastolic BP (DBP), mean arterial pressure (MAP), and pulse pressure (PP). We tested a model that included a joint model of SNV main effect on BP and SNV-alcohol consumption interaction, in each ancestry and across ancestries. Alcohol consumption was defined by two categories: (I) as current drinking (yes/no), and (II) in the subset of drinkers, as light/heavy drinking (1–7 drinks/week or ≥8 drinks/week). Individual cohort results were meta-analyzed using a modified version of METAL applicable to the statistics summary results accounting for interactions[24]. We also performed multi-trait correlated meta-analyses[25, 26] in participants of European ancestry using the joint model *P*-values from each meta-analysis of all four BP traits.

## Results

### Genetic associations for BP identified via gene-alcohol interaction

The overall description of the CHARGE Gene-Lifestyle Interactions Working Group was previously reported[22, 23]. We studied the joint model of SNV main effect and SNV-alcohol consumption interaction for BP in a two-stage study design, as depicted in S1 Fig. GWAS discovery (Stage 1), was conducted in each of 47 multi-ancestry cohorts including a total of 130,828 individuals of African ancestry (N = 21,417), Asian ancestry (N = 9,838), Brazilian (4,415), European ancestry (N = 91,102), and Hispanic ancestry (N = 4,056) (S1–S4 Tables and S1 Note). A total of 3,514 SNVs (245 loci) attained *P* < 1.0 x 10^−5^ in Stage 1 meta-analyses (for at least one combination of BP trait and alcohol consumption status in one ancestry or multi-ancestries). We considered a locus to be independent, if our lead variant (i.e., most significant) was in low linkage disequilibrium (LD, r^2^ ≤ 0.2) and at least 500 kb away from any variant associated with BP in previous GWAS (*P* ≤ 5.0 x 10^−8^). The meta-analysis distributions of–log_10_ *P*-values of observed versus–log_10_ *P*-values expected (QQ plots) are shown in S2 and S3 Figs.

The 3,514 SNVs were taken forward to replication, Stage 2, which included 440,824 individuals from 68 cohorts of African ancestry (N = 5,041), Asian ancestry (N = 141,026), European ancestry (N = 281,380), and Hispanic ancestry (N = 13,377, S5–S8 Tables and S1 Note). We identified and replicated (Stage 2, at Bonferroni correction *P* < 0.0002) five novel BP loci in European ancestry, four loci on 8p23.1 and one locus (*FTO*) on 16q12.2, which included 380 SNVs in 21 genes. These findings achieved genome-wide statistical significance (*P* < 5.0 x 10^−8^) in Stage 1 and Stage 2 combined meta-analyses. Tables 1 and 2 show the most significant SNVs per BP trait, per alcohol consumption and gene for European ancestry participants. The loci containing novel BP associations at 8p23.1 were detected for all four BP traits in current drinkers and in light/heavy drinkers. The regional association plots on chromosomes 8p23 and 16q12 in European ancestry are shown in S4 and S5 Figs. For African ancestry, 18 potentially novel BP loci were found in discovery (*P* ≤ 5.0 x 10^−8^), but without replication (Table 3). Further, we performed combined meta-analyses of Stage 1 and Stage 2 across all ancestries, which reproduced our European ancestry findings (*P* ≤ 5.0 x 10^−8^, Table 4 and S9 Table). We also identified and replicated 49 previously reported BP loci (2,159 SNVs in 109 genes) for European ancestry participants (S10 Table). For African Ancestry, and multi-ancestry analyses, additional reported BP loci were significant (*P* < 5.0 x 10^−8^) in Stage 1 and Stage 2 combined meta-analyses (S11 and S12 Tables). Manhattan plots for BP trait and alcohol consumption status are shown in S6–S15 Figs, for Stage 1 and Stage 2 combined meta-analyses of European, African and Asian ancestries.

**Table 1:** Novel SNVs/Genes associated with BP traits in European ancestry.

										Stage 1 (S1)			Stage 2 (S2)			S1 & S2
SNV	Chr	Position	Gene	Near Gene	Role	A1/2	Frq1	Trait	Drink	b_M	b_I	*P*-Value	b_M	b_I	*P*-Value	*P*-Meta
rs2979172	8	8452998	LOC107986913	SGK223		C/G	0.48	PP	LHD	0.24	0.25	7.59 x 10^−6^	0.32	-0.20	5.13 X 10^−6^	6.17 X 10^−10^
rs2921064	8	8459127	LOC107986913	SGK223		T/C	0.45	PP	CURD	0.19	0.10	7.76 X 10^−6^	0.24	-0.02	3.63 X 10^−9^	2.69 x 10^−14^
rs2979181	8	8465578	LOC107986913	SGK223		A/T	0.52	SBP	CURD	-0.25	-0.23	9.33 x 10^−8^	-0.35	0.01	1.15 x 10^−10^	7.41 x 10^−18^
rs2979181	8	8465578	LOC107986913	SGK223		A/T	0.52	SBP	LHD	-0.47	-0.14	5.37 x 10^−7^	-0.42	0.16	4.79 x 10^−5^	3.98 x 10^−11^
rs2980755	8	8506173	LOC105379224	SGK223		A/G	0.55	PP	LHD	-0.28	-0.20	4.17 x 10^−6^	-0.32	0.17	4.90 x 10^−6^	1.35 x 10^−10^
rs2980755	8	8506173	LOC105379224	SGK223		A/G	0.55	SBP	LHD	-0.49	-0.20	2.63 x 10^−7^	-0.42	0.12	5.25 x 10^−5^	2.51 x 10^−11^
rs13270194	8	8520592	LOC105379224	SGK223		T/C	0.51	SBP	CURD	-0.26	-0.24	2.46 x 10^−8^	-0.42	0.05	1.23 x 10^−12^	2.34 x 10^−20^
rs6995407	8	8527137	LOC105379224	SGK223		C/G	0.51	PP	CURD	-0.16	-0.15	7.59 x 10^−7^	-0.25	0.02	2.34 x 10^−10^	2.34 x 10^−16^
rs453301	8	9172877	LOC102724880	PPP1R3B		T/G	0.51	SBP	CURD	-0.17	-0.33	1.59 x 10^−6^	-0.27	-0.08	8.13 x 10^−10^	1.23 x 10^−15^
rs11774915	8	9331252	LOC157273		Intron	T/C	0.33	SBP	CURD	0.45	0.01	1.02 x 10^−7^	0.35	-0.05	7.94 x 10^−8^	8.91 x 10^−15^
rs6601302	8	9381948	LOC105379231	LOC157273	Intron	T/G	0.24	SBP	CURD	0.35	0.17	7.94 x 10^−7^	0.20	0.06	7.59 x 10^−5^	2.57 x 10^−10^
rs35231275	8	9762399	TNKS		Intron	A/T	0.31	PP	CURD	-0.38	0.03	1.26 x 10^−6^	-0.05	-0.12	3.31 x 10^−4^	1.35 x 10^−8^
rs1976671	8	9822124	TNKS			A/G	0.62	SBP	CURD	-0.21	-0.31	4.68 x 10^−8^	-0.37	-0.02	2.24 x 10^−10^	7.24 x 10^−18^
rs55868514	8	9822890	TNKS			T/C	0.38	DBP	CURD	0.20	0.09	1.32 x 10^−6^	0.17	0.01	1.20 x 10^−7^	1.70 x 10^−13^
rs483916	8	9936091	MIR124-1			A/C	0.47	DBP	CURD	0.25	0.01	1.18 x 10^−6^	0.04	0.14	1.29 x 10^−6^	5.89 x 10^−12^
rs483916	8	9936091	MIR124-1			A/C	0.47	PP	CURD	0.20	0.09	7.94 x 10^−6^	0.16	0.03	4.68 x 10^−12^	6.61 x 10^−17^
rs483916	8	9936091	MIR124-1			A/C	0.47	SBP	CURD	0.38	0.17	1.05 x 10^−9^	0.21	0.16	3.24 x 10^−11^	3.31 x 10^−20^
rs615632	8	9938811	MIR124-1			T/C	0.53	SBP	LHD	-0.50	-0.30	7.41 x 10^−9^	-0.40	0.09	1.07 x 10^−4^	3.63 x 10^−12^
rs9650622	8	9946782	LOC105379235	MIR124-1		T/G	0.53	DBP	CURD	-0.24	-0.01	4.07 x 10^−6^	-0.12	-0.07	1.10 x 10^−7^	4.27 x 10^−13^
rs56243511	8	9948185	LOC105379235	MIR124-1		T/C	0.47	SBP	CURD	0.37	0.11	2.57 x 10^−8^	0.27	0.14	1.91 x 10^−13^	1.74 x 10^−21^
rs656319	8	9956901	LOC105379235	MIR124-1		A/G	0.45	MAP	LHD	0.29	0.20	1.29 x 10^−6^	0.24	0.06	6.03 x 10^−5^	7.59 x 10^−11^
rs656319	8	9956901	LOC105379235	MIR124-1		A/G	0.45	SBP	LHD	0.39	0.35	8.71 x 10^−7^	0.43	0.01	1.62 x 10^−6^	1.59 x 10^−12^
rs11786677	8	10406750	MSRA		Intron	A/G	0.58	SBP	CURD	-0.25	-0.22	2.57 x 10^−7^	-0.40	0.03	1.35 x 10^−42^	5.62 x 10^−49^
rs2062331	8	10122482	MSRA		Intron	A/G	0.54	DBP	CURD	-0.18	-0.15	2.00 x 10^−8^	-0.18	0.00	7.59 x 10^−8^	5.01 x 10^−15^
rs11993089	8	10152442	MSRA		Intron	T/G	0.42	PP	CURD	0.24	0.05	5.25 x 10^−6^	0.32	-0.13	4.68 x 10^−18^	6.17 x 10^−23^
rs7832708	8	10332530	MSRA		Intron	T/C	0.49	SBP	LHD	0.55	0.07	2.19 x 10^−8^	0.42	-0.09	2.19 x 10^−5^	5.89 x 10^−13^
rs4841409	8	10658864	RP1L1			A/G	0.44	MAP	CURD	0.18	0.14	7.59 x 10^−7^	0.27	-0.12	9.77 x 10^−6^	5.13 x 10^−11^
rs4841409	8	10658864	RP1L1			A/G	0.44	MAP	LHD	0.37	-0.14	6.03 x 10^−6^	0.36	-0.19	2.14 x 10^−6^	6.46 x 10^−12^
rs4841409	8	10658864	RP1L1			A/G	0.44	SBP	CURD	0.23	0.25	1.91 x 10^−7^	0.32	0.12	9.55 x 10^−16^	4.90 x 10^−23^
rs10096777	8	10660990	RP1L1			A/G	0.56	SBP	LHD	-0.52	0.10	1.55 x 10^−6^	-0.60	0.39	2.88 x 10^−8^	3.80 x 10^−14^
rs7814795	8	10661775	MIR4286			T/C	0.55	MAP	CURD	-0.18	-0.14	7.59 x 10^−7^	-0.22	0.08	1.45 x 10^−4^	9.77 x 10^−10^
rs7814795	8	10661775	MIR4286			T/C	0.55	SBP	CURD	-0.22	-0.26	1.78 x 10^−7^	-0.2	-0.15	2.29 x 10^−14^	1.48 x 10^−21^
rs7814795	8	10661775	MIR4286			T/C	0.55	SBP	LHD	-0.50	0.06	2.04 x 10^−6^	-0.59	0.38	3.80 x 10^−8^	7.76 x 10^−14^

The most significantly associated SNVs are shown per gene for each Blood Pressure (BP) trait and alcohol status. Abbreviations: SNV, single nucleotide variant; Chr, chromosome; Position, Gene, and Role in dbSNP build 150 (hg38); Role: Intronic, Non-coding transcript (NCT) or intergenic (blank space) SNV; Near gene reflects genes at up to +/-500 kb and related to BP / alcohol; A1/2, Coded and non-coded alleles; Frq1, Frequency of coded allele; Trait, SBP: Systolic BP, DBP: Diastolic BP, MAP: Mean Arterial Pressure, PP: Pulse Pressure; Drink: Alcohol consumption, CURD, Current drinker (yes/no), LHD, Light(1–7 drinks/week) or heavy (≥8 drinks/week) drinker; Stage 1, Discovery cohorts; Stage 2, Replication cohorts; S1 & S2,Combined Discovery and Replication; b_M, beta coefficient of SNV; b_I: SNV*E is SNV-alcohol interaction effect; P-Value: modified-interaction METAL P-Value; P-Meta, modified-interaction METAL P-Value of Meta-analysis in combined Stage 1 and Stage 2.

**Table 2:** Novel SNVs/Genes associated with BP traits in European ancestry.

										Stage 1 (S1)			Stage 2 (S2)			S1 & S2
SNV	Chr	Position	Gene	Near Gene	Role	A1/2	Frq1	Trait	Drink	b_M	b_I	*P*-Value	b_M	b_I	*P*-Value	*P*-Meta
rs28680211	8	10661935	MIR4286			A/T	0.55	MAP	LHD	-0.36	0.13	7.76 x 10^−6^	-0.35	0.19	3.98 x 10^−6^	1.59 x 10^−11^
rs13276026	8	10752445	LOC102723313	SOX7	Intron	A/G	0.56	SBP	CURD	-0.23	-0.23	5.62 x 10^−7^	-0.26	-0.19	2.29 x 10^−15^	3.98 x 10^−22^
rs7814757	8	10817678	PINX1		Intron	T/C	0.40	SBP	CURD	0.24	0.22	7.94 x 10^−7^	0.21	0.26	8.71 x 10^−16^	2.63 x 10^−22^
rs4841465	8	10962344	XKR6		Intron	T/C	0.52	SBP	CURD	-0.21	-0.27	6.17 x 10^−7^	-0.21	-0.21	6.03 x 10^−14^	1.41 x 10^−20^
rs4841465	8	10962344	XKR6		Intron	T/C	0.52	SBP	LHD	-0.51	-0.10	3.89 x 10^−7^	-0.43	0.04	4.07 x 10^−6^	1.23 x 10^−12^
rs9969423	8	11398066	FAM167A-AS1	C8orf12	Intron	A/C	0.50	SBP	CURD	0.21	0.2	3.98 X 10^−6^	0.29	0.01	1.20 x 10^−7^	5.37 x 10^−13^
rs9969423	8	11398066	FAM167A-AS1	C8orf12	Intron	A/C	0.50	SBP	LHD	0.52	-0.09	4.90 X 10^−6^	0.38	-0.07	1.95 X 10^−4^	8.13 X 10^−10^
rs12156009	8	11427710	FAM167A	C8orf12	Intron	A/C	0.51	SBP	CURD	0.29	0.21	1.66 X 10^−7^	0.17	0.10	1.02 X 10^−5^	5.37 X 10^−12^
rs13255193	8	11451683	FAM167A	FAM167A	Intron	T/C	0.46	SBP	LHD	0.53	-0.11	6.76 X 10^−7^	0.36	-0.11	7.76 X 10^−4^	6.17 X 10^−10^
rs6983727	8	11558303	BLK		Intron	T/C	0.48	PP	CURD	-0.15	-0.15	4.68 X 10^−6^	-0.17	-0.08	1.66 X 10^−10^	5.89 X 10^−16^
rs6983727	8	11558303	BLK		Intron	T/C	0.48	PP	LHD	-0.24	-0.25	5.89 X 10^−6^	-0.26	0.07	6.03 X 10^−5^	1.74 X 10^−9^
rs6983727	8	11558303	BLK		Intron	T/C	0.48	SBP	LHD	-0.47	-0.17	4.27 X 10^−7^	-0.34	0.00	1.55 X 10^−4^	1 X 10^−10^
rs34190028	8	11559641	BLK		Intron	T/G	0.48	SBP	CURD	-0.16	-0.31	5.13 X 10^−7^	-0.36	-0.04	3.47 X 10^−13^	1.26 X 10^−19^
rs899366	8	11572976	LINC00208			A/G	0.33	MAP	CURD	0.15	0.18	3.39 X 10^−6^	0.28	0.00	3.47 X 10^−79^	1.51 X 10^−82^
rs7464263	8	11576667	LINC00208		NCT	A/T	0.48	SBP	LHD	0.48	0.24	6.03 X 10^−8^	0.41	-0.08	3.72 X 10^−5^	4.37 X 10^−12^
rs1478894	8	11591245	LINC00208			T/C	0.36	SBP	CURD	0.33	0.21	1.00 X 10^−8^	0.24	0.16	3.31 X 10^−11^	2.51 X 10^−19^
rs4841569	8	11594668	LINC00208			A/G	0.42	PP	CURD	-0.10	-0.28	1.95 X 10^−7^	-0.07	-0.18	1.23 X 10^−10^	4.17 X 10^−17^
rs4841569	8	11594668	LINC00208			A/G	0.42	PP	LHD	-0.27	-0.44	2.88 X 10^−8^	-0.28	0.08	2.40 X 10^−5^	4.79 X 10^−11^
rs17807624	8	11605506	LINC00208			T/C	0.35	DBP	CURD	0.11	0.20	5.37 X 10^−6^	0.14	0.05	8.13 X 10^−8^	6.03 X 10^−13^
rs17807624	8	11605506	LINC00208			T/C	0.35	MAP	LHD	0.45	-0.22	5.13 X 10^−7^	0.32	-0.16	6.03 X 10^−5^	2.57 X 10^−11^
rs13280442	8	11610048	LOC105379242	LINC00208		C/G	0.55	MAP	CURD	0.23	0.11	1.29 X 10^−6^	0.28	-0.17	4.90 X 10^−4^	1.62 X 10^−8^
rs13280442	8	11610048	LOC105379242	LINC00208		C/G	0.55	MAP	LHD	0.40	-0.11	3.39 X 10^−6^	0.28	-0.01	5.25 X 10^−5^	1.38 X 10^−10^
rs13280442	8	11610048	LOC105379242	LINC00208		C/G	0.55	SBP	CURD	0.30	0.24	8.32 X 10^−8^	0.48	-0.03	1.91 X 10^−16^	9.12 X 10^−24^
rs13280442	8	11610048	LOC105379242	LINC00208		C/G	0.55	SBP	LHD	0.57	0.10	1.38 X 10^−7^	0.50	-0.10	4.68 X 10^−7^	5.01 X 10^−14^
rs13250871	8	11610254	LOC105379242	LINC00208		A/G	0.4	PP	CURD	-0.10	-0.27	8.51 X 10^−7^	-0.21	-0.10	2.63 X 10^−17^	1.91 X 10^−23^
rs13250871	8	11610254	LOC105379242	LINC00208		A/G	0.39	PP	LHD	-0.24	-0.49	7.59 X 10^−8^	-0.29	0.10	2.69 X 10^−5^	2.14 X 10^−10^
rs36038176	8	11752486	GATA4		Intron	T/C	0.28	SBP	CURD	-0.21	-0.29	1.07 X 10^−6^	-0.39	0.15	3.89 X 10^−5^	3.24 X 10^−10^
rs55872725	16	53775211	FTO		Intron	T/C	0.41	SBP	CURD	0.69	-0.31	3.39 X 10^−9^	0.36	-0.16	2.14 X 10^−5^	2.40 X 10^−13^
rs7185735	16	53788739	FTO		Intron	A/G	0.59	PP	CURD	-0.36	0.07	6.31 X 10^−8^	-0.25	0.14	3.31 X 10^−4^	2.09 X 10^−10^

The most significantly associated SNVs are shown per gene for each Blood Pressure (BP) trait and alcohol status. Abbreviations: SNV, single nucleotide variant; Chr, chromosome; Position, Gene, and Role in dbSNP build 150 (hg38); Role: Intronic, Non-coding transcript (NCT) or intergenic (blank space) SNV; Near gene reflects genes at up to +/-500 kb and related to BP / alcohol; A1/2, Coded and non-coded alleles; Frq1, Frequency of coded allele; Trait, SBP: Systolic BP, DBP: Diastolic BP, MAP: Mean Arterial Pressure, PP: Pulse Pressure; Drink: Alcohol consumption, CURD, Current drinker (yes/no), LHD, Light(1–7 drinks/week) or heavy (≥8 drinks/week) drinker; Stage 1, Discovery cohorts; Stage 2, Replication cohorts; S1 & S2,Combined Discovery and Replication; b_M, beta coefficient of SNV; b_I: SNV*E is SNV-alcohol interaction effect; P-Value: modified-interaction METAL P-Value; P-Meta, modified-interaction METAL P-Value of Meta-analysis in combined Stage 1 and Stage 2.

**Table 3:** Potential novel SNVs/Genes associated with BP traits in African ancestry.

										Stage 1 (S1)			Stage 2 (S2)			S1 & S2
SNV	Chr	Position	Gene	Near Gene	Role	A1/2	Frq1	Trait	Drink	b_M	b_I	*P*-Value	b_M	b_I	*P*-Value	*P*-Meta
rs80158983	6	65489746	EYS	EYS	intron	T/C	0.02	SBP	CURD	3.53	-10.05	1.29 x 10^−8^	0.95	-3.08	8.32 x 10^−1^	6.92 x 10^−9^
rs76987554	6	133759717	TARID	MGC34034, SGK1	intron	T/C	0.09	SBP	CURD	-2.45	0.80	2.19 x 10^−8^	-1.48	-0.42	2.09 x 10^−1^	1.86 x 10^−9^
rs79505281	8	35841899	UNC5D			A/C	0.02	PP	CURD	-5.66	1.26	6.03 x 10^−7^	1.50	-6.67	2.82 x 10^−3^	3.24 x 10^−9^
rs115888294	8	94105161	CDH17			T/C	0.93	PP	CURD	-1.18	-0.55	1.59 x 10^−7^	-0.71	-0.84	2.19 x 10^−1^	1.29 x 10^−8^
rs73655199	9	98145201	CORO2A	GABBR2	intron	A/G	0.01	PP	CURD	-5.09	-0.13	3.16 x 10^−9^	-0.45	-2.71	2.95 x 10^−1^	1.41 x 10^−9^
rs4253197	10	49473111	ERCC6	CHAT	intron	A/G	0.89	PP	CURD	0.66	0.67	6.61 x 10^−7^	-0.80	2.57	3.63 x 10^−2^	4.90 x 10^−8^
rs11200509	10	122256927	TACC2			C/G	0.17	PP	LHD	-0.27	-4.05	6.76 x 10^−9^	1.72	-2.92	1.45 x 10^−1^	1.00 x 10^−8^
rs10741534	11	11233360	GALNT18			T/C	0.09	SBP	CURD	2.34	-3.76	8.32 x 10^−8^	0.94	-2.76	2.29 x 10^−1^	1.18 x 10^−8^
rs139077481	11	107579224	ELMOD1			T/C	0.99	PP	CURD	-3.18	10.41	1.32 x 10^−7^	-0.81	4.67	3.47 x 10^−1^	3.39 x 10^−8^
rs140520944	18	29508647	LOC105372045	MIR302F		T/G	0.02	PP	CURD	-0.49	-4.83	1 x 10^−12^	1.94	-3.30	6.03 x 10^−1^	4.07 x 10^−13^
rs142673685	19	31669942	LOC105372361	THEG5		T/C	0.01	PP	CURD	-3.04	-2.20	5.01 x 10^−8^	-2.92	2.29	4.47 x 10^−1^	3.63 x 10^−8^
										**Stage 1 (S1)**			**No Stage 2 (S2)**			
**SNV**	**Chr**	**Position**	**Gene**	**Near Gene**	**Role**	**A1/2**	**Frq1**	**Trait**	**Drink**	**b_M**	**b_I**	***P*-Value**				
rs9862344	3	178283140	LOC105374235	KCNMB2, KCNMB2-IT1		T/C	0.02	SBP	CURD	3.53	-10.05	1.29 x 10^−8^				
rs73884351	3	178287933	LOC105374235	KCNMB2, KCNMB2-IT1		T/C	0.09	SBP	CURD	-2.45	0.80	2.19 x 10^−8^				
rs145429126	4	47000363	GABRB1	GABRA4	intron	A/C	0.02	PP	CURD	-5.66	1.26	6.03 x 10^−7^				
rs61494734	9	29196976	LINGO2		intron	T/C	0.93	PP	CURD	-1.18	-0.55	1.59 x 10^−7^				
rs201383951	10	119468517	GRK5	BAG3		A/G	0.01	PP	CURD	-5.09	-0.13	3.16 x 10^−9^				
rs186331780	12	61317029	LOC105369793	FAM19A2		A/G	0.89	PP	CURD	0.66	0.67	6.61 x 10^−7^				
rs187888844	13	67705907	LOC105370250	PCDH9		C/G	0.17	PP	LHD	-0.27	-4.05	6.76 x 10^−9^				
rs116464496	13	105934773	LINC00343			T/C	0.09	SBP	CURD	2.34	-3.76	8.32 x 10^−8^				

The most significantly associated SNVs are shown per gene for each Blood Pressure (BP) trait and alcohol status. Abbreviations: SNV, single nucleotide variant; Chr, chromosome; Position, Gene, and Role in dbSNP build 150 (hg38); Role: Intronic or intergenic (blank space) SNV; Near gene reflects genes at up to +/-500 kb and related to BP / alcohol; A1/2, Coded and non-coded alleles; Frq1, Frequency of coded allele; Trait, SBP: Systolic BP, DBP: Diastolic BP, MAP: Mean Arterial Pressure, PP: Pulse Pressure; Drink: Alcohol consumption, CURD, Current drinker (yes/no), LHD, Light(1–7 drinks/week) or heavy (≥8 drinks/week) drinker; Stage 1, Discovery cohorts; Stage 2, Replication cohorts; S1 & S2,Combined Discovery and Replication; b_M, beta coefficient of SNV; b_I: SNV*E is SNV-alcohol interaction effect; P-Value: modified-interaction METAL P-Value; P-Meta, modified-interaction METAL P-Value of Meta-analysis in combined Stage 1 and Stage 2

**Table 4:** Novel SNVs/Genes associated with BP traits in Multi-ancestry meta-analysis in combined Stage 1 and Stage 2.

											Stage 1 and Stage 2			
SNV	Chr	Position	Gene	Near Gene	Role	A1/2	Frq1	Ancestry	Trait	Drink	b_M	b_I	*P*-Meta	N
rs10092965	8	8515975	LOC105379224	SGK223		A/G	0.53	EA, HA	DBP	CURD	-0.19	0.01	1.74 x 10^−12^	373,915
rs7823056	8	8525195	LOC105379224	SGK223		A/G	0.5	AA, EA	PP	LHD	-0.31	0.10	3.31 x 10^−11^	161,080
rs7823056	8	8525195	LOC105379224	SGK223		A/G	0.41	AA, EA	SBP	LHD	-0.44	0.11	1.38 x 10^−11^	214,814
rs453301	8	9172877	LOC102724880	PPP1R3B		T/G	0.5	EA, HA	DBP	CURD	-0.13	-0.07	4.90 x 10^−12^	365,537
rs10503387	8	9293015	LOC157273			T/C	0.37	AA, EA	SBP	CURD	0.32	0.03	1.07 x 10^−14^	381,431
rs11781008	8	9295729	LOC157273			T/G	0.37	EA, HA	DBP	CURD	0.13	0.07	1.05 x 10^−11^	373,915
rs4383974	8	9761838	TNKS		intron	C/G	0.7	AA, EA	SBP	CURD	-0.28	-0.08	2.04 x 10^−13^	381,431
rs9286060	8	9795635	TNKS			A/C	0.38	AA, EA	DBP	CURD	0.21	-0.02	2.29 x 10^−13^	371,053
rs34919878	8	10241994	MSRA		intron	A/G	0.41	EA, HA	DBP	CURD	-0.18	-0.05	5.75 x 10^−17^	365,537
rs4841294	8	10247558	MSRA		intron	A/C	0.43	AA, EA	SBP	LHD	-0.40	0.01	2.69 x 10^−10^	166,956
rs17693945	8	10248500	MSRA		intron	T/C	0.41	AA, EA	MAP	LHD	-0.30	0.08	1.51 x 10^−9^	166,054
rs13276026	8	10752445	LOC102723313	PINX1	intron	A/G	0.55	EA, HA	DBP	CURD	-0.11	-0.10	4.47 x 10^−14^	373,915
rs13276026	8	10752445	LOC102723313	PINX1	intron	A/G	0.55	EA, HA	MAP	CURD	-0.15	-0.03	4.68 x 10^−9^	373,911
rs13276026	8	10752445	LOC102723313	PINX1	intron	A/G	0.55	EA, HA	SBP	CURD	-0.22	-0.24	3.89 x 10^−23^	373,919
rs4551304	8	10807559	PINX1		intron	A/G	0.4	EA, HA	DBP	CURD	0.10	0.12	1.70 x 10^−14^	373,915
rs4551304	8	10807559	PINX1		intron	A/G	0.4	EA, HA	MAP	CURD	0.15	0.03	2.24 x 10^−8^	373,911
rs9969436	8	10985149	XKR6		intron	T/G	0.47	AA, EA	MAP	LHD	0.28	-0.01	3.09 x 10^−9^	165,894
rs2409784	8	11539347	BLK		intron	A/C	0.51	EA, HA	DBP	CURD	-0.11	-0.09	5.62 x 10^−12^	374,975
rs2244894	8	11591150	LINC00208			C/G	0.44	ASA, EA	PP	CURD	-0.07	-0.19	3.24 x 10^−15^	493,402
rs13249843	8	11601509	LINC00208			T/G	0.33	EA, HA	DBP	CURD	0.18	0.04	2.51 x 10^−15^	398,330
rs3735814	8	11749887	GATA4		intron	A/G	0.52	EA, HA	SBP	CURD	0.09	0.22	2.14 x 10^−10^	373,919
rs9928094	16	53765993	FTO		intron	A/G	0.63	ASA, EA	PP	CURD	-0.33	0.19	2.63 x 10^−15^	499,179
rs62033406	16	53790314	FTO		intron	A/G	0.55	ASA, EA	MAP	CURD	-0.22	0.12	3.31 x 10^−8^	511,074

The most significantly associated SNVs are shown per gene for each Blood Pressure (BP) trait and alcohol status. Abbreviations: SNV, single nucleotide variant; Chr, chromosome; Position, Gene, and Role, in dbSNP build 150 (hg38) annotation; Role: Intronic or intergenic (blank space) SNV; Near gene reflects genes at up to +/-500 kb and related to BP / alcohol; A1/2, Coded and non-coded alleles; Frq1, Frequency of coded allele; Trait, SBP: Systolic BP, DBP: Diastolic BP, MAP: Mean Arterial Pressure, PP: Pulse Pressure; Drink: Alcohol consumption, CURD, Current drinker (yes/no), LHD, Light(1–7 drinks/week) or heavy (≥8 drinks/week) drinker; Stage 1 and Stage 2, Combined Discovery and Replication; b_M, beta coefficient of SNV; b_I: SNV*E is SNV-alcohol interaction effect; P-Meta, modified-interaction METAL P-Value of Meta-analysis in combined Stage 1 and Stage 2; N, Number of individuals.

Finally, we leveraged the added power of correlated meta-analysis[25, 26] for BP traits to detect additional variants. We performed correlated meta-analysis on *P*-values from METAL-meta-analysis[24] of DBP, SBP, MAP and PP traits separately for current drinkers and light/heavy drinkers in Stage 1 European ancestry cohorts. A variant was considered pleiotropic if the *P*- METAL-meta reached *P* ≤ 0.0001 in two or more BP traits and the correlated meta-analysis *P*-value was *P* ≤ 5.0 x 10^−8^[27]. We identified eight novel BP loci (11 genes, Table 5), the above five novel loci (14 genes, Tables 1 and 2), and the 22 previously reported BP loci (49 genes).

**Table 5:** Novel SNVs/Genes associated with BP traits from correlated meta-analysis in European ancestry in Stage 1.

**Associations NOT Present in Tables 1 and 2, in Current Drinkers**												
**SNV**	**Chr**	**Position**	**Gene**	**Near Gene**	**Role**	**Frq1**	***P*-Correlated Meta**	***P*-DBP**	***P*-SBP**	***P*-MAP**	***P*-PP**	**N**
rs200124401	1	83336112	LOC107985037	TTLL7	intron	0.70	4.29 x 10^−8^	1.82 x 10^−5^	1.86 x 10^−6^	1.20 x 10^−6^	4.68 x 10^−4^	89,035
rs3813963	1	206648224	DYRK3	DYRK3, IL10	Synon	0.99	2.95 x 10^−8^	1.66 x 10^−4^	8.32 x 10^−8^	8.13 x 10^−7^	3.72 x 10^−4^	39,497
rs80169249	1	206683281	LOC105372875	MAPKAPK2		0.99	3.52 x 10^−8^	2.45 x 10^−4^	7.41 x 10^−8^	1.00 x 10^−6^	3.39 x 10^−4^	39,497
rs185597356	4	161336738	FSTL5	FSTL5		0.99	1.77 x 10^−8^	7.24 x 10^−7^	8.71 x 10^−7^	4.37 x 10^−8^	1.00 x 10^−2^	55,056
rs77779142	11	65832185	SNX32	SNX32		0.84	3.89 x 10^−8^	8.32 x 10^−5^	1.12 x 10^−6^	2.88 x 10^−6^	7.08 x 10^−5^	90,689
rs11227333	11	65874946	EFEMP2	EFEMP2		0.80	2.34 x 10^−8^	3.24 x 10^−5^	5.89 x 10^−7^	1.15 x 10^−6^	2.00 x 10^−4^	86,262
rs201407003	11	65894964	FOSL1	FOSL1, MALAT1	intron	0.85	1.76 x 10^−8^	2.09 x 10^−5^	6.31 x 10^−7^	7.94 x 10^−7^	2.04 x 10^−4^	86,262
**Associations Present in Tables 1 and 2, in Current Drinkers**												
**SNV**	**Chr**	**Position**	**Gene**	**Near Gene**	**Role**	**Frq1**	***P*-Correlated Meta**	***P*-DBP**	***P*-SBP**	***P*-MAP**	***P*-PP**	**N**
rs2980755	8	8506173	LOC107986913	SGK223		0.55	4.59 x 10^−9^	5.13 x 10^−4^	4.27 x 10^−8^	1.74 x 10^−6^	1.15 x 10^−6^	90,691
rs13270194	8	8520592	LOC105379224	CLDN23		0.51	1.59 x 10^−9^	2.14 x 10^−4^	2.45 x 10^−8^	8.13 x 10^−7^	8.51 x 10^−7^	90,691
rs1976671	8	9822124	TNKS	TNKS		0.62	2.01 x 10^−9^	1.58 x 10^−6^	4.68 x 10^−8^	3.02 x 10^−8^	1.26 x 10^−3^	90,691
rs483916	8	9936091	MIR124-1	MIR124-1		0.47	1.55 x 10^−11^	1.17 x 10^−6^	1.05 x 10^−9^	3.55 x 10^−9^	7.94 x 10^−6^	90,691
rs2062331	8	10122482	MSRA	MSRA	intron	0.54	5.49 x 10^−13^	2.00 x 10^−8^	1.70 x 10^−10^	1.20 x 10^−10^	1.32 x 10^−5^	90,691
rs10096777	8	10660990	RP1L1	RP1L1		0.44	7.58 x 10^−9^	9.77 x 10^−5^	1.91 x 10^−7^	9.55 x 10^−7^	1.51 x 10^−5^	90,691
rs7814795	8	10661775	MIR4286	MIR4286		0.45	6.86 x 10^−9^	7.76 x 10^−5^	1.78 x 10^−7^	7.59 x 10^−7^	2.00 x 10^−5^	90,691
rs13276026	8	10752445	LOC102723313	SOX7	intron	0.44	4.79 x 10^−8^	1.38 x 10^−4^	5.62 x 10^−7^	1.58 x 10^−6^	1.91 x 10^−4^	90,691
rs12156009	8	11427710	FAM167A	FAM167A	intron	0.51	9.49 x 10^−9^	1.82 x 10^−4^	1.66 x 10^−7^	1.32 x 10^−6^	1.07 x 10^−5^	90,691
rs1478894	8	11591245	LINC00208	LINC00208		0.64	3.69 x 10^−10^	1.66 x 10^−5^	1.00 x 10^−8^	8.51 x 10^−8^	8.32 x 10^−6^	90,691
rs13280442	8	11610048	LOC105379242	GATA4		0.45	5.23 x 10^−9^	1.86 x 10^−4^	8.32 x 10^−8^	1.29 x 10^−6^	4.47 x 10^−6^	90,691
rs9937521	16	53765384	FTO	FTO	intron	0.61	2.89 x 10^−10^	8.13 x 10^−5^	4.68 x 10^−9^	6.46 x 10^−7^	2.04 x 10^−7^	90,691
**Associations NOT Present in Tables 1 and 2, in Light / Heavy Drinkers**												
**SNV**	**Chr**	**Position**	**Gene**	**Near Gene**	**Role**	**Frq1**	***P*-Correlated Meta**	***P*-DBP**	***P*-SBP**	***P*-MAP**	***P*-PP**	**N**
rs117519896	15	43645473	CATSPER2	CATSPER2	intron	0.98	8.25 x 10^−9^	7.76 x 10^−5^	2.88 x 10^−7^	9.77 x 10^−7^	2.75 x 10^−5^	13,141
rs2957398	17	53625691	LOC107984982	LOC107984982		0.29	1.11 x 10^−8^	8.91 x 10^−5^	1.23 x 10^−7^	2.69 x 10^−6^	3.80 x 10^−5^	54,785
rs146091319	18	71962177	LOC102725148	LOC102725148		0.99	1.50 x 10^−8^	1.26 x 10^−3^	1.74 x 10^−8^	3.39 x 10^−6^	1.26 x 10^−5^	26,187
rs111700101	19	11433340	CCDC151	CCDC151	intron	0.94	2.78 x 10^−8^	3.80 x 10^−6^	8.13 x 10^−7^	3.80 x 10^−7^	3.55 x 10^−3^	37,996
**Associations Present in Tables 1 and 2, in Light / Heavy Drinkers**												
**SNV**	**Chr**	**Position**	**Gene**	**Near Gene**	**Role**	**Frq1**	***P*-Correlated Meta**	***P*-DBP**	***P*-SBP**	***P*-MAP**	***P*-PP**	**N**
rs34062996	8	9802688	TNKS	TNKS		0.39	2.26 x 10^−9^	6.17 x 10^−5^	2.40 x 10^−8^	3.24 x 10^−7^	3.47 x 10^−5^	54,785
rs615632	8	9938811	MIR124-1	MIR124-1		0.47	4.18 x 10^−10^	1.78 x 10^−5^	7.41 x 10^−9^	8.13 x 10^−8^	2.34 x 10^−5^	54,785
rs7843924	8	10119030	MSRA	MSRA	intron	0.54	2.46 x 10^−13^	1.38 x 10^−8^	1.58 x 10^−10^	1.58 x 10^−10^	6.46 x 10^−6^	54,785
rs11250099	8	10961147	XKR6	XKR6	intron	0.48	4.13 x 10^−8^	1.82 x 10^−4^	3.98 x 10^−7^	2.19 x 10^−6^	1.62 x 10^−4^	54,785
rs13255193	8	11451683	FAM167A	FAM167A	intron	0.46	2.41 x 10^−8^	7.76 x 10^−5^	6.76 x 10^−7^	1.66 x 10^−6^	9.77 x 10^−5^	54,785
rs4841559	8	11559376	BLK	BLK	intron	0.51	4.12 x 10^−8^	4.79 x 10^−4^	4.47 x 10^−7^	9.55 x 10^−6^	1.35 x 10^−5^	54,785
rs4840573	8	11605721	LINC00208	LINC00208		0.60	3.94 x 10^−9^	1.15 x 10^−3^	7.76 x 10^−8^	7.59 x 10^−6^	4.57 x 10^−8^	53,371
rs13280442	8	11610048	LOC105379242	GATA4		0.45	6.26 x 10^−9^	2.40 x 10^−4^	1.38 x 10^−7^	3.39 x 10^−6^	2.24 x 10^−6^	54,785

The most significantly associated SNVs are shown per gene for correlated BP traits and alcohol status: Current drinker (yes/no), and Light (1–7 drinks/week) or heavy (≥8 drinks/ week) drinker. The “NOT Present in Tables 1 and 2” represents the associations detected using correlated meta-approach, otherwise the associations were already presented in Tables 1 and 2 using modified-interaction METAL approach. Abbreviations: SNV, single nucleotide variant; Chr, chromosome; Position, Gene, and Role in dbSNP build 150 (hg38); Role: Intronic, synonymous codon (Synon), or intergenic (blank space) SNV; Near gene reflects genes at up to +/-500 kb and related to BP / alcohol; Frq1, Frequency of coded allele; P-Correlated Meta, P-Value of BP-correlated meta-analysis; P-DBP, modified-interaction METAL P-Value for Diastolic BP; P-SBP, modified-interaction METAL P-Value for Systolic BP; P-MAP, modified-interaction METAL P-Value for Mean Arterial Pressure; P-PP, modified-interaction METAL P-Value for Pulse Pressure; N, Number of individuals.

### Gene transcription regulation

HaploReg[28, 29], RegulomeDB[30, 31], GTEx[32], GWAS3D[33], and GRASP[34] provided evidence that several SNVs on 8p23.1 have regulatory features (S13 and S14 Tables). From the analyses with GTEx, a total of 227 (56 novel and 171 BP-known S14 Tables) SNVs had tissue specific eQTL results. Seven out of 56 novel SNVs were associated with eQTLs that have expression in brain, thyroid, and/or blood. From 171 BP-known SNVs, 44 were significantly associated with eQTLs with expression in adipose, artery, esophagus, lung, pancreas, thyroid and/or fibroblasts. In addition, GWAS3D analyses suggested trans-regulation features for our BP candidate SNVs. It identified 215 SNVs with long-range interactions.

### BP genes show enrichment for alcohol and cardiovascular disease

We used GeneGO[35] and Literature Lab[36] to perform enrichment analyses for the full set of novel and reported (179 BP candidate) genes identified from our analyses. Literature Lab, based on 106,967 abstracts for “*Drinking”* Physiology from MeSH (Medical Subject Headings), identified enrichment (*P* < 0.00001) related to *ALDH2* (known to be associated with alcohol dependence)[15] and several other genes, including our novel finding for *ERCC6*, *CATSPER2*, *GABRB1* and *GATA4*. The main contributor for “*Angiotensin II”* (*P* < 0.00001) was *AGT* and *ACE* for “*Hypertension”* (*P* = 0.0002). *AGT* and *ACE* are part of *Renin-Angiotensin System* pathway (*KEGG*, *map04614*), involved in BP homeostasis, fluid-electrolyte balance, and essential hypertension[37, 38].

Our results were significantly enriched for cardiovascular disease-related biological functions. For example, “Cardiovascular Diseases” (*P* = 0.0034) enriched with genes *AGT*, *NPPA*, *ACE*, *NOS3*, *ADRB1*, *MTHFR*, *FBN1* and *GATA4*. “Heart Failure” (*P* = 0.0003) and “Cardiomegaly” (*P* = 0.0003); from Pathological Conditions: “Hypertrophy” (*P* = 0.0001); from Anatomy MeSH: “Heart” (*P* = 0.0001), “Cardiovascular System” (*P* = 0.0002) and “Aorta” (*P* = 0.0002); and from domain Tissue Type MeSH: “Myocardium” (*P* = 0.0008) enriched with *NPPA*, *GATA4*, *AGT*, *ADRB1*, *NOS3*, *ACE* and *KCNJ11*. GeneGO identified an additional term “Cardiac Arrhythmias” (*P-FDR* = 3.2 x 10^−20^).

### Protein-protein interactions and pathways enriched for BP genes

The protein-protein interactions (PPI) analyses showed that several novel gene proteins are important hubs in interaction with many other proteins. For example, *MAPKAPK2* (1q32.1, Table 5) interacts among others with *BAG2*, *LISP1* and *ELAVL1*. *ELAVL1* interacts also with novel *XKR6* from 8p23.1 (S16 Fig). Of the novel genes *GRK5*, *MAPKAPK2*, *BLK*, *EFEMP2* and *ERCC6* ranked the highest in protein-protein interconnectivity (degree), while *MAPKAPK2*, *PINX1*, *EFEMP2*, *FAM167A* and *GRK5* were ranked the highest for important interconnections based on PageRank algorithm. Further, we entered the gene labels of the combined PPI network into the GeneGo software and found enrichment for *Cytoskeleton Remodeling/TGF/ Wnt* (*P*-FDR = 1.7 x 10^−17^), among other pathways.

## Discussion

This is the first large-scale study to systematically evaluate the role of joint effect of main gene and gene-alcohol interaction on BP in a very large meta-analysis across multiple ancestries.

### BP genes interacting with alcohol show association with alcohol metabolism or dependence

**Fig 1:**
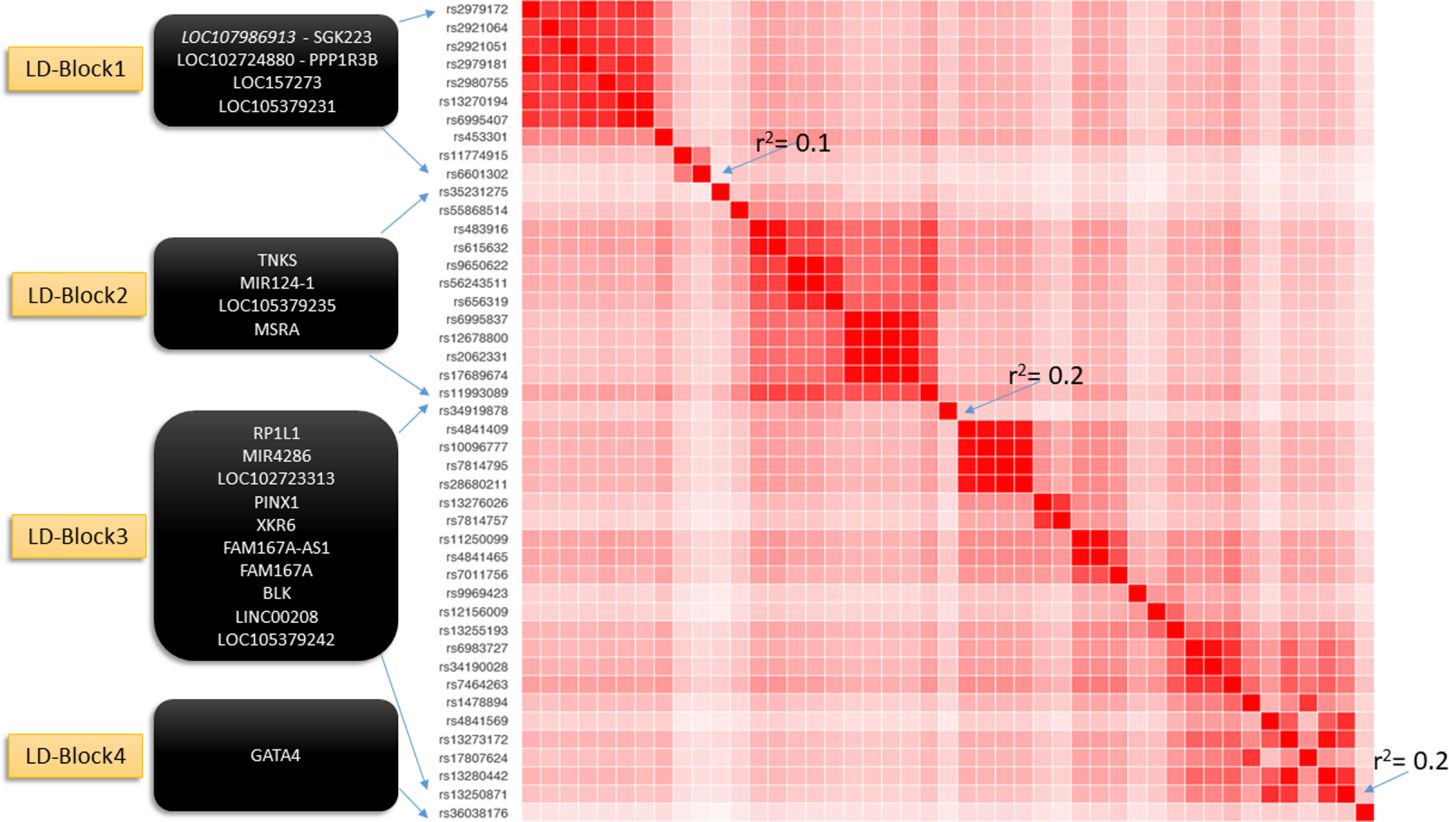
Identification of four independent LD blocks in the 8p23.1 region *(~3*.*3 MBs*).

The 8p23.1 containing novel BP associations spans ~3.3 Mb from *LOC107986913-SGK223* (8,452,998 bp) to *GATA4* (11,752,486 bp) (Tables 1 and 2). Chromosome 8p23.1 is a complex region of deletions and replications, with repeated inverse structures[39, 40]. We identified four LD blocks in 8p23.1 (Fig 1). The significant GWAS results on 8p23.1 are from European ancestry participants in Stage 1, Stage 2 follow up, and combined Stage 1 and Stage 2 meta-analyses. For this region, the evidence of genetic associations was identified from all four BP traits at both current drinking and light/heavy drinking status (Tables 1 and 2). The association on 8p23.1 found in the large European ancestry sample may also occur in other ancestries. The genome-wide significance levels in meta-analysis of European ancestry combined with African (5 genes), Asian (2 genes), and/or Hispanic (9 genes) ancestries have shown small improvements in their *P*-values compared to European ancestry meta-analysis alone (Tables 4 and S9). For some of these associated SNVs on 8p23.1, the allele frequencies in European ancestry are higher than in African ancestry (e.g., rs4841294: 0.44 versus 0.25, respectively), and Hispanic Ancestry (e.g., rs34919878: 0.42 versus 0.25, respectively). These findings suggest the presence of cross-population association patterns between European, African, and Hispanic ancestries, although they are not genome-wide significant in African and Hispanic ancestries presumably because of small sample sizes.

Several of the genes residing on 8p23.1 have been reported for alcohol metabolism and/or dependence. Overexpression of *PINX1* was reported to be associated with alcohol-related cirrhosis and fibrosis[41]. The transcription factor *GATA4* has been reported to be associated with alcohol dependence in several studies[42–45]. *GATA4* was suggested to regulate atrial natriuretic peptide (*ANP*, officially known as *NPPA*) modulating the amygdala’s response to alcohol dependence[39] and is associated with BP[46]. In addition, a suggestive GWAS finding was observed between a variant near *BLK-LINC00208* with alcohol dependence[47]. The S2 Note provides a comprehensive summary of novel and neighboring genes and their potential biological relevance.

*FTO* (16q12.2) variants in interaction with alcohol consumption were significant for BP in European ancestry (Table 2) and in combined meta-analysis of European and Asian ancestries (Table 4). *FTO* is involved in the regulation of thermogenesis and the control of adipocyte differentiation into brown or white fat cells[48]. *FTO* variants have been associated in diverse ancestries with obesity-related traits[49, 50], as well as alcohol consumption and alcohol dependency[51, 52]. Frequency of alcohol consumption was suggested to modify the effect of *FTO* variants on body mass index[53].

*IL10* (interleukin 10, ~49 Kb upstream of rs3813963, Table 5) has been associated with hypertension[54] and with alcoholic cirrhosis[55]. *MALAT1* (ncRNA, ~390 Kb upstream of rs201407003) is upregulated in the cerebellum, hippocampus and brain stem of alcoholics[56], which may represent an important mechanism for alcohol actions in the central nervous system.

It is worth to note that the allele frequencies for several potential SNVs in African ancestry (Table 3) are low (<0.10) but they are monomorphic in Europeans, which may suggest African-specific associations. Even though we did not have true replications for African ancestry associations (some of them due to missing SNVs or very low sample size in Stage 2), the identified candidate loci include genes previously related to alcohol consumption and dependence (Table 3). *GABRB1*[57] (4p12) and *GABBR2*[58] (9q22.33, 143 kb upstream of rs73655199) are major neurotransmitters in the vertebrate brain, representing ligand-gated ion channels and have been shown to associate with alcohol dependence. *EYS* (6q12) displayed association with alcohol dependence in multi-ancestry population studies for rare[59] and common[60] variants. *LINGO2* (9p21.1) was reported to be associated with age at onset of alcohol dependence in the Collaborative Study on the Genetics of Alcoholism[16]. *ERCC6* (10q11.23) participates in DNA repair in response to oxidative stress[61]. Carriers of Arg1230Pro at *ERCC6* had a decreased risk for laryngeal cancer, strongest in heavy smokers and high alcohol consumers[62]. *CHAT* (10q11.23, 136 kb downstream of rs4253197) encodes an enzyme that catalyzes the biosynthesis of the neurotransmitter acetylcholine, and binge ethanol in adolescents was reported to decrease *CHAT* expression[63]. *BAG3* (10q26.11, 183 Kb downstream of rs201383951) was also suggested to contribute to alcohol-induced neurodegenerations[64]. A mouse study suggested that *BAG3* exerts a vaso-relaxing effect through the activation of the PI3K/Akt/eNOS signaling pathway, and may influence BP regulation[64]. A GWAS identified association of *BAG3* with dilated cardiomyopathy[65], and suggestive association with alcohol dependence[44]. *SGK1* (409 kb upstream of rs76987554) is associated with increased BP[66] and may contribute to the mechanisms underlying behavioral response to chronic ethanol exposure[67]. In addition, our two potential genes by alcohol interaction, *TARID* (rs76987554) and *CDH17* (rs115888294), have been recently reported association with BP in African ancestry, which supports our findings[68].

### Regulatory features of BP genes

Analysis of our significant BP variants for cis- transcription regulation via HaploReg[29] (S13 Table) showed that in total about 11% of variants were localized in promoter histone marks, 55% in enhancer histone marks, 34% at DNAse hypersensitive sites, 10% located at protein regulatory binding sites, and 88% were predicted to change regulatory protein binding motifs. These feature findings are inflated, because several variants are in LD blocks. Several of our variants had *P*-values ≤ 5.0 x 10^−8^ for being eQTLs for one or more target genes. The rs2921053 is the best eSNV regulating the transcription of *SGK223* in thyroid tissue (*P*-value = 1.04 x 10^−67^). Thyroid hormones are known to affect BP, heart and cardiovascular system[69].

### Pathways enriched for BP genes

Our findings, *TNKS* (Table 1), *FSTL5* and *MAPKAPK2* (Table 5) and many other genes from PPI networks (S17 Fig), are part of *Wnt/beta-catenin*[70] signaling pathway. The *TNKS* forms a complex for degrading β-catenin (*CTNNB1*)[70] in interaction with *AXIN1*, *AXIN*2, and glycogen synthase kinase 3β (*GSK-3β*) (S17 and S18 Figs). The *Wnt/beta-catenin* pathway is known to be involved in renal injury and fibrosis induced by hypertension[71]. In addition, *TNKS* is involved in the regulation of *GLUT4* trafficking in adipocytes[72]. Other findings from correlated meta-analysis also contributed to pathways. For example, rs206648224 is intronic to *DYRK3*, 37 Kb upstream of *MAPKAPK2*, and 119 Kb downstream of *IL10*. *MAPKAPK2* is a stress-activated serine/threonine-protein kinase involved in cytokine production especially for *TNF* and *IL6*, and phosphorylates among others *LSP1*, already identified in association with BP[9]. *MAPKAPK2*[73] augments and *FSTL5*[74] diminishes the expression of Wnt/β-catenin signaling pathway.

### Limitations

Despite large sample sizes in Stages 1 and 2 (≈131K individuals and ≈440K individuals, respectively), our novel variants (8p23 and 16q12) are common in their allele frequencies. For an analysis of gene by alcohol interactions in BP, even larger sample sizes are required to have sufficient power for detecting (and replicating) variants with lower allele frequency in the genome.

Our findings were based on a joint test of the main and interaction effects, which limits our ability to statistically differentiate the effect of interaction from the main effect. However, there is evidence that several of our novel and previously reported findings suggest association with alcohol consumption and dependency.

For African ancestry, the findings were not replicated, due to low sample size in Stage 2 (≈3K individuals) versus Stage 1 (≈21K individuals) and because seven potential variants for African ancestry were not available in Stage 2.

There are fewer associations of SNVs interacting with light/heavy drinkers compared to current drinkers, which is probably due to the reduced sample size in light/heavy drinkers. We also found an association in light/heavy drinkers which is not present in current drinkers. The *LOC105374235* gene interacts with light/heavy drinkers for SBP but does not interact with current drinkers for SBP in African ancestry (Table 3 and S10 Fig). These findings suggest that novel loci for BP can be expected to be discovered when increasing the sample size for light/heavy drinkers.

The two Brazilian cohorts (from discovery only) were included in the multi-ancestry meta-analyses. However, their association results did not contribute to SNV-alcohol interactions for BP traits, which could be in part to the relative small sample size (4,415 subjects) affecting the power of associations in the joint gene-environmental interaction model.

### Conclusion

We identified and replicated five novel loci (380 SNVs in 21 genes) via joint test of main genetic effect and gene-alcohol interaction, and eight novel loci (11 genes) using correlated meta-analysis in European ancestry. We also found 18 potentially novel BP loci in discovery (*P* ≤ 5.0 x 10^−8^) in gene-alcohol interaction model in African ancestry participants, but without replication. In addition, we identified 49 loci previously reported for BP (2,159 SNVs in 109 genes) using the joint test for interaction in European and multi-ancestries meta-analyses. Several of these SNVs/genes are related to alcohol metabolism and dependence, have evidence for regulatory features, and are enriched in pathways for cardiovascular disease, hypertension and blood pressure homeostasis. Our findings provide novel insights into mechanisms of BP regulation and may highlight new therapeutic targets.

## Methods

Individuals between the ages of 18–80, who participated in the studies, provided written informed consent and approval by their research ethics committees and/or institutional review boards. The description of each participating study cohort is shown in S1 Note.

### Phenotypes, alcohol consumption, and study cohorts

SBP (in mmHg) and diastolic BP (DBP in mmHg) were measured at resting or sitting positions by averaging up to three BP readings at the same clinical visit. To account for the reduction in BP levels due to anti-hypertensive medication use, the BP levels were adjusted by adding 15 mm Hg to SBP and 10 mm Hg to DBP values. After adjustment, mean arterial pressure (MAP) was defined as the sum of two-thirds of DBP and one-third of SBP, and pulse pressure (PP) was estimated as the difference between SBP and DBP. Hypertension was defined whether participants presented: (i) SBP ≥ 140 mm Hg, (ii) DBP ≥ 90 mm Hg, and/or (iii) taking anti-hypertensive medication. For quality control (QC), SE-N (*i*.*e*., inverse of the median standard error versus the square root of the sample size) plots were produced[75]. If cohort-specific analytical problems existed, they were corrected.

Definition of “a dose or a drink” is about 17.7 grams of ethanol, which is the amount of a typical beverage of 12 oz. (354.882 ml) bottle or can of beer, a 5 oz. (147.868 ml) glass of wine, or a standard 1.5 oz. (44.3603 ml) shot of 80-proof spirits, such as gin, vodka, or whiskey[76]. Alcohol consumption was defined by two categories: (I) as current drinking (yes/no), and (II) in the subset of drinkers, as light/heavy drinking (1–7 drinks/week or ≥8 drinks/week).

### Genotyping

Genotyping was performed using Illumina (San Diego, CA, USA) or Affymetrix (Santa Clara, CA, USA) arrays. 1000 Genomes Imputation was implemented using MACH and Minimac, IMPUTE2, and/or BEAGLE software, based on the cosmopolitan panel from Phase I Integrated Release Version 3 Haplotypes (2010–11 data freeze, 2012-03-14 haplotypes). Dosages from 1000 Genomes were used in 106 cohorts out of 115 Stage 1 and Stage 2 cohorts. If 1000 Genomes were not available in a cohort, dosages based on HapMap Phase II / III reference panel (2 Stage 1 cohorts and 4 Stage 2 cohorts) or genotyped data (3 Stage 2 cohorts) were used in the analyses. Information of study characteristics, genotyping, imputation, covariates, and analyses are summarized for Stage 1 in S1–S4 Tables, and for Stage 2 in S5–S8 Tables.

### Interaction association analysis

Each Stage 1 and Stage 2 cohort conducted a joint statistical model analysis[24]:

$$E\left(Y\right)=b_{0}+b_{G}SNV+\;b_{E}E+\;b_{GE}SNV*E+b_{C}C,$$

where *SNV* is the dosage of the genetic (*G*) variant, *E* is the alcohol consumption (current drinker or light/heavy drinker) effect, SNV*E is SNV-alcohol interaction effect, *b* values are the respective beta coefficients from regression analysis and *C* represents covariates (age, sex, principal components (PCs), and other study-specific covariates). The joint model provides estimates of *b*_*G*_ and *b*_*GE*_, robust estimates of the corresponding standard errors (SEs) and covariance, and *P*-values from the joint 2 degree-of-freedom Wald test. The *SNV* effect (*b*_*G*_) is context-dependent and thus should not be interpreted as the “main effect”[23]. Principal components were derived from genotyped SNVs and used for controlling population stratification and genomic confounding effects. Each cohort decided the number of PCs to be included in the joint statistical model analysis, as shown in S4 Table (Discovery, in Stage 1) and S8 Table (Replication, in Stage 2). Particularly for African ancestry, it was required to include at the least the first PC and additional PCs as appropriate.

The association analyses were implemented by programming in R or using ProbABEL[77] for studies of unrelated individuals, or by GenABEL/MixABEL[78] or MMAP (O’Connell, unpublished; personal communication), which account for family relatedness.

### Meta-analysis and quality control

We employed a modified METAL software[24] to perform 2 degrees of freedom joint meta-analysis, using the inverse-variance weighted fixed-effects approach. We applied multiple steps of QC, both at cohort association analysis and at meta-analysis level, implemented with EasyQC, an R package[75]. They included filtering of markers with imputation quality < 0.5; with minor allele frequency < 1%; minor allele count ≤ 10; if alleles were mismatched when comparing the cohort’s alleles with the 1000 Genomes cosmopolitan panel; and/or if the allele frequencies were different from those of the 1000 Genomes. In addition, a cohort participated in the meta-analysis if it had more than 50 individuals consuming alcohol. The meta-analysis results were reported if they had more than 5,000 individuals and if at least two studies for each SNV contributed to the analysis. Markers with meta-heterogeneity *P* < 1.0 x 10^−6^ were dropped. We used (double) study- and meta- level genomic control corrections to account for population stratification accumulated across studies or due to unaccounted relatedness. Distributions of–log_10_ *P*-values of observed versus–log_10_ *P*-values expected (QQ plots) are shown in S2 and S3 Figs.

### Correlated meta-analysis

The genome (millions of SNPs) are under the null hypothesis of no genotype-phenotype association, which is only mildly contaminated with a relatively smaller set of SNVs that are under the alternative. The correlated meta-analysis[25, 26] performs a large sampling of genome and produces the polychoric correlation estimator (using SAS PROC FREQ). The estimator measures the relation degree of any non-independence between scans. The correlated meta-analysis corrects the inference for it, retaining the proper type I error structure. The correlated meta-analysis[25, 26] uses the Fisher’s 1925 method by combining *P*-values at each location of the genome. This technique uses the fact that for number of scans, sum of −2 ln (*p*_*i*_), approximately chi-square (*X*^2^) with two degrees of freedom. In the case of correlated GWAS, this sum is no longer distributed as a simple *X*^2^. Instead, the correlated meta-analysis method[25, 26] uses an inverse-normal transform, *Z*_i_ = *θ*^−1^ (*p*_*i*_) forming the N dimensional vector Z of all *Z*_i_ s. Then, the method applies the basic theorem of multidimensional statistics for the matrix D, if *Z~N*(*O*, *E*) then *DZ~N*(*O*, *E*∑*D*’). In particular, when *D* is a 1×*N* vector of all 1’s, *SUM*(*Z*) = *D Z ~ N*(0, *SUM*(∑)), whose tail probability gives the *Z* meta-analysis *P*-value. In this case, for estimating ∑, the SNV *P*-values are dichotomized across the genome as (*P* ≤ 0.5; *P* > 0.5). The software was developed in SAS.

### Bioinformatics analyses

The annotation of variants was sourced from NCBI dbSNP build 138 (hg19) during the analyses and updated to dbSNP build 150 (hg38) for reporting results. Our candidate SNVs for BP were questioned if they resided in any of regulatory marks, analyzing information from the NCBI Entrez gene, dbSNP, Encyclopedia of DNA Elements Consortium (ENCODE) project and the Roadmap Epigenomics Mapping Consortium (ROADMAP), as summarized by HaploReg[28, 29], and RegulomeDB[30, 31].

HaploReg (v.4.1) queries were used to identify functional annotations including the chromatin state segmentation on the Roadmap reference epigenomes, conserved regions by GERP and SiPhy, the experiments of DNAse hypersensitivity and ChIP-seq experiments from ENCODE. UCSC Genome Browser and GENCODE were used for gene annotations. We calculated the proximity of each variant to a gene.

RegulomeDB (v. 1.1, accessed on 06.15.2017) provided regulatory information of gene expression via ChIP factors, DNase sensitivity, and transcription factor (TF) binding sites from ENCODE. RegulomeDB uses the Position-Weight Matrix for TF binding, and databases JASPAR CORE, TRANSFAC and UniPROBE[79]. RegulomeDB reported Chromatin States from ROADMAP, eQTLs from several tissue types, DNase footprinting[80, 81], differentially methylated regions[82], manually curated regions and validated functional SNVs.

GWAS3D[33] (accessed on 03.15.2017) was used to analyze genetic variants that may affect regulatory elements, by integrating annotations from cell type-specific chromatin states, epigenetic modifications, sequence motifs and cross-species conservation. The regulatory elements are inferred from the genome-wide chromosome interaction data, chromatin marks in different cell types measured by high-throughput chromosome conformation capture technologies (5C, ChIA-PET and Hi-C) from ENCODE, Gene Expression Omnibus (GEO) database, published resources and regulatory factor motifs. We gathered also evidence for eQTLs based on GTEx (v. 7), GRASP software and special gene expression reported results[83, 84].

The importance of our novel and potential novel BP genes (Tables 1–5) were mined by means of four methods: enrichment analysis, protein- protein interactions (PPI), analytical gene expression cis-regulation, and analytical gene expression trans-regulation.

The GeneGO and Literature Lab of ACUMENTA software (accessed on 03.15. 2017) were used for enrichment analysis. We tested if novel genes were significantly enriched among pre-specified gene sets defined in pathways, or by shared roles in particular diseases or biological processes from Gene Ontology. The GeneGO enrichment analysis consists of matching unique gene symbols of possible targets for the "common", "similar" and "unique" sets with gene symbols in functional ontologies. The probability of a random intersection between a set of gene symbols, the size of target list with ontology entities, is estimated by *P*-value of a hypergeometric intersection. The lower *P*-value means higher relevance of the entity to the dataset, which shows in higher rating for the entity.

Literature Lab is an interface between experimentally-derived gene lists and scientific literature in a curated vocabulary of 24,000 biological and biochemical terms. It employs statistical and clustering analysis on over 17.5 million PubMed abstracts (from 01.01.1990 to the present) to identify pathways (809 pathways), diseases, compounds, cell biology and other areas of biology and biochemistry. The analysis engine compares statistically the submitted gene set to 1,000 random gene sets generated in the analysis to identify term relationships that are associated with the gene set more than by chance alone.

The BP candidate genes were assessed via PPI of databases from Biological General Repository for Interaction Datasets (BioGrid), Escherichia coli K-12 (EcoCyc), and Human Protein Database (HPRD) as summarized by the National Center for Biotechnology Information (NCBI, accessed on 02.28.2017). The gene list from PPI was evaluated using igraph package[85]. The network was built using our programs in SAS, to a Pajek format and imported into igraph in R language. “Google” PageRank algorithm provided the importance of genes (website pages) in a network, which was implemented by igraph.

Information of data analysis tools and databases, including their website links (when available) and the corresponding literature citations, are provided in S15 Table.

## Supporting information

S1 Note Description of participating studies. Study descriptions of discovery cohorts (Stage 1) and replication cohorts (Stage 2).
(DOCX)

S2 Note Summary of biological description for novel BP loci. Information summary of the nearest genes for blood pressure novel loci.
(DOCX)

S1 Fig Study design of SNV x alcohol interactions for BP. Schematic study design of the joint model of SNV main effect and SNV-alcohol consumption interaction; Blood pressure (BP) traits: systolic BP (SBP), diastolic BP (DBP), mean arterial pressure (MAP), and pulse pressure (PP); Alcohol consumption was defined by two categories: (I) as current drinking (yes/no), and (II), in the subset of drinkers, as light/heavy drinking (1–7 drinks/week or ≥8 drinks/week); Meta-analysis using a modified version of METAL: Stage 1 (discovery), Stage 2 (replication) and combined Stage 1 and Stage 2; Cohorts: European ancestry (EA), African ancestry, Asian ancestry (ASA), Hispanic ancestry (HA), Brazilian (BRA); Correlated meta-analysis in EA for four BP traits; Number of BP loci (genes), novel and reported.
(TIF)

S2 Fig QQ plots for BP traits for current drinkers. Meta-analysis distributions of–log_10_ *P*-values of observed versus–log_10_ *P*-values expected (QQ plots) for current drinkers (yes/no) European ancestry (A) and in African ancestry (B).
(TIF)

S3 Fig QQ plots for BP traits for light/heavy drinkers. Meta-analysis distributions of–log_10_ *P*-values of observed versus–log_10_ *P*-values expected (QQ plots) for light/heavy drinkers (1–7 drinks/week or ≥8 drinks/week) in European ancestry (A) and in African ancestry (B).
(TIF)

S4 Fig Regional association plots on 8p23. SNV x current drinker interaction for SBP (A), DBP (B), MAP (C) and PP (D) in European Ancestry; four linkage disequilibrium (LD) blocks (see also Fig 1).
(TIF)

S5 Fig Regional association plots on 16q12. SNV x current drinker interaction for SBP (A), DBP (B), MAP (C) and PP (D) in European Ancestry.
(TIF)

S6 Fig **Manhattan plots of combined Stage 1 and Stage 2 meta-analysis for SBP in current drinkers (A) and in light/heavy drinkers (B) in European ancestry**. Novel loci are highlighted in blue.
(TIF)

S7 Fig **Manhattan plots of combined Stage 1 and Stage 2 meta-analysis for DBP in current drinkers (A) and in light/heavy drinkers (B) in European ancestry**. Novel loci are highlighted in blue.
(TIF)

S8 Fig **Manhattan plots of combined Stage 1 and Stage 2 meta-analysis for MAP in current drinkers (A) and in light/heavy drinkers (B) in European ancestry**. Novel loci are highlighted in blue.
(TIF)

S9 Fig **Manhattan plots of combined Stage 1 and Stage 2 meta-analysis for PP in current drinkers (A) and in light/heavy drinkers (B) in European ancestry**. Novel loci are highlighted in blue.
(TIF)

S10 Fig **Manhattan plots of combined Stage 1 and Stage 2 meta-analysis for SBP in current drinkers (A) and in light/heavy drinkers (B) in African ancestry**. Novel loci are highlighted in blue.
(TIF)

S11 Fig **Manhattan plots of combined Stage 1 and Stage 2 meta-analysis for DBP in current drinkers (A) and in light/heavy drinkers (B) in African ancestry**.
(TIF)

S12 Fig **Manhattan plots of combined Stage 1 and Stage 2 meta-analysis for MAP in current drinkers (A) and in light/heavy drinkers (B) in African ancestry**.
(TIF)

S13 Fig **Manhattan plots of combined Stage 1 and Stage 2 meta-analysis for PP in current drinkers (A) and in light/heavy drinkers (B) in African ancestry**. Novel loci are highlighted in blue.
(TIF)

S14 Fig **Manhattan plots of combined Stage 1 and Stage 2 meta-analysis for SBP (A) and DBP (B) in current drinkers in Asian ancestry**.
(TIF)

S15 Fig **Manhattan plots of combined Stage 1 and Stage 2 meta-analysis for MAP (A) and PP (B) in current drinkers in Asian ancestry**.
(TIF)

S16 Fig Protein-protein interactions network. In the figure, ellipses in black represent all novel genes; ellipses in red represent novel from EA; squares in blue represent potential novel findings from African ancestry; and triangles in black from correlated-meta. Labeled with A and B free-hand circles are proteins that have two connections, while labeled within C are proteins that have three-five connections with our findings. *APP* interacts with five of our BP candidate novel genes *TTLL7*, *SOX7*, *PINX1*, *LINGO2* and *KCNMB2* (circle C).
(TIF)

S17 Fig Protein-protein interactions between tankyrase and beta-catenin. Tankyrase (from *TNKS* gene) and β-catenin (from *CTNNB1* gene).
(TIF)

S18 Fig *Wnt* signaling KEGG pathway. *TNKS* interacts with *CTNNB1*.
(TIF)

S1 Table Descriptive analyses for discovery data (Stage 1) in current drinkers. Characteristics of blood pressure (BP) in current drinkers (yes or no), within sub-sample of individuals with or without anti-hypertensive (BP Lowering) medications, and in combined samples; SBP, systolic BP; DBP, diastolic BP; MAP, mean arterial pressure; PP, pulse pressure; N, number of individuals; mean, mean levels; SD, standard deviation of mean; Min, minimum value; Max, maximum value; For each BP trait (SBP, DBP, MAP, and PP), the extreme BP values were winsorised if a BP value was greater than 6 SD, above or below the mean, setting the BP value exactly at 6 SDs from the mean.
(XLSX)

S2 Table Descriptive analyses for discovery data (Stage 1) in light/heavy drinkers. Characteristics of blood pressure (BP) in light/heavy drinkers (1–7 drinks/week or ≥8 drinks/week), within sub-sample of individuals with or without anti-hypertensive (BP Lowering) medications, and in combined samples; SBP, systolic BP; DBP, diastolic BP; MAP, mean arterial pressure; PP, pulse pressure; N, number of individuals; mean, mean levels; SD, standard deviation of mean; Min, minimum value; Max, maximum value; For each BP trait (SBP, DBP, MAP, and PP), the extreme BP values were winsorised if a BP value was greater than 6 SD, above or below the mean, setting the BP value exactly at 6 SDs from the mean.
(XLSX)

S3 Table Descriptive analyses for blood pressure (BP) stratified by alcohol consumption for discovery data (Stage 1). Characteristics of systolic BP and diastolic BP, after correcting for BP lowering medication and winsorizing observations.
(XLSX)

S4 Table Characteristics of each study and their genotype data for discovery data (Stage 1). Study design, population-based or cohort-unrelated; Principal components used; Other covariates entered in the model; Genotyping platforms; Genotyping calling algorithm; Quality Control Filters; Imputation reference panel; Number of SNVs (single nucleotide variants).
(XLSX)

S5 Table Descriptive analyses for replication data (Stage 2) in current drinkers. Characteristics of blood pressure (BP) within current drinkers (CURD: yes or no), and in alcohol combined samples; SBP, systolic BP; DBP, diastolic BP; MAP, mean arterial pressure; PP, pulse pressure; N, number of individuals; mean, mean levels; SD, standard deviation of mean; Min, minimum value; Max, maximum value.
(XLSX)

S6 Table Descriptive analyses for replication data (Stage 2) in light/heavy drinkers. Characteristics of blood pressure (BP) within light/heavy drinkers (LHD: 1–7 drinks/week or ≥8 drinks/week), and in alcohol combined samples; SBP, systolic BP; DBP, diastolic BP; MAP, mean arterial pressure; PP, pulse pressure; N, number of individuals; mean, mean levels; SD, standard deviation of mean; Min, minimum value; Max, maximum value.
(XLSX)

S7 Table Demographic statistics for replication data (Stage 2). N, Number of subjects; % Hypertensive, defined whether participants presented: (i) SBP ≥ 140 mm Hg, (ii) DBP ≥ 90 mm Hg, and/or (iii) taking anti-hypertensive medication; Mean, age mean; SD, standard deviation of mean; Min, minimum age; Max, maximum age.
(XLSX)

S8 Table Characteristics of each study and their genotype data for replication data (Stage 2). Study design, population-based or cohort-unrelated; Principal components used; Other covariates entered in the model; Genotyping platforms; Genotyping calling algorithm; Imputation reference panel; NCBI dbSNP build; Analysis software; Robust or model-based statistics; Family studies: Method of handling relatedness.
(XLSX)

S9 Table Novel SNVs/ genes associated with BP traits in multi-ancestry and specific-ancestry meta-combined results. Top significant associated SNVs are shown per gene for each trait and alcohol exposure.
(XLSX)

S10 Table SNVs/genes associated with BP traits in European ancestry. Variants previously reported for blood pressure (BP) in genome-wide association studies. The most significant associated SNVs are shown per gene for each Blood Pressure (BP) trait and alcohol status. Abbreviations: Nb, order number based on genes; SNV, single nucleotide variant; Chr, chromosome; Position, Gene, and Role in dbSNP build 150 (hg38) annotation; Role: Intronic, missense, up-stream or downstream, or intergenic (blank space) SNV; Near gene reflects genes at up to +/-500 kb and related to BP / alcohol; A1/2, Coded and non-coded alleles; Frq1, Frequency of coded allele; Trait, SBP: Systolic BP, DBP: Diastolic BP, MAP: Mean Arterial Pressure, PP: Pulse Pressure; Drink: Alcohol consumption, CURD, Current drinker (yes/no), LHD, Light(1–7 drinks/week) or heavy (≥8 drinks/week) drinker; Stage 1, Discovery cohorts; Stage 2, Replication cohorts; Stage 1 & Stage 2, Discovery and Replication combined; b_M(S.E.), beta coefficient of SNV (standard error); b_I(S.E.): SNV*E is SNV-alcohol interaction effect (standard error); *P*-Value: modified-interaction METAL *P*-Value; N, Number of subjects; *P*-Meta, *P*-Meta, modified-interaction METAL *P*-Value of Meta-analysis in combined Stage 1 and Stage 2; Het-P value, Heterogeneity *P*-Value. * These genes were detected also via correlated meta-analysis.
(XLSX)

S11 Table SNVs/genes associated with BP traits in African ancestry. Variants previously reported for blood pressure (BP) in genome-wide association studies. The most significant associated SNVs are shown per gene for each Blood Pressure (BP) trait and alcohol status. Abbreviations: Nb, order number based on genes; SNV, single nucleotide variant; Chr, chromosome; Position, Gene, and Role in dbSNP build 150 (hg38) annotation; Role: Intronic or intergenic (blank space) SNV; Near gene reflects genes at up to +/-500 kb and related to BP / alcohol; A1/2, Coded and non-coded alleles; Frq1, Frequency of coded allele; Trait, SBP: Systolic BP, DBP: Diastolic BP, MAP: Mean Arterial Pressure, PP: Pulse Pressure; Drink: Alcohol consumption, CURD, Current drinker (yes/no); Stage 1, Discovery cohorts; Stage 2, Replication cohorts; Stage 1 & Stage 2, Discovery and Replication combined; b_M(S.E.), beta coefficient of SNV (standard error); b_I(S.E.): SNV*E is SNV-alcohol interaction effect (standard error); *P*-Value: modified-interaction METAL *P*-Value; N, Number of subjects; *P*-Meta, *P*-Meta, modified-interaction METAL *P*-Value of Meta-analysis in combined Stage 1 and Stage 2; Het-P value, Heterogeneity *P*-Value. * These genes were detected also via correlated meta-analysis.
(XLSX)

S12 Table SNVs/genes associated with BP traits in multi-ancestry meta-analysis in combined Stage 1 and Stage 2. Variants previously reported for blood pressure (BP) in genome-wide association studies. The most significant associated SNVs are shown per gene for each Blood Pressure (BP) trait and alcohol status. Abbreviations: Nb, order number based on genes; SNV, single nucleotide variant; Chr, chromosome; Position, Gene, and Role in dbSNP build 150 (hg38) annotation; Role: Intronic, missense, up-stream or downstream, or intergenic (blank space) SNV; Near gene reflects genes at up to +/-500 kb and related to BP / alcohol; A1/2, Coded and non-coded alleles; Frq1, Frequency of coded allele; Ancestry, EA: European Ancestry, AA: African American Ancestry, ASA: Asian American Ancestry, HA: Hispanic Ancestry; Trait, SBP: Systolic BP, DBP: Diastolic BP, MAP: Mean Arterial Pressure, PP: Pulse Pressure; Drink: Alcohol consumption, CURD, Current drinker (yes/no), LHD, Light(1–7 drinks/week) or heavy (≥8 drinks/week) drinker; Stage 1 and Stage 2, Combined Discovery and Replication; b_M, beta coefficient of SNV; b_I: SNV*E is SNV-alcohol interaction effect; *P*-Value, modified-interaction METAL *P*-Value of meta-analysis in combined Stage 1 and Stage 2; N, Number of subjects; Het-P value, Heterogeneity *P*-Value.
(XLSX)

S13 Table SNVs/genes associated with BP traits for regulatory features using HaploReg and RegulomeDB. Association findings from European Ancestry (novel), African Ancestry (potential) and correlated meta-analysis (novel variants). The annotation of variants was sourced from NCBI dbSNP build 138 (hg19) during the analyses and updated to dbSNP build 150 (hg38) for reporting results. Abbreviations: Nb, order number based on SNVs; Position, dbSNP build 150 (hg38) annotation; Variant, single nucleotide variant (SNV); Ref, reference allele; Alt, alternative allele; AFR Freq, Freq of Ref in African ancestry; ASN Freq, Freq of Ref in East Asian ancestry; EUR Freq, Freq of Ref in European ancestry; GERP cons and Siphy cons, measured conserved regions. RegulomeDB scoring has classes defined as 1b, 1d and 1f: likely to affect binding and linked to expression of a gene target, with details: 1b (eQTL + TF binding + any motif + DNase footprint + DNase peak); 1d (eQTL + TF binding + any motif + DNase peak); 1f (eQTL + TF binding/DNase peak), 2a and 2b: likely to affect binding, 3a: less likely to affect binding, 4, 5, and 6: minimal binding evidence, and 7: no data. This software was accessed on 06.15.2017. Regulatory function measured by Promoter histone marks, Enhancer histone marks, DNase (DNAse hypersensitivity), Proteins bound, Motifs changed.
(XLSX)

S14 Table Novel SNVs/genes associated with BP traits for eSNV/eQTL using GTEx. Target genes (Tissues and *P*-Values). Association findings from European Ancestry (novel) and correlated meta-analysis (novel variants). The annotation of variants was sourced from NCBI dbSNP build 138 (hg19) during the analyses and updated to dbSNP build 150 (hg38) for reporting results. Abbreviations: Nb, order number based on SNVs; Position, dbSNP build 150 (hg38) annotation; Variant, single nucleotide variant (SNV); Ref, reference allele; Alt, alternative allele; AFR Freq, Freq of Ref in African ancestry; ASN Freq, Freq of Ref in East Asian ancestry; EUR Freq, Freq of Ref in European ancestry. * RegulomeDB scoring has classes defined as 1b, 1d and 1f: likely to affect binding and linked to expression of a gene target, with details: 1b (eQTL + TF binding + any motif + DNase footprint + DNase peak); 1d (eQTL + TF binding + any motif + DNase peak); 1f (eQTL + TF binding/DNase peak), 2a and 2b: likely to affect binding, 3a: less likely to affect binding, 4, 5, and 6: minimal binding evidence, and 7: no data. This software was accessed on 06.15.2017. Regulatory function measured by Promoter histone marks, Enhancer histone marks, DNase (DNAse hypersensitivity), Proteins bound, Motifs changed.
(XLSX)

S15 Table Data analysis tools and databases. (DOCX)
