# Supplementary material for: Novel genetic associations for blood pressure identified via gene-alcohol interaction in up to 570K individuals across multiple ancestries
Source: PLoS One. 2018 Jun 18;13(6):e0198166. doi: 10.1371/journal.pone.0198166 (PMC6005576; doi:10.1371/journal.pone.0198166)
Supplement: S1 Note — Study descriptions of discovery cohorts (Stage 1) and replication cohorts (Stage 2). (DOCX) [file pone.0198166.s001.docx]

**S1 Note. Description of participating studies**

The following are brief descriptions of each discovery and replication study:

**1.1. Discovery study description (Stage 1)**

**AGES (Age Gene/Environment Susceptibility Reykjavik Study):** The AGES Reykjavik study originally comprised a random sample of 30,795 men and women born in 1907-1935 and living in Reykjavik in 1967. A total of 19,381 people attended, resulting in a 71% recruitment rate. The study sample was divided into six groups by birth year and birth date within month. One group was designated for longitudinal follow up and was examined in all stages; another was designated as a control group and was not included in examinations until 1991. Other groups were invited to participate in specific stages of the study. Between 2002 and 2006, the AGES Reykjavik study re-examined 5,764 survivors of the original cohort who had participated before in the Reykjavik Study. The midlife data blood pressure measurement was taken from stage 3 of the Reykjavik Study (1974-1979), if available. Half of the cohort attended during this period. Otherwise an observation was selected closest in time to the stage 3 visit. The supine blood pressure was measured twice by a nurse using a mercury sphygmomanometer after 5 minutes’ rest following World Health Organization recommendations.

**ARIC (Atherosclerosis Risk in Communities):** The ARIC study is a population-based prospective cohort study of cardiovascular disease sponsored by the National Heart, Lung, and Blood Institute (NHLBI). ARIC included 15,792 individuals, predominantly European American and African American, aged 45-64 years at baseline (1987-89), chosen by probability sampling from four US communities. Cohort members completed three additional triennial follow-up examinations and a fifth exam in 2011-2013. The ARIC study has been described in detail previously[1]. Blood pressure was measured using a standardized Hawksley random-zero mercury column sphygmomanometer with participants in a sitting position after a resting period of 5 minutes. The size of the cuff was chosen according to the arm circumference. Three sequential recordings for systolic and diastolic blood pressure were obtained; the mean of the last two measurements was used in this analysis, discarding the first reading. Blood pressure lowering medication use was recorded from the medication history.

**Baependi (Baependi Heart Study (Brazil):** The Baependi is an ongoing family-based cohort conducted in a rural town of the state of Minas Gerais. The study has enrolled approximate 2,200 individuals (over 10% of the town’s adult population) and 10-year follow up period of longitudinal data. Briefly, probands were selected at random across 11 out of the 12 census districts in Baependi. After enrolment, the proband's first-degree (parents, siblings, and offspring), second-degree (half-siblings, grandparents/grandchildren, uncles/aunts, nephews/nieces, and double cousins), and third-degree (first cousins, great uncles/aunts, and great nephews/nieces) relatives, and his/her respective spouse's relatives resident both within Baependi (municipal and rural area) and surrounding towns were invited to participate. Only individuals age 18 and older were eligible to participate in the study. The study is conducted from a clinic/office in an easily accessible sector of the town, where the questionnaires were completed. A broad range of phenotypes ranging from cardiovascular, neurocognitive, psychiatric, imaging, physiologic and several layers of endophenotypes like metabolomics and lipidomics have been collected throughout the years Details about follow-up visits and available data can be found in the cohort profile paper[2]. DNA samples were genotyped using the Affymetrix 6.0 genechip. After quality control, the data were prephased using SHAPEIT and imputed using IMPUTE2 based on 1000 Genomes haplotypes.

**CARDIA (Coronary Artery Risk Development in Young Adults):** CARDIA is a prospective multicenter study with 5,115 adults Caucasian and African American participants of the age group 18-30 years, recruited from four centers at the baseline examination in 1985-1986. The recruitment was done from the total community in Birmingham, AL, from selected census tracts in Chicago, IL and Minneapolis, MN; and from the Kaiser Permanente health plan membership in Oakland, CA. The details of the study design for the CARDIA study have been previously published[3]. Nine examinations have been completed since initiation of the study, respectively in the years 0, 2, 5, 7, 10, 15, 20, 25, and 30. Written informed consent was obtained from participants at each examination and all study protocols were approved by the institutional review boards of the participating institutions. Systolic and diastolic blood pressure was measured in triplicate on the right arm using a random-zero sphygmomanometer with the participant seated and following a 5-min. rest. The average of the second and third measurements was taken as the blood pressure value. Blood pressure medication use was obtained by questionnaire.

**CHS (Cardiovascular Health Study):** CHS is a population-based cohort study of risk factors for cardiovascular disease in adults 65 years of age or older conducted across four field centers [4]. The original predominantly European ancestry cohort of 5,201 persons was recruited in 1989-1990 from random samples of the Medicare eligibility lists and an additional predominately African-American cohort of 687 persons was enrolled in 1992-93 for a total sample of 5,888. Blood samples were drawn from all participants at their baseline examination and DNA was subsequently extracted from available samples. European ancestry participants were excluded from the GWAS study sample due to prevalent coronary heart disease, congestive heart failure, peripheral vascular disease, valvular heart disease, stroke, or transient ischemic attack at baseline. After QC, genotyping was successful for 3271 European ancestry and 823 African-American participants. CHS was approved by institutional review committees at each site and individuals in the present analysis gave informed consent including consent to use of genetic information for the study of cardiovascular disease. Research staff with central training in blood pressure measurement assessed repeated right-arm seated systolic and diastolic blood pressure levels at baseline with a Hawksley random-zero sphygmomanometer.

**ERF (Erasmus Rucphen Family study):** ERF is a family based study that includes inhabitants of a genetically isolated community in the South-West of the Netherlands, studied as part of the Genetic Research in Isolated Population (GRIP) program. The goal of the study is to identify the risk factors in the development of complex disorders. Study population includes approximately 3,000 individuals who are living descendants of 22 couples who lived in the isolate between 1850 and 1900 and had at least six children baptized in the community church. All data were collected between 2002 and 2005[5, 6]. All participants gave informed consent, and the Medical Ethics Committee of the Erasmus University Medical Centre approved the study.

**FamHS (Family Heart Study):** The NHLBI FamHS study design, collection of phenotypes and covariates as well as clinical examination have been previously described (<https://dsgweb.wustl.edu/fhscc/>)[7]. In brief, the FamHS recruited 1,200 families (approximately 6,000 individuals), half randomly sampled, and half selected because of an excess of coronary heart disease (CHD) or risk factor abnormalities as compared with age- and sex-specific population rates. The participants were sampled from four population-based parent studies: the Framingham Heart Study, the Utah Family Tree Study, and two centers for the Atherosclerosis Risk in Communities study (ARIC: Minneapolis, and Forsyth County, NC). These individuals attended a clinic exam (1994-1996) and a broad range of phenotypes were assessed in the general domains of CHD, atherosclerosis, cardiac and vascular function, inflammation and hemostasis, lipids and lipoproteins, blood pressure, diabetes and insulin resistance, pulmonary function, diet, education, socioeconomic status, habitual behavior, physical activity, anthropometry, medical history and medication use. Approximately 8 years later, study participants belonging to the largest pedigrees were invited for a second clinical exam (2002-04). The most important CHD risk factors were measured again, including lipids, parameters of glucose metabolism, blood pressure, anthropometry, and several biochemical and hematologic markers. In addition, a computed tomography examination provided measures of coronary and aortic calcification, and abdominal and liver fat burden. Medical history and medication use was updated. A total of 2,756 European ancestry subjects in 510 extended random and high CHD risk families were studied. Also, 633 African ancestry subjects were recruited at ARIC field center at the University of Alabama in Birmingham. Informed consent was obtained from all participants.

**FHS (Framingham Heart Study):** FHS began in 1948 with the recruitment of an original cohort of 5,209 men and women (mean age 44 years; 55 percent women). In 1971 a second generation of study participants was enrolled; this cohort (mean age 37 years; 52% women) consisted of 5,124 children and spouses of children of the original cohort. A third-generation cohort of 4,095 children of offspring cohort participants (mean age 40 years; 53 percent women) was enrolled in 2002-2005 and are seen every 4 to 8 years. Details of study designs for the three cohorts are summarized elsewhere. At each clinic visit, a medical history was obtained with a focus on cardiovascular content, and participants underwent a physical examination including measurement of height and weight from which BMI was calculated. Systolic and diastolic blood pressures were measured twice by a physician on the left arm of the resting and seated participant using a mercury column sphygmomanometer. Blood pressures were recorded to the nearest even number. The means of two separate systolic and diastolic blood pressure readings at each clinic examination were used for statistical analyses.

**GENOA (Genetic Epidemiology Network of Arteriopathy):** GENOA is one of four networks in the NHLBI Family-Blood Pressure Program (FBPP)[8, 9]. GENOA's long-term objective is to elucidate the genetics of target organ complications of hypertension, including both atherosclerotic complications involving the heart, brain, kidneys, and peripheral arteries. The longitudinal GENOA Study recruited European-American and African-American sibships with at least 2 individuals with clinically diagnosed essential hypertension before age 60 years. All other members of the sibships were invited to participate regardless of their hypertension status. Participants were diagnosed with hypertension if they had either 1) a previous clinical diagnosis of hypertension by a physician with current anti-hypertensive treatment, or 2) an average systolic blood pressure ≥ 140 mm Hg or diastolic blood pressure ≥ 90 mm Hg based on the second and third readings at the time of their clinic visit. Exclusion criteria were secondary hypertension, alcoholism or drug abuse, pregnancy, insulin-dependent diabetes mellitus, or active malignancy. During the first exam (1995-2000), 1,583 European Americans from Rochester, MN and 1,854 African Americans from Jackson, MS were examined. Between 2000 and 2005, 1,241 of the European Americans and 1,482 of the African Americans returned for a second examination. Because African-American probands for GENOA were recruited through the Atherosclerosis Risk in Communities (ARIC) Jackson field center participants, we excluded ARIC participants from analyses.

**GenSalt (Genetic Epidemiology Network of Salt Sensitivity):** GenSalt is a multi-center, family based study designed to identify, through dietary sodium and potassium intervention, salt-sensitivity susceptibility genes which may underlie essential hypertension in rural Han Chinese families. Approximately 629 families with at least one ‘proband’ with high blood pressure were recruited and tested for a wide variety of physiological, metabolic and biochemical measures at baseline and at multiple times during the 3-week intervention. The intervention consisted of one week on a low sodium diet, followed by one week on a high sodium diet, and finally one week on a high sodium diet with a potassium supplement.

**GOLDN (Genetics of Diet and Lipid Lowering Network):** GOLDN is a multi-center family pharmacogenetic study that is investigating gene- environment interactions on lipid profiles. 1,200 subjects in extended pedigrees were measured before and after two environmental exposures: 1) a dietary fat challenge to assess genetic regulators of fat uptake and clearance and 2) a 3-week clinical trial of fenofibrate to assess pharmacogenetic influences on response to treatment. The goals of the study are to identify and characterize genetic loci that predict the lipid profile treatment responses. https://dsgweb.wustl.edu/PROJECTS/MP5.html

**HANDLS (Healthy Aging in Neighborhoods of Diversity across the Life Span):** HANDLS is a community-based, longitudinal epidemiologic study examining the influences of race and socioeconomic status (SES) on the development of age-related health disparities among a sample of socioeconomically diverse African Americans and whites. This unique study will assess over 20-year period physical parameters and also evaluate genetic, biologic, demographic, and psychosocial, parameters of African American and white participants in higher and lower SES to understand the driving factors behind persistent black-white health disparities in overall longevity, cardiovascular disease, and cognitive decline. The study recruited 3,722 participants from Baltimore, MD with a mean age of 47.7 years, 2,200 African Americans and 1,522 whites, with 41% reporting household incomes below the 125% poverty delimiter.

Genotyping was done on a subset of self-reporting African American participants by the Laboratory of Neurogenetics, National Institute on Aging, National Institutes of Health (NIH). A larger genotyping effort included a small subset of self-reporting European ancestry samples. This research was supported by the Intramural Research Program of the NIH, NIA and the National Center on Minority Health and Health Disparities.

**Health ABC (Health, Aging, and Body Composition):** Cohort description: The Health ABC study is a prospective cohort study investigating the associations between body composition, weight-related health conditions, and incident functional limitation in older adults. Health ABC enrolled well-functioning, community-dwelling black (n=1281) and white (n=1794) men and women aged 70-79 years between April 1997 and June 1998. Participants were recruited from a random sample of white and all black Medicare eligible residents in the Pittsburgh, PA, and Memphis, TN, metropolitan areas. Participants have undergone annual exams and semi-annual phone interviews. The current study sample consists of 1559 white participants who attended the second exam in 1998-1999 with available genotyping data.

Genotyping: Genotyping was performed by the Center for Inherited Disease Research (CIDR) using the Illumina Human1M-Duo BeadChip system. Samples were excluded from the dataset for the reasons of sample failure, genotypic sex mismatch, and first-degree relative of an included individual based on genotype data. Genotyping was successful in 1663 Caucasians. Analysis was restricted to SNPs with minor allele frequency ≥ 1%, call rate ≥ 97% and HWE p ≥1.0 x 10^-6^. Genotypes were available on 914,263 high quality SNPs for imputation based on the HapMap CEU (release 22, build 36) using the MACH software (version 1.0.16). A total of 2,543,888 imputed SNPs was analyzed for association with vitamin D levels. Association analysis: Linear regression models were used to generate cohort-specific residuals of naturally log transformed vitamin D levels adjusted for age, sex, BMI and season defined as summer (June-August), fall (September-November), winter (December to February) and spring (March to May) standardized to have mean 0 and variance of 1. Association between the additively coded SNP genotypes and the vitamin D residuals standardized was assessed using linear regression models. For imputed SNPs, expected number of minor alleles (i.e. dosage) was used in assessing association with the vitamin D residuals.

**HERITAGE (Health, Risk Factors, Exercise Training and Genetics):** The HERITAGE is the only known family-based study of exercise intervention to evaluate the role of genes and sequence variants involved in the response to a physically active lifestyle. The current study is based on the data collected at baseline of the study from 99 White families (244 males, 255 females). All subjects were required to be sedentary and free of chronic diseases at baseline. There are over 18 trait domains (e.g. dietary, lipids and lipoproteins, glucose and insulin metabolism [fasting and IVGTT], steroids, body composition and body fat distribution, cardiorespiratory fitness), for a grand total of over one thousand variables. Moreover, most of the outcome traits were measured twice on two separate days both at baseline and after exercise training was completed. Marker data include a genome-wide linkage scan and GWAS, in addition to a large number of candidate genes.

**HUFS (Howard University Family Study):** HUFS followed a population-based selection strategy designed to be representative of African American families living in the Washington, DC metropolitan area. The major objectives of the HUFS were to study the genetic and environmental basis of common complex diseases including hypertension, obesity and associated phenotypes. Participants were sought through door-to-door canvassing, advertisements in local print media and at health fairs and other community gatherings. To maximize the utility of this cohort for the study of multiple common traits, families were not ascertained based on any phenotype. During a clinical examination, demographic information was collected by interview.

**HyperGEN (Hypertension Genetic Epidemiology Network):** HyperGEN is a family-based study that looks at the genetic causes of hypertension and related conditions in EA and AA subjects. HyperGEN recruited hypertensive sibships, along with their normotensive adult offspring, and an age-matched random sample. HyperGEN has collected data on 2,471 Caucasian-American subjects and 2,300 African-American subjects, from five field centers in Alabama, Massachusetts, Minnesota, North Carolina, and Utah.

**IGMM (Institute of Genetics and Molecular Medicine):** IGMM oversees three participating studies: CROATIA-Korcula; CROATIA-Vis; GS:SFHS (Generation Scotland: Scottish Family Health Study. **CROATIA-Korcula:** The CROATIA-Korcula study is a family-based, cross-sectional study in the isolated island of Korcula that included 965 examinees aged 18-95. Blood samples were collected in 2007 along with many clinical and biochemical measures and lifestyle and health questionnaires. **CROATIA-Vis:** The CROATIA-Vis study is a family-based, cross-sectional study in the isolated island of Vis that included 1,056 examinees aged 8-93. Blood samples were collected in 2003 and 2004 along with many clinical and biochemical measures and lifestyle and health questionnaires. **GS:SFHS:** The Generation Scotland (www.generationscotland.org) Scottish Family Health Study (GS:SFHS) is a family-based genetic epidemiology cohort with DNA, other biological samples (serum, urine and cryopreserved whole blood) and socio-demographic and clinical data from approximately 24,000 volunteers, aged 18-98 years, in ~7,000 family groups. An important feature of GS:SFHS is the breadth of phenotype information, including detailed data on cognitive function, personality traits and mental health. Although data collection was cross-sectional, GS:SFHS becomes a longitudinal cohort as a result of the ability to link to routine NHS data, using the community health index (CHI) number.

**JHS (Jackson Heart Study):** JHS is a longitudinal, community-based observational cohort study investigating the role of environmental and genetic factors in the development of cardiovascular disease in African Americans. Between 2000 and 2004, a total of 5301 participants were recruited from a tri-county area (Hinds, Madison, and Rankin Counties) that encompasses Jackson, MS. Details of the design and recruitment for the Jackson Heart Study cohort has been previously published[10-12]. Briefly, approximately 30% of participants were former members of the Atherosclerosis Risk in Communities (ARIC) study. The remainder were recruited by either 1) random selection from the Accudata list, 2) commercial listing, 3) a constrained volunteer sample, in which recruitment was distributed among defined demographic cells in proportions designed to mirror those in the overall population, or through the Jackson Heart Study Family Study.

**MESA (Multi-Ethnic Study of Atherosclerosis):** MESA is a study of the characteristics of subclinical cardiovascular disease and the risk factors that predict progression to clinically overt cardiovascular disease or progression of the subclinical disease. MESA consisted of a diverse, population-based sample of an initial 6,814 asymptomatic men and women aged 45-84. 38 percent of the recruited participants were white, 28 percent African American, 22 percent Hispanic, and 12 percent Asian, predominantly of Chinese descent. Participants were recruited from six field centers across the United States: Wake Forest University, Columbia University, Johns Hopkins University, University of Minnesota, Northwestern University and University of California - Los Angeles. Each participant received an extensive physical exam and determination of coronary calcification, ventricular mass and function, flow-mediated endothelial vasodilation, carotid intimal-medial wall thickness and presence of echogenic lucencies in the carotid artery, lower extremity vascular insufficiency, arterial wave forms, electrocardiographic (ECG) measures, standard coronary risk factors, socio-demographic factors, lifestyle factors, and psychosocial factors. Selected repetition of subclinical disease measures and risk factors at follow-up visits allowed study of the progression of disease. Participants are being followed for identification and characterization of cardiovascular disease events, including acute myocardial infarction and other forms of coronary heart disease (CHD), stroke, and congestive heart failure; for cardiovascular disease interventions; and for mortality. The first examination took place over two years, from July 2000 - July 2002. It was followed by four examination periods that were 17-20 months in length. Participants have been contacted every 9 to 12 months throughout the study to assess clinical morbidity and mortality[13].

**NEO (The Netherlands Epidemiology of Obesity study):** The NEO was designed for extensive phenotyping to investigate pathways that lead to obesity-related diseases. The NEO study is a population-based, prospective cohort study that includes 6,671 individuals aged 45–65 years, with an oversampling of individuals with overweight or obesity. At baseline, information on demography, lifestyle, and medical history have been collected by questionnaires. In addition, samples of 24-h urine, fasting and postprandial blood plasma and serum, and DNA were collected. Genotyping was performed using the Illumina HumanCoreExome chip, which was subsequently imputed to the 1000 genome reference panel. Participants underwent an extensive physical examination, including anthropometry, electrocardiography, spirometry, and measurement of the carotid artery intima-media thickness by ultrasonography. In random subsamples of participants, magnetic resonance imaging of abdominal fat, pulse wave velocity of the aorta, heart, and brain, magnetic resonance spectroscopy of the liver, indirect calorimetry, dual energy X-ray absorptiometry, or accelerometry measurements were performed. The collection of data started in September 2008 and completed at the end of September 2012. Participants are currently being followed for the incidence of obesity-related diseases and mortality.

**Pelotas (The 1982 Pelotas Birth Cohort Study, Brazil):** The maternity hospitals in Pelotas, a southern Brazilian city (current population ~330,000), were visited daily in the year of 1982. The 5,914 live-born whose families lived in the urban area were examined and their mothers interviewed. Information was obtained for more than 99% of the live births. These subjects have been followed-up at the following mean ages: 11.3 months (all children born from January to Abril 1982; n=1457), 19.4 months (entire cohort; n=4934), 43.1 months (entire cohort; n=4742), 13.1 years (random subsample; n=715), 14.7 years (systematic subsample; n=1076); 18.2 (male cohorts attending to compulsory Army recruitment examination; n=2250), 18.9 (systematic subsample; n=1031), 22.8 years (entire cohort; n=4297) and 30.2 years (entire cohort; n=3701). Details about follow-up visits and available data can be found in the two Cohort Profile papers[14, 15]. DNA samples (collected at the mean age of 22.8 years) were genotyped for ~2.5 million of SNPs using the Illumina HumanOmni2.5-8v1 array (which includes autosomal, X and Y chromosomes, and mitochondrial variants). After quality control, the data were pre-phased using SHAPEIT and imputed using IMPUTE2 based on 1000 Genomes haplotypes.

**RS (Rotterdam Study):** RS is a prospective, population-based cohort study among individuals living in the well-defined Ommoord district in the city of Rotterdam in The Netherlands. The aim of the study is to determine the occurrence of cardiovascular, neurological, ophthalmic, endocrine, hepatic, respiratory, and psychiatric diseases in elderly people. The cohort was initially defined in 1990 among approximately 7,900 persons, aged 55 years and older, who underwent a home interview and extensive physical examination at the baseline and during follow-up rounds every 3-4 years (RS-I). Cohort was extended in 2000/2001 (RS-II, 3,011 individuals aged 55 years and older) and 2006/2008 (RS-III, 3,932 subjects, aged 45 and older)[16]. Written informed consent was obtained from all participants and the Medical Ethics Committee of the Erasmus Medical Center, Rotterdam, approved the study.

**SCES (Singapore Chinese Eye Study):** SCES is a population-based, cross-sectional study of Chinese adults aged 40–80+ years residing in the South-Western part of Singapore, which is part of the Singapore Epidemiology of Eye Disease (SEED). Age stratified random sampling was used to select 6,350 eligible participants, of which 3,300 participated in the study (73% response rate). Detailed methodology has been published[17]. Two readings of blood pressure were taken from participants after 5 minutes of rest, seated, using an automated blood pressure monitor (Dinamap Pro100V2; Criticon, Norderstedt, Germany) by trained observers. One of two cuff sizes (regular, large) was chosen on the basis of the circumference of the participant’s arm. A third reading was performed if the difference between two readings of either the systolic blood pressure was greater than 10mmHg or the diastolic blood pressure was greater than 5mmHg. The mean values of the closest two readings were calculated.

**SiMES (Singapore Malay Eye Study):** SiMES is a population-based cross-sectional epidemiological study of 3,280 individuals from one of the three major ethnic groups residing in Singapore. SiMES is part of the Singapore Epidemiology of Eye Disease (SEED) study. In summary, 5,600 individuals have been selected by an age-stratified sampling strategy. Among these 4,168 individuals are eligible for this study. 3,280 individuals finally participated in the study. All subjects were Malay and aged 40-79 years[18]. Two readings of blood pressure were taken from participants after 5 minutes of rest, seated, using an automated blood pressure monitor (Dinamap Pro100V2; Criticon, Norderstedt, Germany) by trained observers. One of two cuff sizes (regular, large) was chosen on the basis of the circumference of the participant’s arm. A third reading was performed if the difference between two readings of either the systolic blood pressure was greater than 10mmHg or the diastolic blood pressure was greater than 5mmHg. The mean values of the closest two readings were calculated.

**SINDI (Singapore Indian Eye Study):** SINDI is a population-based, cross-sectional study of Asian Indian adults aged 40–80+ years residing in the South-Western part of Singapore, which is part of the Singapore Epidemiology of Eye Disease (SEED). Age stratified random sampling was used to select 6,350 eligible participants, of which 3,400 participated in the study (75.6% response rate). Detailed methodology has been published[17]. Two readings of blood pressure were taken from participants after 5 minutes of rest, seated, using an automated blood pressure monitor (Dinamap Pro100V2; Criticon, Norderstedt, Germany) by trained observers. One of two cuff sizes (regular, large) was chosen on the basis of the circumference of the participant’s arm. A third reading was performed if the difference between two readings of either the systolic blood pressure was greater than 10mmHg or the diastolic blood pressure was greater than 5mmHg. The mean values of the closest two readings were calculated.

**SP2 (Singapore Prospective Study Program):** SP2 is a population-based study of diabetes and cardiovascular disease in Singapore. It first surveyed subjects (Chinese, Malay and Indian) from four cross-sectional studies that were conducted in Singapore between 1982 and 1998. Subjects were between the ages of 24-95 years and represented a random sample of the Singapore population. Subjects were re-visited between 2003 and 2007. Among the 10,747 individuals who were eligible, 5,157 subjects completed a questionnaire and the subsequent clinical examinations. Data from this re-visit were utilized for this study[19]. Two readings of blood pressure were taken from participants after 5 min of rest, seated, using an automated blood pressure monitor (Dinamap Pro100V2; Criticon, Norderstedt, Germany) by trained observers. One of two cuff sizes (regular, large) was chosen on the basis of the circumference of the participant’s arm. A third reading was performed if the difference between two readings of either the systolic blood pressure was greater than 10mmHg or the diastolic blood pressure was greater than 5mmHg. The mean values of the closest two readings were calculated.

**SCHS-CHD (Singapore Chinese Health Study - Coronary Heart Disease):** SCHS-CHD is a case-control study of coronary heart disease that was nested within the Singapore Chinese Health Study (SCHS), a prospective cohort study of 63,257 Singaporean Chinese men and women aged 45-74 years living in Singapore. We selected cases and controls from participants that provided blood samples and were free of coronary heart disease and stroke at the time of blood collection (N=24,454). Cases (N=760) had acute myocardial infarction (AMI) or died of coronary heart disease. AMI was identified through the Singapore Myocardial Infarction Registry or through the nationwide hospital discharge database followed by confirmation of AMI by cardiologists’ review of medical records using the Multi-Ethnic Study of Atherosclerosis criteria (available at: http://www.mesa-nhlbi.org/manuals.aspx). Coronary heart disease deaths were identified through the Singapore Registry of Births and Deaths (ICD9 410-414 as first stated cause of death). Matched controls (N=1,491) were selected using a risk-set sampling strategy. Controls were participants who were alive and free of coronary heart disease at the time of the diagnosis or death of the index cases and were matched for age, sex, dialect group, year of recruitment and date of blood collection. In-person interviews and phlebotomy were conducted before the onset of disease and non-fasting venous blood was stored at -80^0^C for extraction of DNA and blood biochemistry.

**WGHS (Women’s Genome Health Study):** WGHS is a prospective cohort of female North American health care professionals representing participants in the Women’s Health Study (WHS) trial who provided a blood sample at baseline and consent for blood-based analyses. Participants in the WHS were 45 years or older at enrollment and free of cardiovascular disease, cancer or other major chronic illness. The current data are derived from 23,294 WGHS participants for whom whole genome genotype information was available at the time of analysis and for whom self-reported European ancestry could be confirmed by multidimensional scaling analysis of 1,443 ancestry informative markers in PLINK v. 1.06. At baseline, BP and lifestyle habits related to smoking, consumption of alcohol, and physical activity as well as other general clinical information were ascertained by a self-reported questionnaire, an approach which has been validated in the WGHS demographic, namely female health care professionals. Questionnaires recorded systolic BP in 9 categories (<110, 110-119, 120-129, 130-139, 140-149, 150-159, 160-169, 170-179, ≥180 mmHg), and diastolic BP in 7 categories (<65, 65-74, 75-84, 85-89, 90-94, 95-104, ≥105 mmHg). All analyses treated these BP responses as quantitative variables representing each category with its midpoint value. Hypertension was defined as one or more of reported physician diagnosis, systolic BP ≥140 mmHg, or diastolic BP ≥ 90 mmHg.

**WHI (Women’s Health Initiative):** WHI is a long-term national health study that focuses on strategies for preventing common diseases such as heart disease, cancer and fracture in postmenopausal women. A total of 161,838 women aged 50–79 years old were recruited from 40 clinical centers in the US between 1993 and 1998. WHI consists of an observational study, two clinical trials of postmenopausal hormone therapy (HT, estrogen alone or estrogen plus progestin), a calcium and vitamin D supplement trial, and a dietary modification trial[20]. Study recruitment and exclusion criteria have been described previously[20]. Recruitment was done through mass mailing to age-eligible women obtained from voter registration, driver’s license and Health Care Financing Administration or other insurance list, with emphasis on recruitment of minorities and older women[21]. Exclusions included participation in other randomized trials, predicted survival < 3 years, alcoholism, drug dependency, mental illness and dementia. For the CT, women were ineligible if they had a systolic BP > 200 mm Hg or diastolic BP > 105 mm Hg, a history of hypertriglyceridemia or breast cancer. Study protocols and consent forms were approved by the IRB at all participating institutions. Socio-demographic characteristics, lifestyle, medical history and self-reported medications were collected using standardized questionnaires at the screening visit. Physical measures of height, weight and blood pressure were measured at a baseline clinical visit[21]. BP was measured by certified staff using standardized procedures and instruments[22]. Two BP measures were recorded after 5 minutes’ rest using a mercury sphygmomanometer. Appropriate cuff bladder size was determined at each visit based on arm circumference. Diastolic BP was taken from the phase V Korotkoff measures. The average of the two measurements, obtained 30 seconds apart, was used in analyses. The genome wide association study (GWAS) non-overlapping samples are composed of a case-control study (WHI Genomics and Randomized Trials Network – GARNET, which included all coronary heart disease, stroke, venous thromboembolic events and selected diabetes cases that happened during the active intervention phase in the WHI HT clinical trials and aged matched controls), women selected to be "representative" of the HT trial (mostly younger white HT subjects that were also enrolled in the WHI memory study - WHIMS) and the WHI SNP Health Association Resource (WHI SHARe), a randomly selected sample of 8,515 African American and 3,642 Hispanic women from WHI. GWAS was performed using Affymetrix 6.0 (WHI-SHARe), HumanOmniExpressExome-8v1_B (WHIMS), and Illumina HumanOmni1-Quad v1-0 B (GARNET). Extensive quality control (QC) of the GWAS data included alignment (“flipping”) to the same reference panel, imputation to the 1000G data (using the recent reference panel - v3.20101123), identification of genetically related individuals, and computations of principal components (PCs) using methods developed by Price et al. (using EIGENSOFT software 53), and finally the comparison with self-reported ethnicity. After QC and exclusions from analysis protocol, the number of women included in analysis is 4,423 whites for GARNET, 5,202 white for WHIMS, 7,919 for SHARe African American and 3,377 for SHARe Hispanics.

**1.2. Replication study description (Stage 2)**

**AA-DHS (African American Diabetes Heart Study):** AA-DHS objectives are to improve understanding of ethnic differences in CAC and CP in populations of African and European ancestry. The AA-DHS consists of self-reported African Americans with T2D recruited from two Wake Forest School of Medicine (WFSM) studies: the family-based Diabetes Heart Study (DHS) and unrelated individuals in the AA-DHS. DHS is a cross-sectional study of European American and African American families with siblings concordant for T2D. AA-DHS started after DHS and enrolled unrelated African Americans. The AA-DHS GWAS utilized the Illumina 5M chip with imputation to 1,000 Genomes.

**ASCOT (Anglo-Scandinavian Cardiac Outcomes Trial):** ASCOT is a randomised control clinical trial investigating the cardiac outcomes of blood pressure lowering and lipid lowering treatments. Of 19,342 hypertensive patients (40–79 years of age with at least three other cardiovascular risk factors) who were randomized to one of two antihypertensive regimens in ASCOT (atenolol, Beta-Blocker vs amlodipine, Calcium-Channel-Blocker), 10,305 patients with non-fasting total cholesterol concentrations of 6.5 mmol/l or less (measured at the non-fasting screening visit) had been randomly assigned additional atorvastatin 10 mg or placebo. Only a proportion of United Kingdom, Irish, Sweden, Norway, Finland and Denmark consented to contribute DNA and participate in genetic studies[23].

**BBJ (Biobank Japan Project):** BBJ was established in 2003 with the aim of the implementation of personalized medicine as a leading project of Ministry of Education, Culture, Sports, Science and Technology (MEXT). In collaboration with twelve cooperating institutes, the BBJ has recruited a total of 200,000 people, suffering from at least one of the 47 target common diseases, in the first phase (5-year period). BBJ has collected biospecimens including DNA and serum as well as various clinical and lifestyle information through interview or medical records by using standardized questionnaire. All participants gave written informed consent to this project and this study was approved by ethical committees of RIKEN and participating institutes.

**BES (Beijing Eye Study):** The BES is a population-based study that assessed the associated and risk factors of ocular and general diseases in a Chinese population. The study was initialized in 2001 and collected data from 4439 subjects aged ≥ 40 years and living in seven communities in the Beijing area. Three of these communities were located in rural districts and four were located in urban districts. The BES was followed-up in 2006, with 3251 of the original subjects participating, and in 2011, with 2695 subjects returned returning for the follow-up examination. At the examinations in 2006 and 2011, trained research staffs asked the subjects questions from a standard questionnaire providing information on family status, level of education, income, quality of life, psychic depression, physical activity, and known major systemic diseases. Fasting blood samples were taken for measurement of concentrations of substances such as blood lipids, glucose, and glycosylated hemoglobin. Individuals were classiﬁed as self-reported non-smokers or self-reported current smokers. Alcohol consumption habits based on number of drinks per day were collected. All variables used in analyses were taken from examinations in 2006 or in 2011. The BES subjects were genotyped on two arrays, Illumina Human610-Quad (N = 832) and Illumina OmniExpress (N = 814).

**BRIGHT (British Genetics of Hypertension):** Participants of the BRIGHT Study are recruited from the Medical Research Council General Practice Framework and other primary care practices in the UK. Each case had a history of hypertension diagnosed prior to 60 years of age with confirmed blood pressure recordings corresponding to seated levels > 150/100mmHg (1 reading) or mean of 3 readings > 145/95 mmHg. BRIGHT is focused on recruitment of hypertensive individuals with BMI < 30. Sample selection for GWAS was based on DNA availability and quantity[24].

**CAGE-Amagasaki (Cardio-metabolic Genome Epidemiology Network, Amagasaki Study):** CAGE-Amagasaki is an ongoing population-based cohort study of 5,743 individuals (3,435 males and 2,310 females), aged >18 years and recruited for a baseline examination between September 2002 to August 2003. Participants were interviewed by trained personnel to obtain information on medical and lifestyle variables, and consented to provide DNA for genotyping of molecular variants to investigate genetic susceptibility for so-called lifestyle-related diseases such as hypertension and cardiovascular disorder.

**CoLaus (Cohorte Lausannoise):** The cohort is a random population sample of the city of Lausanne aged 35-75 years. Recruitment began in June 2003 and ended in May 2006, and the first follow-up was conducted between April 2009 and September 2012. The CoLaus study was approved by the Institutional Ethics Committee of the University of Lausanne and informed consent was appropriately obtained by all participants. Both at baseline and follow-up, all participants attended the outpatient clinic of the University Hospital of Lausanne in the morning after an overnight fast. Data were collected by trained field interviewers in a single visit lasting about 60 min.

**DESIR (Data from an Epidemiological Study on the Insulin Resistance):** The DESIR cohort study aims to: describe and understand the relations between the abnormalities of the syndrome, their evolution, according to age and sex; search for risk factors of insulin resistance, in particular factors associated with the environment, lifestyle and genetic markers; quantify the links between the syndrome and both cardiovascular disease and diabetes; evaluate the frequency of the syndrome in terms of its consequences on public health.

**DFTJ (Dongfeng-Tongji Cohort Study):** The DFTJ-cohort study includes 27,009 retired employees from a state-owned automobile enterprise in China. This study was launched in 2008 and will be followed up every 5 years. In 2013 we conducted the first follow-up. By using semi-structural questionnaire and health examination, those having cancer or severe diseases were excluded. Fasting blood samples and detailed epidemiology data were collected. The main goal of the cohort was to identify the environmental and genetic risk factors and the gene-environment interactions on chronic diseases, and to find novel biomarkers for chronic disease and mortality prediction. Finally, 1,461 included in the present study with GWAS data. All of the participants wrote informed consent and the ethical committees in the Tongji Medical College approved this research project. Detailed information has been described in elsewhere [25]. We did the GWAS scan on the DFTJ-cohort with Affymetrix Genome-Wide Human SNP Array 6.0 chips. In total, we genotyped 906,703 SNPs among 1,461 subjects. After stringent QC filtering, SNPs with MAF < 0.01, Hardy-Weinberg Equilibrium (HWE) < 0.0001, and SNP call rate < 95% were excluded. Individuals with call rates < 95% were also not included for further analysis. In total, we retained 1,452 subjects with 658,288 autosomal SNPs for statistical analyses, with an overall call rate of 99.68%. We used MACH 1.0 software to impute untyped SNPs using the LD information from the HapMap phase II database (CHB+JPT as a reference set (2007-08_rel22, released 2007-03-02). Imputed SNPs with high genotype information content (Rsq > 0.3 for MACH) were kept for the further association analysis.

**DHS (Diabetes Heart Study):** DHS is an ongoing family-based cohort study investigating the epidemiology and genetics of cardiovascular disease (CVD) in a population-based sample. The DHS recruited T2D-affected siblings without advanced renal insufficiency from 1998 through 2005 in western North Carolina. DHS has collected genetic data on 1,220 self-described European American (EA) individuals from 475 families. Genotyping was completed using an Affymetrix Genome-Wide Human SNP Array 5.0 with imputation of 1,000 Genomes project SNPs from this array using IMPUTE2 and the Phase I v2, cosmopolitan (integrated) reference panel, build 37.

**DR’s EXTRA (Dose-Responses to Exercise Training):** The DR’s EXTRA study is a 4-year RCT on the effects of regular physical exercise and healthy diet on endothelial function, atherosclerosis and cognition in a randomly selected population sample (n = 3000) of Eastern Finnish men and women, identified from the national population register, aged 55-74 years. Of the eligible sample, 1410 individuals were randomized into one of the 6 groups: aerobic exercise, resistance exercise, diet, combined aerobic exercise and diet, combined resistance exercise and diet, or reference group following baseline assessments. During the four-year intervention the drop-out rate was 15%.

**EGCUT (Estonian Genome Center - University of Tartu (Estonian Biobank)):** EGCUT is the population-based biobank of the Estonian Genome Center at the University of Tartu (www.biobank.ee; EGCUT). The entire project is conducted according to the Estonian Gene Research Act and all the participants have signed the broad informed consent. The cohort size is up to 51535 individuals from 18 years of age and up, which closely reflects the age, sex and geographical distribution of the Estonian population. All the subjects are recruited randomly by general practitioners and physicians in hospitals. A Computer Assisted Personal interview is filled within 1-2 hours at a doctor’s office, which includes personal, genealogical, educational, occupational history and lifestyle data. Anthropometric measurements, blood pressure and resting heart rate are measured, and venous blood taken during the visit. Medical history and current health status is recorded according to ICD-10 codes.

**EPIC (European Prospective Investigation into Cancer and Nutrition)**-Norfolk: EPIC began as a large multi-center cohort study primarily looking at the connection between diet, lifestyle factors and cancer, although the study was broadened from the outset to include other conditions. The EPIC-Norfolk participants are men and women who were aged between 40 and 79 when they joined the study and who lived in Norwich and the surrounding towns and rural areas. They have been contributing information about their diet, lifestyle and health through questionnaires and health checks over two decades. The Norwich Local Research Ethics Committee granted ethical approval for the study. All participants gave written informed consent.

**FENLAND (The Fenland Study):** FENLAND is a population-based cohort study that uses objective measures of disease exposure to investigate the influence of diet, lifestyle and genetic factors on the development of diabetes and obesity. The volunteers are recruited from general practice lists in and around Cambridgeshire (Cambridge, Ely, and Wisbech) in the United Kingdom from birth cohorts from 1950–1975.

**FUSION (Finland-United States Investigation of NIDDM Genetics):** FUSION study is a long-term effort to identify genetic variants that predispose to type 2 diabetes (T2D) or that impact the variability of T2D-related quantitative traits. The FUSION GWAS sample consists of 1,161 Finnish T2D cases and 1,174 Finnish normal glucose-tolerant (NGT) controls [26]. Cases are defined by fasting plasma glucose ≥ 7.0 mmol/l or 2-h plasma glucose ≥ 11.1 mmol/l, by report of diabetes medication use, or based on medical record review. 789 FUSION cases each reported at least one T2D sibling; 372 Finrisk 2002 T2D cases came from a Finnish population-based risk factor survey. NGT controls are defined by fasting glucose < 6.1 mmol/l and 2-h glucose < 7.8 mmol/l. FUSION controls include 119 subjects from Vantaa, Finland who were NGT at ages 65 and 70 years, 304 NGT spouses from FUSION families, and 651 Finrisk 2002 subjects. The controls were approximately frequency matched to the cases by age, sex, and birth province. Smoking and alcohol data are only available in the FUSION subset of our GWAS samples.

**GeneSTAR (Genetic Studies of Atherosclerosis Risk):** GeneSTAR is a family-based prospective study of more than 4000 participants begun in 1983 to determine phenotypic and genetic causes of premature cardiovascular disease. Families were identified from 1983-2006 from probands with a premature coronary disease event prior to 60 years of age who were identified at the time of hospitalization in any of 10 hospitals in the Baltimore, Maryland area. Their apparently healthy 30-59-year-old siblings without known coronary disease were recruited and screened between 1983 and 2006. From 2003-2006, adult offspring over 21 years of age of all participating siblings and probands, as well as the coparents of the offspring were recruited and screened. Genotyping was performed in 3,232 participants on the Illumina 1Mv1_c platform.

**GLACIER (Gene x Lifestyle Interactions and Complex Traits Involved in Elevated Disease Risk):** GLACIER study is nested within the Västerbotten Health Survey, which is part of the Northern Sweden Health and Disease Study, a population-based prospective cohort study from northern Sweden. Participants were genotyped with Illumina CardioMetaboChip array. This array contains ~200,000 variants, the majority being common variants. Systolic and diastolic blood pressures were measured once following a period of five minutes rest with the participant in the supine position using a mercury-gauge sphygmomanometer. Analysis of serum lipids (HDL-C, triglycerides and total cholesterol) were undertaken at the Department of Clinical Chemistry at Umeå University Hospital using routine methods. LDL-C was determined using the Friedewald formula. All participants completed a detailed, optically readable, health and lifestyle questionnaire including questions about smoking status and alcohol intake (FFQ). Cohort description is published[27].

**GRAPHIC (Genetic Regulation of Arterial Pressure of Humans in the Community):** The GRAPHIC study comprises 2024 individuals from 520 nuclear families recruited from the general population in Leicestershire, UK between 2003-2005 for the purpose of investigating the genetic determinants of blood pressure and related cardiovascular traits. A detailed medical history was obtained from study subjects by standardized questionnaires and clinical examination was performed by research nurses following standard procedures. Measurements obtained included height, weight, waist-hip ratio, clinic and ambulatory blood pressure and a 12-lead ECG.

**HCHS/SOL (Hispanic Community Health Study/ Study of Latinos):** The HCHS/SOL is a community-based cohort study of 16,415 self-identified Hispanic/Latino persons aged 18–74 years and selected from households in predefined census-block groups across four US field centers (in Chicago, Miami, the Bronx, and San Diego). The census-block groups were chosen to provide diversity among cohort participants with regard to socioeconomic status and national origin or background. The HCHS/SOL cohort includes participants who self-identified as having a Hispanic/Latino background; the largest groups are Central American (n = 1,730), Cuban (n = 2,348), Dominican (n = 1,460), Mexican (n = 6,471), Puerto Rican (n = 2,728), and South American (n = 1,068). The HCHS/SOL baseline clinical examination occurred between 2008 and 2011 and included comprehensive biological, behavioral, and sociodemographic assessments. Consenting HCHS/SOL subjects were genotyped at Illumina on the HCHS/SOL custom 15041502 B3 array. The custom array comprised the Illumina Omni 2.5M array (HumanOmni2.5-8v.1-1) ancestry-informative markers, known GWAS hits and drug absorption, distribution, metabolism, and excretion (ADME) markers, and additional custom content including ~150,000 SNPs selected from the CLM (Colombian in Medellin, Colombia), MXL (Mexican Ancestry in Los Angeles, California), and PUR (Puerto Rican in Puerto Rico) samples in the 1000Genomes phase 1 data to capture a greater amount of Amerindian genetic variation. QA/QC procedures yielded a total of 12,803 unique study participants for imputation and downstream association analyses.

**HRS (Health & Retirement Study):** HRS is a longitudinal survey of a representative sample of Americans over the age of 50. The current sample is over 26,000 persons in 17,000 households. Respondents are interviewed every two years about income and wealth, health and use of health services, work and retirement, and family connections. DNA was extracted from saliva collected during a face-to-face interview in the respondents' homes. These data represent respondents who provided DNA samples and signed consent forms in 2006, 2008, and 2010. Respondents were removed if they had missing genotype or phenotype data[28]. (<http://hrsonline.isr.umich.edu/sitedocs/userg/dr-011.pdf> )

**HyperGEN-AXIOM (Hypertension Genetic Epidemiology Network):** HyperGEN is a family-based study that investigates the genetic causes of hypertension and related conditions in EA and AA subjects. HyperGEN recruited hypertensive sibships, along with their normotensive adult offspring, and an age-matched random sample. HyperGEN has collected data on 2,471 Caucasian-American subjects and 2,300 African-American subjects, from five field centers in Alabama, Massachusetts, Minnesota, North Carolina, and Utah. HyperGEN participates as a discovery study using GWAS available in a large subset of the samples. The remaining AA subjects without GWAS data were genotyped on the Affymetrix Axiom chip as part of a HyperGEN admixture mapping ancillary study. After excluding subjects already included in the original HyperGEN (or with family members included), this subset of approximately 450 AA subjects are included in the HyperGEN-AXIOM study which participates in replications.

**INGI-CARL** and **INGI-FVG (Italian Network Genetic Isolates):** INGI FVG and INGI CARL studies include samples coming from isolated populations and belong to the ITALIAN NETWORK OF GENETIC ISOLATES (INGI). INGI CARL examined about 1000 subjects between 1998 and 2005 coming from a small village of the South of Italy situated in the extreme northern part of the Puglia Region, while INGI FVG involved about 1700 subjects between 2008 and 2011 coming from six different villages located in the North-East of Italy in the Friuli Venezia Giulia region. A questionnaire was administered to each participant to obtain socio-demographic information, as well as data on professional activity, family history, eating habits and lifestyle, such as smoking, coffee and alcohol consumption, physical activity. Furthermore, a medical screening, including anamnesis, blood pressure, drugs and clinical chemistry evaluation (blood count and different biochemical parameters, such as lipids) were made. All participants gave their written informed consent.

**InterAct (The EPIC-InterAct Case-Cohort Study):** The large prospective InterAct type 2 diabetes case-cohort study is coordinated by the MRC Epidemiology Unit in Cambridge and nested within the European Prospective Investigation into Cancer and Nutrition (EPIC). EPIC was initiated in the late 1980s and involves collaboration between 23 research institutions across Europe in 10 countries (Denmark, France, Germany, Greece, Italy, the Netherlands, Norway, Spain, Sweden and the United Kingdom). Most EPIC cohorts were recruited from the general population, with some exceptions. French cohorts included women who were members of a health insurance scheme for school and university employees; Turin and Ragusa (Italy) and the Spanish centers included some blood donors. Participants from Utrecht (Netherlands) and Florence (Italy) were recruited via a breast cancer screening program. Most participants recruited by the EPIC Oxford (UK) center consisted of vegetarian and “health conscious” volunteers from England, Wales, Scotland, and Northern Ireland.

**IRAS (Insulin Resistance Atherosclerosis Study):** IRAS was an epidemiologic cohort study designed to examine the relationship between insulin resistance and carotid atherosclerosis across a range of glucose tolerance. Individuals of self-reported Mexican-American ethnicity were recruited in San Antonio, TX and San Luis Valley, CO. Recruitment was balanced across age and glucose tolerance status. Inclusion of IRAS data is limited to 194 normoglycemic individuals with genotype data from the Illumina OmniExpress and Omni 1S arrays and imputation to the 1000 Genome Integrated Reference Panel (phase I).

**IRAS Family Study (Insulin Resistance Atherosclerosis Study):** The IRASFS was a family study designed to examine the genetic and epidemiologic basis of glucose homeostasis traits and abdominal adiposity. Briefly, self-reported Mexican pedigrees were recruited in San Antonio, TX and San Luis Valley, CO. Probands with large families were recruited from the initial non-family-based IRAS, which was modestly enriched for impaired glucose tolerance and T2D. Inclusion of IRASFS data is limited to 1040 normoglycemic individuals in 88 pedigrees with genotype data from the Illumina OmniExpress and Omni 1S arrays and imputation to the 1000 Genome Integrated Reference Panel (phase I).

**KORA (Cooperative Health Research in the Augsburg Region):** The KORA study is a series of independent population-based epidemiological surveys of participants living in the region of Augsburg, Southern Germany. All survey participants are residents of German nationality identified through the registration office and were examined in 1994/95 (KORA S3) and 1999/2001 (KORA S4). In the KORA S3 and S4 studies 4,856 and 4,261 subjects have been examined implying response rates of 75% and 67%, respectively. 3,006 subjects participated in a 10-year follow-up examination of S3 in 2004/05 (KORA F3), and 3080 of S4 in 2006/2008 (KORA F4). The age range of the participants was 25 to 74 years at recruitment. Informed consent has been given by all participants. The study has been approved by the local ethics committee. Individuals for genotyping in KORA F3 and KORA F4 were randomly selected and these genotypes are taken for the analysis of the phenotypes in KORA S3 and KORA S4.

**LBC1921 (Lothian Birth Cohort 1921):** LBC1921 consists of 550 (234 male) relatively healthy individuals, assessed on cognitive and medical traits at a mean age of 79.1 years (SD = 0.6). They were born in 1921, most took part in the Scottish Mental Survey of 1932, and almost all lived independently in the Lothian region (Edinburgh City and surrounding area) of Scotland.^1^

**LBC1936 (Lothian Birth Cohort 1936):** LBC1936 consists of 1091 (548 male) relatively healthy individuals who underwent cognitive and medical testing at a mean age of 69.6 years (SD = 0.8). They were born in 1936, most took part in the Scottish Mental Survey of 1947, and almost all lived independently in the Lothian region of Scotland[29].

**LifeLines (Lifelines Cohort Study):** Lifelines (https://lifelines.nl/) is a multi-disciplinary prospective population-based cohort study using a unique three-generation design to examine the health and health-related behaviors of 165,000 persons living in the North East region of The Netherlands. It employs a broad range of investigative procedures in assessing the biomedical, socio-demographic, behavioral, physical and psychological factors which contribute to the health and disease of the general population, with a special focus on multimorbidity. In addition, the LifeLines project comprises a number of cross-sectional sub-studies which investigate specific age-related conditions. These include investigations into metabolic and hormonal diseases, including obesity, cardiovascular and renal diseases, pulmonary diseases and allergy, cognitive function and depression, and musculoskeletal conditions. All survey participants are between 18 and 90 years old at the time of enrollment. Recruitment has been going on since the end of 2006, and over 130,000 participants had been included by April 2013. At the baseline examination, the participants in the study were asked to fill in a questionnaire (on paper or online) before the first visit. During the first and second visit, the first or second part of the questionnaire, respectively, are checked for completeness, a number of investigations are conducted, and blood and urine samples are taken.

**LLFS (The Long Life Family Study):** LLFS is a family-based cohort study, including four clinical centers: Boston University Medical Center in Boston, MA, USA, Columbia College of Physicians and Surgeons in New York City, NY, USA, the University of Pittsburgh in Pittsburgh PA, USA, and University of Southern Denmark, Denmark. The study characteristics, recruitment, eligibility and enrollment have been previously described[30-32]. In brief, the LLFS was designed to determine genetic, behavioral, and environmental factors related to families of exceptionally healthy, elderly individuals. Phase 1 was conducted between 2006 and 2009 recruiting 4,953 individuals from 539 families. The probands were at least 79 years old in the USA centers, and 90 years old or above in Denmark. The families were selected to participate in the study based on The Family Longevity Selection Score (FLoSS)[31], a score generated according to birth-year cohort survival probabilities of the proband and siblings; probands and their families with FLoSS score of 7 or higher, at least one living sibling, and at least one living offspring (minimum family size of 3), who were able to give informed consent and willing to participate were recruited. The individuals were genotyped using ~2.3 million SNVs from the Illumina Omni chip, and then imputed on phased 1000 Genomes with Cosmopolitan data as a reference using MACH and MINIMAC. After excluding participants with 80 years and older, ~3,200 individuals have been included in the analyses for replication.

**LOLIPOP (London Life Sciences Prospective Population Study):** LOLIPOP is a population based prospective study of about 28K Indian Asian and European men and women, recruited from the lists of 58 General Practitioners in West London, United Kingdom between 2003 and 2008. Indian Asians had all four grandparents born on the Indian subcontinent. Europeans were of self-reported white ancestry. At enrolment, all participants completed an interviewer-administered questionnaire for demographic data, medical history, and smoking and alcohol drinking habits. Anthropometric data were collected, and blood pressure measured using an Omron 705CP with the mean of three measurements recorded. Blood samples were collected for the measurement of lipid profile after an overnight fasting of at least 8 hours. Aliquots of whole blood were stored at -80C for extraction of genomic DNA. The LOLIPOP study is approved by the local Research Ethics Committees and all participants provided written informed consent.

**Loyola GxE (Kingston Gene-by-environment; subset of International Collaborative Study of Hypertension in Blacks (ICSHIB)):** The Kingston GxE cohort was obtained from a survey conducted in Kingston, Jamaica as part of a larger project to examine gene by environment interactions in the determination of blood pressure among adults 25-74 years[33]. The principal criterion for eligibility was a body mass index in either the top or bottom third of BMI for the Jamaican population. Participants were identified principally from the records of the Heart Foundation of Jamaica, a non-governmental organization based in Kingston, which provides low-cost screening services (height and weight, blood pressure, glucose, cholesterol) to the general public. Other participants were identified from among participants in family studies of blood pressure at the Tropical Metabolism Research Unit (TMRU) and from among staff members at the University of the West Indies, Mona.

**Loyola SPT (Spanish Town; subset of International Collaborative Study of Hypertension in Blacks (ICSHIB)):** Participants were recruited from Spanish Town, a stable, residential urban area neighboring the capital city of Kingston, Jamaica as part of the ICSHIB[33]. A stratified random sampling scheme was used to recruit adult males and females aged 25–74 years from the general population. Spanish Town was chosen because its demographic make-up was broadly representative of Jamaica as a whole.

**METSIM (Metabolic Syndrome In Men):** The METSIM study includes 10,197 men, aged from 45 to 73 years at recruitment, randomly selected from the population register of the Kuopio town, Eastern Finland, and examined in 2005-2010[34]. The aim of the study is to investigate genetic and non-genetic factors associated with type 2 diabetes and cardiovascular disease and its risk factors.

**NESDA (Netherlands Study of Depression and Anxiety):** NESDA is a multi-center study designed to examine the long-term course and consequences of depressive and anxiety disorders (http://www.nesda.nl)[35]. NESDA included both individuals with depressive and/or anxiety disorders and controls without psychiatric conditions. Inclusion criteria were age 18-65 years and self-reported western European ancestry while exclusion criteria were not being fluent in Dutch and having a primary diagnosis of another psychiatric condition (psychotic disorder, obsessive compulsive disorder, bipolar disorder, or severe substance use disorder).

**OBA (French obese cases):** Study of the genetic of obesity in adults.

**PREVEND (The Prevention of REnal and Vascular ENd stage Disease study):** PREVEND is an ongoing prospective study investigating the natural course of increased levels of urinary albumin excretion and its relation to renal and cardiovascular disease. Inhabitants 28 to 75 years of age (n = 85,421) in the city of Groningen, The Netherlands, were asked to complete a short questionnaire, 47% responded, and individuals were then selected with a urinary albumin concentration of at least 10 mg/L (n = 7,768) and a randomly selected control group with a urinary albumin concentration less than 10 mg/L (n = 3,395). Details of the protocol have been described elsewhere[36].

**PROCARDIS (Precocious Coronary Artery Disease):** The PROCARDIS (European collaborative study of the genetics of precocious CAD) study is a multi-centre case-control study in which CAD cases and controls were recruited from the United Kingdom, Italy, Sweden and Germany. Cases were defined as symptomatic CAD before age 66 years and 80% of cases also had a sibling in whom CAD had been diagnosed before age 66 years. CAD was defined as clinically documented evidence of myocardial infarction (MI) (80%), coronary artery bypass graft (CABG) (10%), acute coronary syndrome (ACS) (6%), coronary angioplasty (CA) (1%) or stable angina (hospitalization for angina or documented obstructive coronary disease) (3%). The cases included 2,136 cases who were half or full siblings. PROCARDIS controls had no personal or sibling history of CAD before age 66 years.

**RHS (Ragama Health Study):** RHS is a population-based study of South Asian men and women aged 35-64yrs living in the Ragama Medical Officer of Health (MOH) area, near Colombo, Sri Lanka[37]. Consenting adults attended a clinic after a 12-h fast with available health records and were interviewed by trained personnel to obtain information on medical, sociodemographic, and lifestyle variables. A 10-mL sample of venous blood was obtained from each subject. The concurrent study was performed in two tea plantation estates in the Lindula MOH area, near Nuwara Eliya (180 km from Colombo), to investigate the gene-environment interaction in a community with differing lifestyles (e.g., physical activity and diet). BP was measured using the Omron 750CP (Omron Co., Japan) in the seated position. The average of two readings was used for the analysis. The RHS is a collaborative effort between the Faculty of Medicine, University of Kelaniya and the National Center for Global Health and Medicine, Japan.

**SHEEP (Stockholm Heart Epidemiology Project):** SHEEP is a population based case-control study of risk factors for first episode of acute myocardial infarction. The study base comprised all Swedish citizens’ resident in the Stockholm county 1992-1994 who were 45-70 years of age and were free of previous clinically diagnosed myocardial infarction. Cases were identified using three different sources: 1) coronary units and internal medicine wards for acute care in all Stockholm hospitals; 2) the National Patient Register; and 3) death certificates. For the present study, only cases who survived at least 28 days were considered (n = 1213). First time incident myocardial infarction cases (n = 1213) were identified during a 2-year period (1992-1993) for men and during a 3-year period (1992-1994) for women. Controls (n = 1561) were randomly recruited from the study population continuously over time within 2 days of the case occurrence and matched to cases on age (5-years interval), sex and hospital catchment area using computerized registers of the population of Stockholm. Five control candidates were sampled simultaneously to be able to replace potential non-respondent controls. Occasionally, because of late response of the initial control, both the first and alternative controls were considered resulting in the inclusion of more controls than cases. Postal questionnaires covering a wide range of exposure areas including occupational exposures, life style factors, social factors and health related factors were distributed to the participants. Clinical investigations were performed at least three months after myocardial infarction of cases and their matched controls. The investigations included blood samplings under fasting conditions with collection of whole blood for DNA extraction, serum and plasma. A biobank was established containing DNA, serum and plasma. Exposure information based on both the questionnaire and biological data from the health examination was available for 78% of the male and 67% of the female non-fatal cases; the corresponding figures for their controls were 68% and 64%.

**SHIP (Study of Health in Pomerania):** SHIP is a prospective longitudinal population-based cohort study in Mecklenburg-West Pomerania assessing the prevalence and incidence of common diseases and their risk factors[38]. SHIP encompasses the two independent cohorts SHIP and SHIP-TREND. Participants aged 20 to 79 with German citizenship and principal residency in the study area were recruited from a random sample of residents living in the three local cities, 12 towns as well as 17 randomly selected smaller towns. Individuals were randomly selected stratified by age and sex in proportion to population size of the city, town or small towns, respectively. A total of 4,308 participants were recruited between 1997 and 2001 in the SHIP cohort. Between 2008 and 2012 a total of 4,420 participants were recruited in the SHIP-TREND cohort. Individuals were invited to the SHIP study centre for a computer-assisted personal interviews and extensive physical examinations. The study protocol was approved by the medical ethics committee of the University of Greifswald. Oral and written informed consents was obtained from each of the study participants. Genome-wide SNP-typing was performed using the Affymetrix Genome-Wide Human SNP Array 6.0 or the Illumina Human Omni 2.5 array (SHIP-TREND samples). Array processing was carried out in accordance with the manufacturer’s standard recommendations. Genotypes were determined using GenomeStudio Genotyping Module v1.0 (GenCall) for SHIP-TREND and the Birdseed2 clustering algorithm for SHIP. Imputation of genotypes in SHIP and SHIP-TREND was performed with the software IMPUTE v2.2.2 based on 1000 Genomes release March 2012.

**SWHS/SMHS (Shanghai Women's Health Study/** **Shanghai Men's Health Study):** SWHS is an ongoing population-based cohort study of approximately 75,000 women who were aged 40-70 years at study enrollment and resided in in urban Shanghai, China; 56,832 (75.8%) provided a blood samples. Recruitment for the SWHS was initiated in 1997 and completed in 2000. The self-administered questionnaire includes information on demographic characteristics, disease and surgery histories, personal habits (such as cigarette smoking, alcohol consumption, tea drinking, and ginseng use), menstrual history, residential history, occupational history, and family history of cancer. The blood pressure was measured by trained interviewers (retired nurses) with a conventional mercury sphygmomanometer according to a standard protocol, after the participants sat quietly for 5 min at the study recruitment. Included in the current project were 2970 women who had GWAS data and blood pressure measurements at the baseline interview. The Shanghai Men’s Health Study (SMHS) is an ongoing population-based cohort study of 61,480 Chinese men who were aged between 40 and 74 years, were free of cancer at enrollment, and lived in urban Shanghai, China; 45,766 (74.4%) provided a blood samples. Recruitment for the SMHS was initiated in 2002 and completed in 2006. The self-administered questionnaire includes information on demographic characteristics, disease and surgery histories, personal habits (such as cigarette smoking, alcohol consumption, tea drinking, and ginseng use), residential history, occupational history, and family history of cancer. The blood pressure was measured by trained interviewers (retired nurses) with a conventional mercury sphygmomanometer according to a standard protocol, after the participants sat quietly for 5 min at the study recruitment. Included in the current project were 892 men who had GWAS data and blood pressure measurements at the baseline interview or 298 men who had GWAS data and lipids data. Genotyping and imputation: Genomic DNA was extracted from buffy coats by using a Qiagen DNA purification kit (Valencia, CA) or Puregene DNA purification kit (Minneapolis, MN) according to the manufacturers’ instructions and then used for genotyping assays. The GWAS genotyping was performed using the Affymetrix Genome-Wide Human SNP Array 6.0 (Affy6.0) platform or Illumina 660, following manufacturers’ protocols. After sample quality control, we exclude SNPs with 1) MAF < 0.01; 2) call rate < 95%; 2) bad genotyping cluster; and 3) concordance rate < 95% among duplicated QC samples. Genotypes were imputed using the program MACH (http://www.sph.umich.edu/csg/abecasis/MACH/download/), which determines the probable distribution of missing genotypes conditional on a set of known haplotypes, while simultaneously estimating the fine-scale recombination map. Phased autosome SNP data from HapMap Phase II Asians (release 22) were used as the reference. To test for associations between the imputed SNP data with BMI, linear regression (additive model) was used, in which SNPs were represented by the expected allele count, an approach that takes into account the degree of uncertainty of genotype imputation (<http://www.sph.umich.edu/csg/abecasis/MACH/download/>).

**TRAILS (Tracking Adolescents’ Individual Lives Survey):** TRAILS is a prospective cohort study of Dutch adolescents with bi or triennial measurements from age 11 to at least age 25 and consists of a general population and a clinical cohort (https://www.trails.nl/en/home). In the population cohort, four assessment waves have been completed to date, which ran from March 2001 to July 2002 (T1), September 2003 to December 2004 (T2), September 2005 to August 2007 (T3), and October 2008 to September 2010 (T4). Data for the present study were collected in the population cohort only, during the third assessment wave.

**TWINGENE (TwinGene of the Swedish Twin Registry):** The aim of the TwinGene project has been to systematically transform the oldest cohorts of the Swedish Twin Registry (STR) into a molecular-genetic resource. Beginning in 2004, about 200 twins were contacted each month until the data collection was completed in 2008. A total of 21,500 twins were contacted where of 12,600 participated. Invitations to the study contained information of the study and its purpose. Along with the invitations consent forms and health questionnaire were sent to the subjects. When the signed consent forms where returned, the subjects were sent blood sampling equipment and asked to contact a local health facility for blood sampling. The study population was recruited among twins participating in the Screening Across the Lifespan Twin Study (SALT) which was a telephone interview study conducted in 1998-2002. Other inclusion criteria were that both twins in the pair had to be alive and living in Sweden. Subjects were excluded from the study if they preciously declined participation in future studies or if they had been enrolled in other STR DNA sampling projects. The subjects were asked to make an appointment for a health check-up at their local health-care facility on the morning Monday to Thursday and not the day before a national holiday, this to ensure that the sample would reach the KI biobank the following morning by over nights mail. The subjects were instructed to fast from 20.00 the previous night. By venipuncture a total of 50 ml of blood was drawn from each subject. Tubes with serum and blood for biobanking as well as for clinical chemistry tests were sent to KI by overnight mail. One 7ml EDTA tube of whole blood is stored in -80°C while a second 7ml EDTA tube of blood is used for DNA extraction using Puregene extraction kit (Gentra systems, Minneapolis, USA). After excluding subjects in which the DNA concentration in the stock-solution was below 20ng/µl as well as subset of 302 female monozygous twin pairs participating in a previous genome wide effort DNA from 9896 individual subjects was sent to SNP&SEQ Technology Platform Uppsala, Sweden for genome wide genotyping with Illumina OmniExpress bead chip (all available dizygous twins + one twin from each available MZ twin pair).

**UKB (United Kingdom Biobank,** www.ukbiobank.ac.uk**):** UKB is a major national health resource with the aim of improving the prevention, diagnosis and treatment of a wide range of serious and life-threatening illnesses. UK Biobank includes data from 502,682 individuals (94% of self-reported European ancestry), with extensive health and lifestyle questionnaire data, physical measures and genetic data. A total of 152,249 participants had genetic and phenotypic (blood pressure) data. Central genotyping quality control (QC) had been performed by UK Biobank [The UK Biobank. UK Biobank Genotyping QC documentation. (2015)]. Further QC was also performed locally.

**References**

1. The Atherosclerosis Risk in Communities (ARIC) Study: design and objectives. The ARIC investigators. Am J Epidemiol. 1989;129(4):687-702. PubMed PMID: 2646917.

2. de Oliveira CM, Pereira AC, de Andrade M, Soler JM, Krieger JE. Heritability of cardiovascular risk factors in a Brazilian population: Baependi Heart Study. BMC Med Genet. 2008;9:32. doi: 10.1186/1471-2350-9-32. PubMed PMID: 18430212; PubMed Central PMCID: PMCPMC2386446.

3. Friedman GD, Cutter GR, Donahue RP, Hughes GH, Hulley SB, Jacobs DR, Jr., et al. CARDIA: study design, recruitment, and some characteristics of the examined subjects. J Clin Epidemiol. 1988;41(11):1105-16. PubMed PMID: 3204420.

4. Fried LP, Borhani NO, Enright P, Furberg CD, Gardin JM, Kronmal RA, et al. The Cardiovascular Health Study: design and rationale. Ann Epidemiol. 1991;1(3):263-76. PubMed PMID: 1669507.

5. Aulchenko YS, Heutink P, Mackay I, Bertoli-Avella AM, Pullen J, Vaessen N, et al. Linkage disequilibrium in young genetically isolated Dutch population. Eur J Hum Genet. 2004;12(7):527-34. doi: 10.1038/sj.ejhg.5201188. PubMed PMID: 15054401.

6. Pardo LM, MacKay I, Oostra B, van Duijn CM, Aulchenko YS. The effect of genetic drift in a young genetically isolated population. Ann Hum Genet. 2005;69(Pt 3):288-95. doi: 10.1046/j.1529-8817.2005.00162.x. PubMed PMID: 15845033.

7. Higgins M, Province M, Heiss G, Eckfeldt J, Ellison RC, Folsom AR, et al. NHLBI Family Heart Study: objectives and design. Am J Epidemiol. 1996;143(12):1219-28. PubMed PMID: 8651220.

8. Investigators F. Multi-center genetic study of hypertension: The Family Blood Pressure Program (FBPP). Hypertension. 2002;39(1):3-9. PubMed PMID: 11799070.

9. Daniels PR, Kardia SL, Hanis CL, Brown CA, Hutchinson R, Boerwinkle E, et al. Familial aggregation of hypertension treatment and control in the Genetic Epidemiology Network of Arteriopathy (GENOA) study. Am J Med. 2004;116(10):676-81. doi: 10.1016/j.amjmed.2003.12.032. PubMed PMID: 15121494.

10. Wyatt SB, Diekelmann N, Henderson F, Andrew ME, Billingsley G, Felder SH, et al. A community-driven model of research participation: the Jackson Heart Study Participant Recruitment and Retention Study. Ethn Dis. 2003;13(4):438-55. PubMed PMID: 14632263.

11. Taylor HA, Jr., Wilson JG, Jones DW, Sarpong DF, Srinivasan A, Garrison RJ, et al. Toward resolution of cardiovascular health disparities in African Americans: design and methods of the Jackson Heart Study. Ethn Dis. 2005;15(4 Suppl 6):S6-4-17. PubMed PMID: 16320381.

12. Fuqua SR, Wyatt SB, Andrew ME, Sarpong DF, Henderson FR, Cunningham MF, et al. Recruiting African-American research participation in the Jackson Heart Study: methods, response rates, and sample description. Ethn Dis. 2005;15(4 Suppl 6):S6-18-29. PubMed PMID: 16317982.

13. Bild DE, Bluemke DA, Burke GL, Detrano R, Diez Roux AV, Folsom AR, et al. Multi-Ethnic Study of Atherosclerosis: objectives and design. Am J Epidemiol. 2002;156(9):871-81. PubMed PMID: 12397006.

14. Victora CG, Barros FC. Cohort profile: the 1982 Pelotas (Brazil) birth cohort study. Int J Epidemiol. 2006;35(2):237-42. doi: 10.1093/ije/dyi290. PubMed PMID: 16373375.

15. Horta BL, Gigante DP, Goncalves H, dos Santos Motta J, Loret de Mola C, Oliveira IO, et al. Cohort Profile Update: The 1982 Pelotas (Brazil) Birth Cohort Study. Int J Epidemiol. 2015;44(2):441, a-e. doi: 10.1093/ije/dyv017. PubMed PMID: 25733577; PubMed Central PMCID: PMCPMC4469796.

16. Ikram MA, Brusselle GGO, Murad SD, van Duijn CM, Franco OH, Goedegebure A, et al. The Rotterdam Study: 2018 update on objectives, design and main results. Eur J Epidemiol. 2017;32(9):807-50. doi: 10.1007/s10654-017-0321-4. PubMed PMID: 29064009; PubMed Central PMCID: PMCPMC5662692.

17. Lavanya R, Jeganathan VS, Zheng Y, Raju P, Cheung N, Tai ES, et al. Methodology of the Singapore Indian Chinese Cohort (SICC) eye study: quantifying ethnic variations in the epidemiology of eye diseases in Asians. Ophthalmic Epidemiol. 2009;16(6):325-36. doi: 10.3109/09286580903144738. PubMed PMID: 19995197.

18. Foong AW, Saw SM, Loo JL, Shen S, Loon SC, Rosman M, et al. Rationale and methodology for a population-based study of eye diseases in Malay people: The Singapore Malay eye study (SiMES). Ophthalmic Epidemiol. 2007;14(1):25-35. doi: 10.1080/09286580600878844. PubMed PMID: 17365815.

19. Nang EE, Khoo CM, Tai ES, Lim SC, Tavintharan S, Wong TY, et al. Is there a clear threshold for fasting plasma glucose that differentiates between those with and without neuropathy and chronic kidney disease?: the Singapore Prospective Study Program. Am J Epidemiol. 2009;169(12):1454-62. doi: 10.1093/aje/kwp076. PubMed PMID: 19406920.

20. Hays J, Hunt JR, Hubbell FA, Anderson GL, Limacher M, Allen C, et al. The Women's Health Initiative recruitment methods and results. Ann Epidemiol. 2003;13(9 Suppl):S18-77. PubMed PMID: 14575939.

21. Design of the Women's Health Initiative clinical trial and observational study. The Women's Health Initiative Study Group. Control Clin Trials. 1998;19(1):61-109. PubMed PMID: 9492970.

22. Hsia J, Margolis KL, Eaton CB, Wenger NK, Allison M, Wu L, et al. Prehypertension and cardiovascular disease risk in the Women's Health Initiative. Circulation. 2007;115(7):855-60. doi: 10.1161/CIRCULATIONAHA.106.656850. PubMed PMID: 17309936.

23. Sever PS, Dahlof B, Poulter NR, Wedel H, Beevers G, Caulfield M, et al. Anglo-Scandinavian Cardiac Outcomes Trial: a brief history, rationale and outline protocol. J Hum Hypertens. 2001;15 Suppl 1:S11-2. PubMed PMID: 11685901.

24. Caulfield M, Munroe P, Pembroke J, Samani N, Dominiczak A, Brown M, et al. Genome-wide mapping of human loci for essential hypertension. Lancet. 2003;361(9375):2118-23. doi: 10.1016/S0140-6736(03)13722-1. PubMed PMID: 12826435.

25. Wang F, Zhu J, Yao P, Li X, He M, Liu Y, et al. Cohort Profile: the Dongfeng-Tongji cohort study of retired workers. Int J Epidemiol. 2013;42(3):731-40. doi: 10.1093/ije/dys053. PubMed PMID: 22531126.

26. Scott LJ, Mohlke KL, Bonnycastle LL, Willer CJ, Li Y, Duren WL, et al. A genome-wide association study of type 2 diabetes in Finns detects multiple susceptibility variants. Science. 2007;316(5829):1341-5. doi: 10.1126/science.1142382. PubMed PMID: 17463248; PubMed Central PMCID: PMCPMC3214617.

27. Kurbasic A, Poveda A, Chen Y, Agren A, Engberg E, Hu FB, et al. Gene-Lifestyle Interactions in Complex Diseases: Design and Description of the GLACIER and VIKING Studies. Curr Nutr Rep. 2014;3(4):400-11. doi: 10.1007/s13668-014-0100-8. PubMed PMID: 25396097; PubMed Central PMCID: PMCPMC4225623.

28. Sonnega A, Faul JD, Ofstedal MB, Langa KM, Phillips JW, Weir DR. Cohort Profile: the Health and Retirement Study (HRS). Int J Epidemiol. 2014;43(2):576-85. doi: 10.1093/ije/dyu067. PubMed PMID: 24671021; PubMed Central PMCID: PMCPMC3997380.

29. Deary IJ, Gow AJ, Pattie A, Starr JM. Cohort profile: the Lothian Birth Cohorts of 1921 and 1936. Int J Epidemiol. 2012;41(6):1576-84. doi: 10.1093/ije/dyr197. PubMed PMID: 22253310.

30. Pedersen CB, Gotzsche H, Moller JO, Mortensen PB. The Danish Civil Registration System. A cohort of eight million persons. Dan Med Bull. 2006;53(4):441-9. PubMed PMID: 17150149.

31. Sebastiani P, Hadley EC, Province M, Christensen K, Rossi W, Perls TT, et al. A family longevity selection score: ranking sibships by their longevity, size, and availability for study. Am J Epidemiol. 2009;170(12):1555-62. doi: 10.1093/aje/kwp309. PubMed PMID: 19910380; PubMed Central PMCID: PMCPMC2800272.

32. Newman AB, Glynn NW, Taylor CA, Sebastiani P, Perls TT, Mayeux R, et al. Health and function of participants in the Long Life Family Study: A comparison with other cohorts. Aging (Albany NY). 2011;3(1):63-76. doi: 10.18632/aging.100242. PubMed PMID: 21258136; PubMed Central PMCID: PMCPMC3047140.

33. Cooper R, Rotimi C, Ataman S, McGee D, Osotimehin B, Kadiri S, et al. The prevalence of hypertension in seven populations of west African origin. Am J Public Health. 1997;87(2):160-8. PubMed PMID: 9103091; PubMed Central PMCID: PMCPMC1380786.

34. Stancakova A, Javorsky M, Kuulasmaa T, Haffner SM, Kuusisto J, Laakso M. Changes in insulin sensitivity and insulin release in relation to glycemia and glucose tolerance in 6,414 Finnish men. Diabetes. 2009;58(5):1212-21. doi: 10.2337/db08-1607. PubMed PMID: 19223598; PubMed Central PMCID: PMCPMC2671053.

35. Penninx BW, Beekman AT, Smit JH, Zitman FG, Nolen WA, Spinhoven P, et al. The Netherlands Study of Depression and Anxiety (NESDA): rationale, objectives and methods. Int J Methods Psychiatr Res. 2008;17(3):121-40. doi: 10.1002/mpr.256. PubMed PMID: 18763692.

36. Hillege HL, Fidler V, Diercks GF, van Gilst WH, de Zeeuw D, van Veldhuisen DJ, et al. Urinary albumin excretion predicts cardiovascular and noncardiovascular mortality in general population. Circulation. 2002;106(14):1777-82. PubMed PMID: 12356629.

37. Dassanayake AS, Kasturiratne A, Rajindrajith S, Kalubowila U, Chakrawarthi S, De Silva AP, et al. Prevalence and risk factors for non-alcoholic fatty liver disease among adults in an urban Sri Lankan population. J Gastroenterol Hepatol. 2009;24(7):1284-8. doi: 10.1111/j.1440-1746.2009.05831.x. PubMed PMID: 19476560.

38. Volzke H, Alte D, Schmidt CO, Radke D, Lorbeer R, Friedrich N, et al. Cohort profile: the study of health in Pomerania. Int J Epidemiol. 2011;40(2):294-307. doi: 10.1093/ije/dyp394. PubMed PMID: 20167617.
