# Supplementary material for: Novel genetic associations for blood pressure identified via gene-alcohol interaction in up to 570K individuals across multiple ancestries
Source: PLoS One. 2018 Jun 18;13(6):e0198166. doi: 10.1371/journal.pone.0198166 (PMC6005576; doi:10.1371/journal.pone.0198166)
Supplement: S2 Note — Information summary of the nearest genes for blood pressure novel loci. (DOCX) [file pone.0198166.s002.docx]

**S2 Note. Summary of biological description for novel BP loci**

Some information of the nearest genes for blood pressure novel loci

**rs73884351** (***LOC105374235-KCNMB2-IT1***, 3q26.32): Two genes reside within ± 500 kb of the lead SNV. The rs73884351 is located within an uncharacterized ***LOC105374235*** and 248 kb downstream of ***KCNMB2-IT1*** (***KCNMB2*** antisense RNA 1, transcript variant 1, long non-coding RNA). ***KCNMB2*** (Potassium Calcium-Activated Channel Subfamily M Regulatory Beta Subunit 2) protein is a regulatory subunit of the calcium activated potassium ***KCNMA1*** (maxiK) channels, which are large conductance, voltage and calcium-sensitive potassium channels that are fundamental to the control of smooth muscle tone and neuronal excitability[1]. The ***KCNMB2*** protein decreases the activation time of MaxiK alpha subunit currents and may participate in ***KCNMA1*** inactivation in chromaffin cells of the adrenal gland or in hippocampal neurons[2, 3]. Studies of ***KCNMB2*** knockout mice have demonstrated a link of hypertension to deficient potassium (K) secretion and aldosteronism[4, 5]. The ***KNCMB2***(-/-) mice had reduced ability to excrete K(+) into the urine but achieved K(+) balance through an aldosterone-mediated, β2-independent mechanism. The ***KNCMB2***(-/-) mice did not display salt-sensitive hypertension and were able to decrease plasma aldosterone on a high-Na(+) diet, although plasma aldosterone remained elevated[5]. GWA studies have found suggestive associations of near or within ***KCNMB2-IT1*** variants with cognitive performance[6], plasma clusterin levels[7], hippocampal sclerosis[8], bipolar disorder and schizophrenia[9], amyotrophic lateral sclerosis[10], and asthma childhood onset[11].

**rs145429126** (***GABRB1***, 4p12): Five genes reside within ± 500 kb of the lead SNV. The rs145429126 is located within an intron of ***GABRB1*** (gamma-aminobutyric acid type A receptor alpha4 subunit). The gamma-aminobutyric acid (GABA) A receptor is a multisubunit chloride channel that acts as inhibitory neurotransmitters in the central nervous system[12]. Most of the genes encoding GABAA receptors are placed in chromosomal clusters, and the GABAA cluster on chromosome 4p12 includes ***GABRB1***, ***GABRA4***, ***GABRA2*** and ***GABRG1***[13]. Alcohol abuse has been associated with facilitation of neurotransmission mediated by the brain’s major inhibitory transmitter, GABA, acting via GABAA receptors[14-16]. Mutations in ***GABRB1*** have been shown to promote alcohol consumption in mice, causing high ethanol consumption accompanied by spontaneous GABA ion channel opening and increased accumbal tonic current[16]. In addition, studies in humans have shown significant allelic association between the risk of alcohol dependence and ***GABRB1*** polymorphisms[14, 15, 17, 18]. ***GABRB1*** has also been associated with bipolar disorder[13], schizophrenia[19], autism spectrum disorder[20], and thalamus volume and their interactive effects on intelligence[21]. GWA studies have shown evidence of association of variants within or near to ***GABRB1*** with age of onset of Alzheimer’s disease[22], or suggestive associations with type 2 diabetes[23], migraine clinic-based[24], and post bronchodilator FEV1[25].

**rs80158983** (***EYS***, 6q12): Two genes reside within ± 500 kb of the lead SNV. The rs80158983 is located within an intron of ***EYS*** (eyes shut homolog, Drosophila). ***EYS*** protein is expressed in the photoreceptor layer of the retina, and the gene is mutated in autosomal recessive retinitis pigmentosa. Analysis of ***EYS*** cDNA has demonstrated that ***EYS*** gene products are expressed with relative abundance in the spinal cord[26]. The structural similarities of these ***EYS*** products to members of the Notch signaling pathway and to agrin suggest a possible functional role in the maintenance and regeneration of the structural integrity of skeletal muscle[26]. Evidence of association of ***PTP4A1-PHF3****-****EYS*** of rare[27] and common[26] variants were reported for alcohol dependence in a multi-racial population study[28]. A GWA study found a suggestive association of ***EYS*** variant with heart failure related metabolomic profile (dihydroxy docosatrienoic acid) in African Ancestry[29] and in European ancestry (serotonin)[30] In addition, GWA studies have reported suggestive associations of variants near or within ***EYS*** with type 2 diabetes[31], statin-induced myopathy[26], and glycosylation of immunoglobulin G[32].

**rs76987554** (***TARID***, 6q23.2): Six genes reside within ± 500 kb of the lead SNV. The rs76987554 is within an intron of ***TARID*** (transcription factor 21). ***TARID*** is a member of the basic helix-loop-helix (bHLH) transcription factor (TF) family and is essential for the development of diverse cell types during embryogenesis of the heart, lung, kidney, and spleen[33-36]. Transcriptional studies in human coronary artery smooth muscle cells demonstrated that ***JUN*** family members and other AP-1 (atypical activator protein 1) -related TFs regulate ***TARID*** transcription, and disruption of ***TARID*** transcription pathway may account in part for coronary artery disease susceptibility[37, 38]. GWA studies have identified variants of ***TARID*** associated with coronary artery disease[39, 40] and coronary heart disease[33, 41]. The lead SNV rs12190287 for these diseases was also identified as an expression quantitative trait locus (eQTL) associated with increased ***TARID*** gene expression in both liver and adipose tissue[34, 41]. The rs12190287 is located within the 3’ untranslated region (3’UTR) of ***TARID***[37]; however rs12190287 and our lead SNV rs76987554 are in low LD (r^2^ = 0.01). In addition, GWA studies have found suggestive associations (1.0x10^-5^<*P*>5.0x10^-8^) near or within variants of ***TARID*** with antihypertensive response to angiotensin II receptor blocker therapy [42], post bronchodilator FEV1[25], schizophrenia[43], age of onset for Alzheimer’s disease[22], developmental language disorder[44], and visceral fat[45]. Another biologically potential candidate gene is ***SGK1***, which encodes serine/threonine-protein kinase that participates in the regulation of renal Na(+) retention, renal K(+) elimination, salt appetite, gastric acid secretion, intestinal Na(+)/H(+) exchange and nutrient transport, insulin-dependent salt sensitivity of blood pressure, salt sensitivity of peripheral glucose uptake, cardiac repolarization and neuroexcitability[46]. Insulin and growth factors, via ***PI3K*** (phosphatidylinositol-3-kinase), ***PDK1*** (3-phosphoinositide-dependent kinase 1), and mTOR (mammalian target of rapamycin), activate ***SGK1***[47] which regulates ***SMCT1/SLC5A8*** (Na+/monocarboxylate transporter 1)[48, 49], ***SGLT1/ SLC5A1*** (Na+-glucose transporter 1)[50], and ***SMIT/SLC5A3*** (myo-inositol cotransporter)[49, 50]. Our lead SNV rs76987554 is located 410 kb downstream of ***SGK1***, which has been associated with increased blood pressure[51-55], obesity and prevalence of type 2 diabetes[47], and stroke[56]. In addition, ***SGK1*** may contribute to the mechanisms underlying behavioral responses to chronic ethanol exposure. A study demonstrated that the hypothalamic pituitary adrenal axis and glucocorticoid receptor signaling mediate acute ethanol induction of Sgk1 transcription in mouse prefrontal cortex[57].

**rs6995407** (***LOC105379224*** (ncRNA)-***SGK223***, 8p23.1): The rs6995407 is located 145 kb upstream of ***SGK223***, which encodes a human pseudokinase and functions as an oncogenic scaffold recruiting a distinct repertoire of signaling proteins[58]. A similar ***SGK223*** protein in rat (Pragmin) binds to Rho family GTPase 2 (Rnd2) and regulates neurite outgrowth via activation of Ras homolog gene family, member A (RhoA)[59]. GWA studies have identified variants near to ***SGK223*** associated with schizophrenia[60] and neuroticism[61].

**rs453301** (***LOC102724880*** (ncRNA)-***PPP1R3B***, 8p23.1): The rs453301 is located 24 kb upstream of ***PPP1R3B***, which encodes the regulatory subunit 3b of protein phosphatase-1 and is expressed in skeletal muscle and liver. ***PPP1R3B*** promotes glycogen synthesis and inhibits glycogen breakdown to glucose 1-phosphate that can be converted to glucose 6-phosphate by phosphoglucomutase[62, 63]. ***PPP1R3B*** regulates protein phosphatase-1 (PP1) catalytic subunit and increases PP1 dephosphorylation of glycogen synthase and phosphorylase kinase[63]. The glycogen synthase is activated by dephosphorylation and the phosphorylase kinase is inactivated by dephosphorylation[62]. ***PPP1R3B*** has been reported to associate with liver enzyme levels (alkaline phosphatase)[64], fasting insulin and glucose interaction with BMI[45], LDL cholesterol, HDL cholesterol and total cholesterol[65-69], C-reactive protein levels[70], glycemic traits (pregnancy)[71], metabolite levels[72], Alzheimer's disease[73], and systemic lupus erythematosus[74].

**rs11774915** (***LOC157273*** (ncRNA), 8p23.1): The rs11774915 is located 29 kb upstream of ***LOC157273***. A GWA study have identified association between ***LOC157273*** with fibrinogen levels[75]. Evidence of association has been observed between ***LOC157273*** with plasma fibrinogen level and incident hypertension among men, but not among women[76, 77]. In addition, moderate drinking was shown to decrease the levels of fibrinogen[78]. and both factors have been suggested to contribute to the protective effect on cardiovascular disease.

**rs55868514** (***TNKS***, 8p23.1): The rs55868514 is located 41 kb upstream of ***TNKS***, which encodes a tankyrase (Tnks). Tnks proteins belong to the superfamily of poly(ADP-ribose) polymerases (PARPs) that catalyze the addition of poly(ADP-ribose) onto substrates, which influence the activity and stability of the modified proteins[79, 80]. ***TNKS*** also regulates the centrosome function[81]. *TNKS* proteins are expressed in a large number of tissues and control a broad range of cellular processes that include DNA damage repair, Wnt signaling, and telomere length maintenance[79, 82]. Deletion of ***TNKS*** gene results in embryonic lethality[83]. Tnks protein binds directly to axin, a negative regulator of the canonical Wnt/β-catenin signaling pathway, forming a destruction complex with glycogen synthase kinase 3β (GSK-3β) and adenomatous polyposis coli (APC) to degrade β-catenin[84]. *TNKS* is also involved in the regulation of ***GLUT4*** (glucose transporter type 4) trafficking in 3T3-L1 adipocytes[85]. Variants of ***TNKS*** are associated with type 2 diabetes[86] and cancers, including gastric cancer[87], breast cancer[88], colon cancer[88], and lung cancer[89]. GWA studies have identified associations between ***TNKS/MSRA*** with obesity limited to children and adolescents[85], neuroticism[61], and osteoarthritis[90].

**rs483916** (***MIR124-1***, 8p23.1): The rs483916 is located 33 kb upstream of ***MIR124-1***, a microRNA 124-1 gene. miRNAs are short non‑coding RNAs that play a key role as post‑transcriptional modulators of gene expression by targeting mRNAs for translational repression or destabilization. miRNAs participate in various biological events and pathological processes[91]. A regulatory network study demonstrated that ***miR-124*** and ***miR-135a*** are potential regulators of the mineralocorticoid receptor gene (***NR3C2***) expression, and could participate in the regulation of renin-angiotensin-aldosterone system and thereby might be involved in BP regulation[92].

**rs11786677** (***MSRA***, 8p23.1): The rs11786677 is located within an intron of ***MSRA***, which encodes a ubiquitous and highly conserved protein, the methionine sulfoxide reductase A. ***MSRA*** catalyzes the enzymatic reduction of methionine sulfoxide to methionine and is implicated in oxidative stress protection, reducing methionine sulfoxide residues in proteins back to methionine[93], thereby repairing and protecting proteins from oxidation. ***MSRA*** is mainly expressed in kidney, liver, brain, and adipose tissue. Chronic excess of ROS leads to mitochondrial dysfunction in liver and skeletal muscle which contribute to insulin resistance[94]. Deletion of *Msra* show high-fat-diet-induced insulin resistance in mice, most likely due to increased oxidative stress[95], while overexpression reduces insulin resistance in old mice[96]. ***MSRA*** may also play a neuroprotective role in Alzheimer’s disease[97, 98]; however the role of methionine sulfoxide reductase in the neurodegenerative diseases is yet to be determined. GWA studies have identified associations of ***MSRA*** with obesity limited to children and adolescents[85], neuroticism[61], schizophrenia[60], chronotype[99], and suggestive association with hypertension[100].

**rs4841409** (***RP1L1***, 8p23.1): The rs4841409 is located 3 kb upstream of ***RP1L1***, which encodes a retinitis pigmentosa 1-like 1 protein that belongs to a member of the doublecortin family. Mutations in the ***RP1L1*** gene cause autosomal dominant occult macular dystrophy [101].The expression of ***RP1L1*** protein is limited to the retina, and it appears to be specific to photoreceptors [102].

**rs7814795** (***MIR4286***, 8p23.1): The rs7814795 is located 5 kb of ***MIR4286*** (Homo sapiens microRNA 4286). A study of microRNA expression revealed that ***MIR4286*** mediates proliferation and apoptosis in melanoma cells[103]. However, the regulatory function of ***MIR4286*** remains unknown.

**rs7814757** (***PINX1***, 8p23.1): The rs7814757 is located within an intron of ***PINX1*** (PIN2/TRF1-interacting telomerase inhibitor 1). ***PINX1*** is involved in preventing telomere degradation and facilitating telomerase-based telomere elongation[104]. Overexpression of ***PINX1*** inhibits telomerase activity, shortens telomeres, and induces crisis, whereas reduction of ***PINX1*** of endogenous PinX1 results in an increase in telomerase activity and elongation of telomeres[105]. ***PINX1*** inhibits cell proliferation and may be a putative tumor suppressor[106]. Studies have suggested that ***PINX1*** participates in the development of cancers[105, 106]. including hepatocellular carcinomas. Overexpression of ***PINX1*** were associated with alcohol-related cirrhosis and fibrosis[107]. ***PINX1*** was reported genome-wide associated with carotid intima-media thickness[108], triglycerides[66], and substance related to lung cancer (3-hydroxy-1-methylpropylmercapturic acid levels) in smokers[109].

**rs4841465** (***XKR6***, 8p23.1): The rs4841465 is located within an intron of ***XKR6*** (XK, Kell blood group complex subunit-related family, member 6). GWA studies have shown evidence of association between ***XKR6*** with triglycerides[110] and eosinophilic esophagitis[66], and suggestive associations with asthma and hay fever[111], systemic lupus erythematosus[112, 113], retinal vascular caliber[114], and response to antipsychotic therapy[115].

XKR6 was found to be

associated with SLE in the SLEGEN GWAS (13). In a further

European case study, evidence of XKR6 susceptibility loci

associated with certain sub-phenotypes of SLE was found,

such as between SNV rs4240671 in XKR6 and lupus nephritis

(P = 0.0006) (14). TMEM39A (rs1132200) was identied as

a susceptibility locus in multiple sclerosis in a comprehen-

sive follow-up of the rst GWAS, which was validated in a

replication study in Spain (15, 16).

**rs9969423** (***FAM167A-AS1***, 8p23.1): The rs9969423 is located within an intron of ***FAM167A-AS1*** (FAM167A antisense RNA 1). A suggestive associations of ***FAM167A-AS1*** with cognitive performance was described in a GWA study[116].

XKR6 was found to be

associated with SLE in the SLEGEN GWAS (13). In a further

European case study, evidence of XKR6 susceptibility loci

associated with certain sub-phenotypes of SLE was found,

such as between SNP rs4240671 in XKR6 and lupus nephritis

(P = 0.0006) (14). TMEM39A (rs1132200) was identied as

a susceptibility locus in multiple sclerosis in a comprehen-

sive follow-up of the rst GWAS, which was validated in a

replication study in Spain (15, 16).

**rs6983727** (***BLK***, 8p23.1): The rs6983727 is located 130 kb downstream of ***BLK***, which encodes a non-receptor tyrosine-kinase of the SRC family of proto-oncogenes that are typically involved in cell proliferation and differentiation. The ***BLK*** protein is present in many tissues, but its expression is highly restricted to the B-cell lineage and is dependent on developmental stage[117]. The ***BLK*** protein stimulates insulin synthesis and secretion in response to glucose and enhances the expression of several pancreatic beta-cell transcription factors. Mutations at the ***BLK*** locus are associated with maturity onset diabetes of the young and β-cell dysfunction[118]. A GWA study identified association of ***BLK-LINC00208*** variants with Barrett's oesophagus or oesophageal adenocarcinoma[119], and the strongest associated SNV rs10108511 (*P* = 2.12 x 10^-9^) is in high LD with our lead SNV rs4841564 for alcohol-BP interaction (r^2^ = 0.91). Evidence of association of alcohol use disorders with an increased risk of both squamous cell carcinoma and adenocarcinoma of the esophagus has been reported in a large population-based study in Sweden[120]. In addition, a suggestive genome-wide association was observed between a variant near to ***BLK-LINC00208*** with alcohol dependence[121]. GWA studies have also shown association between ***BLK*** or near genes with systemic lupus erythematosus[117, 122-124], rheumatoid arthritis[125, 126], and Kawasaki disease[127, 128].

**rs13280442** (***LINC00208***, 8p23.1): The rs13280442 is located 106 kb upstream of ***LINC00208*** (long intergenic non-protein coding RNA 208).

**rs36038176** (***GATA4***, 8p23.1): Twenty genes reside within ± 500 kb of the lead SNV. The rs36038176 is located within intron of ***GATA4***, which encodes a binding protein 4 of zinc-finger transcription factor (TF)[129]. ***GATA4*** regulates genes involved in embryogenesis and myocardial differentiation and function[130] .***GATA4*** also regulates negatively astrocyte cell proliferation and positively apoptosis[131], and is a TF of atrial natriuretic peptide (ANP) associated with the pathophysiology of alcohol dependence[132]. ***GATA4*** rs13273672 has been associated with alcohol dependence in several studies[133-136]. A functional magnetic resonance imaging study in alcohol-dependent patients observed a stronger alcohol-specific amygdala response, which predicted a lowered risk for relapse to heavy drinking in the ***GATA4*** rs13273672 -AA-homozygotes as compared with -G allele carriers[137]. In addition, studies have reported association between ***GATA4*** with idiopathic atrial fibrillation[138] and neurological and psychological disorders[131].

**rs79505281** (***UNC5D***, 8p12): ***UNC5D*** is the only gene that resides within ± 500 kb of the lead SNV. The rs79505281 is located 47 kb downstream of ***UNC5D***, which is a netrin-1 receptor UNC5H family member. ***UNC5D*** is induced during DNA damage-mediated apoptosis and transcriptional target of tumor suppressor p53[139]. Experimental model suggested that Unc5D regulates p53-dependent apoptosis in neuroblastoma cells[140] and in renal cell carcinoma[141]. A GWA study found a suggestive association between variant near ***UNC5D*** with post bronchodilator FEV1/FVC ratio[25].

**rs115888294** (***CDH17***, 8q22.1): Nine genes reside within ± 500 kb of the lead SNV. The rs115888294 is 22 kb downstream of ***CDH17*** (cadherin 17). This gene is a member of the cadherin superfamily, genes encoding calcium-dependent, membrane-associated glycoproteins. ***CDH17*** protein catalyzes the initial reaction in O-linked oligosaccharide biosynthesis, the transfer of an N-acetyl-D-galactosamine residue to a serine or threonine residue on the protein receptor. The protein is a component of the gastrointestinal tract and pancreatic ducts, acting as an intestinal proton-dependent peptide transporter in the first step in oral absorption of many medically important peptide-based drugs[142], and may be involved in the morphological organization of liver and intestine[143]. Association of ***CDH17*** with hypertension-related traits was suggested in a correlated meta-analysis of the African ancestry of the Continental Origin and Genetic Epidemiology Network (COGENT). The cross-phenotype association method was demonstrated to improve statistical power with summary statistics in the combined effects of hypertension, SBP and DBP traits over a single-trait analysis[144]. A GWA study showed evidence of association of ***CDH17*** with diisocyanate-induced asthma[145].

**rs61494734** (***LINGO2***, 9p21.1): Three genes reside within ± 500 kb of the lead SNV. The rs61494734 is located within an intron of ***LINGO2*** (leucine rich repeat and Ig domain containing 2), which has been implicated in essential tremor and Parkinson disease[146, 147], and neurological pathways[148]. A positron emission tomography (PET) study indicated that alcohol-induced suppression of essential tremor patients, which is mediated via a reduction of cerebellar synaptic overactivity, resulted in increased afferent input to the inferior olivary nuclei[149]. Evidence of association between ***LINGO2***-rs12348435 with age at onset of alcohol dependence has been reported in a genome-wide survival analysis in large high-risk families from the Collaborative Study on the Genetics of Alcoholism (COGA)[150]. Also, a suggestive association between ***LINGO2***-rs10968576 with body mass has been described in GIANT Consortium[151] and in a cohort of elderly Swedes[152]. In addition, GWA studies reported significant association of ***LINGO2*** with motion sickness[148], and suggestive associations between variants near or within ***LINGO2*** with pharmacokinetics of olanzapine in severe mental disorder[153], schizophrenia[60], cannabis dependence[154], type 2 diabetes[155], post bronchodilator FEV1/FVC ratio[25], airway responsiveness in COPD[156], and lupus nephritis in systemic lupus erythematosus[157].

**rs73655199** (***CORO2A***, 9q22.33): Eleven genes reside within ± 500 kb of the lead SNV. The rs73655199 is located within an intron of ***CORO2A*** (coronin 2A). Coronins are highly conserved F-actin binding proteins that are important for cell motility, actin dynamics, cell cycle progression, signal transduction, apoptosis, and gene regulation[158]. ***CORO2A*** has been implicated in the regulation of the focal adhesion turnover rate[158], and identified as a component of the nuclear receptor co-repressor (NCoR) complex with a function as an NCoR exchange factor[159]. ***CORO2A*** mediates toll-like receptors (TLRs) -induced NCoR turnover by a mechanism involving interaction with oligomeric nuclear actin[160, 161]. The interaction of ***CORO2A*** via a SIM-motif (small ubiquitin-like modifier (SUMO) 2/3 interacting motif) located in its coiled coil region with SUMOylated liver X receptors (LXRs) prevents NCoR clearance from target gene promoters[160, 161]. ***CORO2A*** expression is associated with colorectal adenoma-adenocarcinoma sequence and oncogenic signaling[160], and may mediate actin-dependent de-repression of inflammatory response genes[161]. Our lead SNV rs73655199 is located 143 kb downstream of ***GABBR2***, which encodes gamma-aminobutyric acid type B receptor subunit 2 that belongs to the G-protein coupled receptor 3 family and GABA-B receptor subfamily. The GABA-B receptors inhibit neuronal activity through G protein-coupled second-messenger systems, which regulate the release of neurotransmitters, and the activity of ion channels and adenylyl cyclase. GABA-B receptors are expressed in human aortic smooth muscle cells and regulate the intracellular Ca(2+) concentration[162]. Evidence of association of ***GABBR2*** variants were described for alcohol dependence[163] and nicotine dependence[164, 165]. In addition, significant reductions were noticed in protein levels of ***GABBR2*** in lateral cerebella from subjects with schizophrenia, bipolar disorder, and major depression when compared with controls[166]. GWA studies have reported significant associations of variants near or within ***CORO2A*** for serum thyroid-stimulating hormone levels[167-169], hypothyroidism[170, 171], thyroid cancer[168, 172-174], severe influenza A (H1N1)[175], and plasma homocysteine[176]. Suggestive genome-wide associations were also detected for coronary heart disease[177], Alzheimer’s disease age of onset[22], schizophrenia[60], and posttraumatic stress disorder[178].

**rs4253197** (***ERCC6***, 10q11.23): Fourteen genes reside within ± 500 kb of the lead SNV. The rs4253197 is located within intron of ***ERCC6*** (excision-repair cross-complementing rodent repair deficiency, complementation group 6), which encodes the Cockayne Syndrome Group B (CSB) protein that participates in DNA repair and gene expression. CSB belongs to the SWI2/SNF2 ATP-dependent chromatin remodeler family, which is conserved from yeast to human[179]. It was indicated that CSB and CTCF (11-zinc finger protein) can regulate each other's chromatin association, and thus modulating chromatin structure and coordinating gene expression in response to oxidative stress [179]. Mutations in ***ERCC6*** are associated with growth failure, intellectual disability, neurological dysfunction and decline[180]. In addition, the pro-carries of ***ERCC6*** 1230Pro allele showed a decreased risk for laryngeal cancer with stronger association in high alcohol consumers, which suggest that ***ERCC6*** may modulate an individual’s ability to repair the effect of alcohol consumption to the risk for laryngeal cancer[181]. Other potential gene influencing alcohol consumption is ***CHAT*** (choline O-acetyltransferase), which encodes an enzyme which catalyzes the biosynthesis of the neurotransmitter acetylcholine. Our lead SNV rs4253197 is located 136 kb upstream of ***CHAT***. A study suggested that adolescent binge ethanol decreases adult ChAT expression, possibly through neuroimmune mechanisms, which might impact adult cognition, arousal, or reward sensitivity[182]. In addition, ***CHAT*** may be involved in the acetylcholine neuronal activity that modulates brain-derived neurotrophic factor production and inflammation in the brain, and in the development of Alzheimer’s disease[183].

**rs201383951** (***GRK5***, 10q26.11): Fourteen genes reside within ± 500 kb of the lead SNV. The rs201383951 is located 13 kb downstream of ***GRK5***, which encodes a member of the guanine nucleotide-binding protein (G protein)-coupled receptor membrane-associated serine/threonine protein kinase. The protein phosphorylates the activated forms of G protein-coupled receptors (GPCRs), which initiates beta-arrestin-mediated receptor desensitization, internalization, and signaling events leading to their down-regulation[184]. Desensitization of GPCRs regulates the number of polymorphonuclear leukocytes (PMNs), which are critical effector cells of the innate immune system and GPCR desensitization is mediated by GRKs[185]. The chemokine macrophage inflammatory protein-2 (***MIP2***) induces ***GRK2*** and ***GRK5*** expression in PMNs through phosphoinositide-3-kinase (PI3K)-gamma signaling. The lipopolysaccharide (LPS)-activated signaling through the Toll-like receptor 4 (***TLR4***) pathway transcriptionally downregulates the expression of ***GRK2*** and ***GRK5*** in response to ***MIP2***. The reduced expression of GRKs lowers chemokine receptor desensitization and augments the PMN migratory response[185]. In addition, it was demonstrated that ***GRK5*** is localized in the centrosome and regulates microtubule nucleation and normal cell cycle progression[186]. Knockdown of ***Grk5*** expression in HeLa cells induced G2/M arrest or delay, which appeared to be due to increased expression of p53, reduced activity of aurora A kinase and a subsequent delay in the activation of polo-like kinase 1[186]. GRKs 2 and 5 are highly expressed in the heart and known to be upregulated in heart failure (HF)[187]. Variant of ***GRK5*** (rs17098707 A>T; Gln41Leu) has revealed association with differential survival in African Americans HF patients[188, 189], but the role of ***GRK5*** in cardiac pathophysiology remains unclear. ***GRK5*** may also have a role in regulating blood pressure through the smooth muscle cells (SMC), which control vascular tone[187, 190]. A study of SMC-specific ***Grk5*** overexpression in mice demonstrated hypertension in a G_i_-dependent manner[191]. However, studies in human have failed to find association of ***GRK5*** with BP variation levels[189, 192]. GWA studies have reported significant association of ***GRK5*** with type 2 diabetes in East Asians[193] and suggestive association with coronary artery aneurysm in Kawasaki disease[194]. Other prominent gene on 10q26.11 for vascular homeostasis is ***BAG3*** (B-cell lymphoma 2-associated athanogene 3). Our lead SNV is located 183 kb upstream of ***BAG3***, which is a member of a conserved family of cyto-protective co-chaperone proteins containing a conserved domain able to interact with heat shock HSC70/HSP70 and sHSPs proteins. Mouse experiment has suggested that ***BAG3*** exerts a vasorelaxing effect through the activation of the PI3K/Akt/eNOS signaling pathway, and may influence blood pressure regulation[195]. A GWA study identified significant association of ***BAG3*** with dilated cardiomyopathy[196], and suggestive association with alcohol dependence[133]. ***BAG3*** was also suggested to contribute to alcohol-induced neurodegeneration[197, 198]. The BAG proteins modulate the switch between autophagy and endoplasmic reticulum (ER)-associated degradation through competing for binding of the adapter proteins, p62 and NBR (neighbor of BRC1). Chronic alcohol intake affects both p62 and ***BAG3***[199-201], which could break the molecular switch and disrupt the balance between the ER and lysosomes for protein degradation[198]. ***BAG3*** is also involved in numerous activities including macro-autophagic protein degradation in aging cells[202]. ***BAG3*** is abundantly expressed in the heart and in striated muscle. Mutations in ***BAG3*** causes severe dominant childhood muscular dystrophy with cardiomyopathy[203]. Our lead SNV is located 258 kb upstream of ***INPP5F***, which encodes an inositol 1,4,5-trisphosphate (InsP3) 5-phosphatase and contains a Sac domain. The activity of this protein is specific for phosphatidylinositol 4,5-bisphosphate and phosphatidylinositol 3,4,5-trisphosphate. A GWA study observed association between ***INPP5F*** (inositol polyphosphate-5-phosphatase F) with Parkinson's disease[155]. Our lead SNV is located 424 kb upstream of ***SEC23IP*** (SEC23 interacting protein), which encodes a member of the phosphatidic acid preferring-phospholipase A1 family that degrades phospholipids and is involved in membrane trafficking[204]. ***SEC23IP*** protein has a role in the organization of ER exit sites and the Golgi apparatus, and in ER Golgi transport[205]. A GWA study found association of ***SEC23IP*** with menarche age at onset[206]. ***SEC23IP*** has been reported to associate with adult attention deficit hyperactivity disorder in exome chip analyses[207] and with neurodevelopmental disorders using exome sequencing in consanguineous families[208].

**rs11200509** (***TACC2***, 10q26.13): Ten genes reside within ± 500 kb of the lead SNV. The rs11200509 is located 2.4 kb downstream of ***TACC2*** (transforming acidic coiled-coil containing protein 2), which encodes a protein that concentrates at centrosomes throughout the cell cycle. TCCA are centrosomes/microtubules interaction-associated proteins containing a highly conserved C-terminal coiled-coil “TACC domain”. ***TACC2*** may promote androgen-mediated growth in the prostate cancer[209] and be involved in the cell proliferation of breast carcinoma[210]. Our lead SNV rs11200509 is located 198 kb and 205 Kb upstream of ***ARMS2*** and ***HTRA1***, respectively. GWA studies have identified several SNVs of ***ARMS2*** and ***HTRA1*** genes associated with age-related macular degeneration[211-218]. Suggestive associations within 1 Mb of the lead SNV were also detected with height[219], type 2 diabetes[220], smoking cessation[221, 222], schizophrenia[60], bipolar disorder[223], late-onset Alzheimer’s disease[224], and breast cancer[225].

**rs10741534** (***GALNT18***, 11p15.4): Seven genes reside within ± 500 kb of the lead SNV. The rs10741534 is 37.5 kb downstream of ***GALNT18*** (polypeptide N-acetylgalactosaminyltransferase 18). ***GALNT18*** protein catalyzes the initial reaction in O-linked oligosaccharide biosynthesis, the transfer of an N-acetyl-D-galactosamine residue to a serine or threonine residue on the protein receptor. GWA studies have suggested associations between variants near or within ***GALNT18*** with type 2 diabetes in the African-American population , post bronchodilator FEV1 [25], psychotic symptoms in prion disease[226], Alzheimer’s disease[22], diisocyanate-induced asthma[145], and response to tocilizumab for the treatment of rheumatoid arthritis[227].

**rs139077481** (***ELMOD1***, 11q22.3): Eight genes reside within ± 500 kb of the lead SNV. The rs139077481 is located within an intron of ***ELMOD1*** (cell engulfment and motility domain containing 1). Its protein acts as a GTPase-activating protein (GAP) toward guanine nucleotide exchange factors like ARL2, ARL3, ARF1 and ARF6, but not for GTPases outside the Arf family[228]. The non-opioid sigma-1 receptor (S1R) is an effector of GAP activity of ELMOD1–3 proteins as its direct binding to either ***ELMOD1*** or ***ELMOD2***[228]. GWA studies have described suggestive associations (6.0 x 10^-8^ ≤ *P* ≤ 9.0 x 10^-6^) within the 1 Mb region of rs139077481 for large artery stroke[40], parental extreme longevity (95 years and older)[229], autism spectrum disorder, attention deficit-hyperactivity disorder, bipolar disorder, major depressive disorder, and schizophrenia (combined)[230], optimism[231], and IgG glycosylation[32].

**rs186331780** (***LOC105369793*** -***FAM19A2***, 12q14.1): One gene reside within ± 500 kb of the lead SNV. The rs186331780 is located 391 kb downstream of ***FAM19A2*** (family with sequence similarity 19 member A2, C-C motif chemokine like). ***FAM19A2*** is a member of the TAFA family of five homologous genes that encode small secreted proteins. TAFA proteins appear distantly related to MIP-1alpha, a member of the CC-chemokine family. TAFA mRNAs are highly expressed in specific brain regions, with little expression in colon, heart, lung, spleen, kidney, and thymus[232]. The biological functions of TAFA family members continues unclear; however, there are some indications that TAFAs may modulate immune responses in the CNS, may represent a novel class of neurokines that acts as regulators of immune nervous cells[233], and may control axonal sprouting following brain injury. A GWA study has identified a significant association of ***FAM19A2*** for modified stumvoll insulin sensitivity index[234]. Other GWA studies have found suggestive associations of ***FAM19A2*** with IgG glycosylation[32], schizoaffective disorder[235], oppositional defiant disorder dimensions in attention-deficit hyperactivity disorder[236], hippocampal volume[237], post bronchodilator FEV1/FVC ratio[25], chronic obstructive pulmonary disease[238], pulmonary function decline[239], and asthma and hay fever[111]. In addition, a genome-wide admixture analysis showed a suggestive association between ***FAM19A2*** (rs348644) with FEV1/FVC among African Americans in the COPDGene Study[240].

**rs187888844** (***LOC105370250*** -***PCDH9***, 13q21.32): One gene resides within ± 500 kb of the lead SNV. The rs187888844 is located 476 kb upstream of ***PCDH9*** (protocadherin 9). ***PCDH9*** protein belongs to a calcium-dependent cell–cell adhesion molecule of the cadherin superfamily. ***PCDH9*** predominantly is expressed in the brain but also in other tissues, and the expression patterns appear to be developmentally regulated[241]. A study of tissue microarrays and immunohistochemistry suggested that ***PCDH9*** might function as a tumor suppressor during cancer development and progression. The ***PCDH9*** expression was decreased in human cerebral glial tumors, and the loss correlated significantly with higher histological grade[242]. GWA studies have found suggestive associations of variants within or near ***PCDH9*** with obesity[243], post bronchodilator FEV1/FVC ratio[25], pulmonary function decline[239], 3-hydroxy-1-methylpropylmercapturic acid levels in smokers[109], symmetrical dimethylarginine levels[244], response to platinum-based chemotherapy[245], sense of smell[246], and schizophrenia[60].

**rs116464496** (***LINC00343***, 13q33.2): Two genes reside within ± 500 kb of the lead SNV. The rs116464496 is located 228 kb upstream of ***LINC00343*** (long intergenic non-protein coding RNA 343) and 444 kb downstream of ***DAOA*** (D-amino acid oxidase activator). ***DAOA*** protein activates d-amino acid oxidase in the brain, which oxidizes d-serine, an important co-agonist for the N-methyl-d-Aspartate receptor[247]. Studies have shown evidence that DAOA may be involved in the pathophysiology of psychotic disorders[248]. GWA studies have described suggestive associations of variants within or near ***DAOA*** with Alzheimer’s disease and age of onset[22], bipolar disorder and schizophrenia[249], left superior temporal gyrus thickness (schizophrenia interaction)[250], subcutaneous adipose tissue[251], visceral fat[45], obesity-related traits[252], smoking initiation[253], adverse response to chemotherapy (neutropenia/leucopenia)[254], and immune response to smallpox (secreted IL-2)[255].

**rs7185735 (*FTO***, 16q12.2): Six genes reside within ± 500 kb of the lead SNV. The rs7185735 is located within an intron in ***FTO*** (alpha-ketoglutarate dependent dioxygenase). The fat-mass and obesity-associated gene, ***FTO***, demethylates nuclear RNA and newly synthesized mRNAs[256]. An epitranscriptomic function study demonstrated that ***FTO*** preferentially demethylates *N*^6^,2′-*O*-dimethyladenosine (m^6^A_m_) rather than *N*^6^-methyladenosine (m^6^A) and reduces the stability of m^6^A_m_ mRNAs. Demethylation of cytoplasmic m^6^A_m_ mRNAs may be induced by stimuli that induce cytosolic translocation of ***FTO***[256]. ***FTO*** has been implicated with enhanced food intake and reduced satiety, and in the regulation of the global metabolic rate, energy expenditure and energy homeostasis, which contribute to the regulation of body size and body fat accumulation[257-262]. ***Fto*** mouse deficiency induces browning of white adipose tissue including enhanced uncoupling protein 1 (***Ucp-1***) expression and mitochondrial uncoupling in adipocytes[258]. The regulatory importance of the ***FTO*** locus in the early adipocyte differentiation with a causal role of a risk-conferring ***FTO*** on adiposity was proposed in a study using epigenomic data, observations from patients and mice, and CRISPR–Cas9 genome editing[261]. This study found that the ***FTO***-intronic rs1421085 disrupts a conserved motif for the ***ARID5B*** repressor leading to derepression of a potent preadipocyte enhancer and a doubling of ***IRX3*** and ***IRX5*** expression during early adipocyte differentiation[261, 262]. These findings suggest a pathway for adipocyte thermogenesis regulation involving ***FTO*** and these other genes in the developmental swing between energy-dissipating beige (brite) fat cells and energy-accumulating white adipocytes, which reduces the thermogenesis and subsequently increases lipid deposition. Variants of ***FTO*** have been associated in diverse ancestries with obesity-related traits factors[260]. T2D susceptibility after adjustment for BMI[257, 263]. long-term incidence of cardiovascular disease and related death independent of traditional risk factors[259], depression and mood[264, 265], Alzheimer’s disease[266], alcohol consumption and alcohol dependency[27, 267, 268]. In addition, frequency of alcohol consumption was suggested to modify the effect of FTO variants on BMI[269]. Inconsistent associations have been reported for ***FTO*** with BP traits. Significant association for ***FTO*** with higher systolic and diastolic blood pressures was found after considering adiposity as a covariate in Mexican children[270], but not in Chinese children[271]. GWA studies have identified SNVs near or within ***FTO*** to be associated with BMI[151, 272-282], weight[282], obesity[282-284], body fat percentage[285], waist circumference[286], adiposity[287], circulating leptin levels[288], type 2 diabetes[31, 220, 289-291], dietary macronutrient intake[292], menarche age at onset[206, 293], triglycerides and HDL-cholesterol[66], vitiligo (non-segmental)[294], breast cancer[295, 296], and melanoma[297].

**rs140520944** (***LOC105372045***-***MIR302F***, 18q12.1): No genes reside within ± 500 kb of the lead SNV. The rs140520944 is located 790 kb upstream of ***MIR302F*** (microRNA 302f). microRNAs (miRNAs) are short (20-24 nt) non-coding RNAs that participate in the gene regulatory networks of several multicellular organisms by regulating a variety of biological processes, such as, cell growth, differentiation, apoptosis and metastasis[298-300]. Evidence suggests that miRNAs are transcribed by RNA polymerase II, the transcripts are capped and polyadenylated, which can be either protein-coding or non-coding[301]. miRNAs can cause mRNA degradation or inhibit protein translation through binding to complementary sequences in the 3′-untranslated region (3′-UTR) of target genes[300, 301]. Reports have suggested that dysregulation of miRNA expression may play a role in a series of human cancers, such as breast cancer, colon cancer, osteosarcoma, lung cancer, melanoma and hepatocellular carcinoma[299, 301, 302]. GWA studies have found suggestive associations between intergenic variants within 1Mb of our lead SNV rs140520944 with LDL peak particle diameter- total fat intake interaction[303], post bronchodilator FEV1[25], cognitive performance[116], and bipolar disorder[304].

**rs142673685** (***LOC105372361***-***THEG5***, 19q12): Five genes reside within ± 500 kb of the lead SNV. The rs142673685 is located 76 kb downstream of ***THEG5*** (testis highly expressed protein 5), and 321 upstream of ***TSHZ3*** (teashirt zinc finger homeobox 3), which encodes a zinc-finger transcription factor involved in developmental processes. ***TSHZ3*** in conjunction with ***APBB1*** (amyloid beta precursor protein binding family B member 1), ***SET*** (SET nuclear oncogene) and histone deacetylase (HDAC1 and DHAC2) factors act as transcriptional repressors, which inhibit the expression of ***CASP4*** (Caspase 4)[305]. ***TSHZ3***-mediated transcription repression involves the recruitment of histone deacetylases HDAC1 and HDAC2. FE65 (the ***APBB1*** protein), simultaneously recruits SET (a component of the inhibitor of acetyl transferase), and that in turn recruits histone deacetylases to produce a powerful gene-silencing complex[306]. ***TSHZ3*** regulates the development of neurons involved in respiratory rhythm and airflow control[306], and is expressed in smooth muscle cell precursors that form the wall of the forming mammalian ureter[307]. GWA studies have described suggestive associations between variants within 1Mb of our lead SNV rs140520944 with post bronchodilator FEV1 in COPD[32], cognitive performance[6], narcolepsy with cataplexy[308], and estradiol levels[309].

Some BP-known genes from European ancestry cohorts[310, 311] extended as significant to other ancestry groups, e.g. the *LSP1-TNNT3* in Asians. The novel findings have been also associated with other traits, from different GWAS.

**References**

1. Zarei MM, Song M, Wilson RJ, Cox N, Colom LV, Knaus HG, et al. Endocytic trafficking signals in KCNMB2 regulate surface expression of a large conductance voltage and Ca(2+)-activated K+ channel. Neuroscience. 2007;147(1):80-9. doi: 10.1016/j.neuroscience.2007.04.019. PubMed PMID: 17521822.

2. Wallner M, Meera P, Toro L. Molecular basis of fast inactivation in voltage and Ca2+-activated K+ channels: a transmembrane beta-subunit homolog. Proc Natl Acad Sci U S A. 1999;96(7):4137-42. PubMed PMID: 10097176; PubMed Central PMCID: PMCPMC22433.

3. Xia XM, Ding JP, Lingle CJ. Molecular basis for the inactivation of Ca2+- and voltage-dependent BK channels in adrenal chromaffin cells and rat insulinoma tumor cells. J Neurosci. 1999;19(13):5255-64. PubMed PMID: 10377337.

4. Larsen CK, Jensen IS, Sorensen MV, de Bruijn PI, Bleich M, Praetorius HA, et al. Hyperaldosteronism after decreased renal K+ excretion in KCNMB2 knockout mice. Am J Physiol Renal Physiol. 2016;310(10):F1035-46. doi: 10.1152/ajprenal.00010.2016. PubMed PMID: 26962098.

5. Grimm PR, Irsik DL, Settles DC, Holtzclaw JD, Sansom SC. Hypertension of Kcnmb1-/- is linked to deficient K secretion and aldosteronism. Proc Natl Acad Sci U S A. 2009;106(28):11800-5. doi: 10.1073/pnas.0904635106. PubMed PMID: 19556540; PubMed Central PMCID: PMCPMC2701967.

6. Need AC, Attix DK, McEvoy JM, Cirulli ET, Linney KL, Hunt P, et al. A genome-wide study of common SNPs and CNVs in cognitive performance in the CANTAB. Hum Mol Genet. 2009;18(23):4650-61. doi: 10.1093/hmg/ddp413. PubMed PMID: 19734545; PubMed Central PMCID: PMCPMC2773267.

7. Deming Y, Xia J, Cai Y, Lord J, Holmans P, Bertelsen S, et al. A potential endophenotype for Alzheimer's disease: cerebrospinal fluid clusterin. Neurobiol Aging. 2016;37:208 e1-9. doi: 10.1016/j.neurobiolaging.2015.09.009. PubMed PMID: 26545630; PubMed Central PMCID: PMCPMC5118651.

8. Beecham GW, Hamilton K, Naj AC, Martin ER, Huentelman M, Myers AJ, et al. Genome-wide association meta-analysis of neuropathologic features of Alzheimer's disease and related dementias. PLoS Genet. 2014;10(9):e1004606. doi: 10.1371/journal.pgen.1004606. PubMed PMID: 25188341; PubMed Central PMCID: PMCPMC4154667.

9. Dube S, Dube P, Hardy JF, Rosenfeld RE. Pyloromyotomy of Ramstedt: experience of a nonspecialized centre. Can J Surg. 1990;33(2):95-6. PubMed PMID: 2268819.

10. Xie T, Deng L, Mei P, Zhou Y, Wang B, Zhang J, et al. Genome-wide association study combining pathway analysis for typical sporadic amyotrophic lateral sclerosis in Chinese Han populations. Neurobiol Aging. 2014;35(7):1778 e9- e23. doi: 10.1016/j.neurobiolaging.2014.01.014. PubMed PMID: 24529757.

11. Ding L, Abebe T, Beyene J, Wilke RA, Goldberg A, Woo JG, et al. Rank-based genome-wide analysis reveals the association of ryanodine receptor-2 gene variants with childhood asthma among human populations. Hum Genomics. 2013;7:16. doi: 10.1186/1479-7364-7-16. PubMed PMID: 23829686; PubMed Central PMCID: PMCPMC3708719.

12. Craddock N, Sklar P. Genetics of bipolar disorder. Lancet. 2013;381(9878):1654-62. doi: 10.1016/S0140-6736(13)60855-7. PubMed PMID: 23663951.

13. Craddock N, Jones L, Jones IR, Kirov G, Green EK, Grozeva D, et al. Strong genetic evidence for a selective influence of GABAA receptors on a component of the bipolar disorder phenotype. Mol Psychiatry. 2010;15(2):146-53. doi: 10.1038/mp.2008.66. PubMed PMID: 19078961; PubMed Central PMCID: PMCPMC3967096.

14. Porjesz B, Almasy L, Edenberg HJ, Wang K, Chorlian DB, Foroud T, et al. Linkage disequilibrium between the beta frequency of the human EEG and a GABAA receptor gene locus. Proc Natl Acad Sci U S A. 2002;99(6):3729-33. doi: 10.1073/pnas.052716399. PubMed PMID: 11891318; PubMed Central PMCID: PMCPMC122592.

15. Parsian A, Zhang ZH. Human chromosomes 11p15 and 4p12 and alcohol dependence: possible association with the GABRB1 gene. Am J Med Genet. 1999;88(5):533-8. PubMed PMID: 10490712.

16. Anstee QM, Knapp S, Maguire EP, Hosie AM, Thomas P, Mortensen M, et al. Mutations in the Gabrb1 gene promote alcohol consumption through increased tonic inhibition. Nat Commun. 2013;4:2816. doi: 10.1038/ncomms3816. PubMed PMID: 24281383; PubMed Central PMCID: PMCPMC3843143.

17. Sun F, Cheng R, Flanders WD, Yang Q, Khoury MJ. Whole genome association studies for genes affecting alcohol dependence. Genet Epidemiol. 1999;17 Suppl 1:S337-42. PubMed PMID: 10597459.

18. Song J, Koller DL, Foroud T, Carr K, Zhao J, Rice J, et al. Association of GABA(A) receptors and alcohol dependence and the effects of genetic imprinting. Am J Med Genet B Neuropsychiatr Genet. 2003;117B(1):39-45. doi: 10.1002/ajmg.b.10022. PubMed PMID: 12555233.

19. Bristow GC, Bostrom JA, Haroutunian V, Sodhi MS. Sex differences in GABAergic gene expression occur in the anterior cingulate cortex in schizophrenia. Schizophr Res. 2015;167(1-3):57-63. doi: 10.1016/j.schres.2015.01.025. PubMed PMID: 25660468; PubMed Central PMCID: PMCPMC4524801.

20. Sesarini CV, Costa L, Granana N, Coto MG, Pallia RC, Argibay PF. Association between GABA(A) receptor subunit polymorphisms and autism spectrum disorder (ASD). Psychiatry Res. 2015;229(1-2):580-2. doi: 10.1016/j.psychres.2015.07.077. PubMed PMID: 26239769.

21. Zhu B, Chen C, Xue G, Lei X, Li J, Moyzis RK, et al. The GABRB1 gene is associated with thalamus volume and modulates the association between thalamus volume and intelligence. Neuroimage. 2014;102 Pt 2:756-63. doi: 10.1016/j.neuroimage.2014.08.048. PubMed PMID: 25192656.

22. Herold C, Hooli BV, Mullin K, Liu T, Roehr JT, Mattheisen M, et al. Family-based association analyses of imputed genotypes reveal genome-wide significant association of Alzheimer's disease with OSBPL6, PTPRG, and PDCL3. Mol Psychiatry. 2016;21(11):1608-12. doi: 10.1038/mp.2015.218. PubMed PMID: 26830138; PubMed Central PMCID: PMCPMC4970971.

23. Al Safar HS, Cordell HJ, Jafer O, Anderson D, Jamieson SE, Fakiola M, et al. A genome-wide search for type 2 diabetes susceptibility genes in an extended Arab family. Ann Hum Genet. 2013;77(6):488-503. doi: 10.1111/ahg.12036. PubMed PMID: 23937595.

24. Anttila V, Winsvold BS, Gormley P, Kurth T, Bettella F, McMahon G, et al. Genome-wide meta-analysis identifies new susceptibility loci for migraine. Nat Genet. 2013;45(8):912-7. doi: 10.1038/ng.2676. PubMed PMID: 23793025; PubMed Central PMCID: PMCPMC4041123.

25. Lutz SM, Cho MH, Young K, Hersh CP, Castaldi PJ, McDonald ML, et al. A genome-wide association study identifies risk loci for spirometric measures among smokers of European and African ancestry. BMC Genet. 2015;16:138. doi: 10.1186/s12863-015-0299-4. PubMed PMID: 26634245; PubMed Central PMCID: PMCPMC4668640.

26. Isackson PJ, Ochs-Balcom HM, Ma C, Harley JB, Peltier W, Tarnopolsky M, et al. Association of common variants in the human eyes shut ortholog (EYS) with statin-induced myopathy: evidence for additional functions of EYS. Muscle Nerve. 2011;44(4):531-8. doi: 10.1002/mus.22115. PubMed PMID: 21826682; PubMed Central PMCID: PMCPMC3175321.

27. Wang L, Liu X, Luo X, Zeng M, Zuo L, Wang KS. Genetic variants in the fat mass- and obesity-associated (FTO) gene are associated with alcohol dependence. J Mol Neurosci. 2013;51(2):416-24. doi: 10.1007/s12031-013-0044-2. PubMed PMID: 23771786.

28. Zuo L, Wang K, Wang G, Pan X, Zhang X, Zhang H, et al. Common PTP4A1-PHF3-EYS variants are specific for alcohol dependence. Am J Addict. 2014;23(4):411-4. doi: 10.1111/j.1521-0391.2013.12115.x. PubMed PMID: 24961364; PubMed Central PMCID: PMCPMC4111256.

29. Park HK, Kim DH, Yun DH, Ban JY. Association between IL10, IL10RA, and IL10RB SNPs and ischemic stroke with hypertension in Korean population. Mol Biol Rep. 2013;40(2):1785-90. doi: 10.1007/s11033-012-2232-5. PubMed PMID: 23096091.

30. Gieger C, Geistlinger L, Altmaier E, Hrabe de Angelis M, Kronenberg F, Meitinger T, et al. Genetics meets metabolomics: a genome-wide association study of metabolite profiles in human serum. PLoS Genet. 2008;4(11):e1000282. doi: 10.1371/journal.pgen.1000282. PubMed PMID: 19043545; PubMed Central PMCID: PMCPMC2581785 products and services in the field of targeted quantitative metabolomics research. The other authors have no competing interests to declare.

31. Imamura M, Takahashi A, Yamauchi T, Hara K, Yasuda K, Grarup N, et al. Genome-wide association studies in the Japanese population identify seven novel loci for type 2 diabetes. Nat Commun. 2016;7:10531. doi: 10.1038/ncomms10531. PubMed PMID: 26818947; PubMed Central PMCID: PMCPMC4738362.

32. Lauc G, Huffman JE, Pucic M, Zgaga L, Adamczyk B, Muzinic A, et al. Loci associated with N-glycosylation of human immunoglobulin G show pleiotropy with autoimmune diseases and haematological cancers. PLoS Genet. 2013;9(1):e1003225. doi: 10.1371/journal.pgen.1003225. PubMed PMID: 23382691; PubMed Central PMCID: PMCPMC3561084.

33. Lu X, Wang L, Chen S, He L, Yang X, Shi Y, et al. Genome-wide association study in Han Chinese identifies four new susceptibility loci for coronary artery disease. Nat Genet. 2012;44(8):890-4. doi: 10.1038/ng.2337. PubMed PMID: 22751097; PubMed Central PMCID: PMCPMC3927410.

34. Zhong H, Beaulaurier J, Lum PY, Molony C, Yang X, Macneil DJ, et al. Liver and adipose expression associated SNPs are enriched for association to type 2 diabetes. PLoS Genet. 2010;6(5):e1000932. doi: 10.1371/journal.pgen.1000932. PubMed PMID: 20463879; PubMed Central PMCID: PMCPMC2865508 and owns stock in that company. A number of the other authors were employees of Merck when the work presented in this manuscript was carried out.

35. Quaggin SE, Vanden Heuvel GB, Igarashi P. Pod-1, a mesoderm-specific basic-helix-loop-helix protein expressed in mesenchymal and glomerular epithelial cells in the developing kidney. Mech Dev. 1998;71(1-2):37-48. PubMed PMID: 9507058.

36. Smith LT, Lin M, Brena RM, Lang JC, Schuller DE, Otterson GA, et al. Epigenetic regulation of the tumor suppressor gene TCF21 on 6q23-q24 in lung and head and neck cancer. Proc Natl Acad Sci U S A. 2006;103(4):982-7. doi: 10.1073/pnas.0510171102. PubMed PMID: 16415157; PubMed Central PMCID: PMCPMC1348006.

37. Miller CL, Anderson DR, Kundu RK, Raiesdana A, Nurnberg ST, Diaz R, et al. Disease-related growth factor and embryonic signaling pathways modulate an enhancer of TCF21 expression at the 6q23.2 coronary heart disease locus. PLoS Genet. 2013;9(7):e1003652. doi: 10.1371/journal.pgen.1003652. PubMed PMID: 23874238; PubMed Central PMCID: PMCPMC3715442.

38. Sazonova O, Zhao Y, Nurnberg S, Miller C, Pjanic M, Castano VG, et al. Characterization of TCF21 Downstream Target Regions Identifies a Transcriptional Network Linking Multiple Independent Coronary Artery Disease Loci. PLoS Genet. 2015;11(5):e1005202. doi: 10.1371/journal.pgen.1005202. PubMed PMID: 26020271; PubMed Central PMCID: PMCPMC4447360.

39. Nikpay M, Goel A, Won HH, Hall LM, Willenborg C, Kanoni S, et al. A comprehensive 1,000 Genomes-based genome-wide association meta-analysis of coronary artery disease. Nat Genet. 2015;47(10):1121-30. doi: 10.1038/ng.3396. PubMed PMID: 26343387; PubMed Central PMCID: PMCPMC4589895.

40. Dichgans M, Malik R, Konig IR, Rosand J, Clarke R, Gretarsdottir S, et al. Shared genetic susceptibility to ischemic stroke and coronary artery disease: a genome-wide analysis of common variants. Stroke. 2014;45(1):24-36. doi: 10.1161/STROKEAHA.113.002707. PubMed PMID: 24262325; PubMed Central PMCID: PMCPMC4112102.

41. Schunkert H, Konig IR, Kathiresan S, Reilly MP, Assimes TL, Holm H, et al. Large-scale association analysis identifies 13 new susceptibility loci for coronary artery disease. Nat Genet. 2011;43(4):333-8. doi: 10.1038/ng.784. PubMed PMID: 21378990; PubMed Central PMCID: PMCPMC3119261.

42. Turner ST, Bailey KR, Schwartz GL, Chapman AB, Chai HS, Boerwinkle E. Genomic association analysis identifies multiple loci influencing antihypertensive response to an angiotensin II receptor blocker. Hypertension. 2012;59(6):1204-11. doi: 10.1161/HYP.0b013e31825b30f8. PubMed PMID: 22566498; PubMed Central PMCID: PMCPMC3530397.

43. Avramopoulos D, Pearce BD, McGrath J, Wolyniec P, Wang R, Eckart N, et al. Infection and inflammation in schizophrenia and bipolar disorder: a genome wide study for interactions with genetic variation. PLoS One. 2015;10(3):e0116696. doi: 10.1371/journal.pone.0116696. PubMed PMID: 25781172; PubMed Central PMCID: PMCPMC4363491.

44. Kornilov SA, Rakhlin N, Koposov R, Lee M, Yrigollen C, Caglayan AO, et al. Genome-Wide Association and Exome Sequencing Study of Language Disorder in an Isolated Population. Pediatrics. 2016;137(4). doi: 10.1542/peds.2015-2469. PubMed PMID: 27016271; PubMed Central PMCID: PMCPMC4811310.

45. Manning AK, Hivert MF, Scott RA, Grimsby JL, Bouatia-Naji N, Chen H, et al. A genome-wide approach accounting for body mass index identifies genetic variants influencing fasting glycemic traits and insulin resistance. Nat Genet. 2012;44(6):659-69. doi: 10.1038/ng.2274. PubMed PMID: 22581228; PubMed Central PMCID: PMCPMC3613127.

46. Lang F, Shumilina E. Regulation of ion channels by the serum- and glucocorticoid-inducible kinase SGK1. FASEB J. 2013;27(1):3-12. doi: 10.1096/fj.12-218230. PubMed PMID: 23012321.

47. Lang F, Pearce D. Regulation of the epithelial Na+ channel by the mTORC2/SGK1 pathway. Nephrol Dial Transplant. 2016;31(2):200-5. doi: 10.1093/ndt/gfv270. PubMed PMID: 26163195.

48. Lopez-Barradas A, Gonzalez-Cid T, Vazquez N, Gavi-Maza M, Reyes-Camacho A, Velazquez-Villegas LA, et al. Insulin and SGK1 reduce the function of Na+/monocarboxylate transporter 1 (SMCT1/SLC5A8). Am J Physiol Cell Physiol. 2016;311(5):C720-C34. doi: 10.1152/ajpcell.00104.2015. PubMed PMID: 27488665; PubMed Central PMCID: PMCPMC5130587.

49. Dieter M, Palmada M, Rajamanickam J, Aydin A, Busjahn A, Boehmer C, et al. Regulation of glucose transporter SGLT1 by ubiquitin ligase Nedd4-2 and kinases SGK1, SGK3, and PKB. Obes Res. 2004;12(5):862-70. doi: 10.1038/oby.2004.104. PubMed PMID: 15166308.

50. Shojaiefard M, Strutz-Seebohm N, Tavare JM, Seebohm G, Lang F. Regulation of the Na(+), glucose cotransporter by PIKfyve and the serum and glucocorticoid inducible kinase SGK1. Biochem Biophys Res Commun. 2007;359(4):843-7. doi: 10.1016/j.bbrc.2007.05.111. PubMed PMID: 17570343.

51. Chu C, Wang Y, Wang M, Mu JJ, Liu FQ, Wang L, et al. Common Variants in Serum/Glucocorticoid Regulated Kinase 1 (SGK1) and Blood Pressure Responses to Dietary Sodium or Potassium Interventions: A family-Based Association Study. Kidney Blood Press Res. 2015;40(4):424-34. doi: 10.1159/000368518. PubMed PMID: 26277930.

52. Li C, Yang X, He J, Hixson JE, Gu D, Rao DC, et al. A gene-based analysis of variants in the serum/glucocorticoid regulated kinase (SGK) genes with blood pressure responses to sodium intake: the GenSalt Study. PLoS One. 2014;9(5):e98432. doi: 10.1371/journal.pone.0098432. PubMed PMID: 24878720; PubMed Central PMCID: PMCPMC4039502.

53. Rao AD, Sun B, Saxena A, Hopkins PN, Jeunemaitre X, Brown NJ, et al. Polymorphisms in the serum- and glucocorticoid-inducible kinase 1 gene are associated with blood pressure and renin response to dietary salt intake. J Hum Hypertens. 2013;27(3):176-80. doi: 10.1038/jhh.2012.22. PubMed PMID: 22648267; PubMed Central PMCID: PMCPMC3463709.

54. Busjahn A, Aydin A, Uhlmann R, Krasko C, Bahring S, Szelestei T, et al. Serum- and glucocorticoid-regulated kinase (SGK1) gene and blood pressure. Hypertension. 2002;40(3):256-60. PubMed PMID: 12215463.

55. von Wowern F, Berglund G, Carlson J, Mansson H, Hedblad B, Melander O. Genetic variance of SGK-1 is associated with blood pressure, blood pressure change over time and strength of the insulin-diastolic blood pressure relationship. Kidney Int. 2005;68(5):2164-72. doi: 10.1111/j.1523-1755.2005.00672.x. PubMed PMID: 16221215.

56. Dahlberg J, Smith G, Norrving B, Nilsson P, Hedblad B, Engstrom G, et al. Genetic variants in serum and glucocortocoid regulated kinase 1, a regulator of the epithelial sodium channel, are associated with ischaemic stroke. J Hypertens. 2011;29(5):884-9. doi: 10.1097/HJH.0b013e3283455117. PubMed PMID: 21430556.

57. Costin BN, Dever SM, Miles MF. Ethanol regulation of serum glucocorticoid kinase 1 expression in DBA2/J mouse prefrontal cortex. PLoS One. 2013;8(8):e72979. doi: 10.1371/journal.pone.0072979. PubMed PMID: 23991167; PubMed Central PMCID: PMCPMC3750005.

58. Liu L, Phua YW, Lee RS, Ma X, Jenkins Y, Novy K, et al. Homo- and Heterotypic Association Regulates Signaling by the SgK269/PEAK1 and SgK223 Pseudokinases. J Biol Chem. 2016;291(41):21571-83. doi: 10.1074/jbc.M116.748897. PubMed PMID: 27531744; PubMed Central PMCID: PMCPMC5076828.

59. Tanaka H, Katoh H, Negishi M. Pragmin, a novel effector of Rnd2 GTPase, stimulates RhoA activity. J Biol Chem. 2006;281(15):10355-64. doi: 10.1074/jbc.M511314200. PubMed PMID: 16481321.

60. Goes FS, McGrath J, Avramopoulos D, Wolyniec P, Pirooznia M, Ruczinski I, et al. Genome-wide association study of schizophrenia in Ashkenazi Jews. Am J Med Genet B Neuropsychiatr Genet. 2015;168(8):649-59. doi: 10.1002/ajmg.b.32349. PubMed PMID: 26198764.

61. Smith DJ, Escott-Price V, Davies G, Bailey ME, Colodro-Conde L, Ward J, et al. Genome-wide analysis of over 106 000 individuals identifies 9 neuroticism-associated loci. Mol Psychiatry. 2016;21(6):749-57. doi: 10.1038/mp.2016.49. PubMed PMID: 27067015; PubMed Central PMCID: PMCPMC4879189.

62. Buchbinder JL, Rath VL, Fletterick RJ. Structural relationships among regulated and unregulated phosphorylases. Annu Rev Biophys Biomol Struct. 2001;30:191-209. doi: 10.1146/annurev.biophys.30.1.191. PubMed PMID: 11340058.

63. Gasa R, Jensen PB, Berman HK, Brady MJ, DePaoli-Roach AA, Newgard CB. Distinctive regulatory and metabolic properties of glycogen-targeting subunits of protein phosphatase-1 (PTG, GL, GM/RGl) expressed in hepatocytes. J Biol Chem. 2000;275(34):26396-403. doi: 10.1074/jbc.M002427200. PubMed PMID: 10862764.

64. Chambers JC, Zhang W, Sehmi J, Li X, Wass MN, Van der Harst P, et al. Genome-wide association study identifies loci influencing concentrations of liver enzymes in plasma. Nat Genet. 2011;43(11):1131-8. doi: 10.1038/ng.970. PubMed PMID: 22001757; PubMed Central PMCID: PMCPMC3482372.

65. Surakka I, Horikoshi M, Magi R, Sarin AP, Mahajan A, Lagou V, et al. The impact of low-frequency and rare variants on lipid levels. Nat Genet. 2015;47(6):589-97. doi: 10.1038/ng.3300. PubMed PMID: 25961943; PubMed Central PMCID: PMCPMC4757735.

66. Global Lipids Genetics C, Willer CJ, Schmidt EM, Sengupta S, Peloso GM, Gustafsson S, et al. Discovery and refinement of loci associated with lipid levels. Nat Genet. 2013;45(11):1274-83. doi: 10.1038/ng.2797. PubMed PMID: 24097068; PubMed Central PMCID: PMCPMC3838666.

67. Lettre G, Palmer CD, Young T, Ejebe KG, Allayee H, Benjamin EJ, et al. Genome-wide association study of coronary heart disease and its risk factors in 8,090 African Americans: the NHLBI CARe Project. PLoS Genet. 2011;7(2):e1001300. doi: 10.1371/journal.pgen.1001300. PubMed PMID: 21347282; PubMed Central PMCID: PMCPMC3037413 Genetics, SLH reports being listed as co-inventor on pending and issued patents held by the Cleveland Clinic relating to cardiovascular diagnostics. SLH reports having been paid as a consultant or speaker for the following companies: AstraZeneca Pharmaceuticals LP, BG Medicine, Merck & Co., Pfizer Takeda, Esperion, and Cleveland Heart Lab. SLH reports receiving research funds from Abbott, Liposcience, and Cleveland Heart Lab. WHWT reports receiving research grant support from Abbott Laboratories.

68. Waterworth DM, Ricketts SL, Song K, Chen L, Zhao JH, Ripatti S, et al. Genetic variants influencing circulating lipid levels and risk of coronary artery disease. Arterioscler Thromb Vasc Biol. 2010;30(11):2264-76. doi: 10.1161/ATVBAHA.109.201020. PubMed PMID: 20864672; PubMed Central PMCID: PMCPMC3891568.

69. Coram MA, Duan Q, Hoffmann TJ, Thornton T, Knowles JW, Johnson NA, et al. Genome-wide characterization of shared and distinct genetic components that influence blood lipid levels in ethnically diverse human populations. Am J Hum Genet. 2013;92(6):904-16. doi: 10.1016/j.ajhg.2013.04.025. PubMed PMID: 23726366; PubMed Central PMCID: PMCPMC3675231.

70. Dehghan A, Dupuis J, Barbalic M, Bis JC, Eiriksdottir G, Lu C, et al. Meta-analysis of genome-wide association studies in >80 000 subjects identifies multiple loci for C-reactive protein levels. Circulation. 2011;123(7):731-8. doi: 10.1161/CIRCULATIONAHA.110.948570. PubMed PMID: 21300955; PubMed Central PMCID: PMCPMC3147232.

71. Hays J, Hunt JR, Hubbell FA, Anderson GL, Limacher M, Allen C, et al. The Women's Health Initiative recruitment methods and results. Ann Epidemiol. 2003;13(9 Suppl):S18-77. PubMed PMID: 14575939.

72. Inouye M, Ripatti S, Kettunen J, Lyytikainen LP, Oksala N, Laurila PP, et al. Novel Loci for metabolic networks and multi-tissue expression studies reveal genes for atherosclerosis. PLoS Genet. 2012;8(8):e1002907. doi: 10.1371/journal.pgen.1002907. PubMed PMID: 22916037; PubMed Central PMCID: PMCPMC3420921.

73. Kamboh MI, Demirci FY, Wang X, Minster RL, Carrasquillo MM, Pankratz VS, et al. Genome-wide association study of Alzheimer's disease. Transl Psychiatry. 2012;2:e117. doi: 10.1038/tp.2012.45. PubMed PMID: 22832961; PubMed Central PMCID: PMCPMC3365264.

74. Alarcon-Riquelme ME, Ziegler JT, Molineros J, Howard TD, Moreno-Estrada A, Sanchez-Rodriguez E, et al. Genome-Wide Association Study in an Amerindian Ancestry Population Reveals Novel Systemic Lupus Erythematosus Risk Loci and the Role of European Admixture. Arthritis Rheumatol. 2016;68(4):932-43. doi: 10.1002/art.39504. PubMed PMID: 26606652; PubMed Central PMCID: PMCPMC4829354.

75. de Vries PS, Chasman DI, Sabater-Lleal M, Chen MH, Huffman JE, Steri M, et al. A meta-analysis of 120 246 individuals identifies 18 new loci for fibrinogen concentration. Hum Mol Genet. 2016;25(2):358-70. doi: 10.1093/hmg/ddv454. PubMed PMID: 26561523; PubMed Central PMCID: PMCPMC4715256.

76. Shankar A, Wang JJ, Rochtchina E, Mitchell P. Positive association between plasma fibrinogen level and incident hypertension among men: population-based cohort study. Hypertension. 2006;48(6):1043-9. doi: 10.1161/01.HYP.0000245700.13817.3c. PubMed PMID: 17000922.

77. Folsom AR, Peacock JM, Nieto FJ, Rosamond WD, Eigenbrodt ML, Davis CE, et al. Plasma fibrinogen and incident hypertension in the Atherosclerosis Risk in Communities (ARIC) Study. J Hypertens. 1998;16(11):1579-83. PubMed PMID: 9856357.

78. Mennen LI, Balkau B, Vol S, Caces E, Eschwege E. Fibrinogen: a possible link between alcohol consumption and cardiovascular disease? DESIR Study Group. Arterioscler Thromb Vasc Biol. 1999;19(4):887-92. PubMed PMID: 10195914.

79. Riffell JL, Lord CJ, Ashworth A. Tankyrase-targeted therapeutics: expanding opportunities in the PARP family. Nat Rev Drug Discov. 2012;11(12):923-36. doi: 10.1038/nrd3868. PubMed PMID: 23197039.

80. Lehtio L, Chi NW, Krauss S. Tankyrases as drug targets. FEBS J. 2013;280(15):3576-93. doi: 10.1111/febs.12320. PubMed PMID: 23648170.

81. Kim MK, Dudognon C, Smith S. Tankyrase 1 regulates centrosome function by controlling CPAP stability. EMBO Rep. 2012;13(8):724-32. doi: 10.1038/embor.2012.86. PubMed PMID: 22699936; PubMed Central PMCID: PMCPMC3410387.

82. Kulak O, Chen H, Holohan B, Wu X, He H, Borek D, et al. Disruption of Wnt/beta-Catenin Signaling and Telomeric Shortening Are Inextricable Consequences of Tankyrase Inhibition in Human Cells. Mol Cell Biol. 2015;35(14):2425-35. doi: 10.1128/MCB.00392-15. PubMed PMID: 25939383; PubMed Central PMCID: PMCPMC4475917.

83. Chiang YJ, Hsiao SJ, Yver D, Cushman SW, Tessarollo L, Smith S, et al. Tankyrase 1 and tankyrase 2 are essential but redundant for mouse embryonic development. PLoS One. 2008;3(7):e2639. doi: 10.1371/journal.pone.0002639. PubMed PMID: 18612384; PubMed Central PMCID: PMCPMC2441437.

84. Huang SM, Mishina YM, Liu S, Cheung A, Stegmeier F, Michaud GA, et al. Tankyrase inhibition stabilizes axin and antagonizes Wnt signalling. Nature. 2009;461(7264):614-20. doi: 10.1038/nature08356. PubMed PMID: 19759537.

85. Scherag A, Dina C, Hinney A, Vatin V, Scherag S, Vogel CI, et al. Two new Loci for body-weight regulation identified in a joint analysis of genome-wide association studies for early-onset extreme obesity in French and german study groups. PLoS Genet. 2010;6(4):e1000916. doi: 10.1371/journal.pgen.1000916. PubMed PMID: 20421936; PubMed Central PMCID: PMCPMC2858696.

86. Zee RY, Ridker PM, Chasman DI. Genetic variants of 11 telomere-pathway gene loci and the risk of incident type 2 diabetes mellitus: the Women's Genome Health Study. Atherosclerosis. 2011;218(1):144-6. doi: 10.1016/j.atherosclerosis.2011.05.013. PubMed PMID: 21665207; PubMed Central PMCID: PMCPMC3175791.

87. Gao J, Zhang J, Long Y, Tian Y, Lu X. Expression of tankyrase 1 in gastric cancer and its correlation with telomerase activity. Pathol Oncol Res. 2011;17(3):685-90. doi: 10.1007/s12253-011-9369-8. PubMed PMID: 21455637.

88. Pellatt AJ, Wolff RK, Torres-Mejia G, John EM, Herrick JS, Lundgreen A, et al. Telomere length, telomere-related genes, and breast cancer risk: the breast cancer health disparities study. Genes Chromosomes Cancer. 2013;52(7):595-609. doi: 10.1002/gcc.22056. PubMed PMID: 23629941; PubMed Central PMCID: PMCPMC3807250.

89. Busch AM, Johnson KC, Stan RV, Sanglikar A, Ahmed Y, Dmitrovsky E, et al. Evidence for tankyrases as antineoplastic targets in lung cancer. BMC Cancer. 2013;13:211. doi: 10.1186/1471-2407-13-211. PubMed PMID: 23621985; PubMed Central PMCID: PMCPMC3644501.

90. Moon S, Keam B, Hwang MY, Lee Y, Park S, Oh JH, et al. A genome-wide association study of copy-number variation identifies putative loci associated with osteoarthritis in Koreans. BMC Musculoskelet Disord. 2015;16:76. doi: 10.1186/s12891-015-0531-4. PubMed PMID: 25880085; PubMed Central PMCID: PMCPMC4395893.

91. Bartel DP. MicroRNAs: genomics, biogenesis, mechanism, and function. Cell. 2004;116(2):281-97. PubMed PMID: 14744438.

92. Sober S, Laan M, Annilo T. MicroRNAs miR-124 and miR-135a are potential regulators of the mineralocorticoid receptor gene (NR3C2) expression. Biochem Biophys Res Commun. 2010;391(1):727-32. doi: 10.1016/j.bbrc.2009.11.128. PubMed PMID: 19944075; PubMed Central PMCID: PMCPMC2806518.

93. Lim JC, You Z, Kim G, Levine RL. Methionine sulfoxide reductase A is a stereospecific methionine oxidase. Proc Natl Acad Sci U S A. 2011;108(26):10472-7. doi: 10.1073/pnas.1101275108. PubMed PMID: 21670260; PubMed Central PMCID: PMCPMC3127874.

94. de Ferranti S, Mozaffarian D. The perfect storm: obesity, adipocyte dysfunction, and metabolic consequences. Clin Chem. 2008;54(6):945-55. doi: 10.1373/clinchem.2007.100156. PubMed PMID: 18436717.

95. Styskal J, Nwagwu FA, Watkins YN, Liang H, Richardson A, Musi N, et al. Methionine sulfoxide reductase A affects insulin resistance by protecting insulin receptor function. Free Radic Biol Med. 2013;56:123-32. doi: 10.1016/j.freeradbiomed.2012.10.544. PubMed PMID: 23089224; PubMed Central PMCID: PMCPMC3578155.

96. Salmon AB, Kim G, Liu C, Wren JD, Georgescu C, Richardson A, et al. Effects of transgenic methionine sulfoxide reductase A (MsrA) expression on lifespan and age-dependent changes in metabolic function in mice. Redox Biol. 2016;10:251-6. doi: 10.1016/j.redox.2016.10.012. PubMed PMID: 27821326; PubMed Central PMCID: PMCPMC5099276.

97. Moskovitz J, Du F, Bowman CF, Yan SS. Methionine sulfoxide reductase A affects beta-amyloid solubility and mitochondrial function in a mouse model of Alzheimer's disease. Am J Physiol Endocrinol Metab. 2016;310(6):E388-93. doi: 10.1152/ajpendo.00453.2015. PubMed PMID: 26786779; PubMed Central PMCID: PMCPMC4796266.

98. Moskovitz J, Maiti P, Lopes DH, Oien DB, Attar A, Liu T, et al. Induction of methionine-sulfoxide reductases protects neurons from amyloid beta-protein insults in vitro and in vivo. Biochemistry. 2011;50(49):10687-97. doi: 10.1021/bi201426b. PubMed PMID: 22059533; PubMed Central PMCID: PMCPMC3235361.

99. Hu Y, Shmygelska A, Tran D, Eriksson N, Tung JY, Hinds DA. GWAS of 89,283 individuals identifies genetic variants associated with self-reporting of being a morning person. Nat Commun. 2016;7:10448. doi: 10.1038/ncomms10448. PubMed PMID: 26835600; PubMed Central PMCID: PMCPMC4740817.

100. Levy D, Ehret GB, Rice K, Verwoert GC, Launer LJ, Dehghan A, et al. Genome-wide association study of blood pressure and hypertension. Nat Genet. 2009;41(6):677-87. doi: 10.1038/ng.384. PubMed PMID: 19430479; PubMed Central PMCID: PMCPMC2998712.

101. Kikuchi S, Kameya S, Gocho K, El Shamieh S, Akeo K, Sugawara Y, et al. Cone dystrophy in patient with homozygous RP1L1 mutation. Biomed Res Int. 2015;2015:545243. doi: 10.1155/2015/545243. PubMed PMID: 25692141; PubMed Central PMCID: PMCPMC4322316.

102. Conte I, Lestingi M, den Hollander A, Alfano G, Ziviello C, Pugliese M, et al. Identification and characterisation of the retinitis pigmentosa 1-like1 gene (RP1L1): a novel candidate for retinal degenerations. Eur J Hum Genet. 2003;11(2):155-62. doi: 10.1038/sj.ejhg.5200942. PubMed PMID: 12634863.

103. Komina A, Palkina N, Aksenenko M, Tsyrenzhapova S, Ruksha T. Antiproliferative and Pro-Apoptotic Effects of MiR-4286 Inhibition in Melanoma Cells. PLoS One. 2016;11(12):e0168229. doi: 10.1371/journal.pone.0168229. PubMed PMID: 28005927; PubMed Central PMCID: PMCPMC5179095.

104. Gilson E, Geli V. How telomeres are replicated. Nat Rev Mol Cell Biol. 2007;8(10):825-38. doi: 10.1038/nrm2259. PubMed PMID: 17885666.

105. Zhou XZ, Lu KP. The Pin2/TRF1-interacting protein PinX1 is a potent telomerase inhibitor. Cell. 2001;107(3):347-59. PubMed PMID: 11701125.

106. Li HL, Song J, Yong HM, Hou PF, Chen YS, Song WB, et al. PinX1: structure, regulation and its functions in cancer. Oncotarget. 2016;7(40):66267-75. doi: 10.18632/oncotarget.11411. PubMed PMID: 27556185; PubMed Central PMCID: PMCPMC5323232.

107. El Idrissi M, Hervieu V, Merle P, Mortreux F, Wattel E. Cause-specific telomere factors deregulation in hepatocellular carcinoma. J Exp Clin Cancer Res. 2013;32:64. doi: 10.1186/1756-9966-32-64. PubMed PMID: 24020493; PubMed Central PMCID: PMCPMC3850108.

108. Bis JC, Kavousi M, Franceschini N, Isaacs A, Abecasis GR, Schminke U, et al. Meta-analysis of genome-wide association studies from the CHARGE consortium identifies common variants associated with carotid intima media thickness and plaque. Nat Genet. 2011;43(10):940-7. doi: 10.1038/ng.920. PubMed PMID: 21909108; PubMed Central PMCID: PMCPMC3257519.

109. Park SL, Carmella SG, Chen M, Patel Y, Stram DO, Haiman CA, et al. Mercapturic Acids Derived from the Toxicants Acrolein and Crotonaldehyde in the Urine of Cigarette Smokers from Five Ethnic Groups with Differing Risks for Lung Cancer. PLoS One. 2015;10(6):e0124841. doi: 10.1371/journal.pone.0124841. PubMed PMID: 26053186; PubMed Central PMCID: PMCPMC4460074.

110. Kathiresan S, Willer CJ, Peloso GM, Demissie S, Musunuru K, Schadt EE, et al. Common variants at 30 loci contribute to polygenic dyslipidemia. Nat Genet. 2009;41(1):56-65. doi: 10.1038/ng.291. PubMed PMID: 19060906; PubMed Central PMCID: PMCPMC2881676.

111. Ferreira MA, Matheson MC, Tang CS, Granell R, Ang W, Hui J, et al. Genome-wide association analysis identifies 11 risk variants associated with the asthma with hay fever phenotype. J Allergy Clin Immunol. 2014;133(6):1564-71. doi: 10.1016/j.jaci.2013.10.030. PubMed PMID: 24388013; PubMed Central PMCID: PMCPMC4280183.

112. Alonso-Perez E, Suarez-Gestal M, Calaza M, Ordi-Ros J, Balada E, Bijl M, et al. Further evidence of subphenotype association with systemic lupus erythematosus susceptibility loci: a European cases only study. PLoS One. 2012;7(9):e45356. doi: 10.1371/journal.pone.0045356. PubMed PMID: 23049788; PubMed Central PMCID: PMCPMC3458859.

113. International Consortium for Systemic Lupus Erythematosus G, Harley JB, Alarcon-Riquelme ME, Criswell LA, Jacob CO, Kimberly RP, et al. Genome-wide association scan in women with systemic lupus erythematosus identifies susceptibility variants in ITGAM, PXK, KIAA1542 and other loci. Nat Genet. 2008;40(2):204-10. doi: 10.1038/ng.81. PubMed PMID: 18204446; PubMed Central PMCID: PMCPMC3712260.

114. Ikram MK, Sim X, Jensen RA, Cotch MF, Hewitt AW, Ikram MA, et al. Four novel Loci (19q13, 6q24, 12q24, and 5q14) influence the microcirculation in vivo. PLoS Genet. 2010;6(10):e1001184. doi: 10.1371/journal.pgen.1001184. PubMed PMID: 21060863; PubMed Central PMCID: PMCPMC2965750.

115. Aberg K, Adkins DE, Bukszar J, Webb BT, Caroff SN, Miller DD, et al. Genomewide association study of movement-related adverse antipsychotic effects. Biol Psychiatry. 2010;67(3):279-82. doi: 10.1016/j.biopsych.2009.08.036. PubMed PMID: 19875103; PubMed Central PMCID: PMCPMC3388725.

116. Cirulli ET, Kasperaviciute D, Attix DK, Need AC, Ge D, Gibson G, et al. Common genetic variation and performance on standardized cognitive tests. Eur J Hum Genet. 2010;18(7):815-20. doi: 10.1038/ejhg.2010.2. PubMed PMID: 20125193; PubMed Central PMCID: PMCPMC2987367.

117. Hom G, Graham RR, Modrek B, Taylor KE, Ortmann W, Garnier S, et al. Association of systemic lupus erythematosus with C8orf13-BLK and ITGAM-ITGAX. N Engl J Med. 2008;358(9):900-9. doi: 10.1056/NEJMoa0707865. PubMed PMID: 18204098.

118. Borowiec M, Liew CW, Thompson R, Boonyasrisawat W, Hu J, Mlynarski WM, et al. Mutations at the BLK locus linked to maturity onset diabetes of the young and beta-cell dysfunction. Proc Natl Acad Sci U S A. 2009;106(34):14460-5. doi: 10.1073/pnas.0906474106. PubMed PMID: 19667185; PubMed Central PMCID: PMCPMC2732833.

119. Gharahkhani P, Fitzgerald RC, Vaughan TL, Palles C, Gockel I, Tomlinson I, et al. Genome-wide association studies in oesophageal adenocarcinoma and Barrett's oesophagus: a large-scale meta-analysis. Lancet Oncol. 2016;17(10):1363-73. doi: 10.1016/S1470-2045(16)30240-6. PubMed PMID: 27527254; PubMed Central PMCID: PMCPMC5052458.

120. Ji J, Sundquist J, Sundquist K. Associations of alcohol use disorders with esophageal and gastric cancers: a population-based study in Sweden. Eur J Cancer Prev. 2017;26(2):119-24. doi: 10.1097/CEJ.0000000000000227. PubMed PMID: 26886238.

121. McGue M, Zhang Y, Miller MB, Basu S, Vrieze S, Hicks B, et al. A genome-wide association study of behavioral disinhibition. Behav Genet. 2013;43(5):363-73. doi: 10.1007/s10519-013-9606-x. PubMed PMID: 23942779; PubMed Central PMCID: PMCPMC3886341.

122. Bentham J, Morris DL, Cunninghame Graham DS, Pinder CL, Tombleson P, Behrens TW, et al. Genetic association analyses implicate aberrant regulation of innate and adaptive immunity genes in the pathogenesis of systemic lupus erythematosus. Nat Genet. 2015;47(12):1457-64. doi: 10.1038/ng.3434. PubMed PMID: 26502338; PubMed Central PMCID: PMCPMC4668589.

123. Han JW, Zheng HF, Cui Y, Sun LD, Ye DQ, Hu Z, et al. Genome-wide association study in a Chinese Han population identifies nine new susceptibility loci for systemic lupus erythematosus. Nat Genet. 2009;41(11):1234-7. doi: 10.1038/ng.472. PubMed PMID: 19838193.

124. Yang W, Tang H, Zhang Y, Tang X, Zhang J, Sun L, et al. Meta-analysis followed by replication identifies loci in or near CDKN1B, TET3, CD80, DRAM1, and ARID5B as associated with systemic lupus erythematosus in Asians. Am J Hum Genet. 2013;92(1):41-51. doi: 10.1016/j.ajhg.2012.11.018. PubMed PMID: 23273568; PubMed Central PMCID: PMCPMC3542470.

125. Okada Y, Wu D, Trynka G, Raj T, Terao C, Ikari K, et al. Genetics of rheumatoid arthritis contributes to biology and drug discovery. Nature. 2014;506(7488):376-81. doi: 10.1038/nature12873. PubMed PMID: 24390342; PubMed Central PMCID: PMCPMC3944098.

126. Gregersen PK, Amos CI, Lee AT, Lu Y, Remmers EF, Kastner DL, et al. REL, encoding a member of the NF-kappaB family of transcription factors, is a newly defined risk locus for rheumatoid arthritis. Nat Genet. 2009;41(7):820-3. doi: 10.1038/ng.395. PubMed PMID: 19503088; PubMed Central PMCID: PMCPMC2705058.

127. Onouchi Y, Ozaki K, Burns JC, Shimizu C, Terai M, Hamada H, et al. A genome-wide association study identifies three new risk loci for Kawasaki disease. Nat Genet. 2012;44(5):517-21. doi: 10.1038/ng.2220. PubMed PMID: 22446962.

128. Lee YC, Kuo HC, Chang JS, Chang LY, Huang LM, Chen MR, et al. Two new susceptibility loci for Kawasaki disease identified through genome-wide association analysis. Nat Genet. 2012;44(5):522-5. doi: 10.1038/ng.2227. PubMed PMID: 22446961.

129. Muiya NP, Wakil SM, Tahir AI, Hagos S, Najai M, Gueco D, et al. A study of the role of GATA4 polymorphism in cardiovascular metabolic disorders. Hum Genomics. 2013;7:25. doi: 10.1186/1479-7364-7-25. PubMed PMID: 24330461; PubMed Central PMCID: PMCPMC3899629.

130. Stefanovic S, Christoffels VM. GATA-dependent transcriptional and epigenetic control of cardiac lineage specification and differentiation. Cell Mol Life Sci. 2015;72(20):3871-81. doi: 10.1007/s00018-015-1974-3. PubMed PMID: 26126786; PubMed Central PMCID: PMCPMC4575685.

131. Liu J, Siyahhan Julnes P, Chen J, Ehrlich S, Walton E, Calhoun VD. The association of DNA methylation and brain volume in healthy individuals and schizophrenia patients. Schizophr Res. 2015;169(1-3):447-52. doi: 10.1016/j.schres.2015.08.035. PubMed PMID: 26381449; PubMed Central PMCID: PMCPMC4681600.

132. Zois E, Vollstadt-Klein S, Hoffmann S, Reinhard I, Bach P, Charlet K, et al. GATA4 variant interaction with brain limbic structure and relapse risk: A voxel-based morphometry study. Eur Neuropsychopharmacol. 2016;26(9):1431-7. doi: 10.1016/j.euroneuro.2016.06.011. PubMed PMID: 27397865.

133. Edenberg HJ, Koller DL, Xuei X, Wetherill L, McClintick JN, Almasy L, et al. Genome-wide association study of alcohol dependence implicates a region on chromosome 11. Alcohol Clin Exp Res. 2010;34(5):840-52. doi: 10.1111/j.1530-0277.2010.01156.x. PubMed PMID: 20201924; PubMed Central PMCID: PMCPMC2884073.

134. Treutlein J, Cichon S, Ridinger M, Wodarz N, Soyka M, Zill P, et al. Genome-wide association study of alcohol dependence. Arch Gen Psychiatry. 2009;66(7):773-84. doi: 10.1001/archgenpsychiatry.2009.83. PubMed PMID: 19581569; PubMed Central PMCID: PMCPMC4229246.

135. Karpyak VM, Winham SJ, Biernacka JM, Cunningham JM, Lewis KA, Geske JR, et al. Association of GATA4 sequence variation with alcohol dependence. Addict Biol. 2014;19(2):312-5. doi: 10.1111/j.1369-1600.2012.00482.x. PubMed PMID: 22862823; PubMed Central PMCID: PMCPMC3504631.

136. Kiefer F, Witt SH, Frank J, Richter A, Treutlein J, Lemenager T, et al. Involvement of the atrial natriuretic peptide transcription factor GATA4 in alcohol dependence, relapse risk and treatment response to acamprosate. Pharmacogenomics J. 2011;11(5):368-74. doi: 10.1038/tpj.2010.51. PubMed PMID: 20585342.

137. Jorde A, Bach P, Witt SH, Becker K, Reinhard I, Vollstadt-Klein S, et al. Genetic variation in the atrial natriuretic peptide transcription factor GATA4 modulates amygdala responsiveness in alcohol dependence. Biol Psychiatry. 2014;75(10):790-7. doi: 10.1016/j.biopsych.2013.10.020. PubMed PMID: 24314346.

138. Wang J, Sun YM, Yang YQ. Mutation spectrum of the GATA4 gene in patients with idiopathic atrial fibrillation. Mol Biol Rep. 2012;39(8):8127-35. doi: 10.1007/s11033-012-1660-6. PubMed PMID: 22552926.

139. Wang H, Ozaki T, Shamim Hossain M, Nakamura Y, Kamijo T, Xue X, et al. A newly identified dependence receptor UNC5H4 is induced during DNA damage-mediated apoptosis and transcriptional target of tumor suppressor p53. Biochem Biophys Res Commun. 2008;370(4):594-8. doi: 10.1016/j.bbrc.2008.03.152. PubMed PMID: 18402767.

140. Wang H, Wu Q, Li S, Zhang B, Chi Z, Hao L. Unc5D regulates p53-dependent apoptosis in neuroblastoma cells. Mol Med Rep. 2014;9(6):2411-6. doi: 10.3892/mmr.2014.2100. PubMed PMID: 24691657.

141. Lu D, Dong D, Zhou Y, Lu M, Pang XW, Li Y, et al. The tumor-suppressive function of UNC5D and its repressed expression in renal cell carcinoma. Clin Cancer Res. 2013;19(11):2883-92. doi: 10.1158/1078-0432.CCR-12-2978. PubMed PMID: 23589179.

142. Dantzig AH, Hoskins JA, Tabas LB, Bright S, Shepard RL, Jenkins IL, et al. Association of intestinal peptide transport with a protein related to the cadherin superfamily. Science. 1994;264(5157):430-3. PubMed PMID: 8153632.

143. Bartolome RA, Barderas R, Torres S, Fernandez-Acenero MJ, Mendes M, Garcia-Foncillas J, et al. Cadherin-17 interacts with alpha2beta1 integrin to regulate cell proliferation and adhesion in colorectal cancer cells causing liver metastasis. Oncogene. 2014;33(13):1658-69. doi: 10.1038/onc.2013.117. PubMed PMID: 23604127.

144. Zhu X, Feng T, Tayo BO, Liang J, Young JH, Franceschini N, et al. Meta-analysis of correlated traits via summary statistics from GWASs with an application in hypertension. Am J Hum Genet. 2015;96(1):21-36. doi: 10.1016/j.ajhg.2014.11.011. PubMed PMID: 25500260; PubMed Central PMCID: PMCPMC4289691.

145. Yucesoy B, Kaufman KM, Lummus ZL, Weirauch MT, Zhang G, Cartier A, et al. Genome-Wide Association Study Identifies Novel Loci Associated With Diisocyanate-Induced Occupational Asthma. Toxicol Sci. 2015;146(1):192-201. doi: 10.1093/toxsci/kfv084. PubMed PMID: 25918132; PubMed Central PMCID: PMCPMC4560052.

146. Vilarino-Guell C, Wider C, Ross OA, Jasinska-Myga B, Kachergus J, Cobb SA, et al. LINGO1 and LINGO2 variants are associated with essential tremor and Parkinson disease. Neurogenetics. 2010;11(4):401-8. doi: 10.1007/s10048-010-0241-x. PubMed PMID: 20369371; PubMed Central PMCID: PMCPMC3930084.

147. Jasinska-Myga B, Wider C. Genetics of essential tremor. Parkinsonism Relat Disord. 2012;18 Suppl 1:S138-9. doi: 10.1016/S1353-8020(11)70043-8. PubMed PMID: 22166413.

148. Hromatka BS, Tung JY, Kiefer AK, Do CB, Hinds DA, Eriksson N. Genetic variants associated with motion sickness point to roles for inner ear development, neurological processes and glucose homeostasis. Hum Mol Genet. 2015;24(9):2700-8. doi: 10.1093/hmg/ddv028. PubMed PMID: 25628336; PubMed Central PMCID: PMCPMC4383869.

149. Boecker H, Wills AJ, Ceballos-Baumann A, Samuel M, Thompson PD, Findley LJ, et al. The effect of ethanol on alcohol-responsive essential tremor: a positron emission tomography study. Ann Neurol. 1996;39(5):650-8. doi: 10.1002/ana.410390515. PubMed PMID: 8619551.

150. Kapoor M, Wang JC, Wetherill L, Le N, Bertelsen S, Hinrichs AL, et al. Genome-wide survival analysis of age at onset of alcohol dependence in extended high-risk COGA families. Drug Alcohol Depend. 2014;142:56-62. doi: 10.1016/j.drugalcdep.2014.05.023. PubMed PMID: 24962325; PubMed Central PMCID: PMCPMC4127128.

151. Speliotes EK, Willer CJ, Berndt SI, Monda KL, Thorleifsson G, Jackson AU, et al. Association analyses of 249,796 individuals reveal 18 new loci associated with body mass index. Nat Genet. 2010;42(11):937-48. doi: 10.1038/ng.686. PubMed PMID: 20935630; PubMed Central PMCID: PMCPMC3014648.

152. Rask-Andersen M, Almen MS, Lind L, Schioth HB. Association of the LINGO2-related SNP rs10968576 with body mass in a cohort of elderly Swedes. Mol Genet Genomics. 2015;290(4):1485-91. doi: 10.1007/s00438-015-1009-7. PubMed PMID: 25711307.

153. Athanasiu L, Smorr LL, Tesli M, Rossberg JI, Sonderby IE, Spigset O, et al. Genome-wide association study identifies common variants associated with pharmacokinetics of psychotropic drugs. J Psychopharmacol. 2015;29(8):884-91. doi: 10.1177/0269881115584469. PubMed PMID: 25944848.

154. Sherva R, Wang Q, Kranzler H, Zhao H, Koesterer R, Herman A, et al. Genome-wide Association Study of Cannabis Dependence Severity, Novel Risk Variants, and Shared Genetic Risks. JAMA Psychiatry. 2016;73(5):472-80. doi: 10.1001/jamapsychiatry.2016.0036. PubMed PMID: 27028160; PubMed Central PMCID: PMCPMC4974817.

155. Nalls MA, Pankratz N, Lill CM, Do CB, Hernandez DG, Saad M, et al. Large-scale meta-analysis of genome-wide association data identifies six new risk loci for Parkinson's disease. Nat Genet. 2014;46(9):989-93. doi: 10.1038/ng.3043. PubMed PMID: 25064009; PubMed Central PMCID: PMCPMC4146673.

156. Hansel NN, Pare PD, Rafaels N, Sin DD, Sandford A, Daley D, et al. Genome-Wide Association Study Identification of Novel Loci Associated with Airway Responsiveness in Chronic Obstructive Pulmonary Disease. Am J Respir Cell Mol Biol. 2015;53(2):226-34. doi: 10.1165/rcmb.2014-0198OC. PubMed PMID: 25514360; PubMed Central PMCID: PMCPMC4566043.

157. Chung SA, Brown EE, Williams AH, Ramos PS, Berthier CC, Bhangale T, et al. Lupus nephritis susceptibility loci in women with systemic lupus erythematosus. J Am Soc Nephrol. 2014;25(12):2859-70. doi: 10.1681/ASN.2013050446. PubMed PMID: 24925725; PubMed Central PMCID: PMCPMC4243339.

158. Marshall TW, Aloor HL, Bear JE. Coronin 2A regulates a subset of focal-adhesion-turnover events through the cofilin pathway. J Cell Sci. 2009;122(Pt 17):3061-9. doi: 10.1242/jcs.051482. PubMed PMID: 19654210; PubMed Central PMCID: PMCPMC2729258.

159. Yoon HG, Chan DW, Huang ZQ, Li J, Fondell JD, Qin J, et al. Purification and functional characterization of the human N-CoR complex: the roles of HDAC3, TBL1 and TBLR1. EMBO J. 2003;22(6):1336-46. doi: 10.1093/emboj/cdg120. PubMed PMID: 12628926; PubMed Central PMCID: PMCPMC151047.

160. Rastetter RH, Blomacher M, Drebber U, Marko M, Behrens J, Solga R, et al. Coronin 2A (CRN5) expression is associated with colorectal adenoma-adenocarcinoma sequence and oncogenic signalling. BMC Cancer. 2015;15:638. doi: 10.1186/s12885-015-1645-7. PubMed PMID: 26373535; PubMed Central PMCID: PMCPMC4612562.

161. Huang W, Ghisletti S, Saijo K, Gandhi M, Aouadi M, Tesz GJ, et al. Coronin 2A mediates actin-dependent de-repression of inflammatory response genes. Nature. 2011;470(7334):414-8. doi: 10.1038/nature09703. PubMed PMID: 21331046; PubMed Central PMCID: PMCPMC3464905.

162. Wang XP, Cheng ZY, Schmid KL. GABAB receptors are expressed in human aortic smooth muscle cells and regulate the intracellular Ca(2+) concentration. Heart Vessels. 2015;30(2):249-57. doi: 10.1007/s00380-014-0499-2. PubMed PMID: 24682435.

163. Caputo F, Ciminelli BM, Jodice C, Blasi P, Vignoli T, Cibin M, et al. Alcohol use disorder and GABAB receptor gene polymorphisms in an Italian sample: haplotype frequencies, linkage disequilibrium and association studies. Ann Hum Biol. 2017:1-5. doi: 10.1080/03014460.2017.1287307. PubMed PMID: 28118741.

164. Yang J, Wang S, Yang Z, Hodgkinson CA, Iarikova P, Ma JZ, et al. The contribution of rare and common variants in 30 genes to risk nicotine dependence. Mol Psychiatry. 2015;20(11):1467-78. doi: 10.1038/mp.2014.156. PubMed PMID: 25450229; PubMed Central PMCID: PMCPMC4452458.

165. Li MD, Mangold JE, Seneviratne C, Chen GB, Ma JZ, Lou XY, et al. Association and interaction analyses of GABBR1 and GABBR2 with nicotine dependence in European- and African-American populations. PLoS One. 2009;4(9):e7055. doi: 10.1371/journal.pone.0007055. PubMed PMID: 19763258; PubMed Central PMCID: PMCPMC2739294.

166. Fatemi SH, Folsom TD, Thuras PD. Deficits in GABA(B) receptor system in schizophrenia and mood disorders: a postmortem study. Schizophr Res. 2011;128(1-3):37-43. doi: 10.1016/j.schres.2010.12.025. PubMed PMID: 21303731; PubMed Central PMCID: PMCPMC3085603.

167. Zhan M, Chen G, Pan CM, Gu ZH, Zhao SX, Liu W, et al. Genome-wide association study identifies a novel susceptibility gene for serum TSH levels in Chinese populations. Hum Mol Genet. 2014;23(20):5505-17. doi: 10.1093/hmg/ddu250. PubMed PMID: 24852370.

168. Mancikova V, Cruz R, Inglada-Perez L, Fernandez-Rozadilla C, Landa I, Cameselle-Teijeiro J, et al. Thyroid cancer GWAS identifies 10q26.12 and 6q14.1 as novel susceptibility loci and reveals genetic heterogeneity among populations. Int J Cancer. 2015;137(8):1870-8. doi: 10.1002/ijc.29557. PubMed PMID: 25855579.

169. Porcu E, Medici M, Pistis G, Volpato CB, Wilson SG, Cappola AR, et al. A meta-analysis of thyroid-related traits reveals novel loci and gender-specific differences in the regulation of thyroid function. PLoS Genet. 2013;9(2):e1003266. doi: 10.1371/journal.pgen.1003266. PubMed PMID: 23408906; PubMed Central PMCID: PMCPMC3567175.

170. Eriksson N, Tung JY, Kiefer AK, Hinds DA, Francke U, Mountain JL, et al. Novel associations for hypothyroidism include known autoimmune risk loci. PLoS One. 2012;7(4):e34442. doi: 10.1371/journal.pone.0034442. PubMed PMID: 22493691; PubMed Central PMCID: PMCPMC3321023.

171. Denny JC, Crawford DC, Ritchie MD, Bielinski SJ, Basford MA, Bradford Y, et al. Variants near FOXE1 are associated with hypothyroidism and other thyroid conditions: using electronic medical records for genome- and phenome-wide studies. Am J Hum Genet. 2011;89(4):529-42. doi: 10.1016/j.ajhg.2011.09.008. PubMed PMID: 21981779; PubMed Central PMCID: PMCPMC3188836.

172. Kohler A, Chen B, Gemignani F, Elisei R, Romei C, Figlioli G, et al. Genome-wide association study on differentiated thyroid cancer. J Clin Endocrinol Metab. 2013;98(10):E1674-81. doi: 10.1210/jc.2013-1941. PubMed PMID: 23894154.

173. Takahashi M, Saenko VA, Rogounovitch TI, Kawaguchi T, Drozd VM, Takigawa-Imamura H, et al. The FOXE1 locus is a major genetic determinant for radiation-related thyroid carcinoma in Chernobyl. Hum Mol Genet. 2010;19(12):2516-23. doi: 10.1093/hmg/ddq123. PubMed PMID: 20350937.

174. Gudmundsson J, Sulem P, Gudbjartsson DF, Jonasson JG, Sigurdsson A, Bergthorsson JT, et al. Common variants on 9q22.33 and 14q13.3 predispose to thyroid cancer in European populations. Nat Genet. 2009;41(4):460-4. doi: 10.1038/ng.339. PubMed PMID: 19198613; PubMed Central PMCID: PMCPMC3664837.

175. Garcia-Etxebarria K, Bracho MA, Galan JC, Pumarola T, Castilla J, Ortiz de Lejarazu R, et al. No Major Host Genetic Risk Factor Contributed to A(H1N1)2009 Influenza Severity. PLoS One. 2015;10(9):e0135983. doi: 10.1371/journal.pone.0135983. PubMed PMID: 26379185; PubMed Central PMCID: PMCPMC4574704.

176. Hazra A, Kraft P, Lazarus R, Chen C, Chanock SJ, Jacques P, et al. Genome-wide significant predictors of metabolites in the one-carbon metabolism pathway. Hum Mol Genet. 2009;18(23):4677-87. doi: 10.1093/hmg/ddp428. PubMed PMID: 19744961; PubMed Central PMCID: PMCPMC2773275.

177. Coronary Artery Disease Genetics C. A genome-wide association study in Europeans and South Asians identifies five new loci for coronary artery disease. Nat Genet. 2011;43(4):339-44. doi: 10.1038/ng.782. PubMed PMID: 21378988.

178. Ashley-Koch AE, Garrett ME, Gibson J, Liu Y, Dennis MF, Kimbrel NA, et al. Genome-wide association study of posttraumatic stress disorder in a cohort of Iraq-Afghanistan era veterans. J Affect Disord. 2015;184:225-34. doi: 10.1016/j.jad.2015.03.049. PubMed PMID: 26114229; PubMed Central PMCID: PMCPMC4697755.

179. Lake RJ, Boetefuer EL, Won KJ, Fan HY. The CSB chromatin remodeler and CTCF architectural protein cooperate in response to oxidative stress. Nucleic Acids Res. 2016;44(5):2125-35. doi: 10.1093/nar/gkv1219. PubMed PMID: 26578602; PubMed Central PMCID: PMCPMC4797267.

180. Shehata L, Simeonov DR, Raams A, Wolfe L, Vanderver A, Li X, et al. ERCC6 dysfunction presenting as progressive neurological decline with brain hypomyelination. Am J Med Genet A. 2014;164A(11):2892-900. doi: 10.1002/ajmg.a.36709. PubMed PMID: 25251875; PubMed Central PMCID: PMCPMC4205164.

181. Abbasi R, Ramroth H, Becher H, Dietz A, Schmezer P, Popanda O. Laryngeal cancer risk associated with smoking and alcohol consumption is modified by genetic polymorphisms in ERCC5, ERCC6 and RAD23B but not by polymorphisms in five other nucleotide excision repair genes. Int J Cancer. 2009;125(6):1431-9. Epub 2009/05/16. doi: 10.1002/ijc.24442. PubMed PMID: 19444904.

182. Vetreno RP, Broadwater M, Liu W, Spear LP, Crews FT. Adolescent, but not adult, binge ethanol exposure leads to persistent global reductions of choline acetyltransferase expressing neurons in brain. PLoS One. 2014;9(11):e113421. doi: 10.1371/journal.pone.0113421. PubMed PMID: 25405505; PubMed Central PMCID: PMCPMC4236188.

183. Hachisu M, Konishi K, Hosoi M, Tani M, Tomioka H, Inamoto A, et al. Beyond the Hypothesis of Serum Anticholinergic Activity in Alzheimer's Disease: Acetylcholine Neuronal Activity Modulates Brain-Derived Neurotrophic Factor Production and Inflammation in the Brain. Neurodegener Dis. 2015;15(3):182-7. doi: 10.1159/000381531. PubMed PMID: 26138497.

184. Barthet G, Carrat G, Cassier E, Barker B, Gaven F, Pillot M, et al. Beta-arrestin1 phosphorylation by GRK5 regulates G protein-independent 5-HT4 receptor signalling. EMBO J. 2009;28(18):2706-18. doi: 10.1038/emboj.2009.215. PubMed PMID: 19661922; PubMed Central PMCID: PMCPMC2750015.

185. Fan J, Malik AB. Toll-like receptor-4 (TLR4) signaling augments chemokine-induced neutrophil migration by modulating cell surface expression of chemokine receptors. Nat Med. 2003;9(3):315-21. doi: 10.1038/nm832. PubMed PMID: 12592402.

186. Michal AM, So CH, Beeharry N, Shankar H, Mashayekhi R, Yen TJ, et al. G Protein-coupled receptor kinase 5 is localized to centrosomes and regulates cell cycle progression. J Biol Chem. 2012;287(9):6928-40. doi: 10.1074/jbc.M111.298034. PubMed PMID: 22223642; PubMed Central PMCID: PMCPMC3307296.

187. Traynham CJ, Hullmann J, Koch WJ. "Canonical and non-canonical actions of GRK5 in the heart". J Mol Cell Cardiol. 2016;92:196-202. doi: 10.1016/j.yjmcc.2016.01.027. PubMed PMID: 26829117; PubMed Central PMCID: PMCPMC4789097.

188. Liggett SB, Cresci S, Kelly RJ, Syed FM, Matkovich SJ, Hahn HS, et al. A GRK5 polymorphism that inhibits beta-adrenergic receptor signaling is protective in heart failure. Nat Med. 2008;14(5):510-7. doi: 10.1038/nm1750. PubMed PMID: 18425130; PubMed Central PMCID: PMCPMC2596476.

189. Lobmeyer MT, Wang L, Zineh I, Turner ST, Gums JG, Chapman AB, et al. Polymorphisms in genes coding for GRK2 and GRK5 and response differences in antihypertensive-treated patients. Pharmacogenet Genomics. 2011;21(1):42-9. doi: 10.1097/FPC.0b013e328341e911. PubMed PMID: 21127457; PubMed Central PMCID: PMCPMC3028503.

190. Harris DM, Cohn HI, Pesant S, Eckhart AD. GPCR signalling in hypertension: role of GRKs. Clin Sci (Lond). 2008;115(3):79-89. doi: 10.1042/CS20070442. PubMed PMID: 18593382.

191. Keys JR, Zhou RH, Harris DM, Druckman CA, Eckhart AD. Vascular smooth muscle overexpression of G protein-coupled receptor kinase 5 elevates blood pressure, which segregates with sex and is dependent on Gi-mediated signaling. Circulation. 2005;112(8):1145-53. doi: 10.1161/CIRCULATIONAHA.104.531657. PubMed PMID: 16103237.

192. Cohn HI, Xi Y, Pesant S, Harris DM, Hyslop T, Falkner B, et al. G protein-coupled receptor kinase 2 expression and activity are associated with blood pressure in black Americans. Hypertension. 2009;54(1):71-6. doi: 10.1161/HYPERTENSIONAHA.108.125955. PubMed PMID: 19487588; PubMed Central PMCID: PMCPMC2745090.

193. Li H, Gan W, Lu L, Dong X, Han X, Hu C, et al. A genome-wide association study identifies GRK5 and RASGRP1 as type 2 diabetes loci in Chinese Hans. Diabetes. 2013;62(1):291-8. doi: 10.2337/db12-0454. PubMed PMID: 22961080; PubMed Central PMCID: PMCPMC3526061.

194. Kuo HC, Li SC, Guo MM, Huang YH, Yu HR, Huang FC, et al. Genome-Wide Association Study Identifies Novel Susceptibility Genes Associated with Coronary Artery Aneurysm Formation in Kawasaki Disease. PLoS One. 2016;11(5):e0154943. doi: 10.1371/journal.pone.0154943. PubMed PMID: 27171184; PubMed Central PMCID: PMCPMC4865092.

195. Carrizzo A, Damato A, Ambrosio M, Falco A, Rosati A, Capunzo M, et al. The prosurvival protein BAG3: a new participant in vascular homeostasis. Cell Death Dis. 2016;7(10):e2431. doi: 10.1038/cddis.2016.321. PubMed PMID: 27763645; PubMed Central PMCID: PMCPMC5133988.

196. Villard E, Perret C, Gary F, Proust C, Dilanian G, Hengstenberg C, et al. A genome-wide association study identifies two loci associated with heart failure due to dilated cardiomyopathy. Eur Heart J. 2011;32(9):1065-76. doi: 10.1093/eurheartj/ehr105. PubMed PMID: 21459883; PubMed Central PMCID: PMCPMC3086901.

197. McClintick JN, Xuei X, Tischfield JA, Goate A, Foroud T, Wetherill L, et al. Stress-response pathways are altered in the hippocampus of chronic alcoholics. Alcohol. 2013;47(7):505-15. doi: 10.1016/j.alcohol.2013.07.002. PubMed PMID: 23981442; PubMed Central PMCID: PMCPMC3836826.

198. Ji C. Advances and New Concepts in Alcohol-Induced Organelle Stress, Unfolded Protein Responses and Organ Damage. Biomolecules. 2015;5(2):1099-121. doi: 10.3390/biom5021099. PubMed PMID: 26047032; PubMed Central PMCID: PMCPMC4496712.

199. Guo Y, Yu W, Sun D, Wang J, Li C, Zhang R, et al. A novel protective mechanism for mitochondrial aldehyde dehydrogenase (ALDH2) in type i diabetes-induced cardiac dysfunction: role of AMPK-regulated autophagy. Biochim Biophys Acta. 2015;1852(2):319-31. doi: 10.1016/j.bbadis.2014.05.017. PubMed PMID: 24874076.

200. Ke Z, Wang X, Liu Y, Fan Z, Chen G, Xu M, et al. Ethanol induces endoplasmic reticulum stress in the developing brain. Alcohol Clin Exp Res. 2011;35(9):1574-83. doi: 10.1111/j.1530-0277.2011.01503.x. PubMed PMID: 21599712; PubMed Central PMCID: PMCPMC4986072.

201. Pani SP, Krishnamoorthy K, Rao AS, Prathiba J. Clinical manifestations in malayan filariasis infection with special reference to lymphoedema grading. Indian J Med Res. 1990;91:200-7. PubMed PMID: 2397942.

202. Gamerdinger M, Hajieva P, Kaya AM, Wolfrum U, Hartl FU, Behl C. Protein quality control during aging involves recruitment of the macroautophagy pathway by BAG3. EMBO J. 2009;28(7):889-901. doi: 10.1038/emboj.2009.29. PubMed PMID: 19229298; PubMed Central PMCID: PMCPMC2647772.

203. Selcen D, Muntoni F, Burton BK, Pegoraro E, Sewry C, Bite AV, et al. Mutation in BAG3 causes severe dominant childhood muscular dystrophy. Ann Neurol. 2009;65(1):83-9. doi: 10.1002/ana.21553. PubMed PMID: 19085932; PubMed Central PMCID: PMCPMC2639628.

204. Tani K, Kogure T, Inoue H. The intracellular phospholipase A1 protein family. Biomol Concepts. 2012;3(5):471-8. doi: 10.1515/bmc-2012-0014. PubMed PMID: 25436551.

205. Ong YS, Tang BL, Loo LS, Hong W. p125A exists as part of the mammalian Sec13/Sec31 COPII subcomplex to facilitate ER-Golgi transport. J Cell Biol. 2010;190(3):331-45. doi: 10.1083/jcb.201003005. PubMed PMID: 20679433; PubMed Central PMCID: PMCPMC2922642.

206. Perry JR, Day F, Elks CE, Sulem P, Thompson DJ, Ferreira T, et al. Parent-of-origin-specific allelic associations among 106 genomic loci for age at menarche. Nature. 2014;514(7520):92-7. doi: 10.1038/nature13545. PubMed PMID: 25231870; PubMed Central PMCID: PMCPMC4185210.

207. Zayats T, Jacobsen KK, Kleppe R, Jacob CP, Kittel-Schneider S, Ribases M, et al. Exome chip analyses in adult attention deficit hyperactivity disorder. Transl Psychiatry. 2016;6(10):e923. doi: 10.1038/tp.2016.196. PubMed PMID: 27754487; PubMed Central PMCID: PMCPMC5315553.

208. Reuter MS, Tawamie H, Buchert R, Hosny Gebril O, Froukh T, Thiel C, et al. Diagnostic Yield and Novel Candidate Genes by Exome Sequencing in 152 Consanguineous Families With Neurodevelopmental Disorders. JAMA Psychiatry. 2017;74(3):293-9. doi: 10.1001/jamapsychiatry.2016.3798. PubMed PMID: 28097321.

209. Takayama K, Horie-Inoue K, Suzuki T, Urano T, Ikeda K, Fujimura T, et al. TACC2 is an androgen-responsive cell cycle regulator promoting androgen-mediated and castration-resistant growth of prostate cancer. Mol Endocrinol. 2012;26(5):748-61. doi: 10.1210/me.2011-1242. PubMed PMID: 22456197.

210. Onodera Y, Takagi K, Miki Y, Takayama K, Shibahara Y, Watanabe M, et al. TACC2 (transforming acidic coiled-coil protein 2) in breast carcinoma as a potent prognostic predictor associated with cell proliferation. Cancer Med. 2016;5(8):1973-82. doi: 10.1002/cam4.736. PubMed PMID: 27333920; PubMed Central PMCID: PMCPMC4971925.

211. Fritsche LG, Chen W, Schu M, Yaspan BL, Yu Y, Thorleifsson G, et al. Seven new loci associated with age-related macular degeneration. Nat Genet. 2013;45(4):433-9, 9e1-2. doi: 10.1038/ng.2578. PubMed PMID: 23455636; PubMed Central PMCID: PMCPMC3739472.

212. Sobrin L, Ripke S, Yu Y, Fagerness J, Bhangale TR, Tan PL, et al. Heritability and genome-wide association study to assess genetic differences between advanced age-related macular degeneration subtypes. Ophthalmology. 2012;119(9):1874-85. doi: 10.1016/j.ophtha.2012.03.014. PubMed PMID: 22705344; PubMed Central PMCID: PMCPMC3899891.

213. Naj AC, Scott WK, Courtenay MD, Cade WH, Schwartz SG, Kovach JL, et al. Genetic factors in nonsmokers with age-related macular degeneration revealed through genome-wide gene-environment interaction analysis. Ann Hum Genet. 2013;77(3):215-31. doi: 10.1111/ahg.12011. PubMed PMID: 23577725; PubMed Central PMCID: PMCPMC3625984.

214. Kopplin LJ, Igo RP, Jr., Wang Y, Sivakumaran TA, Hagstrom SA, Peachey NS, et al. Genome-wide association identifies SKIV2L and MYRIP as protective factors for age-related macular degeneration. Genes Immun. 2010;11(8):609-21. doi: 10.1038/gene.2010.39. PubMed PMID: 20861866; PubMed Central PMCID: PMCPMC3375062.

215. Neale BM, Fagerness J, Reynolds R, Sobrin L, Parker M, Raychaudhuri S, et al. Genome-wide association study of advanced age-related macular degeneration identifies a role of the hepatic lipase gene (LIPC). Proc Natl Acad Sci U S A. 2010;107(16):7395-400. doi: 10.1073/pnas.0912019107. PubMed PMID: 20385826; PubMed Central PMCID: PMCPMC2867697.

216. Yu Y, Bhangale TR, Fagerness J, Ripke S, Thorleifsson G, Tan PL, et al. Common variants near FRK/COL10A1 and VEGFA are associated with advanced age-related macular degeneration. Hum Mol Genet. 2011;20(18):3699-709. doi: 10.1093/hmg/ddr270. PubMed PMID: 21665990; PubMed Central PMCID: PMCPMC3159552.

217. Dewan A, Liu M, Hartman S, Zhang SS, Liu DT, Zhao C, et al. HTRA1 promoter polymorphism in wet age-related macular degeneration. Science. 2006;314(5801):989-92. doi: 10.1126/science.1133807. PubMed PMID: 17053108.

218. Cipriani V, Leung HT, Plagnol V, Bunce C, Khan JC, Shahid H, et al. Genome-wide association study of age-related macular degeneration identifies associated variants in the TNXB-FKBPL-NOTCH4 region of chromosome 6p21.3. Hum Mol Genet. 2012;21(18):4138-50. doi: 10.1093/hmg/dds225. PubMed PMID: 22694956; PubMed Central PMCID: PMCPMC3428154.

219. Okada Y, Kamatani Y, Takahashi A, Matsuda K, Hosono N, Ohmiya H, et al. A genome-wide association study in 19 633 Japanese subjects identified LHX3-QSOX2 and IGF1 as adult height loci. Hum Mol Genet. 2010;19(11):2303-12. doi: 10.1093/hmg/ddq091. PubMed PMID: 20189936.

220. Replication DIG, Meta-analysis C, Asian Genetic Epidemiology Network Type 2 Diabetes C, South Asian Type 2 Diabetes C, Mexican American Type 2 Diabetes C, Type 2 Diabetes Genetic Exploration by Nex-generation sequencing in muylti-Ethnic Samples C, et al. Genome-wide trans-ancestry meta-analysis provides insight into the genetic architecture of type 2 diabetes susceptibility. Nat Genet. 2014;46(3):234-44. doi: 10.1038/ng.2897. PubMed PMID: 24509480; PubMed Central PMCID: PMCPMC3969612.

221. Rose JE, Behm FM, Drgon T, Johnson C, Uhl GR. Personalized smoking cessation: interactions between nicotine dose, dependence and quit-success genotype score. Mol Med. 2010;16(7-8):247-53. doi: 10.2119/molmed.2009.00159. PubMed PMID: 20379614; PubMed Central PMCID: PMCPMC2896464.

222. Siedlinski M, Cho MH, Bakke P, Gulsvik A, Lomas DA, Anderson W, et al. Genome-wide association study of smoking behaviours in patients with COPD. Thorax. 2011;66(10):894-902. doi: 10.1136/thoraxjnl-2011-200154. PubMed PMID: 21685187; PubMed Central PMCID: PMCPMC3302576.

223. Smith EN, Bloss CS, Badner JA, Barrett T, Belmonte PL, Berrettini W, et al. Genome-wide association study of bipolar disorder in European American and African American individuals. Mol Psychiatry. 2009;14(8):755-63. doi: 10.1038/mp.2009.43. PubMed PMID: 19488044; PubMed Central PMCID: PMCPMC3035981.

224. Grupe A, Li Y, Rowland C, Nowotny P, Hinrichs AL, Smemo S, et al. A scan of chromosome 10 identifies a novel locus showing strong association with late-onset Alzheimer disease. Am J Hum Genet. 2006;78(1):78-88. doi: 10.1086/498851. PubMed PMID: 16385451; PubMed Central PMCID: PMCPMC1380225.

225. Fletcher O, Johnson N, Orr N, Hosking FJ, Gibson LJ, Walker K, et al. Novel breast cancer susceptibility locus at 9q31.2: results of a genome-wide association study. J Natl Cancer Inst. 2011;103(5):425-35. doi: 10.1093/jnci/djq563. PubMed PMID: 21263130.

226. Thompson AG, Uphill J, Lowe J, Porter MC, Lukic A, Carswell C, et al. Genome-wide association study of behavioural and psychiatric features in human prion disease. Transl Psychiatry. 2015;5:e552. doi: 10.1038/tp.2015.42. PubMed PMID: 25897833; PubMed Central PMCID: PMCPMC4462605.

227. Wang J, Bansal AT, Martin M, Germer S, Benayed R, Essioux L, et al. Genome-wide association analysis implicates the involvement of eight loci with response to tocilizumab for the treatment of rheumatoid arthritis. Pharmacogenomics J. 2013;13(3):235-41. doi: 10.1038/tpj.2012.8. PubMed PMID: 22491018.

228. Ivanova AA, East MP, Yi SL, Kahn RA. Characterization of recombinant ELMOD (cell engulfment and motility domain) proteins as GTPase-activating proteins (GAPs) for ARF family GTPases. J Biol Chem. 2014;289(16):11111-21. doi: 10.1074/jbc.M114.548529. PubMed PMID: 24616099; PubMed Central PMCID: PMCPMC4036250.

229. Pilling LC, Atkins JL, Bowman K, Jones SE, Tyrrell J, Beaumont RN, et al. Human longevity is influenced by many genetic variants: evidence from 75,000 UK Biobank participants. Aging (Albany NY). 2016;8(3):547-60. doi: 10.18632/aging.100930. PubMed PMID: 27015805; PubMed Central PMCID: PMCPMC4833145.

230. Cross-Disorder Group of the Psychiatric Genomics C. Identification of risk loci with shared effects on five major psychiatric disorders: a genome-wide analysis. Lancet. 2013;381(9875):1371-9. doi: 10.1016/S0140-6736(12)62129-1. PubMed PMID: 23453885; PubMed Central PMCID: PMCPMC3714010.

231. Eriksson N, Macpherson JM, Tung JY, Hon LS, Naughton B, Saxonov S, et al. Web-based, participant-driven studies yield novel genetic associations for common traits. PLoS Genet. 2010;6(6):e1000993. doi: 10.1371/journal.pgen.1000993. PubMed PMID: 20585627; PubMed Central PMCID: PMCPMC2891811 and own stock options in the company. 23andMe co-president AW has provided general guidance, including guidance related to the company's research undertakings and direction. PLoS Genetics' Editor-in-Chief Gregory S. Barsh is a potential consultant to 23andMe and therefore recused himself from the editorial and peer-review process. PLoS co-founder Michael B. Eisen is a member of the 23andMe Scientific Advisory Board.

232. Tom Tang Y, Emtage P, Funk WD, Hu T, Arterburn M, Park EE, et al. TAFA: a novel secreted family with conserved cysteine residues and restricted expression in the brain. Genomics. 2004;83(4):727-34. doi: 10.1016/j.ygeno.2003.10.006. PubMed PMID: 15028294.

233. Benveniste EN. Cytokine actions in the central nervous system. Cytokine Growth Factor Rev. 1998;9(3-4):259-75. PubMed PMID: 9918124.

234. Walford GA, Gustafsson S, Rybin D, Stancakova A, Chen H, Liu CT, et al. Genome-Wide Association Study of the Modified Stumvoll Insulin Sensitivity Index Identifies BCL2 and FAM19A2 as Novel Insulin Sensitivity Loci. Diabetes. 2016;65(10):3200-11. doi: 10.2337/db16-0199. PubMed PMID: 27416945; PubMed Central PMCID: PMCPMC5033262.

235. Koga AT, Strauss J, Zai C, Remington G, De Luca V. Genome-wide association analysis to predict optimal antipsychotic dosage in schizophrenia: a pilot study. J Neural Transm (Vienna). 2016;123(3):329-38. doi: 10.1007/s00702-015-1472-7. PubMed PMID: 26821981.

236. Aebi M, van Donkelaar MM, Poelmans G, Buitelaar JK, Sonuga-Barke EJ, Stringaris A, et al. Gene-set and multivariate genome-wide association analysis of oppositional defiant behavior subtypes in attention-deficit/hyperactivity disorder. Am J Med Genet B Neuropsychiatr Genet. 2016;171(5):573-88. doi: 10.1002/ajmg.b.32346. PubMed PMID: 26184070; PubMed Central PMCID: PMCPMC4715802.

237. Mather KA, Armstrong NJ, Wen W, Kwok JB, Assareh AA, Thalamuthu A, et al. Investigating the genetics of hippocampal volume in older adults without dementia. PLoS One. 2015;10(1):e0116920. doi: 10.1371/journal.pone.0116920. PubMed PMID: 25625606; PubMed Central PMCID: PMCPMC4308067.

238. Cho MH, McDonald ML, Zhou X, Mattheisen M, Castaldi PJ, Hersh CP, et al. Risk loci for chronic obstructive pulmonary disease: a genome-wide association study and meta-analysis. Lancet Respir Med. 2014;2(3):214-25. doi: 10.1016/S2213-2600(14)70002-5. PubMed PMID: 24621683; PubMed Central PMCID: PMCPMC4176924.

239. Imboden M, Bouzigon E, Curjuric I, Ramasamy A, Kumar A, Hancock DB, et al. Genome-wide association study of lung function decline in adults with and without asthma. J Allergy Clin Immunol. 2012;129(5):1218-28. doi: 10.1016/j.jaci.2012.01.074. PubMed PMID: 22424883; PubMed Central PMCID: PMCPMC3340499.

240. Parker MM, Foreman MG, Abel HJ, Mathias RA, Hetmanski JB, Crapo JD, et al. Admixture mapping identifies a quantitative trait locus associated with FEV1/FVC in the COPDGene Study. Genet Epidemiol. 2014;38(7):652-9. doi: 10.1002/gepi.21847. PubMed PMID: 25112515; PubMed Central PMCID: PMCPMC4190160.

241. Strehl S, Glatt K, Liu QM, Glatt H, Lalande M. Characterization of two novel protocadherins (PCDH8 and PCDH9) localized on human chromosome 13 and mouse chromosome 14. Genomics. 1998;53(1):81-9. doi: 10.1006/geno.1998.5467. PubMed PMID: 9787079.

242. Wang C, Yu G, Liu J, Wang J, Zhang Y, Zhang X, et al. Downregulation of PCDH9 predicts prognosis for patients with glioma. J Clin Neurosci. 2012;19(4):541-5. doi: 10.1016/j.jocn.2011.04.047. PubMed PMID: 22300792.

243. Wang K, Li WD, Zhang CK, Wang Z, Glessner JT, Grant SF, et al. A genome-wide association study on obesity and obesity-related traits. PLoS One. 2011;6(4):e18939. doi: 10.1371/journal.pone.0018939. PubMed PMID: 21552555; PubMed Central PMCID: PMCPMC3084240.

244. Seppala I, Kleber ME, Lyytikainen LP, Hernesniemi JA, Makela KM, Oksala N, et al. Genome-wide association study on dimethylarginines reveals novel AGXT2 variants associated with heart rate variability but not with overall mortality. Eur Heart J. 2014;35(8):524-31. doi: 10.1093/eurheartj/eht447. PubMed PMID: 24159190.

245. Wheeler HE, Gamazon ER, Stark AL, O'Donnell PH, Gorsic LK, Huang RS, et al. Genome-wide meta-analysis identifies variants associated with platinating agent susceptibility across populations. Pharmacogenomics J. 2013;13(1):35-43. doi: 10.1038/tpj.2011.38. PubMed PMID: 21844884; PubMed Central PMCID: PMCPMC3370147.

246. Dong J, Yang J, Tranah G, Franceschini N, Parimi N, Alkorta-Aranburu G, et al. Genome-wide Meta-analysis on the Sense of Smell Among US Older Adults. Medicine (Baltimore). 2015;94(47):e1892. doi: 10.1097/MD.0000000000001892. PubMed PMID: 26632684; PubMed Central PMCID: PMCPMC5058953 authors declare no competing financial interests.

247. Chumakov I, Blumenfeld M, Guerassimenko O, Cavarec L, Palicio M, Abderrahim H, et al. Genetic and physiological data implicating the new human gene G72 and the gene for D-amino acid oxidase in schizophrenia. Proc Natl Acad Sci U S A. 2002;99(21):13675-80. doi: 10.1073/pnas.182412499. PubMed PMID: 12364586; PubMed Central PMCID: PMCPMC129739.

248. Soler J, Miret S, Lazaro L, Parellada M, Martin M, Lera-Miguel S, et al. Influence of DAOA and RGS4 genes on the risk for psychotic disorders and their associated executive dysfunctions: A family-based study. Eur Psychiatry. 2016;32:42-7. doi: 10.1016/j.eurpsy.2015.11.002. PubMed PMID: 26803614.

249. Wang KS, Liu XF, Aragam N. A genome-wide meta-analysis identifies novel loci associated with schizophrenia and bipolar disorder. Schizophr Res. 2010;124(1-3):192-9. doi: 10.1016/j.schres.2010.09.002. PubMed PMID: 20889312.

250. Wolthusen RP, Hass J, Walton E, Turner JA, Rossner V, Sponheim SR, et al. Genetic underpinnings of left superior temporal gyrus thickness in patients with schizophrenia. World J Biol Psychiatry. 2015:1-11. PubMed PMID: 26249676; PubMed Central PMCID: PMCPMC4795983.

251. Sung YJ, Perusse L, Sarzynski MA, Fornage M, Sidney S, Sternfeld B, et al. Genome-wide association studies suggest sex-specific loci associated with abdominal and visceral fat. Int J Obes (Lond). 2016;40(4):662-74. doi: 10.1038/ijo.2015.217. PubMed PMID: 26480920; PubMed Central PMCID: PMCPMC4821694.

252. Comuzzie AG, Cole SA, Laston SL, Voruganti VS, Haack K, Gibbs RA, et al. Novel genetic loci identified for the pathophysiology of childhood obesity in the Hispanic population. PLoS One. 2012;7(12):e51954. doi: 10.1371/journal.pone.0051954. PubMed PMID: 23251661; PubMed Central PMCID: PMCPMC3522587.

253. Argos M, Tong L, Pierce BL, Rakibuz-Zaman M, Ahmed A, Islam T, et al. Genome-wide association study of smoking behaviours among Bangladeshi adults. J Med Genet. 2014;51(5):327-33. doi: 10.1136/jmedgenet-2013-102151. PubMed PMID: 24665060; PubMed Central PMCID: PMCPMC4126189.

254. Low SK, Chung S, Takahashi A, Zembutsu H, Mushiroda T, Kubo M, et al. Genome-wide association study of chemotherapeutic agent-induced severe neutropenia/leucopenia for patients in Biobank Japan. Cancer Sci. 2013;104(8):1074-82. doi: 10.1111/cas.12186. PubMed PMID: 23648065.

255. Kennedy RB, Ovsyannikova IG, Pankratz VS, Haralambieva IH, Vierkant RA, Poland GA. Genome-wide analysis of polymorphisms associated with cytokine responses in smallpox vaccine recipients. Hum Genet. 2012;131(9):1403-21. doi: 10.1007/s00439-012-1174-2. PubMed PMID: 22610502; PubMed Central PMCID: PMCPMC4170585.

256. Mauer J, Luo X, Blanjoie A, Jiao X, Grozhik AV, Patil DP, et al. Reversible methylation of m6Am in the 5' cap controls mRNA stability. Nature. 2017;541(7637):371-5. doi: 10.1038/nature21022. PubMed PMID: 28002401.

257. Phani NM, Vohra M, Rajesh S, Adhikari P, Nagri SK, D'Souza SC, et al. Implications of critical PPARgamma2, ADIPOQ and FTO gene polymorphisms in type 2 diabetes and obesity-mediated susceptibility to type 2 diabetes in an Indian population. Mol Genet Genomics. 2016;291(1):193-204. doi: 10.1007/s00438-015-1097-4. PubMed PMID: 26243686.

258. Tews D, Fischer-Posovszky P, Fromme T, Klingenspor M, Fischer J, Ruther U, et al. FTO deficiency induces UCP-1 expression and mitochondrial uncoupling in adipocytes. Endocrinology. 2013;154(9):3141-51. doi: 10.1210/en.2012-1873. PubMed PMID: 23751871.

259. Aijala M, Ronkainen J, Huusko T, Malo E, Savolainen ER, Savolainen MJ, et al. The fat mass and obesity-associated (FTO) gene variant rs9939609 predicts long-term incidence of cardiovascular disease and related death independent of the traditional risk factors. Ann Med. 2015;47(8):655-63. doi: 10.3109/07853890.2015.1091088. PubMed PMID: 26555680.

260. Loos RJ, Yeo GS. The bigger picture of FTO: the first GWAS-identified obesity gene. Nat Rev Endocrinol. 2014;10(1):51-61. doi: 10.1038/nrendo.2013.227. PubMed PMID: 24247219; PubMed Central PMCID: PMCPMC4188449.

261. Claussnitzer M, Dankel SN, Kim KH, Quon G, Meuleman W, Haugen C, et al. FTO Obesity Variant Circuitry and Adipocyte Browning in Humans. N Engl J Med. 2015;373(10):895-907. doi: 10.1056/NEJMoa1502214. PubMed PMID: 26287746; PubMed Central PMCID: PMCPMC4959911.

262. Claussnitzer M, Hui CC, Kellis M. FTO Obesity Variant and Adipocyte Browning in Humans. N Engl J Med. 2016;374(2):192-3. doi: 10.1056/NEJMc1513316. PubMed PMID: 26760096.

263. Binh TQ, Phuong PT, Nhung BT, Thoang DD, Lien HT, Thanh DV. Association of the common FTO-rs9939609 polymorphism with type 2 diabetes, independent of obesity-related traits in a Vietnamese population. Gene. 2013;513(1):31-5. doi: 10.1016/j.gene.2012.10.082. PubMed PMID: 23142383.

264. Amare AT, Schubert KO, Klingler-Hoffmann M, Cohen-Woods S, Baune BT. The genetic overlap between mood disorders and cardiometabolic diseases: a systematic review of genome wide and candidate gene studies. Transl Psychiatry. 2017;7(1):e1007. doi: 10.1038/tp.2016.261. PubMed PMID: 28117839.

265. Samaan Z, Anand SS, Zhang X, Desai D, Rivera M, Pare G, et al. The protective effect of the obesity-associated rs9939609 A variant in fat mass- and obesity-associated gene on depression. Mol Psychiatry. 2013;18(12):1281-6. doi: 10.1038/mp.2012.160. PubMed PMID: 23164817.

266. Keller L, Xu W, Wang HX, Winblad B, Fratiglioni L, Graff C. The obesity related gene, FTO, interacts with APOE, and is associated with Alzheimer's disease risk: a prospective cohort study. J Alzheimers Dis. 2011;23(3):461-9. doi: 10.3233/JAD-2010-101068. PubMed PMID: 21098976.

267. Corella D, Ortega-Azorin C, Sorli JV, Covas MI, Carrasco P, Salas-Salvado J, et al. Statistical and biological gene-lifestyle interactions of MC4R and FTO with diet and physical activity on obesity: new effects on alcohol consumption. PLoS One. 2012;7(12):e52344. doi: 10.1371/journal.pone.0052344. PubMed PMID: 23284998; PubMed Central PMCID: PMCPMC3528751.

268. Sobczyk-Kopciol A, Broda G, Wojnar M, Kurjata P, Jakubczyk A, Klimkiewicz A, et al. Inverse association of the obesity predisposing FTO rs9939609 genotype with alcohol consumption and risk for alcohol dependence. Addiction. 2011;106(4):739-48. doi: 10.1111/j.1360-0443.2010.03248.x. PubMed PMID: 21182554.

269. Young AI, Wauthier F, Donnelly P. Multiple novel gene-by-environment interactions modify the effect of FTO variants on body mass index. Nat Commun. 2016;7:12724. Epub 2016/09/07. doi: 10.1038/ncomms12724. PubMed PMID: 27596730; PubMed Central PMCID: PMCPMC5025863 LLP. The remaining authors declare no competing financial interests.

270. Garcia-Solis P, Reyes-Bastidas M, Flores K, Garcia OP, Rosado JL, Mendez-Villa L, et al. Fat mass obesity-associated (FTO) (rs9939609) and melanocortin 4 receptor (MC4R) (rs17782313) SNP are positively associated with obesity and blood pressure in Mexican school-aged children. Br J Nutr. 2016:1-7. doi: 10.1017/S0007114516003779. PubMed PMID: 27829468.

271. Xi B, Zhao X, Shen Y, Wu L, Hotta K, Hou D, et al. Associations of obesity susceptibility loci with hypertension in Chinese children. Int J Obes (Lond). 2013;37(7):926-30. doi: 10.1038/ijo.2013.37. PubMed PMID: 23588626.

272. Monda KL, Chen GK, Taylor KC, Palmer C, Edwards TL, Lange LA, et al. A meta-analysis identifies new loci associated with body mass index in individuals of African ancestry. Nat Genet. 2013;45(6):690-6. doi: 10.1038/ng.2608. PubMed PMID: 23583978; PubMed Central PMCID: PMCPMC3694490.

273. Yang J, Loos RJ, Powell JE, Medland SE, Speliotes EK, Chasman DI, et al. FTO genotype is associated with phenotypic variability of body mass index. Nature. 2012;490(7419):267-72. doi: 10.1038/nature11401. PubMed PMID: 22982992; PubMed Central PMCID: PMCPMC3564953.

274. Wen W, Zheng W, Okada Y, Takeuchi F, Tabara Y, Hwang JY, et al. Meta-analysis of genome-wide association studies in East Asian-ancestry populations identifies four new loci for body mass index. Hum Mol Genet. 2014;23(20):5492-504. doi: 10.1093/hmg/ddu248. PubMed PMID: 24861553; PubMed Central PMCID: PMCPMC4168820.

275. Locke AE, Kahali B, Berndt SI, Justice AE, Pers TH, Day FR, et al. Genetic studies of body mass index yield new insights for obesity biology. Nature. 2015;518(7538):197-206. doi: 10.1038/nature14177. PubMed PMID: 25673413; PubMed Central PMCID: PMCPMC4382211.

276. Warrington NM, Howe LD, Paternoster L, Kaakinen M, Herrala S, Huikari V, et al. A genome-wide association study of body mass index across early life and childhood. Int J Epidemiol. 2015;44(2):700-12. doi: 10.1093/ije/dyv077. PubMed PMID: 25953783; PubMed Central PMCID: PMCPMC4469798.

277. Felix JF, Bradfield JP, Monnereau C, van der Valk RJ, Stergiakouli E, Chesi A, et al. Genome-wide association analysis identifies three new susceptibility loci for childhood body mass index. Hum Mol Genet. 2016;25(2):389-403. doi: 10.1093/hmg/ddv472. PubMed PMID: 26604143; PubMed Central PMCID: PMCPMC4854022.

278. Graff M, Ngwa JS, Workalemahu T, Homuth G, Schipf S, Teumer A, et al. Genome-wide analysis of BMI in adolescents and young adults reveals additional insight into the effects of genetic loci over the life course. Hum Mol Genet. 2013;22(17):3597-607. doi: 10.1093/hmg/ddt205. PubMed PMID: 23669352; PubMed Central PMCID: PMCPMC3736869.

279. Okada Y, Kubo M, Ohmiya H, Takahashi A, Kumasaka N, Hosono N, et al. Common variants at CDKAL1 and KLF9 are associated with body mass index in east Asian populations. Nat Genet. 2012;44(3):302-6. doi: 10.1038/ng.1086. PubMed PMID: 22344221; PubMed Central PMCID: PMCPMC3838874.

280. Frayling TM, Timpson NJ, Weedon MN, Zeggini E, Freathy RM, Lindgren CM, et al. A common variant in the FTO gene is associated with body mass index and predisposes to childhood and adult obesity. Science. 2007;316(5826):889-94. doi: 10.1126/science.1141634. PubMed PMID: 17434869; PubMed Central PMCID: PMCPMC2646098.

281. Willer CJ, Speliotes EK, Loos RJ, Li S, Lindgren CM, Heid IM, et al. Six new loci associated with body mass index highlight a neuronal influence on body weight regulation. Nat Genet. 2009;41(1):25-34. doi: 10.1038/ng.287. PubMed PMID: 19079261; PubMed Central PMCID: PMCPMC2695662.

282. Thorleifsson G, Walters GB, Gudbjartsson DF, Steinthorsdottir V, Sulem P, Helgadottir A, et al. Genome-wide association yields new sequence variants at seven loci that associate with measures of obesity. Nat Genet. 2009;41(1):18-24. doi: 10.1038/ng.274. PubMed PMID: 19079260.

283. Wheeler E, Huang N, Bochukova EG, Keogh JM, Lindsay S, Garg S, et al. Genome-wide SNP and CNV analysis identifies common and low-frequency variants associated with severe early-onset obesity. Nat Genet. 2013;45(5):513-7. doi: 10.1038/ng.2607. PubMed PMID: 23563609; PubMed Central PMCID: PMCPMC4106235.

284. Meyre D, Delplanque J, Chevre JC, Lecoeur C, Lobbens S, Gallina S, et al. Genome-wide association study for early-onset and morbid adult obesity identifies three new risk loci in European populations. Nat Genet. 2009;41(2):157-9. doi: 10.1038/ng.301. PubMed PMID: 19151714.

285. Lu Y, Day FR, Gustafsson S, Buchkovich ML, Na J, Bataille V, et al. New loci for body fat percentage reveal link between adiposity and cardiometabolic disease risk. Nat Commun. 2016;7:10495. doi: 10.1038/ncomms10495. PubMed PMID: 26833246; PubMed Central PMCID: PMCPMC4740398.

286. Heard-Costa NL, Zillikens MC, Monda KL, Johansson A, Harris TB, Fu M, et al. NRXN3 is a novel locus for waist circumference: a genome-wide association study from the CHARGE Consortium. PLoS Genet. 2009;5(6):e1000539. doi: 10.1371/journal.pgen.1000539. PubMed PMID: 19557197; PubMed Central PMCID: PMCPMC2695005.

287. Kilpelainen TO, Zillikens MC, Stancakova A, Finucane FM, Ried JS, Langenberg C, et al. Genetic variation near IRS1 associates with reduced adiposity and an impaired metabolic profile. Nat Genet. 2011;43(8):753-60. doi: 10.1038/ng.866. PubMed PMID: 21706003; PubMed Central PMCID: PMCPMC3262230.

288. Kilpelainen TO, Carli JF, Skowronski AA, Sun Q, Kriebel J, Feitosa MF, et al. Genome-wide meta-analysis uncovers novel loci influencing circulating leptin levels. Nat Commun. 2016;7:10494. doi: 10.1038/ncomms10494. PubMed PMID: 26833098; PubMed Central PMCID: PMCPMC4740377.

289. Scott LJ, Mohlke KL, Bonnycastle LL, Willer CJ, Li Y, Duren WL, et al. A genome-wide association study of type 2 diabetes in Finns detects multiple susceptibility variants. Science. 2007;316(5829):1341-5. doi: 10.1126/science.1142382. PubMed PMID: 17463248; PubMed Central PMCID: PMCPMC3214617.

290. Perry JR, Voight BF, Yengo L, Amin N, Dupuis J, Ganser M, et al. Stratifying type 2 diabetes cases by BMI identifies genetic risk variants in LAMA1 and enrichment for risk variants in lean compared to obese cases. PLoS Genet. 2012;8(5):e1002741. doi: 10.1371/journal.pgen.1002741. PubMed PMID: 22693455; PubMed Central PMCID: PMCPMC3364960.

291. Zeggini E, Weedon MN, Lindgren CM, Frayling TM, Elliott KS, Lango H, et al. Replication of genome-wide association signals in UK samples reveals risk loci for type 2 diabetes. Science. 2007;316(5829):1336-41. doi: 10.1126/science.1142364. PubMed PMID: 17463249; PubMed Central PMCID: PMCPMC3772310.

292. Tanaka T, Ngwa JS, van Rooij FJ, Zillikens MC, Wojczynski MK, Frazier-Wood AC, et al. Genome-wide meta-analysis of observational studies shows common genetic variants associated with macronutrient intake. Am J Clin Nutr. 2013;97(6):1395-402. doi: 10.3945/ajcn.112.052183. PubMed PMID: 23636237; PubMed Central PMCID: PMCPMC3652928.

293. Elks CE, Perry JR, Sulem P, Chasman DI, Franceschini N, He C, et al. Thirty new loci for age at menarche identified by a meta-analysis of genome-wide association studies. Nat Genet. 2010;42(12):1077-85. doi: 10.1038/ng.714. PubMed PMID: 21102462; PubMed Central PMCID: PMCPMC3140055.

294. Cheong KA, Kim NH, Noh M, Lee AY. Three new single nucleotide polymorphisms identified by a genome-wide association study in Korean patients with vitiligo. J Korean Med Sci. 2013;28(5):775-9. doi: 10.3346/jkms.2013.28.5.775. PubMed PMID: 23678272; PubMed Central PMCID: PMCPMC3653093.

295. Michailidou K, Hall P, Gonzalez-Neira A, Ghoussaini M, Dennis J, Milne RL, et al. Large-scale genotyping identifies 41 new loci associated with breast cancer risk. Nat Genet. 2013;45(4):353-61, 61e1-2. doi: 10.1038/ng.2563. PubMed PMID: 23535729; PubMed Central PMCID: PMCPMC3771688.

296. Garcia-Closas M, Couch FJ, Lindstrom S, Michailidou K, Schmidt MK, Brook MN, et al. Genome-wide association studies identify four ER negative-specific breast cancer risk loci. Nat Genet. 2013;45(4):392-8, 8e1-2. doi: 10.1038/ng.2561. PubMed PMID: 23535733; PubMed Central PMCID: PMCPMC3771695.

297. Iles MM, Law MH, Stacey SN, Han J, Fang S, Pfeiffer R, et al. A variant in FTO shows association with melanoma risk not due to BMI. Nat Genet. 2013;45(4):428-32, 32e1. doi: 10.1038/ng.2571. PubMed PMID: 23455637; PubMed Central PMCID: PMCPMC3640814.

298. Ghosh T, Aprea J, Nardelli J, Engel H, Selinger C, Mombereau C, et al. MicroRNAs establish robustness and adaptability of a critical gene network to regulate progenitor fate decisions during cortical neurogenesis. Cell Rep. 2014;7(6):1779-88. doi: 10.1016/j.celrep.2014.05.029. PubMed PMID: 24931612.

299. Landgraf P, Rusu M, Sheridan R, Sewer A, Iovino N, Aravin A, et al. A mammalian microRNA expression atlas based on small RNA library sequencing. Cell. 2007;129(7):1401-14. doi: 10.1016/j.cell.2007.04.040. PubMed PMID: 17604727; PubMed Central PMCID: PMCPMC2681231.

300. Kim K, Vinayagam A, Perrimon N. A rapid genome-wide microRNA screen identifies miR-14 as a modulator of Hedgehog signaling. Cell Rep. 2014;7(6):2066-77. doi: 10.1016/j.celrep.2014.05.025. PubMed PMID: 24931604; PubMed Central PMCID: PMCPMC4142207.

301. Griffiths-Jones S, Grocock RJ, van Dongen S, Bateman A, Enright AJ. miRBase: microRNA sequences, targets and gene nomenclature. Nucleic Acids Res. 2006;34(Database issue):D140-4. doi: 10.1093/nar/gkj112. PubMed PMID: 16381832; PubMed Central PMCID: PMCPMC1347474.

302. Lin Z, Song D, Wei H, Yang X, Liu T, Yan W, et al. TGF-beta1-induced miR-202 mediates drug resistance by inhibiting apoptosis in human osteosarcoma. J Cancer Res Clin Oncol. 2016;142(1):239-46. doi: 10.1007/s00432-015-2028-9. PubMed PMID: 26276504.

303. Rudkowska I, Perusse L, Bellis C, Blangero J, Despres JP, Bouchard C, et al. Interaction between Common Genetic Variants and Total Fat Intake on Low-Density Lipoprotein Peak Particle Diameter: A Genome-Wide Association Study. J Nutrigenet Nutrigenomics. 2015;8(1):44-53. doi: 10.1159/000431151. PubMed PMID: 26112879.

304. Greenwood TA, Akiskal HS, Akiskal KK, Bipolar Genome S, Kelsoe JR. Genome-wide association study of temperament in bipolar disorder reveals significant associations with three novel Loci. Biol Psychiatry. 2012;72(4):303-10. doi: 10.1016/j.biopsych.2012.01.018. PubMed PMID: 22365631; PubMed Central PMCID: PMCPMC3925336.

305. Kajiwara Y, Akram A, Katsel P, Haroutunian V, Schmeidler J, Beecham G, et al. FE65 binds Teashirt, inhibiting expression of the primate-specific caspase-4. PLoS One. 2009;4(4):e5071. doi: 10.1371/journal.pone.0005071. PubMed PMID: 19343227; PubMed Central PMCID: PMCPMC2660419.

306. Caubit X, Thoby-Brisson M, Voituron N, Filippi P, Bevengut M, Faralli H, et al. Teashirt 3 regulates development of neurons involved in both respiratory rhythm and airflow control. J Neurosci. 2010;30(28):9465-76. doi: 10.1523/JNEUROSCI.1765-10.2010. PubMed PMID: 20631175.

307. Lye CM, Fasano L, Woolf AS. Ureter myogenesis: putting Teashirt into context. J Am Soc Nephrol. 2010;21(1):24-30. doi: 10.1681/ASN.2008111206. PubMed PMID: 19926888.

308. Luca G, Haba-Rubio J, Dauvilliers Y, Lammers GJ, Overeem S, Donjacour CE, et al. Clinical, polysomnographic and genome-wide association analyses of narcolepsy with cataplexy: a European Narcolepsy Network study. J Sleep Res. 2013;22(5):482-95. doi: 10.1111/jsr.12044. PubMed PMID: 23496005.

309. Prescott J, Thompson DJ, Kraft P, Chanock SJ, Audley T, Brown J, et al. Genome-wide association study of circulating estradiol, testosterone, and sex hormone-binding globulin in postmenopausal women. PLoS One. 2012;7(6):e37815. doi: 10.1371/journal.pone.0037815. PubMed PMID: 22675492; PubMed Central PMCID: PMCPMC3366971.

310. Ganesh SK, Tragante V, Guo W, Guo Y, Lanktree MB, Smith EN, et al. Loci influencing blood pressure identified using a cardiovascular gene-centric array. Hum Mol Genet. 2013;22(8):1663-78. doi: 10.1093/hmg/dds555. PubMed PMID: 23303523; PubMed Central PMCID: PMCPMC3657476.

311. Tragante V, Barnes MR, Ganesh SK, Lanktree MB, Guo W, Franceschini N, et al. Gene-centric meta-analysis in 87,736 individuals of European ancestry identifies multiple blood-pressure-related loci. Am J Hum Genet. 2014;94(3):349-60. doi: 10.1016/j.ajhg.2013.12.016. PubMed PMID: 24560520; PubMed Central PMCID: PMCPMC3951943.
