# Supplementary figures and images for: Novel genetic associations for blood pressure identified via gene-alcohol interaction in up to 570K individuals across multiple ancestries
Source: PLoS One. 2018 Jun 18;13(6):e0198166. doi: 10.1371/journal.pone.0198166 (PMC6005576; doi:10.1371/journal.pone.0198166)

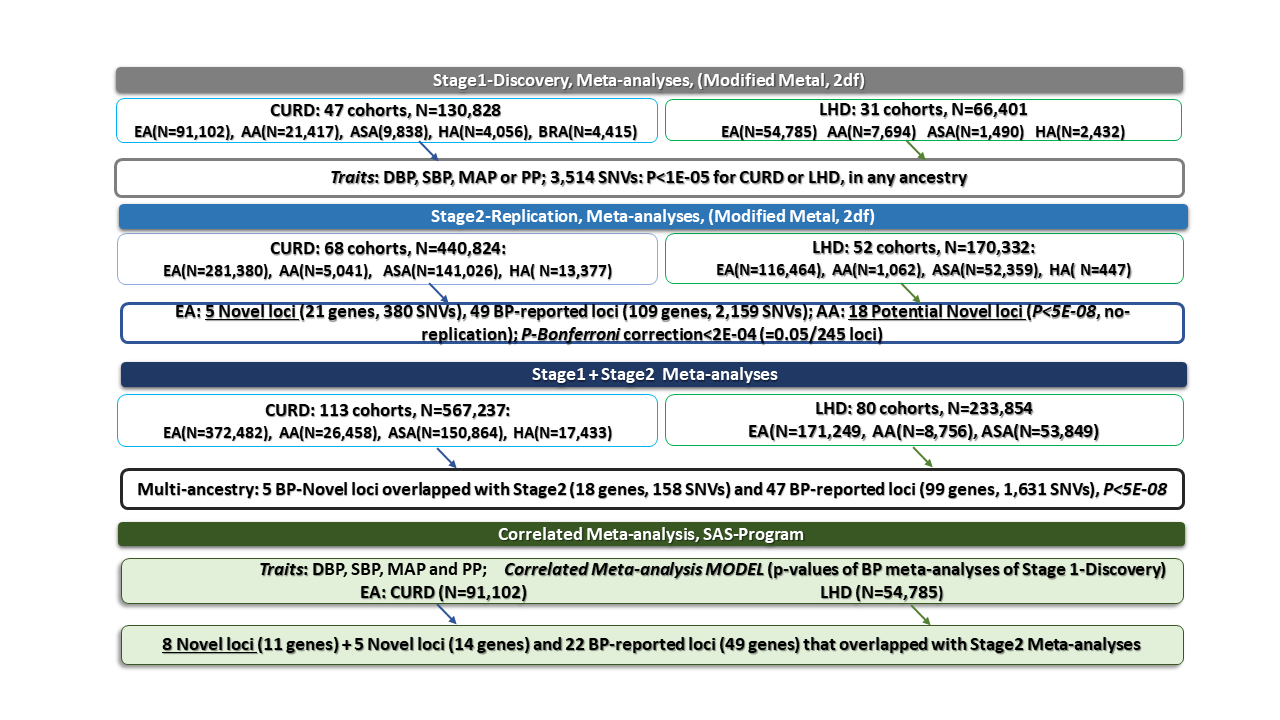

Supplement: S1 Fig — Schematic study design of the joint model of SNV main effect and SNV-alcohol consumption interaction; Blood pressure (BP) traits: systolic BP (SBP), diastolic BP (DBP), mean arterial pressure (MAP), and pulse pressure (PP); Alcohol consumption was defined by two categories: (I) as current drinking (yes/no), and (II), in the subset of drinkers, as light/heavy drinking (1–7 drinks/week or ≥8 drinks/week); Meta-analysis using a modified version of METAL: Stage 1 (discovery), Stage 2 (replication) and combined Stage 1 and Stage 2; Cohorts: European ancestry (EA), African ancestry, Asian ancestry (ASA), Hispanic ancestry (HA), Brazilian (BRA); Correlated meta-analysis in EA for four BP traits; Number of BP loci (genes), novel and reported. (TIF) [file pone.0198166.s003.TIF]

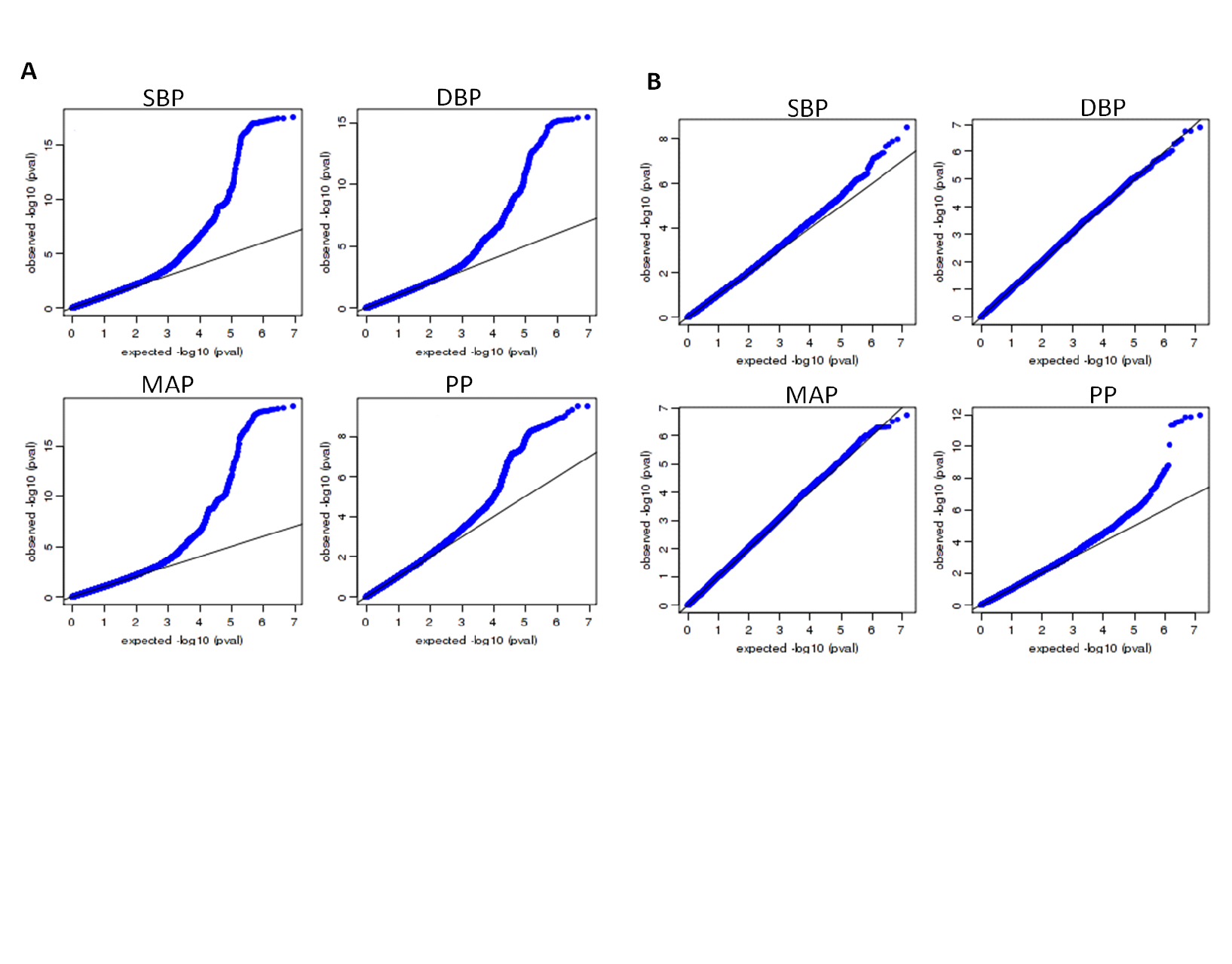

Supplement: S2 Fig — Meta-analysis distributions of–log10 P-values of observed versus–log10 P-values expected (QQ plots) for current drinkers (yes/no) European ancestry (A) and in African ancestry (B). (TIF) [file pone.0198166.s004.tif]

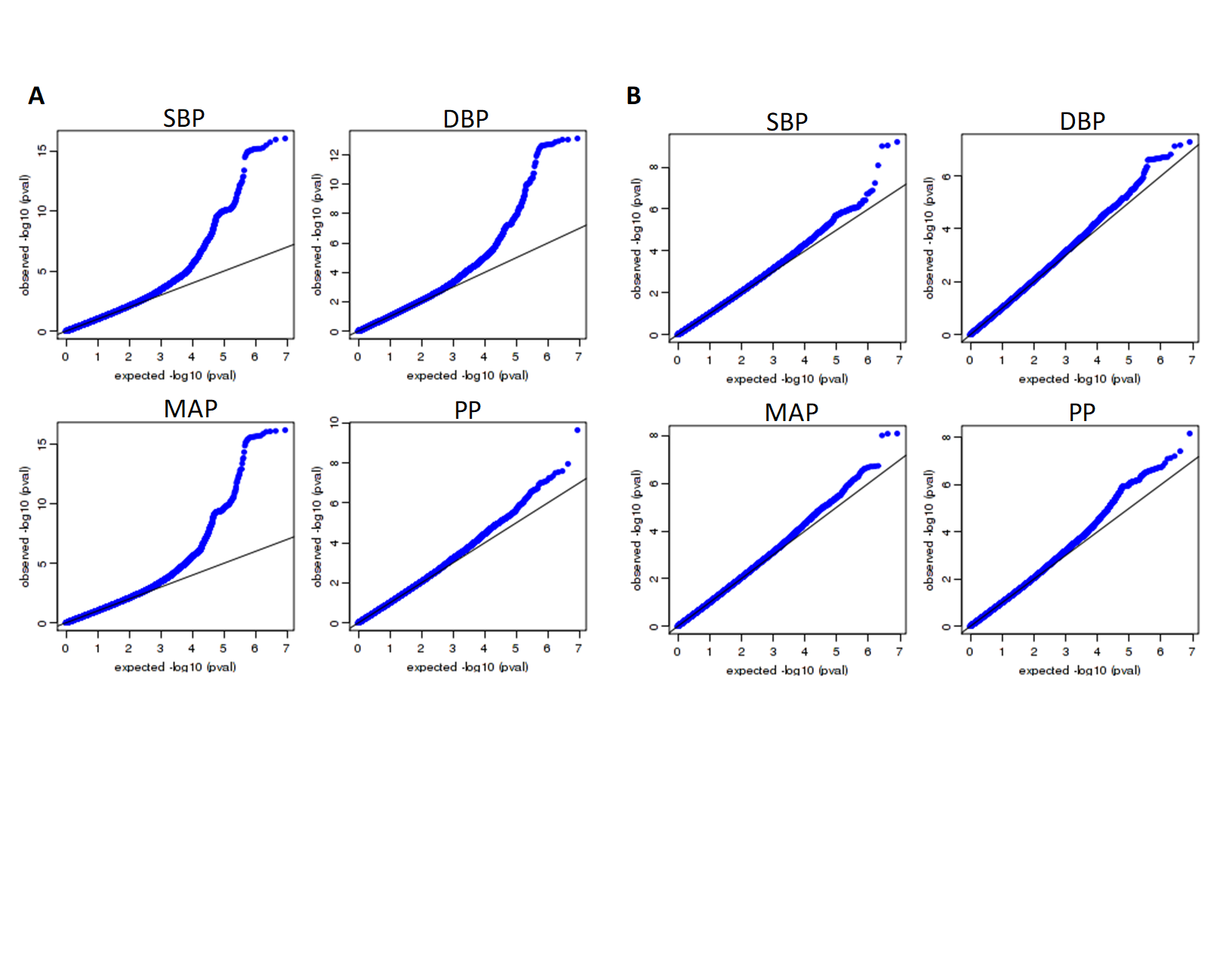

Supplement: S3 Fig — Meta-analysis distributions of–log10 P-values of observed versus–log10 P-values expected (QQ plots) for light/heavy drinkers (1–7 drinks/week or ≥8 drinks/week) in European ancestry (A) and in African ancestry (B). (TIF) [file pone.0198166.s005.TIF]

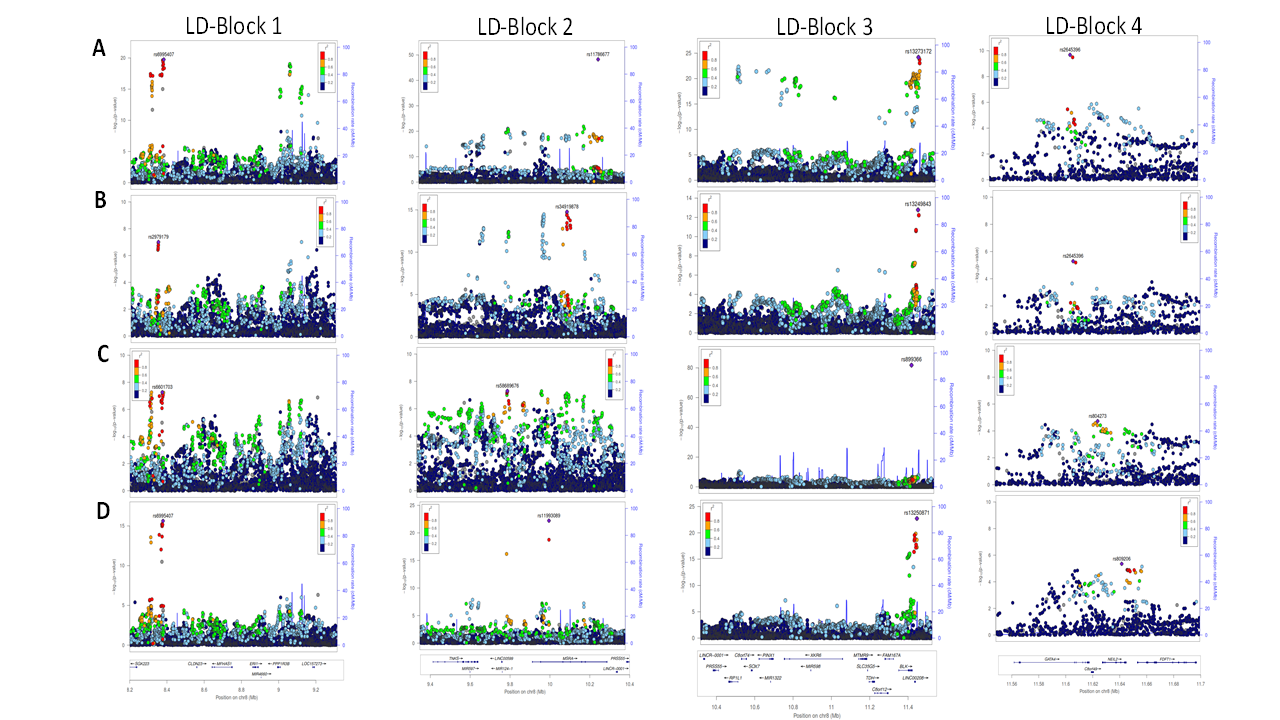

Supplement: S4 Fig — SNV x current drinker interaction for SBP (A), DBP (B), MAP (C) and PP (D) in European Ancestry; four linkage disequilibrium (LD) blocks (see also Fig 1). (TIF) [file pone.0198166.s006.tif]

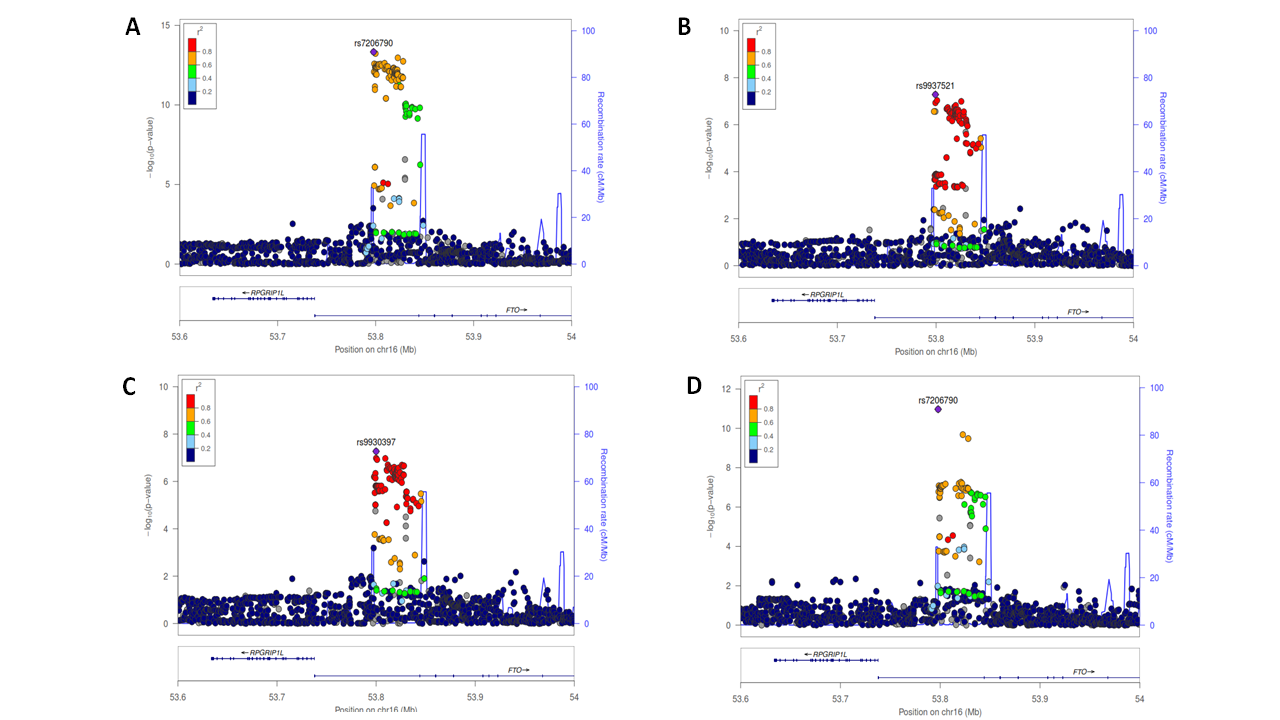

Supplement: S5 Fig — SNV x current drinker interaction for SBP (A), DBP (B), MAP (C) and PP (D) in European Ancestry. (TIF) [file pone.0198166.s007.tif]

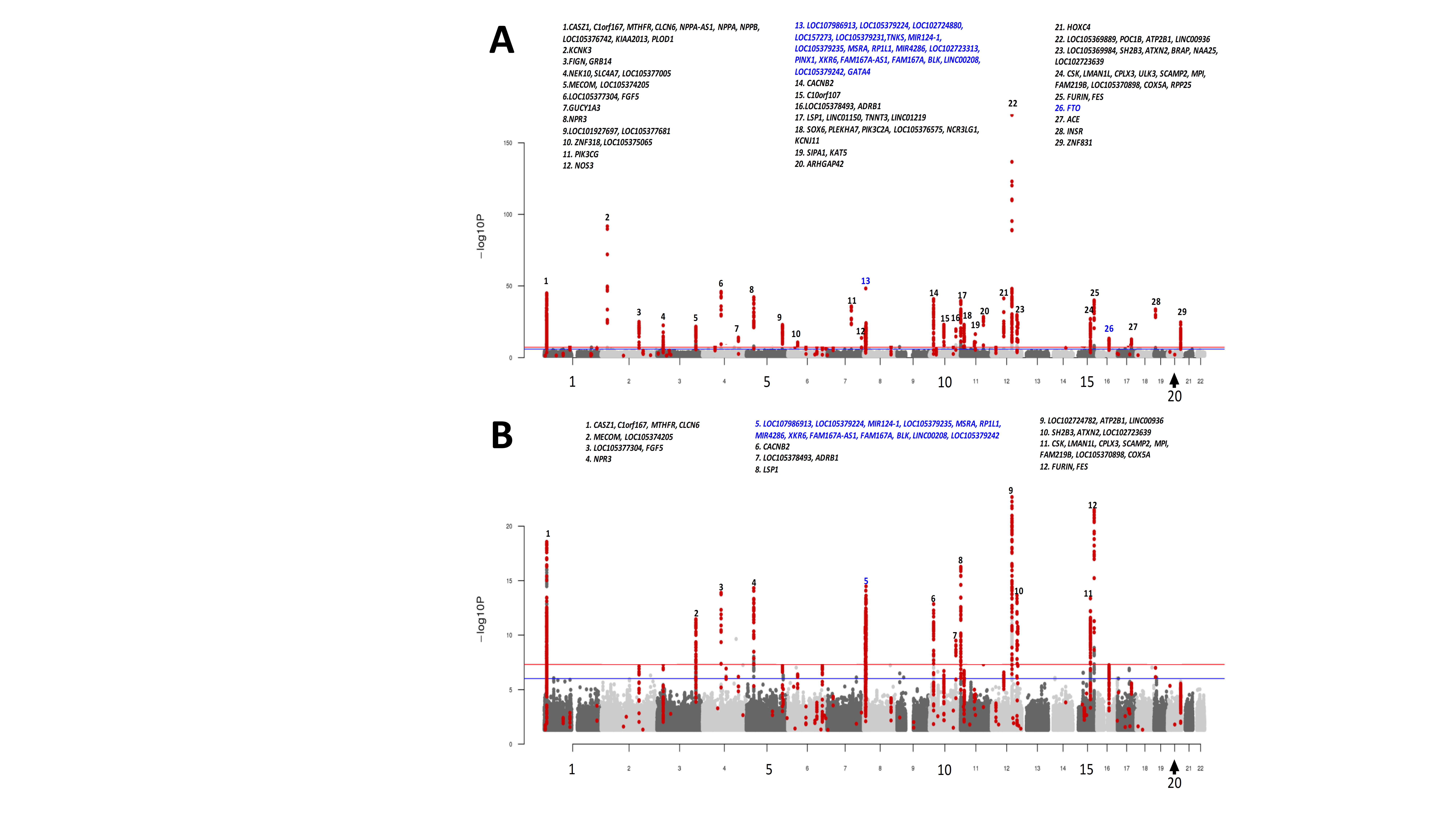

Supplement: S6 Fig — Manhattan plots of combined Stage 1 and Stage 2 meta-analysis for SBP in current drinkers (A) and in light/heavy drinkers (B) in European ancestry. Novel loci are highlighted in blue. (TIF) [file pone.0198166.s008.tif]

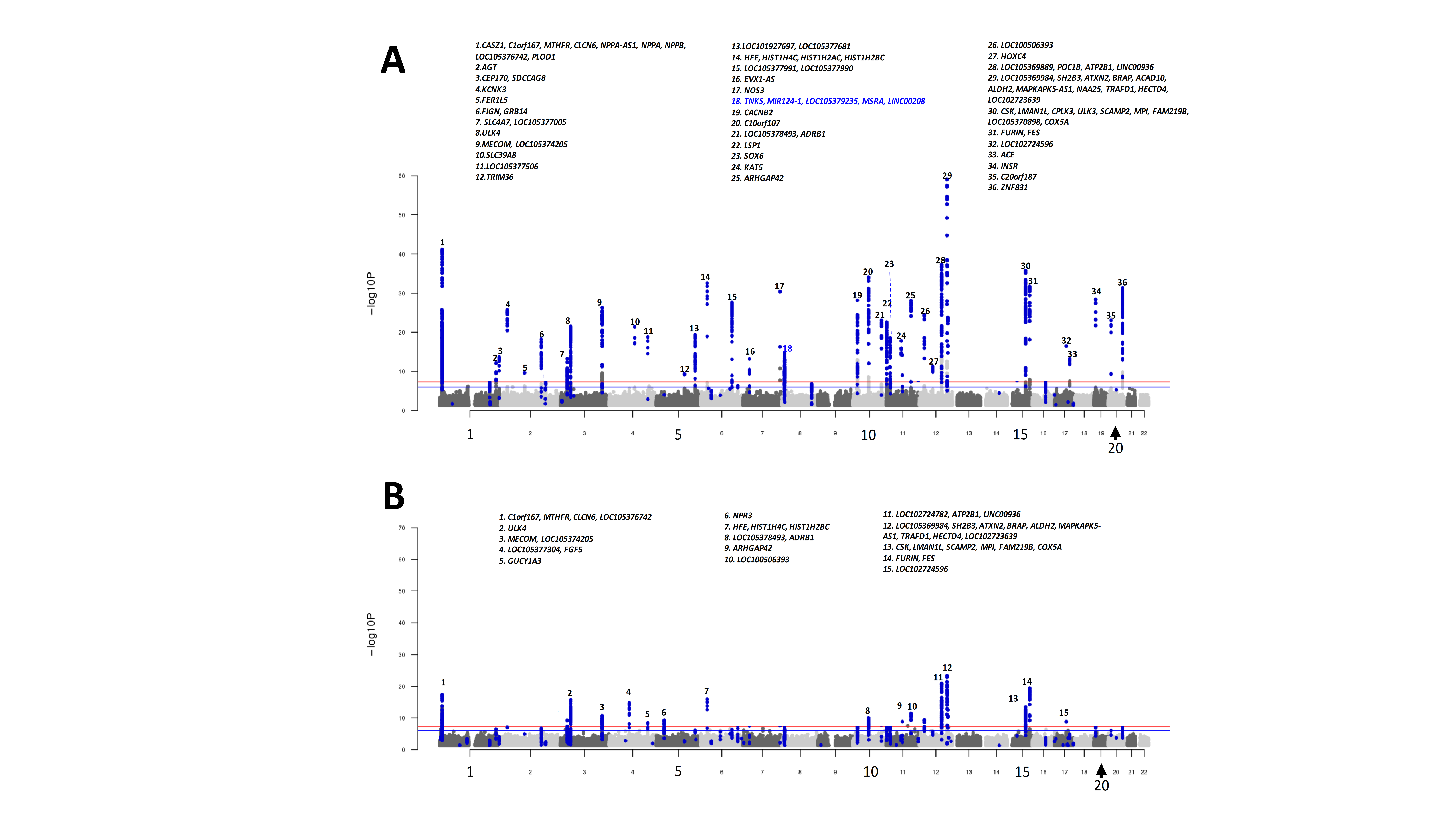

Supplement: S7 Fig — Manhattan plots of combined Stage 1 and Stage 2 meta-analysis for DBP in current drinkers (A) and in light/heavy drinkers (B) in European ancestry. Novel loci are highlighted in blue. (TIF) [file pone.0198166.s009.tif]

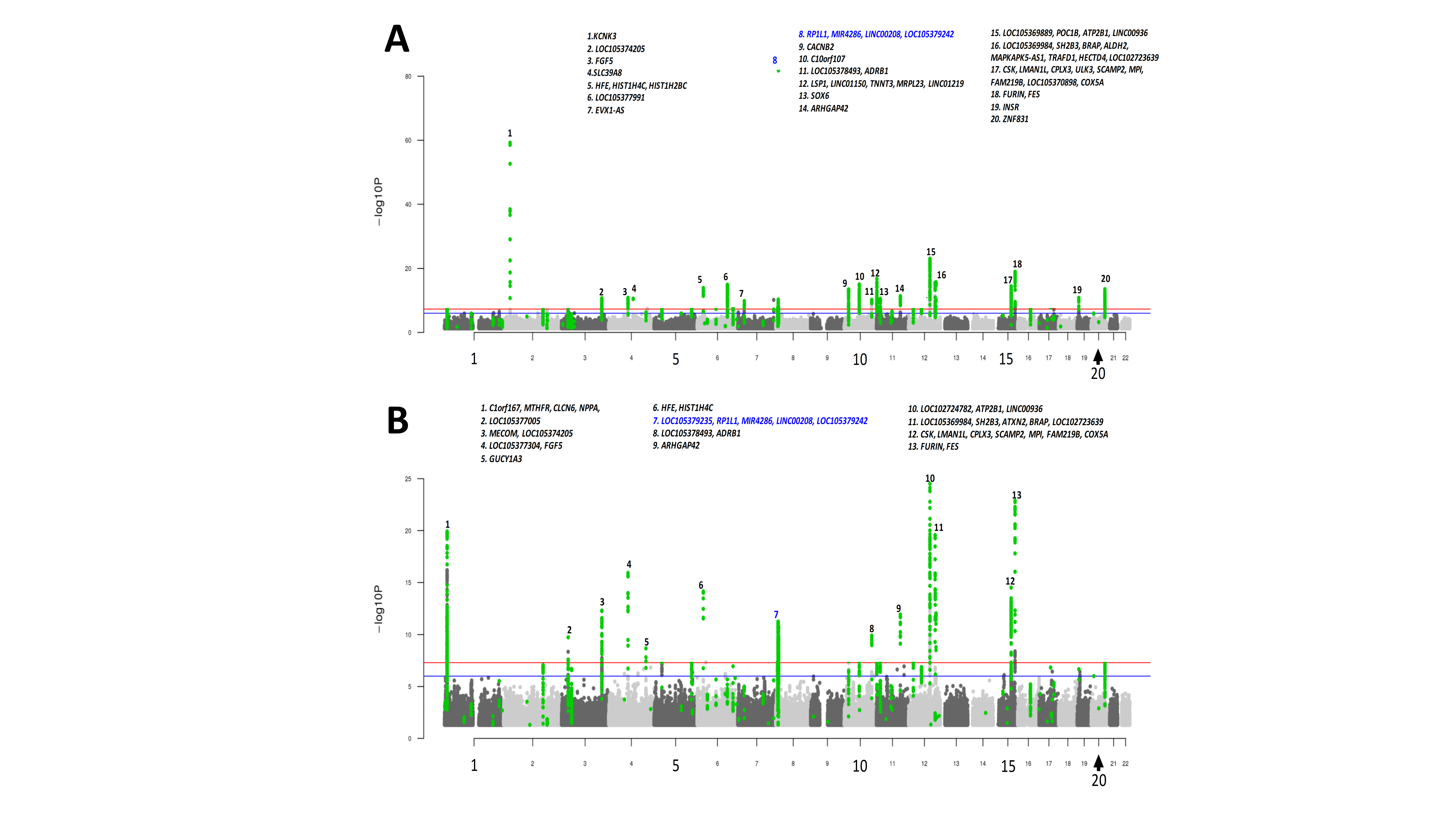

Supplement: S8 Fig — Manhattan plots of combined Stage 1 and Stage 2 meta-analysis for MAP in current drinkers (A) and in light/heavy drinkers (B) in European ancestry. Novel loci are highlighted in blue. (TIF) [file pone.0198166.s010.tif]

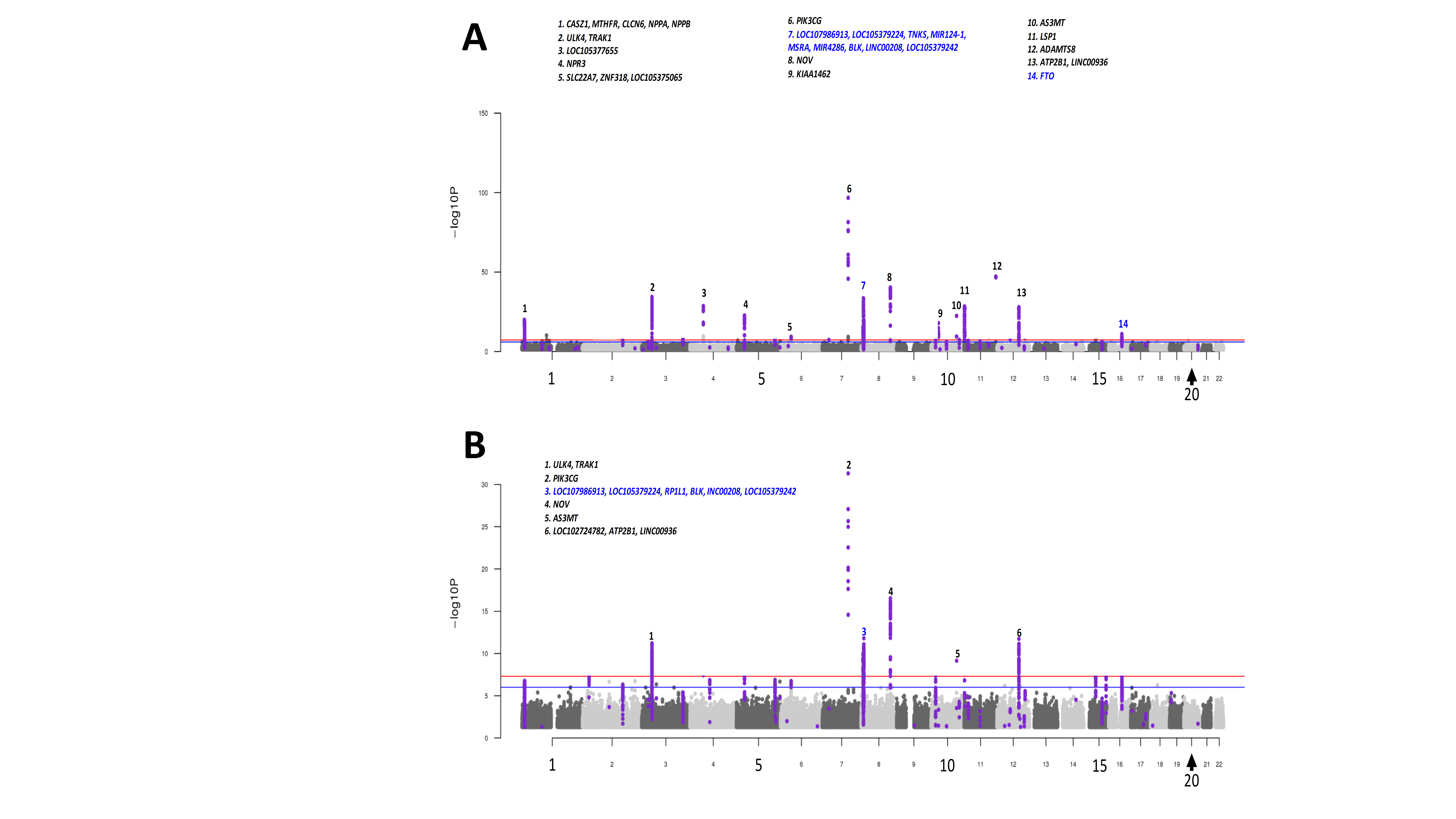

Supplement: S9 Fig — Manhattan plots of combined Stage 1 and Stage 2 meta-analysis for PP in current drinkers (A) and in light/heavy drinkers (B) in European ancestry. Novel loci are highlighted in blue. (TIF) [file pone.0198166.s011.tif]

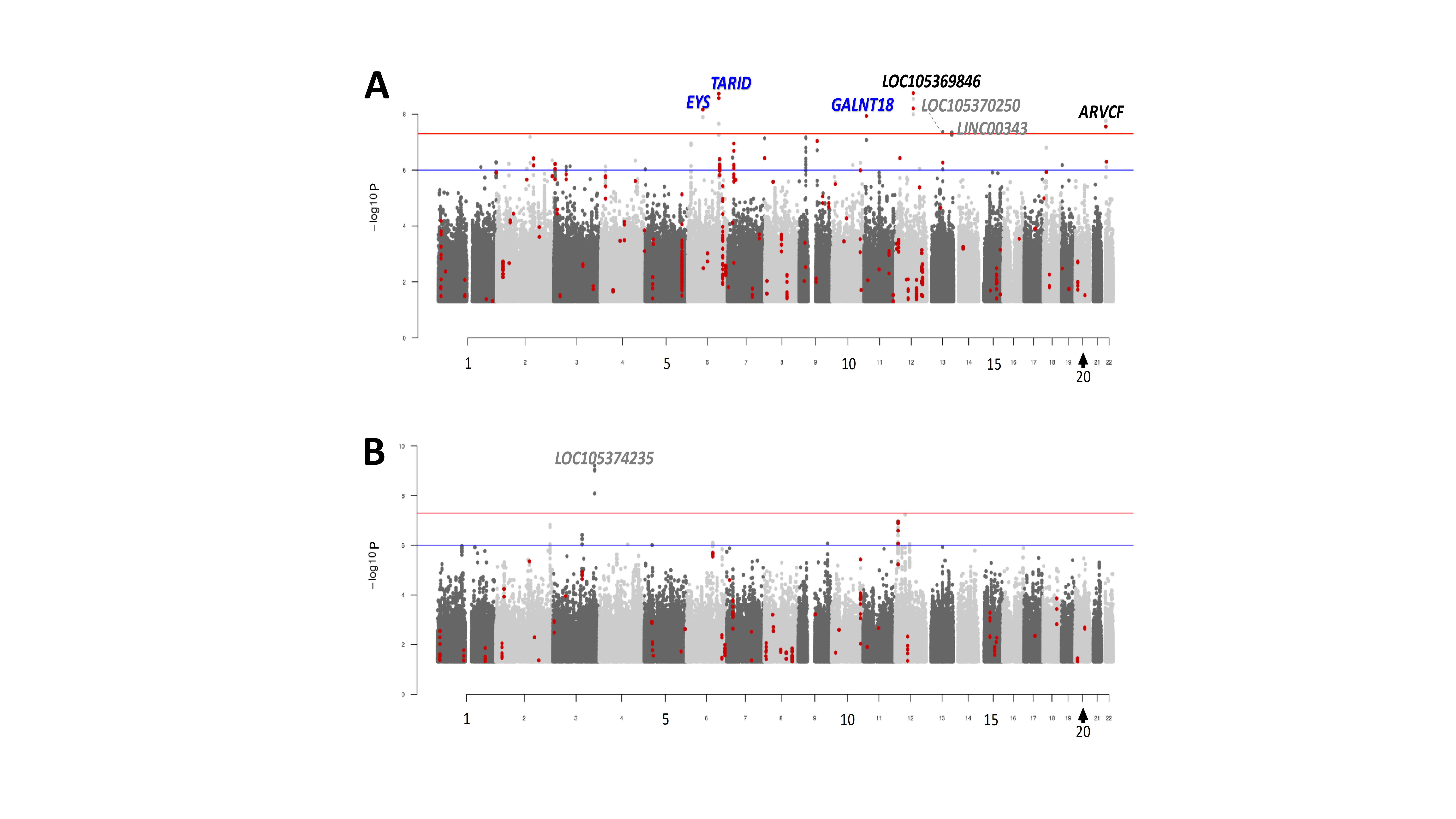

Supplement: S10 Fig — Manhattan plots of combined Stage 1 and Stage 2 meta-analysis for SBP in current drinkers (A) and in light/heavy drinkers (B) in African ancestry. Novel loci are highlighted in blue. (TIF) [file pone.0198166.s012.tif]

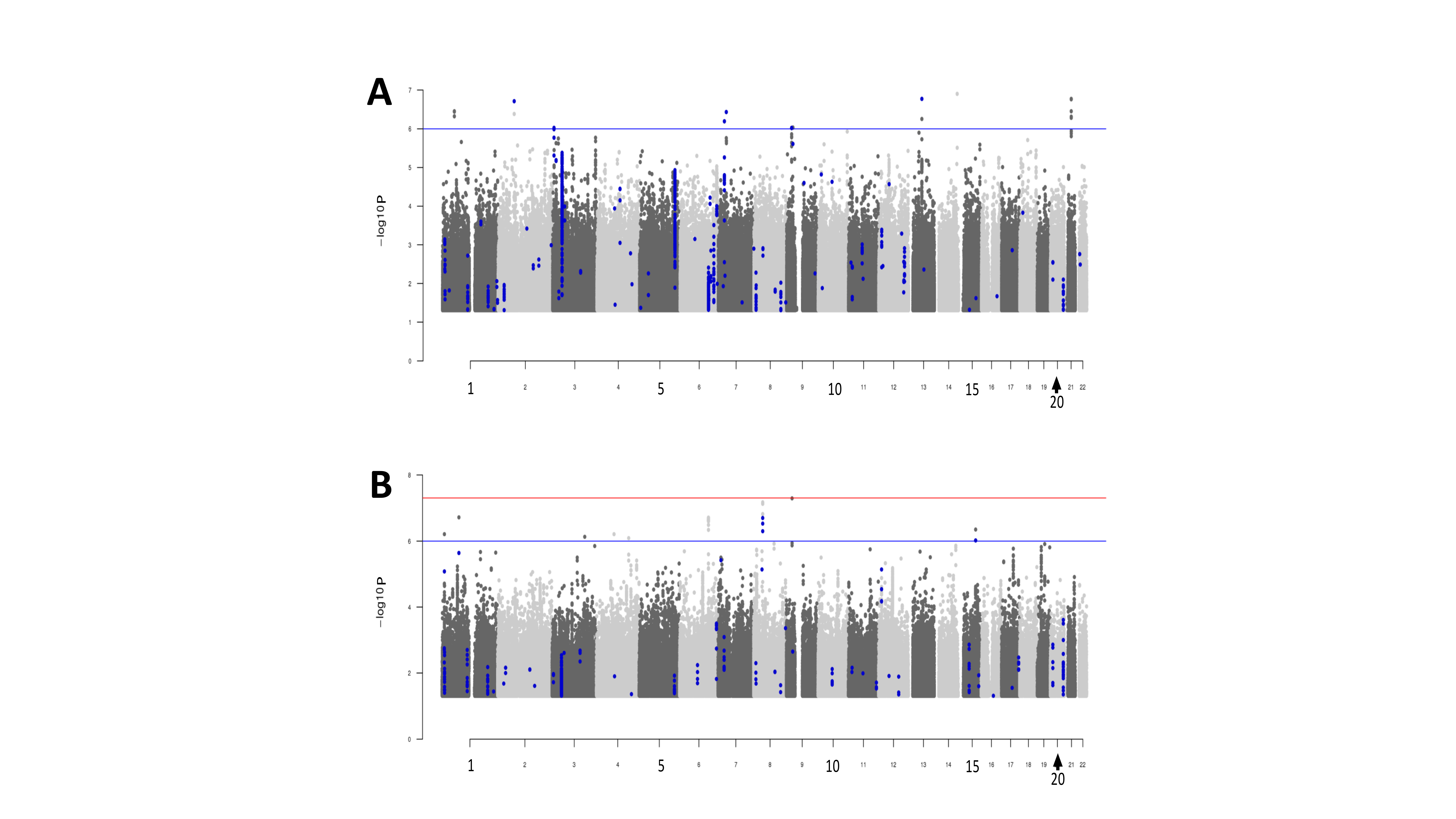

Supplement: S11 Fig — Manhattan plots of combined Stage 1 and Stage 2 meta-analysis for DBP in current drinkers (A) and in light/heavy drinkers (B) in African ancestry. (TIF) [file pone.0198166.s013.tif]

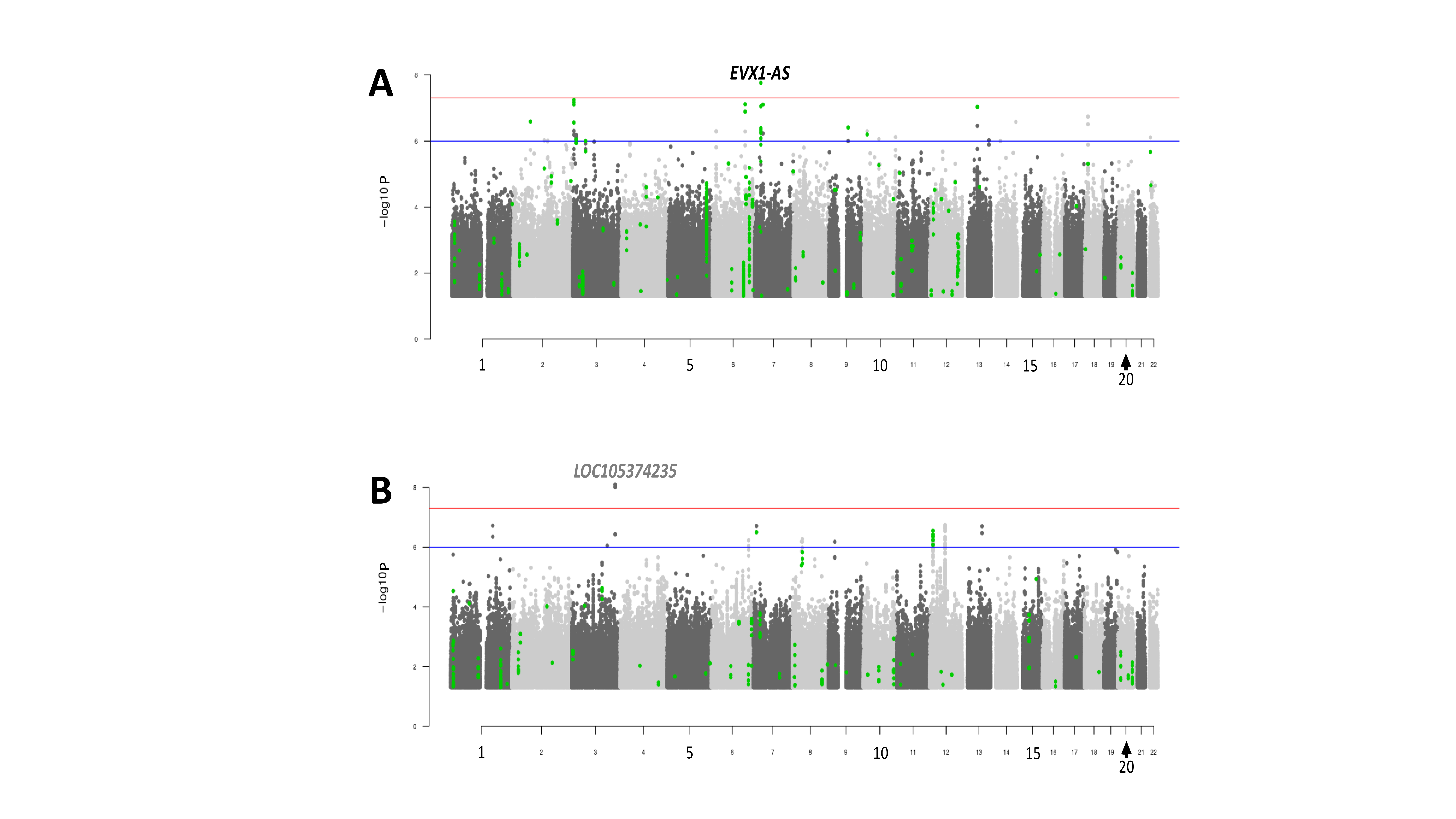

Supplement: S12 Fig — Manhattan plots of combined Stage 1 and Stage 2 meta-analysis for MAP in current drinkers (A) and in light/heavy drinkers (B) in African ancestry. (TIF) [file pone.0198166.s014.tif]

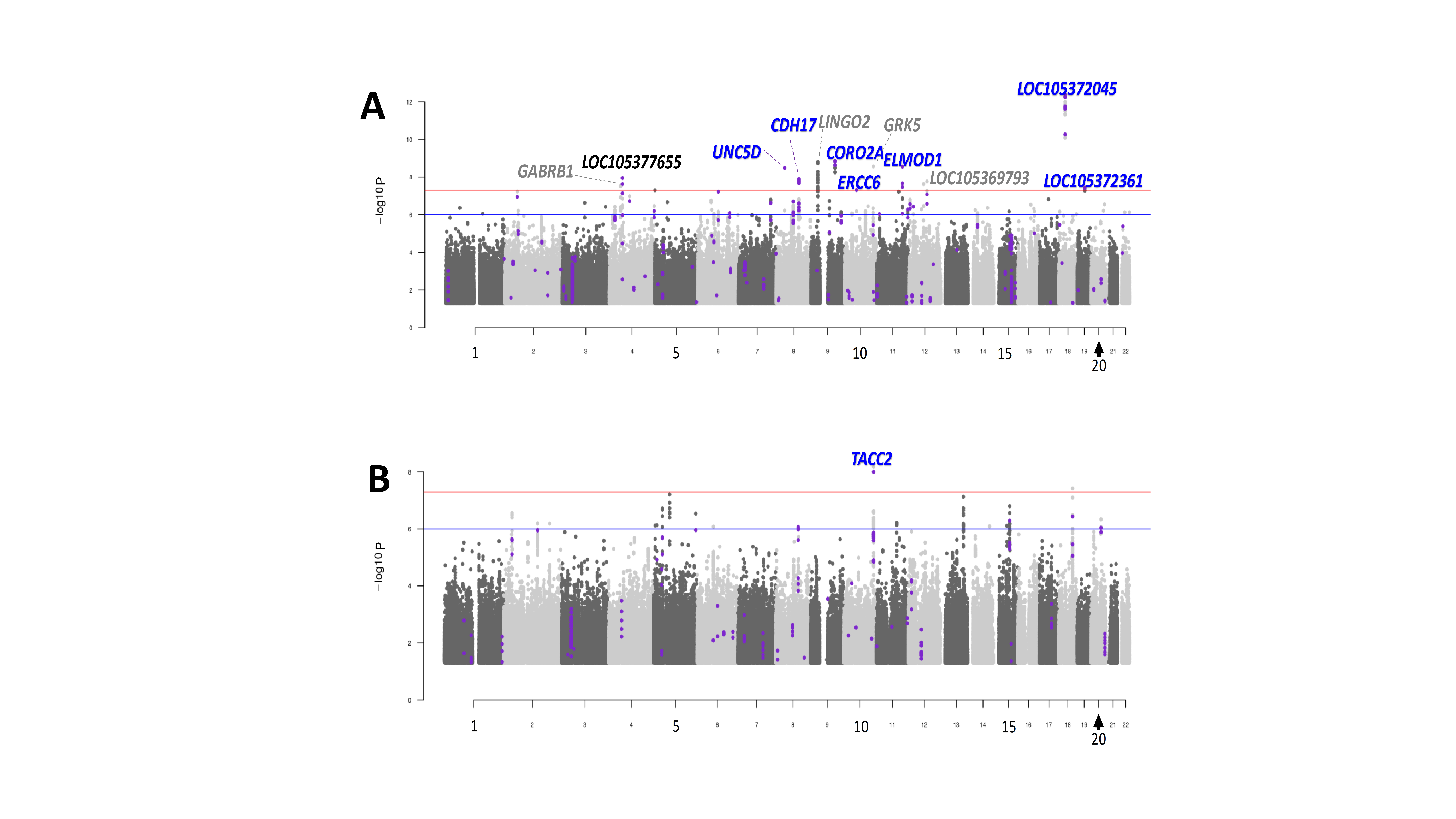

Supplement: S13 Fig — Manhattan plots of combined Stage 1 and Stage 2 meta-analysis for PP in current drinkers (A) and in light/heavy drinkers (B) in African ancestry. Novel loci are highlighted in blue. (TIF) [file pone.0198166.s015.tif]

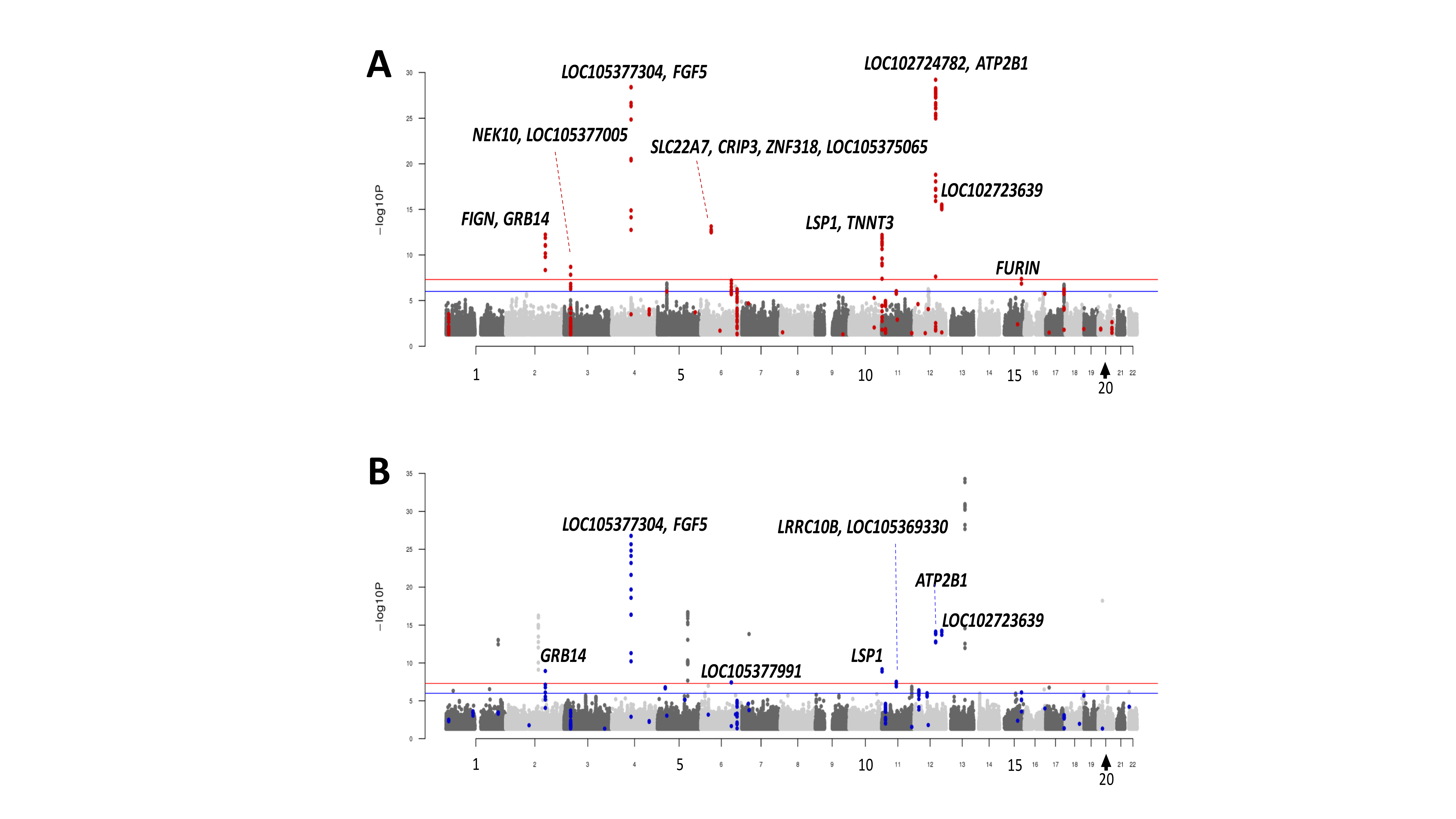

Supplement: S14 Fig — Manhattan plots of combined Stage 1 and Stage 2 meta-analysis for SBP (A) and DBP (B) in current drinkers in Asian ancestry. (TIF) [file pone.0198166.s016.tif]

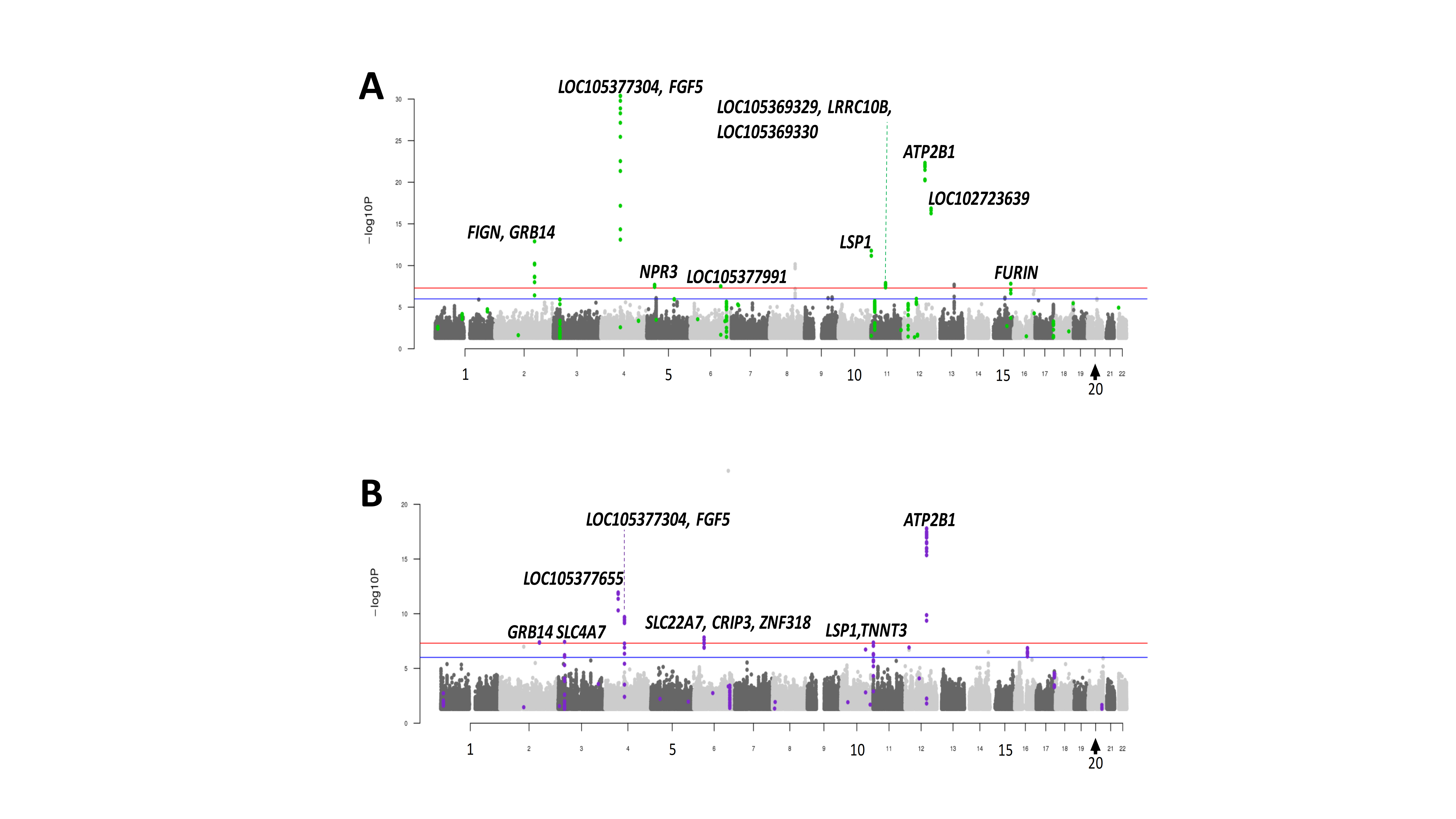

Supplement: S15 Fig — Manhattan plots of combined Stage 1 and Stage 2 meta-analysis for MAP (A) and PP (B) in current drinkers in Asian ancestry. (TIF) [file pone.0198166.s017.tif]

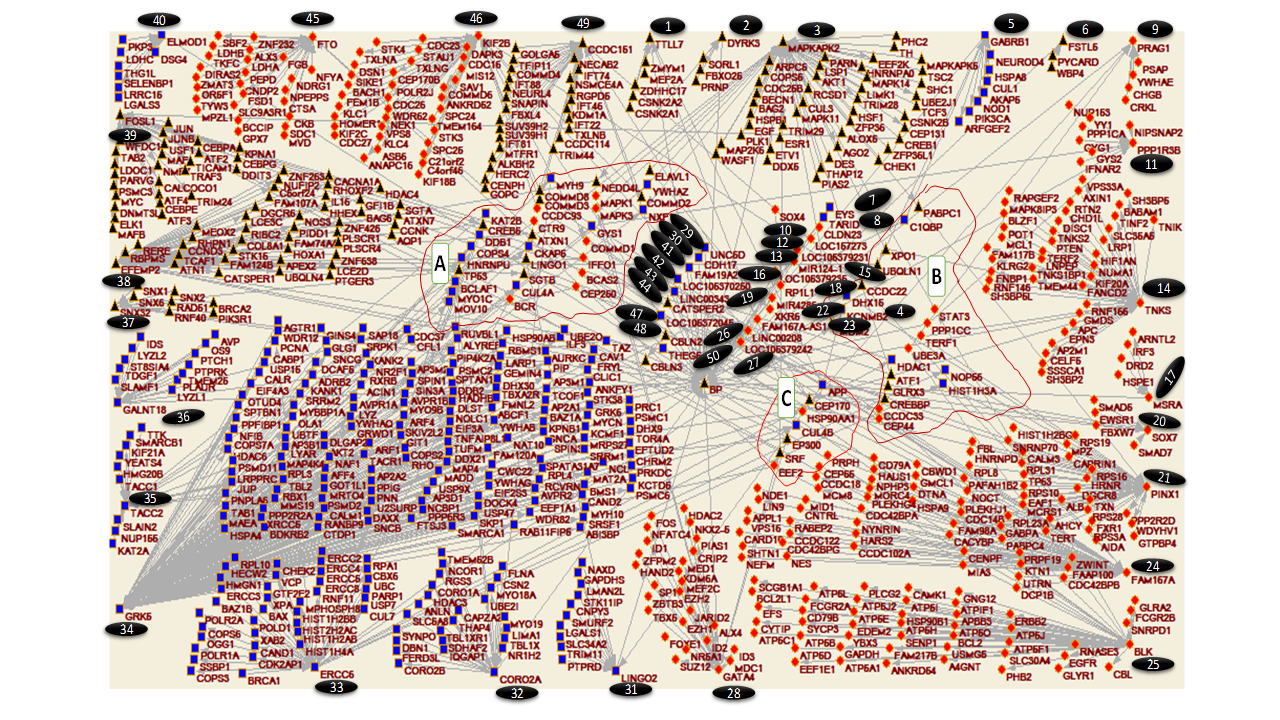

Supplement: S16 Fig — In the figure, ellipses in black represent all novel genes; ellipses in red represent novel from EA; squares in blue represent potential novel findings from African ancestry; and triangles in black from correlated-meta. Labeled with A and B free-hand circles are proteins that have two connections, while labeled within C are proteins that have three-five connections with our findings. APP interacts with five of our BP candidate novel genes TTLL7, SOX7, PINX1, LINGO2 and KCNMB2 (circle C). (TIF) [file pone.0198166.s018.tif]

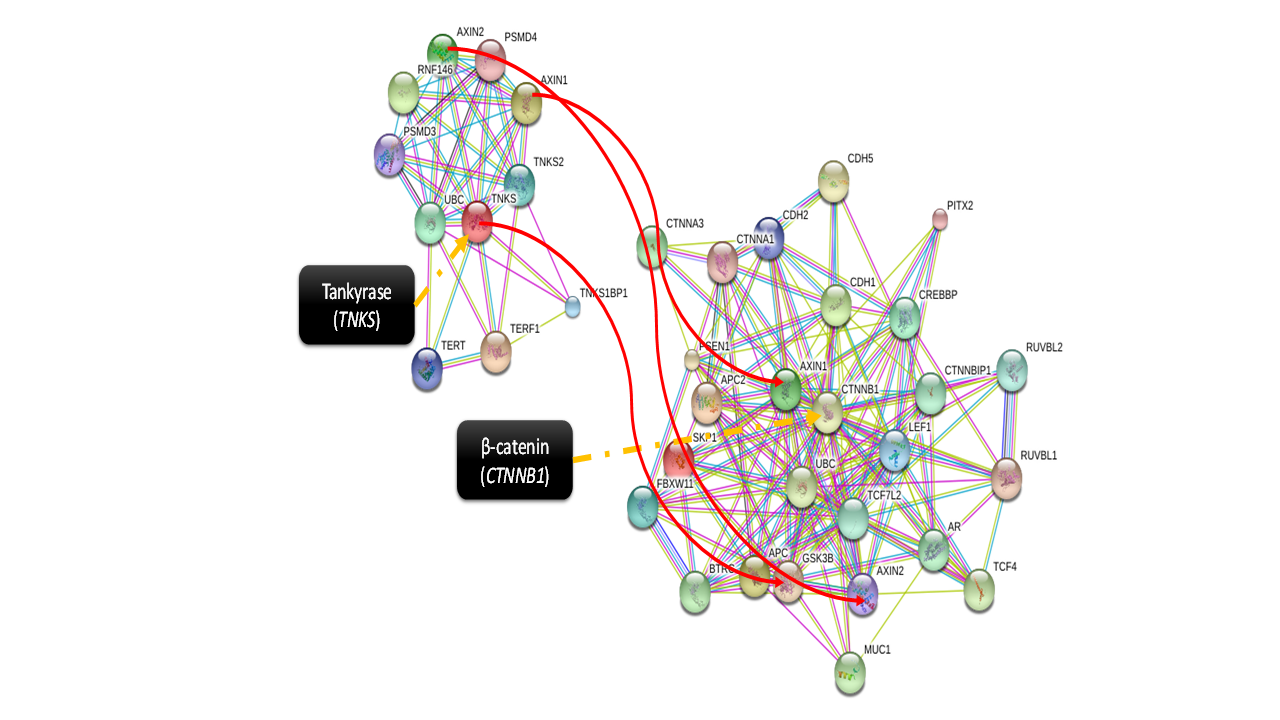

Supplement: S17 Fig — Tankyrase (from TNKS gene) and β-catenin (from CTNNB1 gene). (TIF) [file pone.0198166.s019.tif]

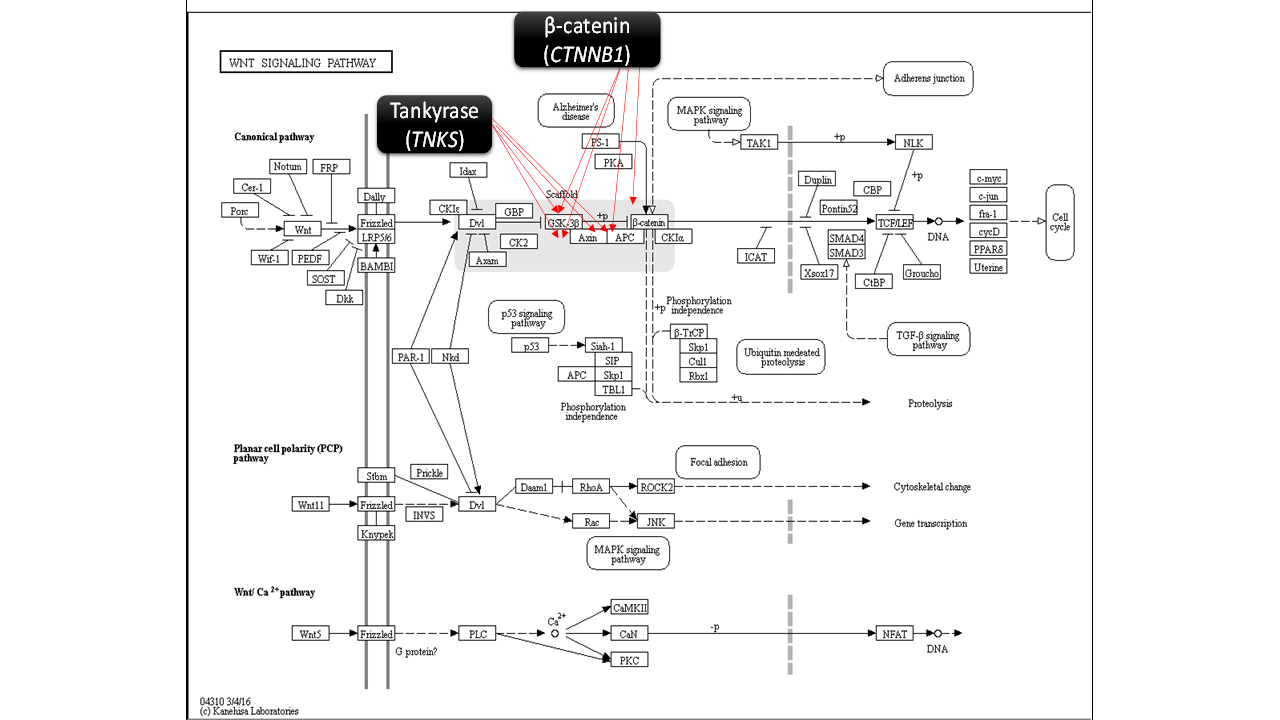

Supplement: S18 Fig — TNKS interacts with CTNNB1. (TIF) [file pone.0198166.s020.tif]
