# Supplementary material for: Novel genetic associations for blood pressure identified via gene-alcohol interaction in up to 570K individuals across multiple ancestries
Source: PLoS One. 2018 Jun 18;13(6):e0198166. doi: 10.1371/journal.pone.0198166 (PMC6005576; doi:10.1371/journal.pone.0198166)
Supplement: S15 Table — (DOCX) [file pone.0198166.s035.docx]

**S15 Table. Data analysis tools and databases**

| Software and Database | Web Resources | References |
| --- | --- | --- |
| Beagle version 4.1 | <https://faculty.washington.edu/browning/beagle/beagle.html> | Browning SR, Browning BL. Am J Hum Genet. 2007;81(5):1084-97. PMID: 17924348 |
| BIND | <http://baderlab.org/BINDTranslation> | Bader GD, Betel D, Hogue CW. BIND: the Biomolecular Interaction Network  Database. Nucleic Acids Res. 2003 Jan 1;31(1):248-50. PubMed PMID: 12519993 |
| BioGrid 3.4 | <http://thebiogrid.org/> | Chatr-Aryamontri A, Oughtred R, Boucher L, Rust J, Chang C, Kolas NK, O'Donnell L, Oster S, Theesfeld C, Sellam A, Stark C, Breitkreutz BJ, Dolinski K, Tyers M. **The BioGRID interaction database: 2017 update**. Nucleic Acids Res. 2016 Dec 14;2017(1) |
| EcoCyc | <http://www.ecocyc.org> | Keseler IM, et al. Nucleic Acids Res. 2017;45(D1):D543-D550. PMID: 27899573 |
| EasyQC | <http://www.uni-regensburg.de/medizin/epidemiologie-praeventivmedizin/genetische-epidemiologie/software/> | Winkler TW, et al. Nat Protoc. 2014;9(5):1192-212. PMID: 24762786 |
| GTEx | <https://www.gtexportal.org/> | Ferreira PG, et al. Nature Communications. 2018. 9: 490. PMID: 29440659 |
| GWAS3D | <http://jjwanglab.org/gwas3d> | Li MJ, et al. Nucleic Acids Res. 2013;41(Web Server issue):W150-8. PMID: 23723249 |
| HaploReg (v.4.1) | <http://www.broadinstitute.org/mammals/haploreg/haploreg.php> | Ernst J, et al. Nature. 2011;473(7345):43-9. PMID: 21441907;  Ward LD, Kellis M. Nucleic Acids Res. 2012;40(Database issue):D930-4. PMID: 22064851 |
| HPRD | <http://www.hprd.org/> | Keshava Prasad TS, et al. Nucleic Acids Res. 2009;37(Database issue):D767-72. PMID: 18988627 |
| Igraph 1.0.0 | [http://igraph.org](http://igraph.org/) | Gábor Csárdi, Tamás Nepusz: The igraph software package for complex network research. InterJournal Complex Systems, 1695, 2006. |
| IMPUTE2 | <http://mathgen.stats.ox.ac.uk/impute/impute_v2.html> | Howie BN, et al. PLoS Genet. 2009;5(6):e1000529. PMID: 19543373 |
| Literature Lab of ACUMENTA | [acumenta.com](http://www.acumenta.com) | Febbo PG, Mulligan MG, Slonina DA, Stegmaier K, Di Vizio D, Martinez PR, Loda M, Taylor SC. Literature Lab: a method of automated literature interrogation to infer biology from microarray analysis. BMC Genomics. 2007 Dec 18;8:461. PubMed PMID: 18088408; PubMed Central PMCID: PMC2244637. |
| 1000G Phase I Integrated Release Version 3 Haplotypes | <http://csg.sph.umich.edu/abecasis/mach/download/1000G.2012-03-14.html> | International HapMap Constortium. A second generation human haplotype map of over 3.1 million SNPs. Nature, 449:851-862. 2007. |
| HapMap Phase II | <https://www.genome.gov/10001688/international-hapmap-project/> | International HapMap Constortium. A second generation human haplotype map of over 3.1 million SNPs. Nature, 449:851-862. 2007. |
| MACH 1.0 | <http://www.sph.umich.edu/csg/abecasis/MaCH/> | Scott LJ, et al. Science 2007; 316:1341-5. PMID: 17463248 |
| Minimac | <https://genome.sph.umich.edu/wiki/Minimac> | Fuchsberger C, et al. 2015;31(5):782-4 PMID: 25338720; Howie B, et al. Nat Genet. 2012;44(8):955-9. PMID: 22820512 |
| MetaCore / GeneGO | [portal.genego.com](https://portal.genego.com) | https://clarivate.com/products/metacore/ |
| METAL | <https://genome.sph.umich.edu/wiki/METAL_Documentation> | Willer CJ, Li Y, Abecasis GR. METAL: fast and efficient meta-analysis of  genomewide association scans. Bioinformatics. 2010 Sep 1;26(17):2190-1. doi:  10.1093/bioinformatics/btq340. Epub 2010 Jul 8. PubMed PMID: 20616382 |
| NCBI | <https://www.ncbi.nlm.nih.gov> |  |
| NCBI Entrez gene | <https://www.ncbi.nlm.nih.gov/gene/> |  |
| NCBI dbSNP | [ncbi.nlm.nih.gov/snp/](http://www.ncbi.nlm.nih.gov/snp/) |  |
| RegulomeDB Version 1.1 | <http://regulome.stanford.edu/> | Xie D, et al. Cell. 2013;155(3):713-24. PMID: 24243024  Boyle AP, et al. Nature. 2014;512(7515):453-6. PMID: 25164757 |
| ProbABEL | <http://www.genabel.org/packages/ProbABEL> | Aulchenko YS, et al. BMC Bioinformatics. 2010;11:134. PMID: 20233392 |
| GenABEL | <http://www.genabel.org/packages/GenABEL> | Aulchenko YS, et al. Bioinformatics. 2007;23(10):1294-6. PMID: 17384015. |
| MixABEL | <http://www.genabel.org/MixABEL/MixABEL-package.html> | Aulchenko YS, et al. Bioinformatics. 2007;23(10):1294-6. PMID: 17384015. |
